# Design, Synthesis, and Biological Evaluation of Small-Molecule-Based Radioligands with Improved Pharmacokinetic Properties for Imaging of Programmed Death Ligand 1

**DOI:** 10.1021/acs.jmedchem.3c01355

**Published:** 2023-12-01

**Authors:** Fabian Krutzek, Cornelius K. Donat, Martin Ullrich, Sven Stadlbauer

**Affiliations:** † Helmholtz-Zentrum Dresden-Rossendorf, Institute of Radiopharmaceutical Cancer Research, Bautzner Landstraße 400, 01328 Dresden, Germany; ‡ Faculty of Chemistry and Food Chemistry, School of Science, Technische Universität Dresden, Mommsenstraße 4, 01069 Dresden, Germany

## Abstract

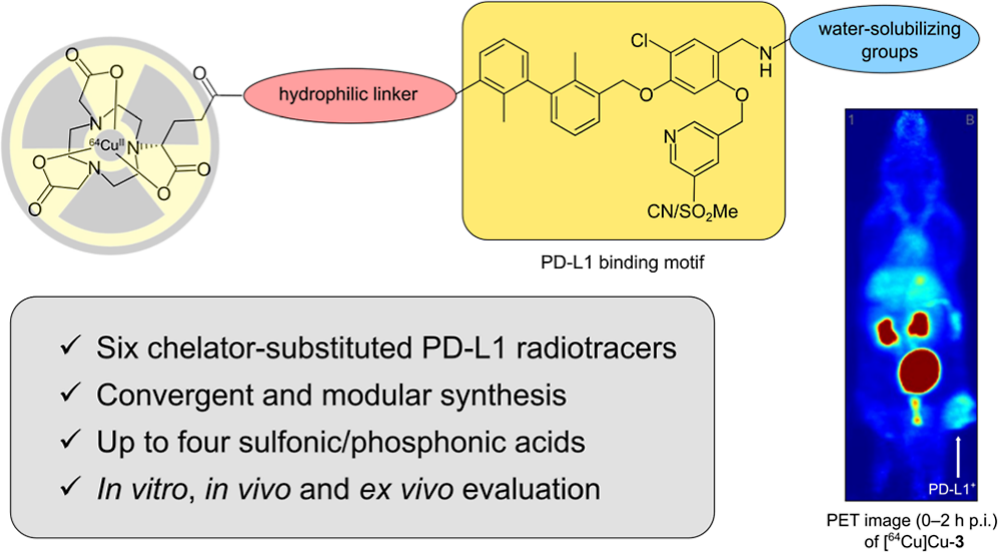

Small molecules offer some advantages for developing positron emission tomography (PET) tracers and are therefore a promising approach for imaging and therapy monitoring of programmed death ligand 1 (PD-L1) positive tumors. Here, we report six biphenyl PD-L1 radioligands using the NODA-GA-chelator for efficient copper-64 complexation. These radioligands contain varying numbers of sulfonic and/or phosphonic acid groups, serving as hydrophilizing units to lower the log *D*_7.4_ value down to −4.28. The binding affinities of compounds were evaluated using saturation binding and a real-time binding assay, with a highest binding affinity of 21 nM. Small-animal PET imaging revealed vastly different pharmacokinetic profiles depending on the quantity and type of hydrophilizing units. Of the investigated radioligands, [^64^Cu]Cu-**3** showed the most favorable kinetics in vitro. This was also found in vivo, with a predominantly renal clearance and a specific uptake in the PD-L1-overexpressing tumor. With further modifications, this compound could be a promising candidate for the imaging of PD-L1 in the clinical setting.

## Introduction

Within the tumor microenvironment, immune checkpoints such as the programmed cell death protein PD-1 (CD279) and its ligand programmed death ligand 1 (PD-L1) (CD274) play a crucial role in regulating the immune response. In the homeostatic context, this limits autoimmune or immune-adverse effects following an inflammatory response. Under malignant conditions, tumor cells have developed strategies to evade the immune response by overexpressing the corresponding targets, e.g., PD-L1. Therefore, the PD-1/PD-L1 axis is a highly attractive target for the therapy of solid cancers through use of the so-called checkpoint inhibitors, which are able to reactivate the local immune response. However, only an average of 30% of patients respond to a checkpoint inhibitor monotherapy.^1−3^ Until now, patient stratification and therapy decisions have relied on immunohistochemical methods, which are less reliable due to the heterogeneity of PD-L1 expression among and within tumor lesions. In contrast, noninvasive molecular imaging techniques such as positron emission tomography (PET) and single-photon emission computed tomography (SPECT) can fully address the issue of PD-L1 heterogeneity over time. In addition, PET and SPECT allow for a noninvasive whole-body monitoring, thus avoiding repeated, painful biopsies for patients. A diagnostic tool for supporting therapy decisions is therefore highly sought after in the field of immune checkpoint inhibitor (ICI) therapy.^4^ Different radiotracer classes, such as antibodies,^5−10^ nanobodies,^11−14^ affibodies,^15^ or adnectins,^16^ targeting PD-L1 have been reported and are partly undergoing clinical trials.^17−19^ These high-molecular-weight compounds exhibit advantageously high accumulation in tumor tissues,^20^ but the larger constructs such as antibodies, also possess long circulation times, thus causing an additional radiation burden for the patients. Their relatively high immunogenicity can potentially cause adverse immunological effects, which are difficult to manage.^21^ Additionally, the manufacturing and healthcare costs of antibodies are generally higher.^22^ In comparison, peptides and nonpeptide small molecules are considered as favorable imaging agents. They possess higher tissue and tumor penetration, a relatively low immunogenicity and short clearance times, therefore producing higher imaging contrast within short time frames and are synthetically easily accessible.^20,23^ In recent years, a number of peptide-based PD-L1 radiotracers have been developed and preclinically evaluated.^24−28^ Especially the cyclic peptide WL12, labeled with copper-64, has already been evaluated in a first clinical study.^29^ While a large number of small molecules have been reported as inhibitors for the treatment of PD-L1-positive cancers,^30−33^ only a few radiolabeled small-molecule-based tracers have been described so far.^34−37^

To reduce the impact of the radiolabel on the physicochemical and biological properties of a small molecule, the labeling site has to be carefully selected. In addition, the pharmacokinetic properties of the resulting radioligand must be adjusted, e.g., by introducing hydrophilic moieties into lipophilic lead structures to achieve favorable renal rather than undesirable hepatobiliary secretion.^38−40^ Hydrophilic chelators in combination with hydrophilic linkers can counterbalance the lipophilicity of a small molecule.^41−44^

Recently, we have reported on the development of new PD-L1-targeting PET tracers^45^ based on biphenyl small-molecule inhibitors. We modified the existing PD-L1 inhibitors with strongly water-soluble sulfonic acid moieties in the solvent-exposed region of the PD-L1 protein, along with a hydrophilic linker and DOTA chelator for labeling with copper-64. These new radioligands showed high binding affinity toward PD-L1 in cell-based assays but exhibited pronounced albumin binding. Small-animal PET/CT in mice bearing PD-L1-overexpressing and mock tumors showed moderate to low tumor uptake together with primarily hepatobiliary clearance. Due to high affinity toward albumin, all radioligands showed an unexpected long circulation time.

## Results

### Rationale

Based on the in vitro and in vivo results of our previously reported series of nine PD-L1 radioligands,^45^ we designed, synthesized, and evaluated a new set of six compounds.

**Figure 1:**
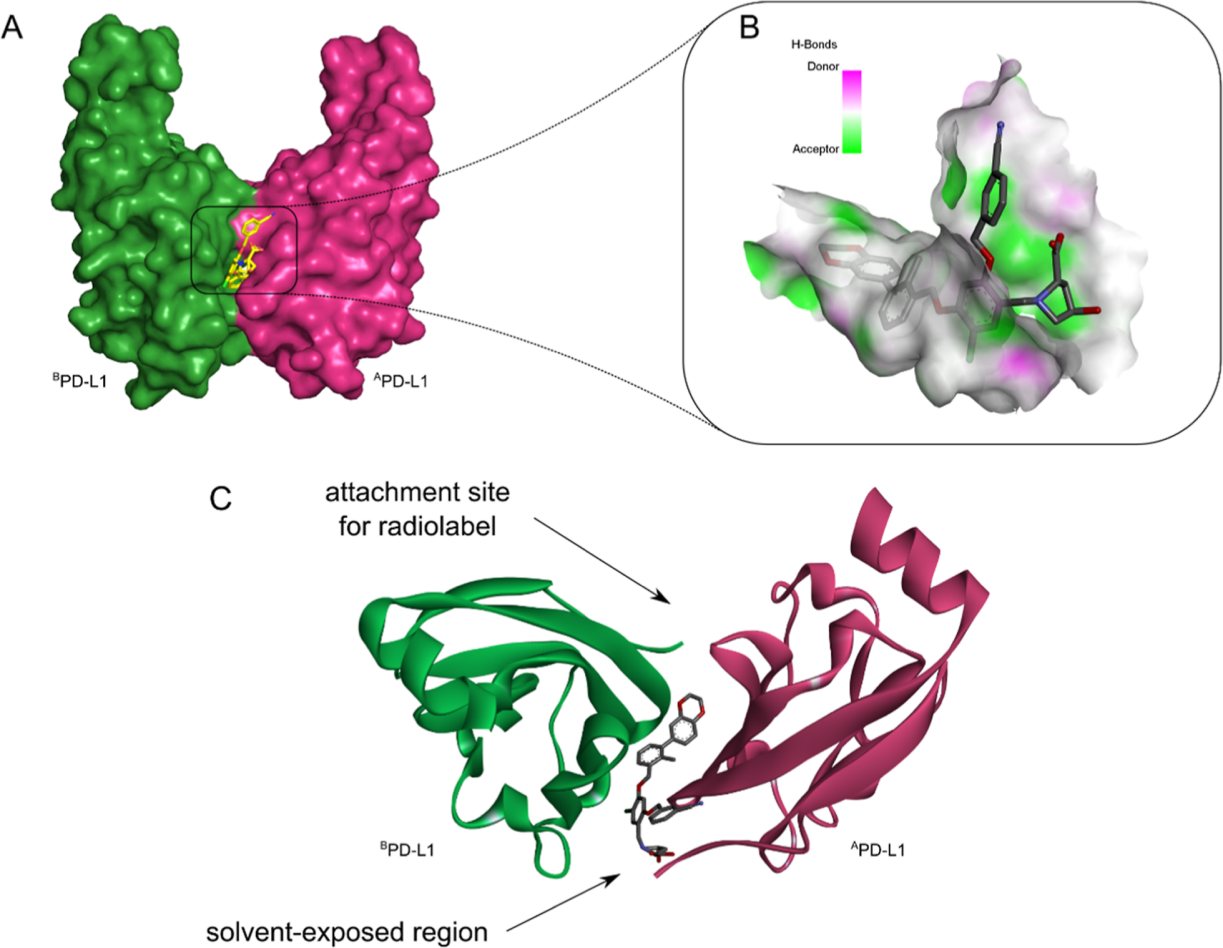
(A) Surface model of dimeric PD-L1 proteins (^A^PD-L1 and ^B^PD-L1) with inhibitor BMS-1166. (B) Receptor surface model with inhibitor BMS-1166 displaying H-bonds donor and acceptor capacity in the solvent-exposed region and hydrophobic tunnel. (C) Top view of the PD-L1 dimer (^A^PD-L1 and ^B^PD-L1) in the cartoon model. The crystal structure of the dimeric PD-L1 protein with the BMS-1166 inhibitor was taken from the PDB databank (PDB code: 6R3K).

From the cocrystal structure of inhibitor BMS-1166 with the PD-L1 protein, reported by Holak et al. in 2017, we rationalized the design of our new radioligands.^46^ It is known that small-molecule inhibitors induce the dimerization of two PD-L1 proteins (^A^PD-L1 and ^B^PD-L1, Figure 1A,C).^33^ As a result, a hydrophobic tunnel is formed, which is occupied by the biphenyl core of the inhibitor (Figure 1B). On the contrary, the amino acid moiety of the inhibitor reaches into the solvent-exposed region of the protein, which is dominated by hydrophilic interactions between inhibitor and protein. This part of the protein exhibits a high H-bonding capacity, indicated by the green area in Figure 1B. In our previously reported radioligands, the hydroxy-proline moiety of BMS-1166 was replaced with a sarcosine spacer. Now in order to enhance the interaction, an H-bond-donating acid group was introduced in the current ligands. The top view of the protein (Figure 1C) indicates a possible attachment site for the radiolabel at the biphenyl core. We pursued this approach already in our first studies, but now, we aimed to extend the linker moiety to achieve a higher spatial distance of the chelator with the hydrophobic tunnel.

Besides a small modification within the binding motif, we aimed to improve the pharmacokinetic profile of the radioligands. In vivo, the previous compounds showed unexpected long circulation times in the range of several hours until the tumor uptake reached its maximum, as shown by small-animal PET imaging experiments. This could be attributed to the sulfonic acids, which are known to be albumin binders. It was shown that the substitution pattern of several sulfonic acids can influence the albumin binding capacity.^47^ Therefore, we replaced the geminal bis(sulfonic) acid moiety with two linearly arranged sulfonic acids, leading to greater spatial distance. Additionally, we partly replaced sulfonic acids with phosphonic acids, which are not known to interact with albumin. Despite exhibiting log *D*_7.4_ values between −2.73 and −3.50, the previous radioligands still showed uptake in intestines and liver, thus indicating a hydrophobic nature. By incorporating additional hydrophilizing units, we aimed to further decrease the log *D*_7.4_ values and improve the pharmacokinetic profile. In addition, we also replaced DOTA with NODA-GA to avoid the transchelation of copper-64 by liver enzymes.

**Scheme 1:**
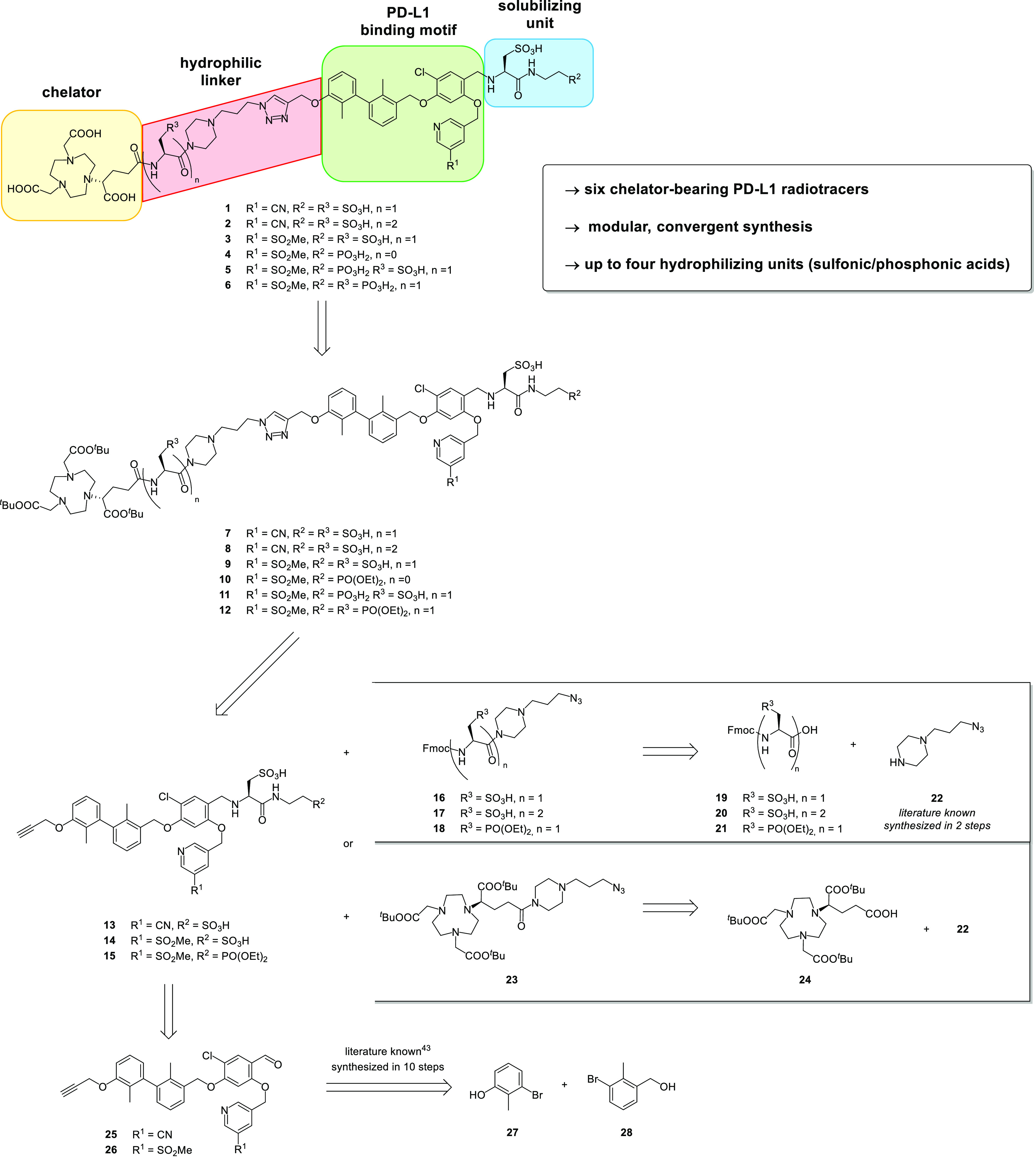
General Structure of the PD-L1 Radioligands and Their Retrosynthetic Analysis

The binding motif (Scheme 1, green) consists of a dimethyl biphenyl unit linked to the central, fourfold substituted chloroaryl, which in turn bears a pyridine moiety substituted with a nitrile or a sulfonyl moiety. At the eastern part of the molecule, we introduced the solubilizing unit (Scheme 1, blue) with either two sulfonic acids or a sulfonic and phosphonic acid. The linker structure (Scheme 1, red)—a piperazine-based linker with no, one, or two hydrophilizing units—connects the NODA-GA-chelator (Scheme 1, yellow) with the binding motif. These structural modifications resulted in a set of six radioligands, which were synthesized according to our previously established convergent synthetic strategy using a modular approach.^45^

The final PD-L1 radioligands were obtained by deprotection of the carboxylic and phosphonic acids. Compound **4** was left without a hydrophilizing unit (*n* = 0) in the linker structure. In this case, the *tert*-butyl-protected NODA-GA-chelator is already attached to the piperazine moiety, which is introduced by copper-catalyzed azide–alkyne cycloaddition (CuAAC-reaction). The linker–chelator system is obtained by amide bond formation of the NODA-GA-chelator employing a literature-known piperazine linker.^48^ In case of one or two hydrophilizing units in the linker structure (*n* = 1 or 2), the NODA-GA-chelator is conjugated after attaching the linker via a CuAAC reaction to the binding motif. The linker is synthesized by amide bond formation of the piperazine moiety with the corresponding Fmoc-protected l-cysteic acid or l-phosphonoalanine. The binding motif itself also contains two hydrophilizing units that are synthesized by a reductive amination of the previously reported aldehydes **25** and **26** with l-cysteic acid followed by amide bond formation with taurine or diethyl-protected ciliatine.^49^

### Synthesis

**Scheme 2:**
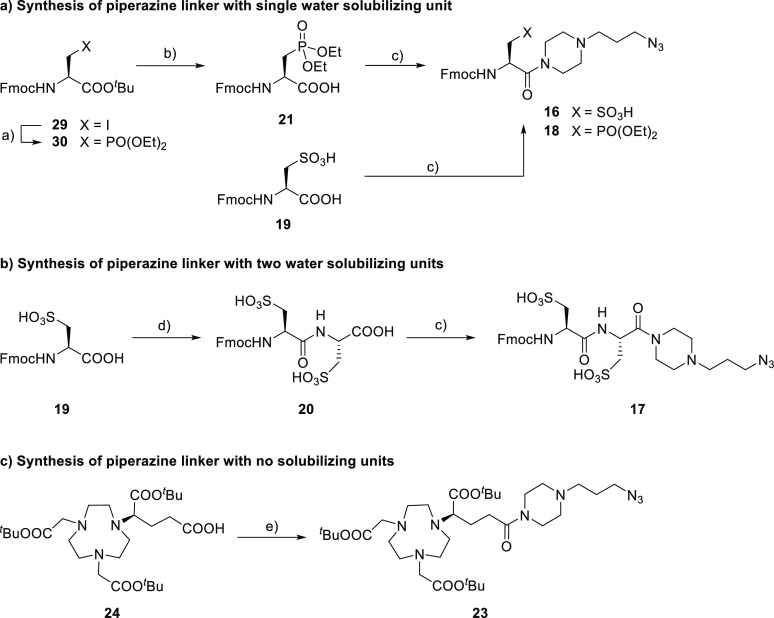
Synthesis of Various Linker Structures P(OEt)_3_, argon, 140 °C, and 16 h; ^b^CH_2_Cl_2_/TFA (1:1), 0 °C to r.t., 5 h, and 57% over two steps; ^c^**22**, HTBU, HOBt, abs. pyridine, abs. DMF, 0 °C to r.t., and 70% (**16**), 57% (**18**), and 52% (**17**); ^d^HOSu, DCC, HOBt, abs. DMF, 0 °C to r.t., 16 h, then l-cysteic acid, Na_2_CO_3(aq)_ (6.8%), 0 °C to r.t., 3 h, and 53% over two steps; ^e^**22**, HBTU, abs. DIPEA, abs. DMF, r.t., 16 h, and 95%.

The development of PD-L1 radioligands started with the synthesis of the linker structures (Scheme 2). For the linker **18** with one phosphonate group, the synthesis commenced with an Arbuzov reaction of commercially available Fmoc-3-iodo-l-alanine *tert*-butyl ester in neat triethyl phosphite. All volatiles were removed by vacuum distillation, and the *tert*-butyl deprotection was performed with TFA. After column chromatography, the diethyl phosphonate derivate **21** was obtained in a 57% yield over two steps. This compound and its sulfonic acid analogue **19** were converted into the hydrophilic linkers **16** and **18** in 70 and 57% yields, respectively, through coupling with 1-(3-azidopropyl)piperazine (**22**).

The linker structure bearing two sulfonic acids was synthesized by adapting a previously reported strategy.^50,51^ After in situ formation of Fmoc-protected l-cysteic acid NHS-ester, the intermediate was reacted with l-cysteic acid under Schotten–Baumann conditions in aqueous sodium hydrogen carbonate solution, affording **20** in a 53% yield. Subsequent coupling with piperazine moiety **22** provided linker **17** in a 52% yield. The most hydrophobic linker **23** was accessed by coupling piperazine **22** with commercially available (*R*)-NODA-GA(*t*-Bu)_3_ (**24**) with HBTU/DIPEA in DMF. Purification was achieved with RP-HPLC and detection was performed at 220 nm, affording structure **23** in a 95% yield.

**Scheme 3:**
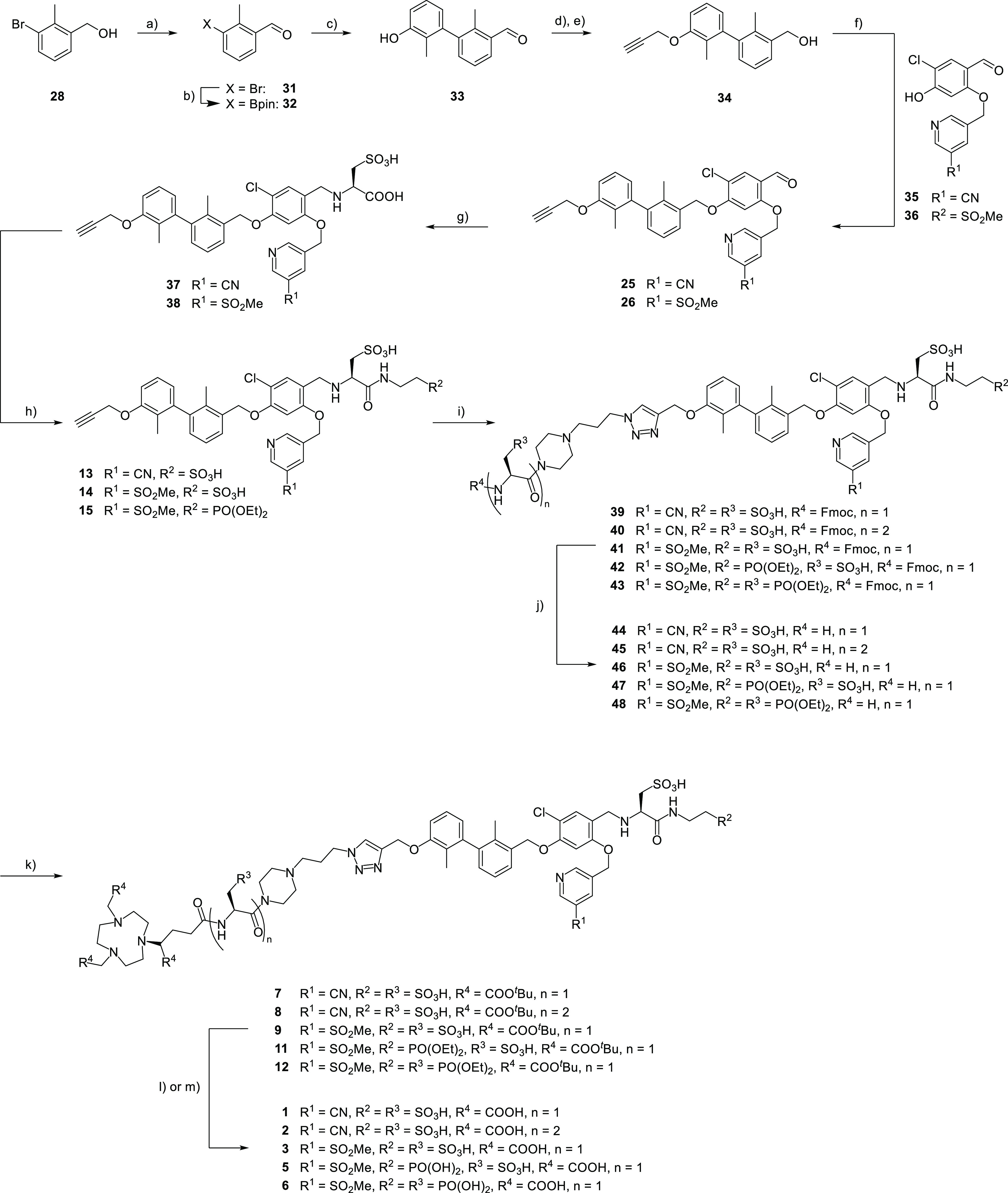
Synthesis of PD-L1 Radioligands Bearing One or Two Solubilizing Units in the Linker Structure MnO_2_, DCM, rt, 16 h, and 65%. ^b^B_2_pin_2_, Pd(dppf)Cl_2_, NaOAc, argon, DMF, 110 °C, 16 h, and 67%. ^c^3-Bromo-2-methylphenol, Pd(PPh_3_)_4_, K_2_CO_3_, argon, toluene/EtOH/H_2_O (20:0.5:0.1), 100 °C, 16 h, and 90%. ^d^Propargyl bromide, K_2_CO_3_, abs. DMF, rt, 16 h, and 91%. ^e^NaBH_4_, abs. MeOH/DCM (1:1), 0 °C to rt, 3 h, and 86%. ^f^DEAD, PPh_3_, abs. DMF, 0 °C to rt, 16 h, and 86%. ^g^l-Cysteic acid, NaBH_3_CN, abs. MeOH/DMF (1:1), 0 °C to r.t., 16 h, and 89% (**37**) and 87% (**38**); ^h^taurine or diethyl ciliatine, HTBU, HOBt, abs. DIPEA, abs. DMF, 0 °C to r.t., 16 h, and 87% (**13**), 86% (**14**), and 60% (**15**). ^i^**16**, **17**, or **18**, sodium ascorbate, CuSO_4_, THPTA, H_2_O/*t*-BuOH (1:1), r.t., and 16 h; ^j^NaN_3_, abs. DMF, 60 °C, 3 h, and 53% (**44**), 41% (**45**), 69% (**46**), 87% (**47**), and 50% (**48**) over two steps; ^k^(*R*)-NODA-GA(*t*-Bu)_3_, HBTU, HOBt, abs. DIPEA, abs. DMF, 0 °C, to r.t., 16 h, and 63% (**11**), 89% (**12**); ^l^TFA/CH_2_Cl_2_/TES/H_2_O (20:20:8:7), 0 °C to r.t., 40 h, and 67% (**1**), 42% (**2**), and 51% (**3**) over two steps; ^m^TMSBr, abs. DMF, r.t., 40 h, then TFA/CH_2_Cl_2_/TES/H_2_O (20:20:8:7), 0 °C to r.t., 40 h, and 57% (**5**), and 53% (**6**) over two steps.

**Scheme 4:**
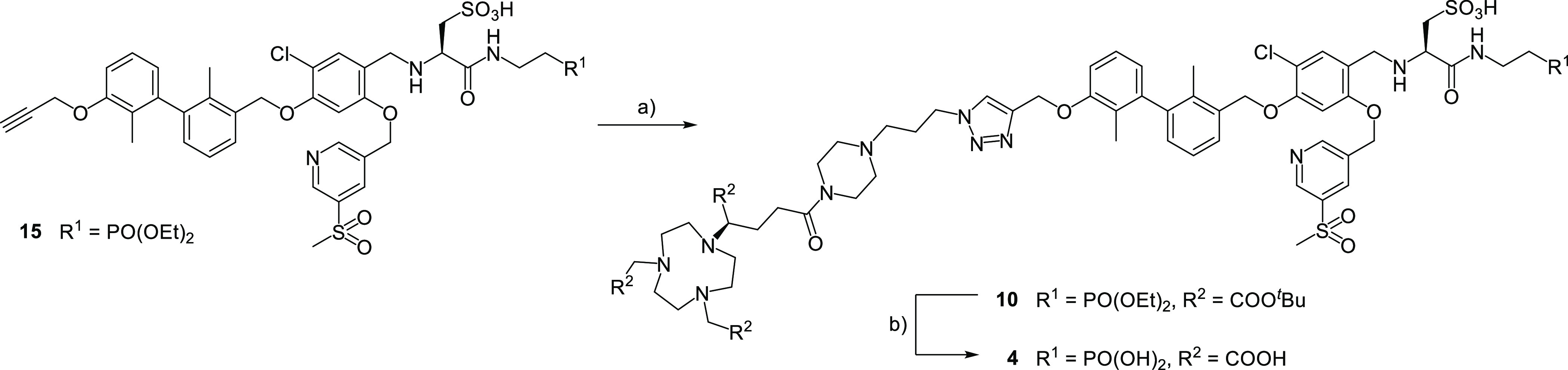
Synthesis of PD-L1 Radioligands Bearing No Solubilizing Unit in the Linker Structure **23**, sodium ascorbate, CuSO_4_, THPTA, H_2_O/*t*-BuOH (1:1), r.t., 16 h, and 41%; ^b^TMSBr, abs. DMF, r.t., 40 h, then TFA/CH_2_Cl_2_/TES/H_2_O (20:20:8:7), 0 °C to r.t., 40 h, and 54% over two steps.

With all linker structures in hand, conjugation with the PD-L1 binding motif was attempted (Scheme 3). We selected our two previously described binding motifs with a nitrile and methylsulfonyl group at the pyridine moiety.^45^ Briefly, the synthesis started from bromobenzyl alcohol **28**, which was reduced to the aldehyde, then borylated, followed by Suzuki coupling to form the biphenyl core **33**. Subsequent attachment of the alkyne moiety and reduction to the alcohol for Mitsunobu reaction with the central aryl moiety provided **25** and **26**. These precursors were modified with the hydrophilizing moieties in the solubilizer unit. To this end, reductive amination with l-cysteic acid and sodium cyanoborohydride in a DMF/MeOH mixture was performed, affording derivatives **37** and **38** in excellent yields (89 and 87%, respectively). In the next step, subjection of either taurine or diethyl-protected ciliatine^49^ to HBTU/DIPEA led to the three derivatives **13** (87%), **14** (86%), and **15** (60%). With that, the stage was set for the CuAAC reaction using CuSO_4_ and sodium ascorbate for attaching the three different linkers **16**, **17**, and **18**. The purified compounds **10**, **11**, and **12** containing protected phosphonic acids were subjected to a McKenna reaction (TMSBr in DMF) to remove the alkyl groups.^52^ The reactions were conducted in NMR tubes for monitoring the reaction progress with ^31^P NMR spectroscopy. Complete conversion to the corresponding TMS-esters was achieved within 48 h, followed by ester hydrolysis in MeOH. After removing the solvents, the substrates were subjected to *tert*-butyl-deprotection and purified by HPLC, providing the final products **1** (67%), **2** (42%), **3** (51%), **4** (54%, Scheme 4), **5** (57%), and **6** (53%).

### Radiochemistry

#### Radiolabeling

Radiolabeling of PD-L1 ligands was performed with [^64^Cu]Cu^2+^ at pH 4.5 in a 1 M aqueous HEPES solution. Incubation at 50 °C for 10 min resulted in quantitative labeling confirmed by radio-TLC. The molar activities ranged between 10 and 15 GBq·μmol^–1^.

#### Determination of log *D*_7.4_ Values

For assessing the lipophilicity of the ^64^Cu-labeled PD-L1 radioligands, distribution coefficients (log *D*_7.4_ values) were determined using the shake flask method between *n*-octanol and PBS buffer (pH 7.4). The obtained values are summarized in Table 1 and range between −3.07 for [^64^Cu]Cu-**4** to −4.28 for [^64^Cu]Cu-**6**. Thus, all compounds in this series are sufficiently water-soluble and are promising with regard to the desired renal excretion.

**Table 1:** Summary of In Vitro Data for ^64^Cu-Labeled PD-L1 Radioligands and Their Respective log *D*_7.4_ Values

compound	*K*_D_ (nM)^a^	*B*_max_ [pmol·mg^–^^1^]^a^	log *D*_7.4_^b^
[^64^Cu]Cu-**1**	80.5 ± 5.02	7.05 ± 0.99	–4.00 ± 0.14
[^64^Cu]Cu-**2**	199.0 ± 23.8	9.66 ± 0.58	–4.15 ± 0.09
[^64^Cu]Cu-**3**	93.7 ± 10.8	8.51 ± 0.48	–3.80 ± 0.02
[^64^Cu]Cu-**4**	82.4 ± 7.42	10.7 ± 1.34	–3.07 ± 0.02
[^64^Cu]Cu-**5**	112.0 ± 11.0	19.1 ± 0.67	–3.81 ± 0.08
[^64^Cu]Cu-**6**	532.8 ± 76.6	10.3 ± 0.74	–4.28 ± 0.08

a Affinity (dissociation constant *K*_D_) and maximum number of binding sites (*B*_max_) as determined under identical conditions in a saturation binding assay using live PC3, PD-L1 overexpressing cells. Data are expressed as mean ± (SD), derived from at least three independent experiments (each in triplicate).

b Data are expressed as mean ± SD, derived from three separate shaking flask experiments.

### Stability Studies

#### Kinetic Stability in PBS Buffer (pH 7.4)

The kinetic stability of all ^64^Cu-labeled ligands was investigated in PBS buffer solution (pH 7.4) by incubation at room temperature and monitoring with radio-HPLC up to 48 h. The obtained radiochromatograms (Figure S114A–C, system C) of compounds [^64^Cu]Cu-**3**, [^64^Cu]Cu-**4**, and [^64^Cu]Cu-**5** show a major peak at ∼13.2–13.6 min originating from the intact radioligands (>99% at all three time points). During the time course, no changes in peak number/shape and retention time were observed, proving the stability of the compounds. The radio-HPLC chromatogram (Figure S114D, system D) of compound [^64^Cu]Cu-**6** shows a major peak at ∼6.7 min, indicating the intact radiotracer (>99%) as well. At the 48 h time point, however, a shoulder appears with a slightly higher retention time (∼7.0 min), indicating minor decomposition (>94% intact radiotracer).

#### Proteolytic Stability in Human Serum

Potential degradation of the ^64^Cu-labeled radiotracers by proteolysis was investigated by incubation in human serum at 37 °C for up to 48 h. At three different time points (1, 24, and 48 h), a sample was withdrawn, and serum proteins were precipitated. The radio-HPLC chromatograms of all ^64^Cu-labeled radiotracers (Figure S111A–D, system D) show a major peak at ∼6.4–6.7 min indicating the radiotracers. After 1 h, the radiotracers [^64^Cu]Cu-**3**–**6** are 94.1, 98.3, 94.0, and 96.3% intact, respectively. While for [^64^Cu]Cu-**3**–**5**, no further decomposition at the 48 h time point is observed, [^64^Cu]Cu-**6** remains 93.4% intact.

### In Vitro Experiments

#### Target Specificity of Employed Cells

Using a monoclonal antibody against human PD-L1, FACS showed stable overexpression of the target by over 99% of analyzed PC3 PD-L1 cells. In contrast, cells transduced with a mock construct or PC3 wild-type cells did not show a substantial expression of human PD-L1 (Figure S116A–C). These findings are corroborated by immunohistochemical analysis of the xenograft tissue, with tumors grown from PC3 PD-L1 cells showing uniform immunoreactivity (Figure S116D,F). Tumors grown from PC3 mock cells were basically devoid of any immunoreactivity, with a few single cells showing plasma staining across the tissue (Figure S116E,G).

However, it has to be noted that a basal PD-L1 expression was observed in FACS analysis of PC3 cells previously.^53^ While our data indicate only negligible basal PD-L1 expression by PC3 wild-type and mock-transduced cells, it cannot be ruled out completely.

#### Saturation Binding Assays

For assessing the binding affinity of compounds [^64^Cu]Cu-**1**–**6**, PC3 cells transduced with PD-L1 were used. Dissociation constants in equilibrium (*K*_D_) and maximum number of binding sites (*B*_max_) were determined in a saturation binding assay. The obtained results are summarized in Table 1, and iterative curve fittings of all compounds are shown in Figure S117. The assays were performed with the addition of 2.5% bovine serum albumin (BSA) in the medium to prevent unspecific binding to plastic wells, as reported previously for our first radioligands.^1^

**Figure 2:**
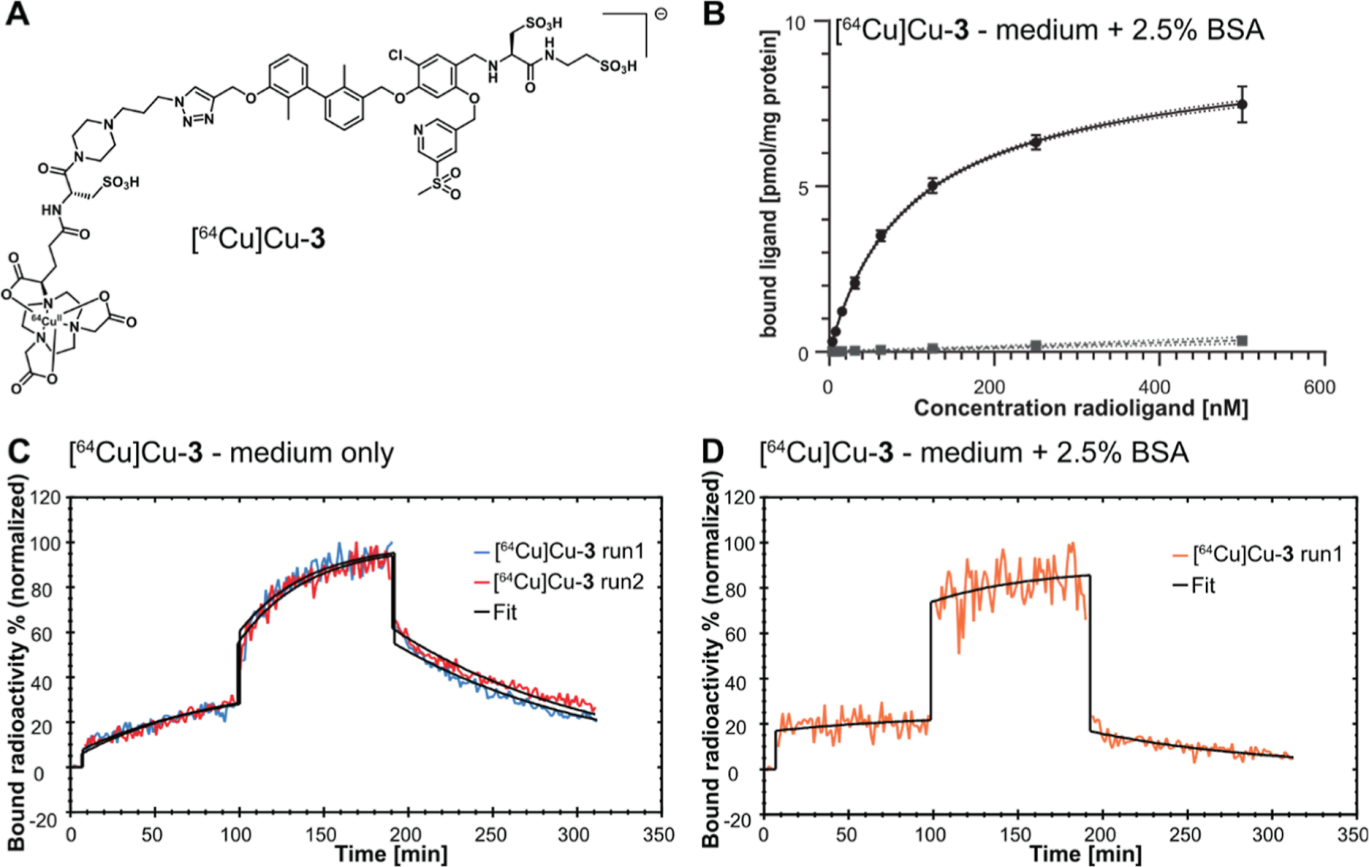
(A) ^64^Cu-complex of radioligand **3**. (B) Nonlinear iterative curve fitting of saturation binding experimental data for [^64^Cu]Cu-**3** (*n* = 3 independent experiments). Total binding is represented by the solid black curve (with the dotted line showing the 95% confidence interval), while the solid gray line (dotted line: the 95% confidence interval) represents nonspecific binding. Real-time radioligand binding (trace) of [^64^Cu]Cu-**3**, in the absence (C) and presence of BSA (D).

Using the saturation binding assay, the investigated compounds can be arbitrarily classified into the *K*_D_ range below 100 nM and of 100–250 nM and above (Table 1 and Figure S117). Compounds [^64^Cu]Cu-**1**, [^64^Cu]Cu-**3** (Figure 2A,B), and [^64^Cu]Cu-**4** fall into the first category, showing only minor differences. Compounds [^64^Cu]Cu-**2** and [^64^Cu]Cu-**5** show a more or less pronounced loss of binding affinity, with [^64^Cu]Cu-**6** showing the lowest (532 nM). The range for the maximum number of binding sites (*B*_max_) was relatively small, with 7.05 pmol·mg^–1^ for [^64^Cu]Cu-**1** to 10.7 pmol·mg^–1^ for ([^64^Cu]Cu-**4**). In contrast, compound [^64^Cu]Cu-**5** showed the highest *B*_max_ value of 19.1 pmol·mg^–1^

#### LigandTracer

Real-time radioligand binding using the ligand tracer platform was performed, similar to our previously reported tracer candidates. As we aimed at reducing the albumin binding of the herein presented compounds, we performed measurements in the presence or absence of a mouse physiological concentration of BSA (2.5 g/dL). Overall, the association and dissociation rate constants did not differ dramatically when the compounds were incubated with medium only (Table 2).

**Table 2:** Kinetic Parameters (Normalized Maximum Number of Binding Sites *B*_max_, Association Rate Constant *k*_a_, Dissociation Rate Constant *k*_d_, and Dissociation Constant *K*_D_) for Radioligands [^64^Cu]Cu-**1**–**6**

compound	*k*_a_ [(M s)^−^^1^]	*k*_d_ [s^–1^]	*K*_D_ [nM]	*B*_max_ [normalized (%)]
Medium only (*n* = 2; Mean ± S.D.)				
[^64^Cu]Cu-**1**	(2.85 ± 0.07) × 10^3^	(17.2 ± 0.35) × 10^–^^5^	60.2 ± 0.35	186 ± 40.2
[^64^Cu]Cu-**2**	(0.43 ± 0.02) × 10^3^	(26.3 ± 0.91) × 10^–^^5^	608 ± 9.19	1167 ± 72.0
[^64^Cu]Cu-**3**	(6.59 ± 0.50) × 10^3^	(13.6 ± 0.25) × 10^–^^5^	20.9 ± 5.44	95.9 ± 6.49
[^64^Cu]Cu-**4**	(0.56 ± 0.35) × 10^3^	(8.80 ± 1.53) × 10^–^^5^	201 ± 149	846 ± 451
[^64^Cu]Cu-**5**	(1.14 ± 0.60) × 10^3^	(10.9 ± 0.49) × 10^–^^5^	110 ± 52.96	446 ± 274
[^64^Cu]Cu-**6**	(5.30 ± 0.52) × 10^3^	(11.7 ± 0.14) × 10^–^^5^	22.2 ± 1.98	107 ± 7.16
Medium + 2.5% BSA (*n* = 1)				
[^64^Cu]Cu-**1**	1.41 × 10^3^	0.17 × 10^–^^3^	119	76.2
[^64^Cu]Cu-**2**	0.36 × 10^3^	0.17 × 10^–^^3^	468	104
[^64^Cu]Cu-**3**	2.97 × 10^3^	0.16 × 10^–^^3^	54.3	46.6
[^64^Cu]Cu-**4**	0.23 × 10^3^	0.17 × 10^–^^3^	744	883
[^64^Cu]Cu-**5**	1.13 × 10^3^	0.11 × 10^–^^3^	100	219
[^64^Cu]Cu-**6**	3.59 × 10^3^	0.14 × 10^–^^3^	41.4	65.3

In the absence of BSA, assumed to reflect actual binding kinetics, [^64^Cu]Cu-**3** (Figure 2C,D) showed the highest affinity (20.9 nM), close to that of [^64^Cu]Cu-**6** (22 nM). [^64^Cu]Cu-**1** and [^64^Cu]Cu-**5** affinities were found to be slightly lower (60/110 nM), while [^64^Cu]Cu-**2** and [^64^Cu]Cu-**4** yielded a low *K*_D_ (608 and 201 nM).

Even though not directly comparable, there was some agreement between saturation (with BSA) and real-time binding (without BSA). Using the statistical range as a measure, the agreement (interpreted as a small range) was good for radioligands [^64^Cu]Cu-**1** and [^64^Cu]Cu-**5**, with a value of 20 and 2, respectively. [^64^Cu]Cu-**3** and [^64^Cu]Cu-**4** showed less agreement (range of 73 and 119). In contrast, agreement of saturation and real-time binding was low for compounds [^64^Cu]Cu-**2** and [^64^Cu]Cu-**6** (range of 409 and 510).

When comparing affinity in real-time binding in the presence and absence of BSA, compounds [^64^Cu]Cu-**1**, **2**, **5**, and **6** showed only a small difference between both conditions (equal to or below a fold change of 2 in either direction). This could indicate a lower BSA off-target binding. However, this conclusion must be treated with caution. Binding curves in the presence of BSA show minimal curvature and pronounced signal increases at the start of an incubation, likely reflecting the very fast binding to albumin. This will affect the accuracy of the data fitting and therefore calculation of the kinetic parameters. Additionally, data were derived from a single experiment. It is therefore difficult to conclude on structural changes affecting albumin binding, thereby interfering with PD-L1 binding. Using PC3 mock cells did not yield any discernible binding (data not shown).

### PET Imaging and Ex Vivo Biodistribution

Similar to our previously reported compounds,^45^ the in vitro affinities did not predict the in vivo behavior, indicating that metabolism or in vivo stability could interfere with target binding. Time points of PET imaging were 0–2, 4–5, and 24–25 h post injection (p.i.). Out of all investigated compounds, only [^64^Cu]Cu-**4** showed an almost exclusive hepatobiliary elimination, as indicated by pronounced early liver (0–2 h) and intestinal (0–2 and 4–5 p.i.) accumulation. All other compounds showed a mixed excretion pattern, with a varying involvement of the renal and hepatobiliary route (Figures 4A–C, S120 and S121). Interestingly, compounds [^64^Cu]Cu-**1** and [^64^Cu]Cu-**6** (Figure 4C) showed a stronger accumulation in the kidneys under blocking conditions at 0–2 h postinjection. The reason is not entirely clear; however, it could be speculated that the additional presence of substantial amounts of nonradioactive compound could saturate first-pass effects in the liver, hence increasing elimination via the kidneys. Compounds [^64^Cu]Cu-**2** and [^64^Cu]Cu-**5** showed some early renal and hepatobiliary excretion, later on intensifying in the liver.

**Figure 3:**
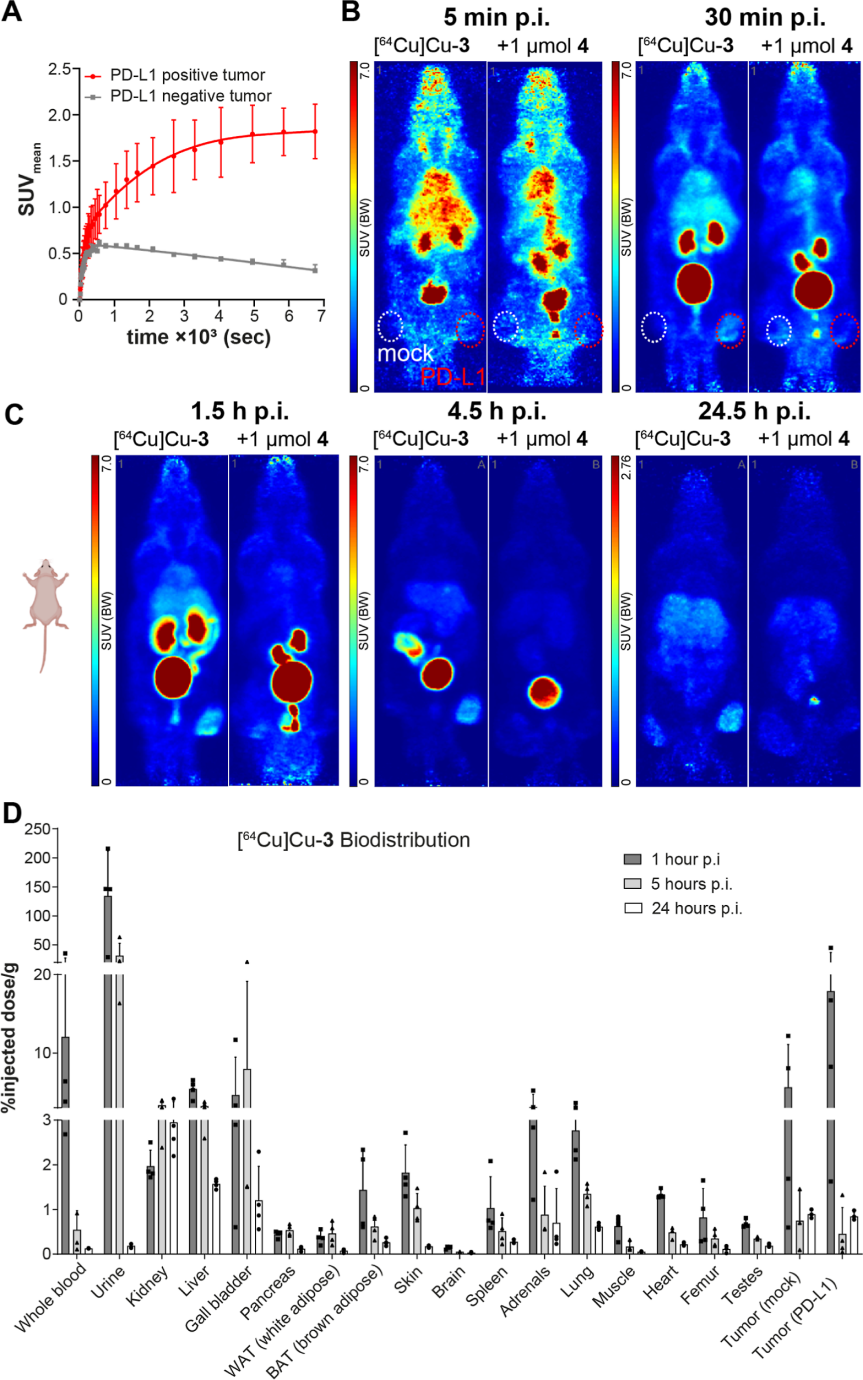
(A) Time–activity curves (SUV_mean_) from two animals with PC3 PD-L1 overexpressing (red circles) and PC3 mock tumors (gray squares) with smoothed spline curves. (A-C) In vivo PET images (maximum intensity projection) shown at selected time points after i.v. injection of [^64^Cu]Cu-**3** and [^64^Cu]Cu-**3** + 1 μmol of **4**. PC3 PD-L1-overexpressing (red) and PC3 mock tumors (white) are highlighted for the two early time points, corresponding to the following time frames: (B) 5 min [4–6 min p.i.] and 30 min [25–40 min p.i.]; (C) 1.5 h [60–120 min p.i.], 4.5 h [4–5 h p.i.], and 24.5 h [24–25 h p.i.]. Scaling differs for 24.5 h postinjection. (D) Ex vivo organ distribution of [^64^Cu]Cu-**3** at 1, 5, and 24 h p.i.

**Figure 4:**
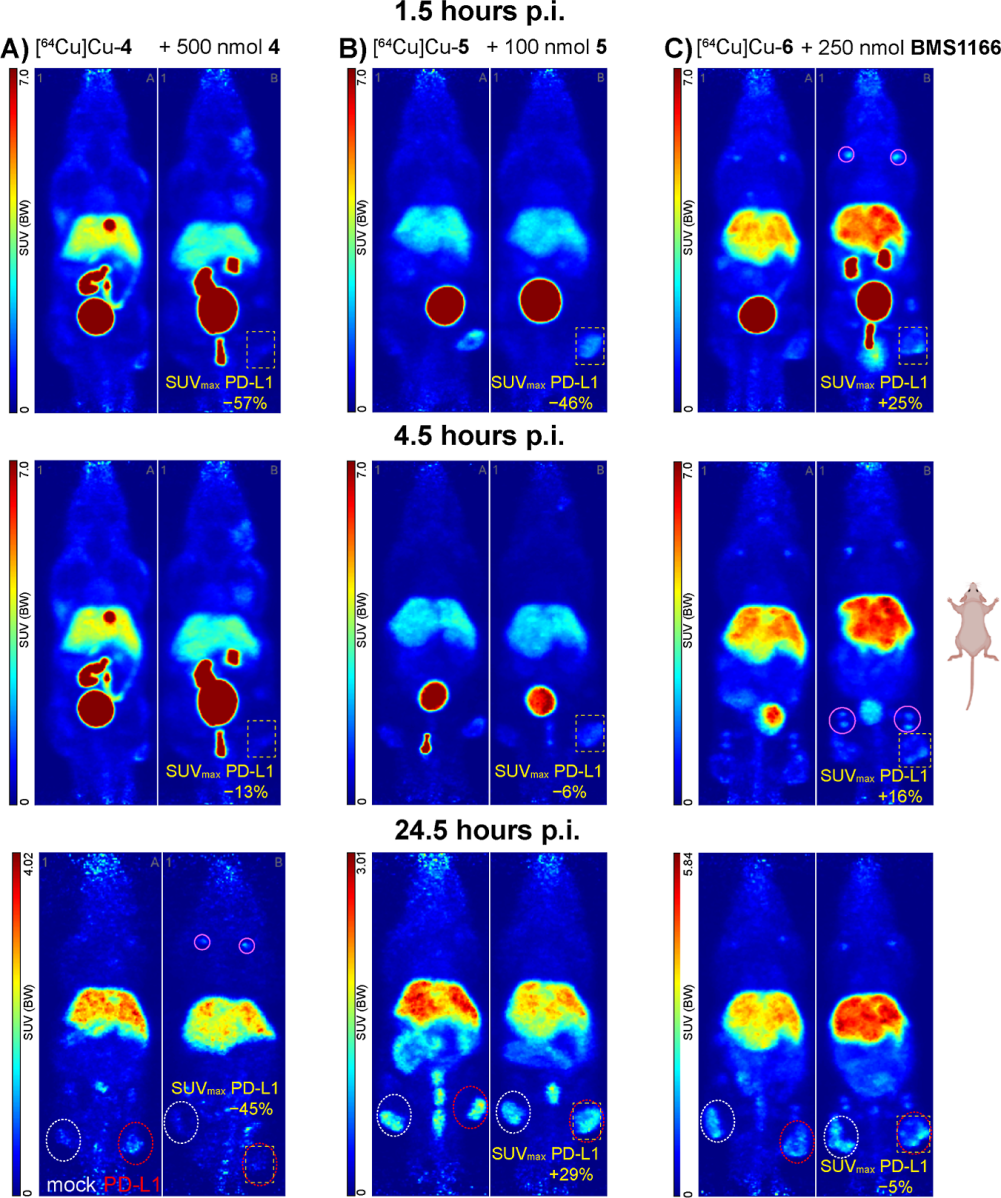
Effect of unlabeled compounds on radioligand binding: (A) compound [^64^Cu]Cu-**4** with 500 nmol of unlabeled **4** (5 min prior, i.v.); (B) compound [^64^Cu]Cu-**5** with 100 nmol of unlabeled **5** (30 min prior, i.v.); and (C) compound [^64^Cu]Cu-**6** with 250 nmol of BMS-1166 (5 min prior, i.v.). Dotted circles indicate PC3 PD-L1-overexpressing (red) and PC3 mock tumors (white). Solid purple circles indicate bone/joint uptake for [^64^Cu]Cu-**4** and [^64^Cu]Cu-**6**. Dashed yellow squares indicate reductions of tracer uptake in the PD-L1-overexpressing tumor of animals subjected to blocking, given for each time point (% reduction in SUV_max_ vs tracer-only). Scaling differs for 24.5 h postinjection.

For [^64^Cu]Cu-**2** and [^64^Cu]Cu-**4**–**6**, a substantial amount was observed in the liver and intestine (feces) at 24–25 h (Figure 4A,B). [^64^Cu]Cu-**3** showed the most promising excretion pattern, with PET indicating predominantly a fast renal and lower hepatobiliary clearance early on (Figure 3B). However, in the biodistribution experiment, both the kidneys and liver showed a comparable tracer uptake (% i.d./g) until 4 h (Figure 3B). Overall, blood retention and/or plasma binding of [^64^Cu]Cu-**3** was markedly reduced around 4 h and the radiolabeled compound quickly cleared, mostly through the kidneys and urine (Figure 3B,C). For compounds [^64^Cu]Cu-**4** and more intensely [^64^Cu]Cu-**6**, some accumulation in the joints can be observed (Figure 4C), potentially caused by nonspecific binding (NSB) to bone marrow or macrophages.^54−57^

Tumor uptake varied, depending on tracer candidate and time points. Changes are reported as % change of the SUV_max_ of the PD-L1 expressing over the respective value of the mock tumor. Similarly, the effect of blocking is reported as % change of the SUV_max_ of the animal injected with cold compound over that of the animal that received tracer only.

At 1.5 h (1–2 h), radioligands [^64^Cu]Cu-**1** and [^64^Cu]Cu-**2** showed a low uptake (SUV_max_ ∼ 1–1.5) in the PC3 PD-L1-overexpressing tumor, with negligible to little contrast between target-overexpressing and mock tumor. Radioligand [^64^Cu]Cu-**4** fared marginally better, mainly showing higher contrast between both tumors (1–2 h: 146%, 4–5 h: 166%, and 24–25 h: 60%).

In the PC3 PD-L1-overexpressing tumor, compound [^64^Cu]Cu-**5** showed a higher SUV_max_ of 3.25 at 1–2 h, 306% higher than that observed in the mock tumor. Tracer accumulation and contrast to mock tumor gradually decreased over time (4–5 h: SUV_max_: 1.75, +59% vs mock; 24–25 h: SUV_max_ 1.2, +14% vs mock).

Similarly, the SUV_max_ of radioligand [^64^Cu]Cu-**6** was 2.28 at 1–2 h, 121% higher than that observed in the PC3 mock tumor. However, at later time points, the contrast between PD-L1-overexpressing tumor and mock tumor was lost, with SUV_max_ indicating no difference between both. However, compared to 1.5 h, SUV_max_ decreased at 4.5 h and increased again at 24.5 h, pointing toward tracer breakdown and/or nonspecific uptake rather than simple washout.

Likely caused by the favorable metabolization pattern, compound [^64^Cu]Cu-**3** showed the highest PD-L1-overexpressing tumor uptake, initially (1–2 h) with a mean SUV_max_ of 3.5, being 311% higher than that in the mock tumor. Time–activity curves (Figure 3A) for tumor uptake (SUV_mean_) support the specificity of binding In the first 5 min, uptake is very similar between both tumors. The mock tumor shows washout from 10 min onward, while radioactivity in the PD-L1-overexpressing tumor continuously increases. At 4–5 h, SUV_max_ in the target-overexpressing tumor was only slightly reduced, but contrast to the mock tumor increased further to 372%. After 24 h, SUV_max_ in the PD-L1-overexpressing tumor had dropped to 1.35 while still maintaining good contrast (93% higher than that in the mock tumor). Biodistribution data for compound [^64^Cu]Cu-**3** mostly confirmed observations from PET experiments (Figure 3D). Kidney passage of the radioligands increased until 4–5 h, followed by a slight reduction. Activity concentration in urine was the highest in the first hour after injection, supporting the considerable renal clearance seen in PET. Still, initial (1 h) liver uptake is slightly higher than that of kidneys, with a reversed pattern at later time points (5 and 24 h). However, compared to our previously reported compounds,^45^ liver uptake is less prominent and reduces over time. PET indicates some tracer uptake in brown adipose tissue for all compounds. [^64^Cu]Cu-**3** biodistribution data support this observation, showing consistently higher % i.d./g in brown vs white adipose tissue (between 33 and 286%). This is in line with previously reported data using some^11,58,59^ but not all^60^ radiolabeled PD-L1 antibodies, further supporting native PD-L1 expression by mouse brown adipocytes.

Importantly, metabolite analysis of [^64^Cu]Cu-**3** (see the Supporting Information for details) revealed the overall stability of the compound in circulation even 4 h postinjection. [^64^Cu]Cu-**3** showed less stable excretion from organs (kidney and liver) after 4 h; however, the only main metabolite was free copper, likely released from the chelator (Figure S123).

Attempts at blocking radiotracer uptake in the tumor by different compounds [unlabeled candidate(s), or reference compound BMS-1166] showed varying effects. Similar to tumor uptake, injection of 100 nmol of unlabeled compound per animal prior to [^64^Cu]Cu-**1** or[^64^Cu]Cu-**2** showed no to little blocking effect. For both compounds, the maximum effect on SUV_max_ in the PD-L1 tumor was between −21 and −36% at 24–25 h postinjection. However, the mock tumor also showed a blocking effect (−41 to −54%), and the overall contrast between both tumors was negligible. Radioligand [^64^Cu]Cu-**4** showed a small blocking effect (after 500/1000 nmol unlabeled **4**) on SUV_max_ in the PD-L1-expressing tumor (0–2 h: −57%, 4–5 h: −12% and 24–25 h: −45%). However, this effect was also observed in the mock tumor to a certain extent, primarily at 24–25 h, again pointing to nonspecific or metabolite accumulation. [^64^Cu]Cu-**6** showed no blocking effect when 250 nmol of the reference compound BMS-1166 was injected 5 min before the radiotracer. Compound [^64^Cu]Cu-**5**, with injection of 100 nmol of unlabeled compound either 30 and 60 min prior to radiotracer, showed a reduction of SUV_max_ by −46% at 1–2 h, reduced to almost tracer levels (−5%) at 4–5 h, and completely lost later on. For the most promising candidate, compound [^64^Cu]Cu-**3**, blocking was performed with both unlabeled compound **4** (1000 nmol, 5 min prior to tracer) and the reference compound BMS-1166 (750 nmol, 60 min prior to tracer, intraperitoneally). BMS-1166 had no effect on SUV_max_ at any time point. In contrast, 1000 nmol of unlabeled compound **4** resulted in a stable reduction of SUV_max_ in PD-L1-expressing tumors (−71% at 1–2 h, −73% at 4–5 h, and −52% at 24–25 h p.i.). In contrast, blocking effect in the mock tumor was negligible at 1–2 and 4–5 h and small at (36%) at 24–25 h postinjection.

For other organs, the blocking effect was not quantified but apparent upon visual inspection. A reduction of tracer accumulation under blocking conditions, especially at 1–2 and 4 h, was observable for [^64^Cu]Cu-**1**, [^64^Cu]Cu-**3** and [^64^Cu]Cu-**4**, particularly in the liver.

In order to assess potential blood flow effects of PD-L1 overexpression vs mock tumors, [^18^F]FDG was employed as the surrogate marker as described before.^61^ Irrespective of whether the first 10 min or the whole 3 h were compared, no significant difference was found between [^18^F]FDG uptake into PD-L1-overexpressing and mock tumors (Figure S122). A detailed description of the results can be found in the Supporting Information.

## Discussion

The development of inhibitors disrupting the immune blockade via the PD-1/PD-L1 axis has resulted in one of the most powerful immunological treatment options in cancer therapy. However, roughly only one-third of cancer patients respond to an ICI therapy, while the majority do not benefit. Clear stratification into responders/nonresponders is beneficial both for the patient and the health care system. While repeated biopsies, though less reliable, are still the clinical standard, radiolabeled PD-L1 ligands as tools for noninvasive molecular imaging are emerging as new diagnostic tools. Low-molecular-weight compounds are considered particularly useful for that application, as they usually exhibit favorable pharmacokinetic profiles.

The design and synthesis of the small-molecule-based PD-L1 radiotracers reported here is based on our initial work.^45^ We have previously identified the dimethyl biphenyl moiety to be superior for binding to PD-L1, as compared to the bromobiphenyl moiety. Additionally, the substituent CN or SO_2_Me at the pyridine ring seemed to have a significant influence on the in vivo performance of the resulting PET ligands. To deepen our understanding, we synthesized and tested derivatives bearing either of these groups. Besides these smaller modifications, we aimed to modulate the pharmacokinetic profile of the radiotracers. Although our previously reported radioligands exhibited log *D*_7.4_ values between −2.73 and −3.50, we observed only hepatobiliary clearance in vivo. To increase the water solubility further, we incorporated up to two additional hydrophilizing units in the linker system. Furthermore, our previous radioligands suffered from strong albumin binding, which we attributed to the highly aromatic core structure and the presence of geminal sulfonic acids, which are known to bind to albumin.^47^ In order to reduce the apparently longer circulation time, we herein modified the solubilizing units on the one hand by spatial separation of both sulfonic acids and on the other hand by partial replacement of the sulfonic acids with phosphonic acids, which provide high water solubility, too.^62^ As a consequence of the unexpected high circulation times and maximum tumor accumulation after several hours as observed previously, we switched the PET nuclide from ^68^Ga to ^64^Cu. Since DOTA-copper complexes are prone to transchelation by liver enzymes in rodents,^63^ we replaced DOTA with NODA-GA as a chelator, because the latter shows sufficiently high kinetic stability with copper.^64,65^

With the aforementioned modifications for tuning the pharmacokinetic profile, the synthesis was based on the two key structures **25** and **26** bearing an aldehyde and alkyne for functionalization. In analogy to our previously reported radioligands, two hydrophilizing units were introduced in the solubilizer unit (blue box, Scheme 1) to avoid negative impact on the binding affinity. The first hydrophilizing unit was introduced via reductive amination, which worked excellent for the sulfonic acids (87–89%). However, for the diethyl-protected phosphonoalanine, a very low yield (7%) was obtained. Mass spectrometry analysis showed (partial) deprotection of the ethyl groups, probably caused by cyanide ions from sodium cyanoborohydride acting as pseudo-halogenides. Therefore, we decided to introduce the sulfonic acid at this position for all six radiotracers. As the second hydrophilizing unit, either taurine or diethyl-protected ciliatine^49^ was coupled. For attaching the linker–chelator system, we followed a convergent synthetic strategy. We prepared the four linker structures with one (**16**) and two sulfonic acids (**17**) and one ethyl-protected phosphonic acid moiety (**18**), all of which were attached by the CuAAC reaction, followed by Fmoc removal and conjugation with *tert*-butyl-protected NODA-GA (**23**). In the case of no hydrophilizing unit in the linker structure, **23** was synthesized with the chelator attached before clicking to the binding motif **15**. The resulting radiotracers were labeled with copper-64 under previously reported conditions.^66^ The obtained log *D*_7.4_ values ranged between −3.07 and −4.15, reflecting the high water solubility of all six radioligands. For the compounds [^64^Cu]Cu-**1**, [^64^Cu]Cu-**3**, and [^64^Cu]Cu-**5**, bearing three hydrophilizing units, the log *D*_7.4_ values were in a similar range from −3.70 to −4.00. The fourth sulfonic acid in compound [^64^Cu]Cu-**2** lowered the log *D*_7.4_ value further down to −4.15. However, the lowest log *D*_7.4_ value of −4.28 was observed for compound [^64^Cu]Cu-**6** bearing two phosphonic acids, which could be explained with an additional negative charge per phosphonate group compared to a sulfonate group. As expected, [^64^Cu]Cu-**4** containing no hydrophilic groups in the linker led to a decrease of water solubility (log *D*_7.4_ value of −3.07).

The observed binding affinities (*K*_D_) were in similar range as for the previously reported PD-L1 radioligands^45^ because no significant changes of the core structure were performed. The substitution of the nitrile with the sulfone moiety did not have a significant impact on the binding affinity, as similar *K*_D_ values for [^64^Cu]Cu-**1** and its analogue [^64^Cu]Cu-**3** were obtained. However, the introduction of the second sulfonic acid in the linker structure ([^64^Cu]Cu-**2**) impaired the binding affinity, which resulted in a *K*_D_ value of approximately 200 nM. An even stronger decrease in binding affinity was observed for radioligand [^64^Cu]Cu-**6** (*K*_D_ = 533 nM), which carried a phosphonic acid in the linker structure. Based on these results, among the six radioligands reported here, [^64^Cu]Cu-**1**, [^64^Cu]Cu-**3**, and [^64^Cu]Cu-**5** seemed most promising for in vivo experiments.

Additional real-time radioligand binding yielded a more diverse result. The two replicates of association/dissociation pattern without BSA showed good consistency, yielding affinities only marginally lower than that reported for our previous compounds.^45^ However, binding in the presence of BSA was more variable. Likely caused by an affinity toward albumin, binding curves with BSA showed little curvature in our cellular real-time in vitro experiments, a necessary requirement for curve fitting.^67^ Additionally, the very fast increase in signal intensity, likely caused by the fast binding to albumin, also makes curve fitting more prone to errors.

Small-animal PET imaging was performed in mice bearing tumors grown from either PC3 PD-L1-overexpressing or PC3 cells transduced with a mock construct. Our analysis of the employed cells and xenograft tissue indicates high and negligible PD-L1 expression by the PC3 PD-L1 and PC3 mock cells, respectively. However, a basal PD-L1 expression by PC3 wild-type cells has been reported before,^53^ which prompted us to avoid considering the PC3 mock cells as completely PD-L1 negative.

PET imaging revealed significantly different pharmacokinetic behavior depending on the amount and type of hydrophilizing units. However, in comparison to our previously reported compounds, we managed to shift the pharmacokinetics from an exclusively hepatobiliary to a more mixed profile. This is exemplified partly by compounds [^64^Cu]Cu-**1** and [^64^Cu]Cu-**2**, showing both a mixed hepatobiliary and renal clearance. As expected, the liver uptake for [^64^Cu]Cu-**2** was slightly lower due to the presence of four sulfonic acids. Despite the lower *K*_D_ value for [^64^Cu]Cu-**1** (approximately 80 nM) compared to [^64^Cu]Cu-**2** (approximately 200 nM), both compounds showed almost no tumor uptake and no contrast to the mock tumor. Compound [^64^Cu]Cu-**3** is an analogue of [^64^Cu]Cu-**1** bearing a methyl sulfonyl instead of the nitrile group at the pyridine ring. Remarkably, this small structural modification seemed to influence the tumor uptake because [^64^Cu]Cu-**3** showed a clear uptake in the PD-L1-overexpressing tumor with good contrast over the mock tumor. [^18^F]FDG uptake was not significantly different at any time after injection between PD-L1-overexpressing and mock tumors. As [^18^F]FDG tumor uptake in the first minutes provides a reliable measure of blood flow,^61^ we conclude that the observed specific tumor uptake of [^64^Cu]Cu-**3** indeed reflects PD-L1 binding. Clearance of [^64^Cu]Cu-**3** was similar to that of [^64^Cu]Cu-**1** due to the same substitution pattern of hydrophilizing units; however, it was slightly more via the renal route. Despite the pronounced albumin binding, indicated by high blood retention at the 2 h time point, a clear contrast between PC3 mock tumor and PD-L1-overexpressing tumor is observed even at 24 h, making [^64^Cu]Cu-**3** the most promising candidate of this series. Furthermore, metabolite data for [^64^Cu]Cu-**3** indicate the stability of the compound in blood even 4 h p.i., further supporting the specificity of tumor uptake. The ligand with one phosphonic acid, [^64^Cu]Cu-**5**, showed a clear reduction of albumin binding, which is reflected by the lower blood retention in the heart and ascending vessel in the first 30 min. However, tumor uptake was found to be decreased, too, when compared to [^64^Cu]Cu-**3**. The least hydrophilic compound [^64^Cu]Cu-**4** showed primarily hepatobiliary clearance, as can be seen from the considerably higher liver uptake compared to the other radioligands. Furthermore, no uptake in the PD-L1-overexpressing tumor was observed. Radioligand [^64^Cu]Cu-**6** with two phosphonic acids showed an unexpected high accumulation in the liver, which is in stark contrast to the high hydrophilicity of the compound. However, as no uptake in the intestines was observed, a hepatobiliary excretion of the radiotracer seems unlikely. We assume a first-pass effect of the phosphonic acids in the liver or binding to macrophages there, which has been reported before.^68^ Additionally, we observed a consistent uptake in the joints for this compound only. This uptake was found to be more pronounced at the early time points. As the synovial membrane contains both synovial fibroblasts and a number of resident synovial tissue macrophages, it would lend additional support to the assumption of a binding to these macrophages.^55−57,69^ Additionally, synovial macrophages and dendritic and T-cells can also express PD-L1.^70,71^ However, to our knowledge, uptake of PD-L1 specific radiolabeled antibodies and peptides to joints has not been reported so far; hence, a NSB is more likely. Besides nonspecific tracer binding to macrophages, decomposition of the radioligand candidates seems unlikely. This assumption is based on incubation with human serum up to 48 h, yielding no significant decomposition. However, metabolization in vivo could still occur, e.g., via hepatic pathways.

In general, the replacement of two sulfonic acids with phosphonic acids resulted in overall lower SUV_max_ values in the studied tissues, e.g., the lower uptake in the heart, compared to all other radioligands, indicating a significantly faster secretion.

The specificity of the radiotracers was studied through blocking/displacement experiments, both in vitro and in vivo. Using the reference compound BMS-1166 at 300-fold excess in the saturation assay blocked ∼80% binding for all compounds. The easy synthetic access to PD-L1 inhibitor BMS-1166^46^ was the main reason for testing this compound in higher excess for blocking experiments.

The ratio between PD-L1-overexpressing tumor and mock-transduced tumor was convincing for compounds [^64^Cu]Cu-**3**−**6**, especially at the early time point and even when assuming a basal PD-L1 expression of the mock tumors. However, attempts at blocking uptake of [^64^Cu]Cu-**4** and [^64^Cu]Cu-**5** with the corresponding unlabeled compound yielded less convincing effects in the PD-L1 expressing tumor. Interestingly, [^64^Cu]Cu-**6** showed an increased renal clearance under blocking conditions. For the most promising compound [^64^Cu]Cu-**3**, injection of BMS-1166 did not result in any notable reduction of tumor uptake. Due to the highly lipophilic character of BMS-1166, complete solution in physiologic buffers was not possible; hence, an intraperitoneal injection 60–30 min prior to tracer application was attempted, but it proved unsuccessful. The different application route and probably different metabolism of BMS-1166 compared to the hydrophilic PD-L1 radiotracers could explain the unsuccessful blocking experiment. In contrast, using 1 μmol of unlabeled compound **4** yielded a consistently robust blocking effect of ∼40% for [^64^Cu]Cu-**3** at all time points, again indicating good specificity. However, the high synthetic demand to synthesize sufficient amounts of the corresponding cold ligands for performing blocking experiments are a limitation for these experiments. Blocking with 100 nmol of the unlabeled compound proved unsuccessful in terms of tumor uptake, as shown for [^64^Cu]Cu-**1** and [^64^Cu]Cu-**2**.

As reported in our previous work, these biphenyl-based PD-L1 radioligands exhibit high albumin binding, which could contribute to the binding pattern through a low fraction of free radioligand and high amount of radioligand bound to albumin.^45^ Presumably, the tumor uptake at 24 h may not be attributed to the free radioligand but to the radioligand-albumin complex, resulting in a loss of specificity between both tumors. This could explain the irregular blocking effects observed, because it is not only necessary to block the free radioligand but also the albumin-radioligand complex. The relatively high concentration of the albumin protein in blood may provide a reason for why a small excess of unlabeled substance (100 nmol) did not cause sufficient blocking of tumor uptake in the PD-L1 tumor, e.g., for [^64^Cu]Cu-**5** (Figure 3).

## Conclusions

To conclude, we synthesized and evaluated in vitro and in vivo six highly hydrophilic, ^64^Cu-labeled PD-L1 radiotracers on the basis of our previously reported radioligands.^45^ Binding affinity studies have been performed with a saturation binding assay and a real-time binding assessment. Depending on the experiment, most of the radioligands revealed binding affinities in the two-digit nanomolar range with the best *K*_D_ of 21 nM for [^64^Cu]Cu-**3**. We aimed to improve the poor pharmacokinetic profile of our previously reported radioligands by replacing DOTA with NODAGA for reducing liver uptake and by the introduction of up to two additional acidic groups for increasing the hydrophilicity. This led to reduced unspecific binding and a robust renal clearance for most radioligands. Lastly, reduction of the blood circulation time was achieved by increasing the spatial distance between the sulfonic acids and their partial replacement with phosphonic acids. These modifications resulted in vastly different pharmacokinetic profiles depending on the type, substitution pattern, and amount of hydrophilizing units. In this set of radioligands, [^64^Cu]Cu-**3** showed the most promising in vivo behavior with specific binding to PD-L1 and a preferentially renal clearance. This radioligand can serve as the basis for further improvements toward PD-L1 imaging agents.

## Experimental Section

### Visualization of PD-L1 Cocrystals

The X-ray cocrystal structure of PD-L1 with BMS-1166 was taken from the Protein Data Bank (PDB code: 6R3K). Molecular graphic manipulation and visualization was performed with PyMOL 2.5.0 and Discovery Studio Visualizer v21.1.

### Radiochemistry

For radiolabeling, a stock solution of 2 M of the corresponding radiotracer in DMSO was produced. For in vitro or in vivo experiments, 8 nmol of the respective radioligand was used. ^64^Cu was produced in-house by the nuclear reaction ^64^Ni(p,n) → ^64^Cu and has already been described in the literature.^72^^64^Cu was obtained from a 0.1–0.01 M HCl solution, which was added to 100–200 μL of a 1 M HEPES solution (pH 4, adjusted with 1 M HCl). The radiotracer was incubated in the HEPES solution with ^64^Cu in 1.5 mL protein LoBind Eppendorf tubes at 50 °C for 10 min with 300 rpm. A small aliquot was placed on iTLC-SG chromatography paper (stationary phase), which was then developed with a 0.1 M citrate solution (pH 4) (adjusted with 1 M NaOH) as the mobile phase. Analysis of the chromatography paper was performed with a radioisotope thin layer analyzer (Rita Star, Elysia-raytest GmbH, Straubenhardt). Labeling efficiency was determined by the ratio of unbound radiometal (*R*_f_ = 0.9) and radiometal complex (*R*_f_ = 0) by integration of the respective areas. Labeling efficiencies with ^64^Cu of >95% were achieved for all radiotracers.

### Log *D*_7.4_ Determination

*n*-Octanol/water distribution coefficients were determined by the shake-flask method. The experiments for each radioligand was performed in triplicate. 30 μL of the reaction mixture was added to a 1.5 mL Eppendorf tube, which contained 570 μL of PBS (pH 7.4) and 600 μL *n*-octanol. After shaking at room temperature at 1500 rpm for 5 min, the mixture was centrifugated. An aliquot for each phase was carefully withdrawn, and the count rates were measured using a γ-counter (ISOMED2160 sodium iodide crystal detector). The mean values were calculated, corrected for the background activity, and the log *D*_7.4_ value was calculated with following formula
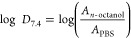


### Kinetic Stability Studies

#### In PBS (pH 7.4)

The corresponding radiotracer was labeled with approximately 500 MBq of ^64^Cu in 50 μL of HEPES solution (adjusted to pH 4.5), as described above. After successful labeling, 450 μL of PBS solution (pH 7.4) was added, and the incubation was performed (room temperature) at 300 rpm. At each time point (1, 24, and 48 h), an aliquot was withdrawn and injected into the radio-HPLC (PerkinElmer, Massachusetts, United States system C or D, Supporting Information). Evaluation and graphical plotting were performed with OriginPro 9.0.

#### In Human Serum

Similar to PBS stability studies, the mixture containing the radiolabeled compound was diluted 1:10 with human serum. The solution was incubated at 37 °C with shaking at 300 rpm. At each time point (1, 24, and 48 h), an aliquot was withdrawn and added to a double amount of “supersol” (water containing 20% v/v EtOH, 5% v/v 5 mM aqueous EDTA, 0.5% v/v Triton X-100, 0.1% m/v saponin, 0.05% v/v 0.5 mM aqueous *o*-phenanthroline) to precipitate serum proteins. The suspension was cooled on ice for 5 min and then centrifugated for 5 min at 12,000*g*. A small amount of the supernatant was analyzed by radio-HPLC (PerkinElmer, Massachusetts, United States system D, Supporting Information). Evaluation and graphical plotting were performed with OriginPro 9.0.

### Cells Lines and Cell Culture

PC3 cells were transduced to overexpress PD-L1 (PC3-PD-L1), along with cells transduced with a control plasmid (PC3-mock). Experimental details of the FACS analysis are reported in the Supporting Information. Following transduction, cells were cultured in RPMI-1640 medium as described before^45^ under normoxic (5% CO_2_; 37 °C) conditions and passaged upon reaching ∼90% confluency.

For saturation binding, cells were trypsinized (0.05% Trypsin–EDTA, Gibco, USA), counted, and diluted to ∼160,000 cells/mL. 0.25 mL of the cell solution was then added to each well of a 48-well plate (Falcon Multiwell #353078) and grown for at least 2 days.

### Saturation Binding Studies

To determine the affinity (*K*_D_) and the number of binding sites (*B*_max_) of the herein described PD-L1 small-molecule ligands, saturation binding was performed in at least three independent experiments, as described before.^45^ Cells were brought to room temperature followed by cooling on ice (each 15 min). The medium was removed and replaced with 200 μL assay buffer [PBS + 2.5% (w/v) BSA] for total binding (TB) conditions or assay buffer containing 300 μM BMS-1166 (in DMSO, resulting in 0.03% v/v) for the assessment NSB. After 15 min preincubation, 200 μL of each of the eight serial 1:1 dilutions (500 to 3.91 nM) of the respective radioligand was added (in triplicate). Cells were then incubated for 90 min at 4 °C, washed (3 × 1 min), and lysed in 500 μL of 0.1 M NaOH + 1% SDS. Radioactivity of the lysate was measured and decay corrected to a reference time (end of radiolabeling). An additional plate was subjected to the same conditions (preincubation, incubation, and washing) using PBS without BSA for each experiment, and the protein content of these lysates was determined by BCA assay. From CPM measurements, final values (pmol/mg protein) were calculated using the mean protein content and molar activity.

Processed data were then subjected to nonlinear iterative curve fitting (GraphPad Prism 9) to yield *B*_max_ (in pmol/mg protein) and *K*_D_ (in nM).

### Real-Time Radioligand Binding Studies

For real-time binding to assess kinetics (association rate constant *k*_a_ and dissociation rate constant *k*_d_) and dissociation constant (*K*_D_), LigandTracer (Yellow, Ridgeview Instruments AB, Sweden) was employed. Approximately 1.2 × 10^6^ cells (in 3 mL medium) were seeded to one side of a Petri dish (Nunc) 1 day prior to experiments as described before.^45^ Binding was then performed at room temperature (using CO_2_-independent medium, Gibco #18045088, Thermo Fisher Germany) with or without BSA (2.5%). First, association was determined by incubation with two concentrations (10 and 40 nM, 90 min each) of the ^64^Cu-labeled PD-L1 ligands. Dissociation was then assessed by replacement with fresh medium for at least 2 h. Data were acquired in decay-corrected counts per second (CPS), and binding data were then evaluated using TraceDrawer (1.9.2, Ridgeview Instruments AB, Sweden). Traces were inspected for spikes and irregularities and then normalized to their own baseline (=0%) and highest value (=100%). Data were then fitted using a one-to-one interaction, accounting for the bulk effect.

### Animals, Biodistribution, and PET Imaging

Animal experiments were performed in accordance with the guidelines of the German Regulations for Animal Welfare, approved by the Saxonian Ethical Committee for Animal Experiments (reference number DD24.1-5131/449/49). Male athymic NMRI-nude mice (Rj: NMRI-Foxn1^nu/nu^, Janvier Laboratories, Le Genest-Saint-Isle, France) between 8 and 22 weeks of age were used. Under general anesthesia, [∼10% desflurane (Baxter, Deerfield, IL, USA) in 30 vol % oxygen + air] and warming, the animals were subcutaneously injected with 3–5 × 10^6^ PC3-PD-L1 and PC3-mock cells [in 50 μL PBS +50 μL Matrigel (Corning, Glendale, USA)] into the right and left thigh, respectively. Tumor growth was monitored three times a week by caliper measurements, and mice with tumor sizes above 7 mm were included in the experiments.

For PET studies, general anesthesia was induced as described above. PET and X-ray computed tomography (CT) were performed in a dedicated small animal nanoScan PET/CT scanner, with four animals imaged simultaneously (Mediso, Budapest, Hungary). CT images were employed for attenuation correction and anatomical referencing. Animals received three individual scans corresponding to 0–2 h (dynamic) and 4.5 and 24 h (static) postinjection.

PD-L1 radiotracer candidates (in 300 μL sterile 0.9% NaCl/HEPES buffer, pH 6–7, between 7 and 12 MBq, molar activities > 12 GBq/μmol) were delivered over 30 s i.v. (lateral tail vein) and PET acquisition started simultaneously. For blocking condition, a defined quantity of compound (the same unlabeled compound, one from the same series or BMS-1166) was diluted in sterile saline and injected i.v. approximately 5 min prior to the radiotracer. Animals not subjected to blocking were injected with vehicle at the same time.

[^18^F]FDG was used a surrogate marker of blood flow, injected in three animals and dynamically followed over 3 h. Experimental details are reported in the Supporting Information.

Three-dimensional list mode data were binned using the 400–600 keV energy window and sorted into 36 time frames (15 × 10 s, 5 × 30 s, 5 × 60 s, 4 × 300 s, 3 × 600 s, 4 × 900 s). Time frames were reconstructed using the Tera-TomoTM 3D algorithm applying a voxel size of 0.4 mm and corrections for decay, scatter, and attenuation. Images were postprocessed and analyzed using Rover (ABX GmbH, Radeberg, Germany) and displayed as maximum intensity projection (MIP) at indicated time points and scaling.

Three-dimensional volumes of interest were delineated applying fixed thresholding at 35% of the measured maximum intensity. Standardized uptake values (SUV = [MBq detected activity/mL tissue]/[MBq injected activity/g body weight], mL/g) were determined in selected volumes of interest, among these PD-L1 and mock tumors.

For radiotracer biodistribution studies, tumor-bearing animals were i.v. injected with approximately 2 MBq (approximately 160 pmol) of ^64^Cu-labeled compound **3**. At three time points (1, 5, and 24 h p.i.), animals were euthanized under general anesthesia, followed by blood (heart puncture) and urine collection. All major organs were dissected and weighted, and activity was measured in a gamma counter (PerkinElmer Wizard 1480), along with radioactive standards.

### Data and Statistical Analysis

Data are presented as mean values ± standard deviation. All statistical procedures were performed using GraphPad Prism, v9.0 (GraphPad Software Inc., USA).

### General Remarks

All manipulations that require the exclusion of oxygen and moisture were carried out under an argon gas atmosphere in heat-gun dried flasks utilizing the Schlenk technique. Solvents and chemicals were purchased from Sigma-Aldrich Laborchemikalien GmbH, Acros Organics, abcr GmbH, and Fisher Scientific and were used without any purification. For NMR studies, deuterated solvents were purchased from Deutero GmbH. The anhydrous solvents DMF, dichloromethane, tetrahydrofuran, and methanol were purchased from Sigma-Aldrich Laborchemikalien GmbH (Sure/Seal bottles). Thin-layer chromatography (TLC) analysis was performed on Merck precoated plates (silica gel 60 F_254_, Art 5715, 0.25 mm), and visualization was accomplished with UV light or KMNO_4_ stain. Attenuated total reflectance Fourier transform infrared (ATR-FTIR) spectra were recorded with a Thermo Scientific Nicolet iS5 device, and the band intensity was described as weak (w), medium (m), strong (s), and broad signal (bs). NMR spectroscopy was performed with an Agilent DD2-400 MHz NMR or an Agilent DD2-600 MHz NMR spectrometer with ProbeOne. Chemical shifts of ^1^H, ^13^C, and ^31^P signals were reported in parts per million (ppm) at 25 °C with TMS as the internal standard. Spectra were calibrated to the respective solvent signal. High-resolution mass spectra (HR-MS) were acquired on a TOF (Q-TOF MS; electrospray ionization), Agilent 1260 Infinity II HPLC (Santa Clara, CA, USA; pump G7111B, autosampler G7129A, column oven G7116N, UV detector G7717C, eluent acetonitrile/water acidified with 0.1% formic acid, bypass mode) coupled to UHD Accurate Mass Q-TOF LC MS G6538A. Analytical reversed HPLC was conducted on an Agilent C18 column (Agilent Zorbax 300SB-C18, 100 mm × 4.6 mm) with acetonitrile/water (0.1% TFA each) as the mobile phase. Preparative and semipreparative reversed HPLC separations were performed on the Knauer Azura on Zorbax SB C-18 5 μm 80 Å, 9.4 × 250 mm as the stationary phase with acetonitrile/water (0.1% TFA each) as the mobile phase.

Purity of the compounds was determined via analytical HPLC and found to be ≥95%.

### HPLC Systems

#### System A

RP-HPLC, analytical (Agilent Zorbax 300 C-18, 5 μm, 4.6 × 150 mm) with 10–95% acetonitrile (0.1% TFA) in water (0.1% TFA) in a linear gradient over 15 min, 1 mL/min, 23 °C, detection at 254 nm.

#### System B

RP-HPLC, analytical (Agilent Zorbax 300 C-18, 5 μm, 4.6 × 200 mm) with 10–95% acetonitrile (0.1% TFA) in water (0.1% TFA) in a linear gradient over 30 min, 1 mL/min, 23 °C, detection at 254 nm.

#### System C

RP-HPLC, analytical (Phenomenex Jupiter 300 C-18, 5 μm, 4.6 × 250 mm) with 10–95% acetonitrile (0.1% TFA) in water (0.1% TFA) in a linear gradient over 40 min, 1 mL/min, 23 °C, γ-detection.

#### System D

RP-HPLC, analytical (Kinetex 5 μm phenyl-hexyl 100 Å) with 5–95% acetonitrile (0.1% TFA) in water (0.1% TFA) in a linear gradient over 20 min, 1 mL/min, 23 °C, γ-detection.

### General Procedures

#### GP-1: Amide Bond Formation

The carboxylic acid, the base, the amine, the coupling reagent, and HOBt (if chirality present; 1.0 equiv) were added to abs. DMF at 0 °C. The reaction was stirred at room temperature, whereas the progress of the reaction was monitored by analytical RP-HPLC (system A). After complete conversion, the solvent was removed and the crude product was purified by semipreparative RP-HPLC. After lyophilization, the amide was obtained.

#### GP-2: Click Reaction

The alkyne (1.00 equiv) and azide components (1.00–5.00 equiv) were dissolved in a 1:1 mixture of H_2_O/*t*-BuOH. CuSO_4_ (0.30 equiv), THPTA (0.10 equiv), and sodium ascorbate (5.00 equiv) were premixed in H_2_O/*t*-BuOH (1:1) and then added to the reaction solution, which was stirred at room temperature until complete conversion was achieved (monitored by RP-HPLC system A). All volatiles were removed in vacuo, the crude product was purified by RP-HPLC, and after lyophilization, the desired triazole was obtained.

#### GP-3: Fmoc Deprotection

The Fmoc-bearing compound (1.0 equiv) was dissolved in abs. DMF, sodium azide (5.0 equiv) was added, and the reaction mixture was heated to 60 °C for 3 h. After confirming complete conversion by analytical HPLC (system A), the solvent was removed under reduced pressure. The residue was purified by semipreparative RP-HPLC, and subsequent lyophilization provided the free amine.

#### GP-4: *tert*-Butyl-Ester Deprotection

The chelator compound bearing *tert*-butyl esters was stirred in the deprotection cocktail (TFA/DCM/TES/H_2_O, 20:20:8:7, v/v) at room temperature for 40 h. The threefold deprotection was confirmed with analytical RP-HPLC (system A). The solvent was removed in vacuo, the residue was purified by semipreparative RP-HPLC, and after lyophilization, the title compound was obtained.

#### GP-5: Dealkylation of Phosphonate Esters and *tert*-Butyl-Ester Deprotection

The alkyl-protected compound was dissolved in an NMR tube under argon in abs. DMF, and then trimethylsilyl bromide (20.0 equiv) was added. The reaction was monitored by ^31^P NMR, and after complete formation of the bis(trimethylsilyl) phosphonate, methanol (50 μL) was added. The NMR tube was shaken and complete hydrolysis of bis(trimethylsilyl) phosphonate was confirmed by ^31^P NMR. The solvent was removed in vacuo, and the residue was directly subjected to the *tert*-butyl-deprotection, which was performed according to GP-4.

### Synthetic Procedures

#### *tert*-Butyl (*R*)-2-((((9*H*-Fluoren-9-yl)methoxy)carbonyl)-amino)-3-(diethoxyphosphoryl)propanoate (**30**)

Triethyl phosphite (3.98 mL, 23.2 mmol, 5.00 equiv) was degassed with argon for 30 min, and then iodide **29** (2.03 g, 4.64 mmol, 1.00 equiv) was added. The solution was stirred at 140 °C for 16 h under argon. Subsequently, all volatiles were removed by vacuum distillation (90 °C, 10^–3^ mbar), and the yellowish residue was used in the next step without further purification.

#### (*R*)-2-((((9*H*-Fluoren-9-yl)methoxy)carbonyl)amino)-3-(diethoxyphosphoryl)propanoic Acid (**21**)

The residue from the previous reaction was dissolved in a 1:1 mixture of TFA/DCM (20 mL). The solution was stirred at room temperature for 5 h, and subsequently, all solvents were removed under reduced pressure. DCM (200 mL) and sodium bicarbonate solution were added (400 mL) to reach pH 8. Phases were separated, the aqueous phase was washed with DCM (150 mL), and then the aqueous phase was adjusted to pH 2 with 1 M hydrochloric acid. The extraction of the aqueous phase was performed with DCM (3 × 200 mL), and the combined organic extracts were dried over sodium sulfate, filtered, and concentrated under reduced pressure. Flash column chromatography on SiO_2_ (EA/MeOH/AcOH, 93:7:1, *R*_f_ = 0.27) was performed to yield the free carboxylic acid **21** (1.04 g, 2.33 mmol, 57% over two steps) as a light yellowish oil. *R*_t_ = 10.90 min (system A), purity = 100%. ^1^H NMR (400 MHz, CD_2_Cl_2_): δ 7.73 (d, ^3^*J* = 7.5 Hz, 2H), 7.54–7.56 (m, 2H), 7.35 (t, ^3^*J* = 7.4 Hz, 2H), 7.24 (t, ^3^*J* = 7.2 Hz, 2H), 4.02–4.46 (m, 7H), 1.20–1.22 ppm (m, 6H). ^13^C NMR (101 MHz, CD_2_Cl_2_): δ 176.7, 156.9, 144.4, 141.8, 128.2, 127.6, 125.8, 120.4, 67.8, 63.6 (d, ^2^*J* = 33.5 Hz), 51.0, 25.5 (d, ^1^*J* = 150.2 Hz), 16.5 ppm (d, ^3^*J* = 5.6 Hz). ^31^P NMR (162 MHz, CD_2_Cl_2_): δ 28.7 ppm. IR (ATR): *ṽ* 1682 (s), 1607 (m), 1519 (w), 1448 (m), 1206 (s), 1137 (s), 1023 (s), 971 (w), 840 (w), 799 (w), 759 (w), 738 (m), 722 cm^–1^ (m). MS (HR-ESI^+^): exact mass calculated for [M + H]^+^: *m*/*z* 448.1480, measured: *m*/*z* 448.1518.

#### (9*H*-Fluoren-9-yl)methyl (*R*)-(1-(4-(3-Azidopropyl)piperazin-1-yl)-3-(diethoxyphosphoryl)-1-oxopropan-2-yl)carbamate (**18**)

Carboxylic acid **21** (50.0 mg, 111.8 μmol, 1.00 equiv), 1-(3-azidopropyl)piperazine (**22**) (28.4 mg, 167.6 μmol, 1.50 equiv), HBTU (50.9 mg, 134.1 μmol, 1.20 equiv), HOBt (15.1 mg, 111.8 mol, 1.00 equiv), and abs. pyridine (18.2 μL, 223.5 μmol, 2.00 equiv) were reacted in abs. DMF (2 mL) according to GP-1. Semipreparative RP-HPLC [Agilent Zorbax SB C-18 5 μm 80 Å, 9.4 × 250 mm with 30–90% acetonitrile (0.1% TFA) in water (0.1% TFA) in a linear gradient over 45 min, 6 mL/min, *R*_t_ = 8 min] with subsequent lyophilization yielded linker **18** (38.0 mg, 63.5 μmol, 57%) as a colorless oil. *R*_t_ = 10.40 min (system A), purity = 96.8%. ^1^H NMR (400 MHz, DMSO-*d*_6_): δ 10.47 (bs, 1H), 7.89 (d, ^3^*J* = 7.5 Hz, 2H), 7.70–7.72 (m, 2H), 7.42 (t, ^3^*J* = 7.4 Hz, 2H), 7.33 (t, ^3^*J* = 7.4 Hz, 2H), 4.65–4.73 (m, 1H), 4.32–4.33 (m, 2H), 4.20–4.23 (m, 1H), 3.94–3.99 (m, 4H), 3.46 (t, ^3^*J* = 6.4 Hz, 2H), 3.11 (bs, 2H), 2.12–2.29 (m, 2H), 1.90 (bs, 2H), 1.20 ppm (q, ^3^*J* = 6.8 Hz, 6H). ^13^C NMR (101 MHz, CD_2_Cl_2_): δ 169.1, 169.0, 158.5, 155.4, 143.7, 140.7, 127.6, 127.0, 125.2, 120.1, 65.8, 61.3 (d, ^3^*J* = 6.2 Hz), 53.3, 50.8, 47.9, 46.6, 45.4, 42.2, 27.5 (d, ^1^*J* = 139.2 Hz), 23.1, 16.2 (d, ^3^*J* = 5.9 Hz). ^31^P NMR (162 MHz, DMSO-*d*_6_): δ 27.1 ppm. IR (ATR): *ṽ* 2101 (s), 1660 (s), 1531 (w), 1448 (m), 1248 (n), 1196 (s), 1130 (n), 1023 (s), 970 (n), 830 (w), 798 (w), 760 (w), 740 (m), 720 cm^–1^ (m). MS (HR-ESI^+^): exact mass calculated for [M + H]^+^: *m*/*z* 599.2747, measured: *m*/*z* 599.2745.

#### (*R*)-2-((((9*H*-Fluoren-9-yl)methoxy)carbonyl)amino)-3-(4-(3-azidopropyl)piperazin-1-yl)-3-oxopropane-1-sulfonic Acid (**16**)

Fmoc-l-cysteic acid (120 mg, 307 μmol, 1.00 equiv), 1-(3-azidopropyl)piperazine (**22**) (77.8 mg, 460 μmol, 1.50 equiv), HBTU (233 mg, 613 μmol, 2.00 equiv), and pyridine (79 μL, 921 μmol, 3.00 equiv) were reacted in abs. DMF (3 mL) according to GP-1. Semipreparative RP-HPLC [Agilent Zorbax SB C-18 5 μm 80 Å, 9.4 × 250 mm with 20–60% acetonitrile (0.1% TFA) in water (0.1% TFA) in a linear gradient over 45 min, 6 mL/min, *R*_t_ = 13 min] with subsequent lyophilization yielded compound **16** (116 mg, 214 μmol, 70%) as a colorless powder. mp 155 °C. *R*_t_ = 9.12 min (system A), purity: 98.1%. ^1^H NMR (400 MHz, DMSO-*d*_6_): δ 7.89 (d, ^3^*J* = 7.5 Hz, 2H), 7.71 (d, ^3^*J* = 7.4 Hz, 2H), 7.41 (t, ^3^*J* = 7.4 Hz, 2H), 7.31–7.34 (m, 2H), 4.77–4.82 (m, 1H), 4.46–4.50 (m, 1H), 4.18–4.27 (m, 3H), 3.45–3.47 (m, 4H), 3.10–3.17 (m, 2H), 2.92–3.03 (m, 2H), 2.72 (m, 1H), 1.88–1.92 ppm (m, 2H). ^13^C NMR (101 MHz, DMSO-*d*_6_): δ 170.3, 155.6, 143.8, 143.7, 140.7, 127.6, 127.1, 127.0, 125.4, 125.3, 120.1, 65.8, 53.6, 53.4, 50.5, 4.78, 47.1, 46.6, 43.6, 42.5, 42.1, 23.8, 22.9 ppm. MS (HR-ESI^+^): exact mass calculated for [M + H]^+^: *m*/*z* 543.2021, measured: *m*/*z* 543.2025.

#### ((((9*H*-Fluoren-9-yl)methoxy)carbonyl)(sulfo)-d-alanyl)(sulfo)-d-alanine (**20**)

Fmoc-l-cysteic acid (100 mg, 256 μmol, 1.00 equiv), *N*-hydroxy succinimide (30.9 mg, 268 μmol, 1.05 equiv), and DCC (55.4 mg, 268 μmol, 1.05 equiv) were dissolved in abs. DMF (2 mL) at 0 °C and were stirred at room temperature for 16 h. The reaction completion was observed by analytical RP-HPLC (system A), and then the reaction solution was added dropwise to a solution of l-cysteic acid (43.2 mg, 256 μmol, 1.00 equiv) in a 6.8% sodium carbonate solution (1 mL) at 0 °C. The reaction mixture was stirred at this temperature for 3 h until complete conversion was verified by analytical RP-HPLC (system A). The mixture was acidified with 10% hydrochloric acid to pH 2, concentrated in vacuo, and purified by semipreparative RP-HPLC [Agilent Zorbax SB C-18 5 μm 80 Å, 9.4 × 250 mm with 10–70% acetonitrile (0.1% TFA) in water (0.1% TFA) in a linear gradient over 45 min, 6 mL/min, *R*_t_ = 8 min]. After lyophilization, the disulfonic acid **20** (74.2 mg, 136 μmol, 53% over two steps) was obtained as a colorless oil. *R*_t_ = 6.82 min (system A), purity = 100%. ^1^H NMR (400 MHz, DMSO-*d*_6_): δ 7.89 (d, ^3^*J* = 7.5 Hz, 2H), 7.71 (d, ^3^*J* = 7.4 Hz, 2H), 7.41 (t, ^3^*J* = 7.4 Hz, 2H), 7.31–7.34 (m, 2H), 4.77–4.82 (m, 1H), 4.46–4.50 (m, 1H), 4.18–4.27 (m, 3H), 3.45–3.47 (m, 4H), 3.10–3.17 (m, 2H), 2.92–3.03 (m, 2H), 2.72 (m, 1H), 1.88–1.92 ppm (m, 2H). ^13^C NMR (101 MHz, DMSO-*d*_6_): δ 8.33–8.34 (m, 1H), 7.88 (t, ^3^*J* = 7.5 Hz, 2H), 7.72–7.73 (m, 2H), 7.41 (t, ^3^*J* = 7.4 Hz, 2H), 7.31–7.39 (m, 2H), 4.36–4.38 (m, 1H), 4.22–4.23 (m, 4H), 2.81–2.99 ppm (m, 4H). ^13^C NMR (101 MHz, DMSO-*d*_6_): δ 171.6, 170.5, 155.8, 143.9, 143.9, 140.7, 127.7, 127.3, 125.5, 120.1, 66.0, 52.5, 51.8, 50.9, 49.9, 46.6 ppm. MS (HR-ESI^+^): exact mass calculated for [M + H]^+^: *m*/*z* 543.0743, measured: *m*/*z* 543.0731.

#### (*R*)-2-((((9*H*-Fluoren-9-yl)methoxy)carbonyl)amino)-3-(((*R*)-1-(4-(3-azidopropyl)piperazin-1-yl)-1-oxo-3-sulfopropan-2-yl)amino)-3-oxopropane-1-sulfonic Acid (**17**)

Disulfonic acid **20** (56.0 mg, 103 μmol, 1.00 equiv), 1-(3-azidopropyl)piperazine (**22**) (35.0 mg, 206 μmol, 2.00 equiv), HBTU (78.2 mg, 103.2 μmol, 2.00 equiv), HOBt (13.9 mg, 103 μmol, 1.00 equiv), and pyridine (18.6 μL, 310 μmol, 3.00 equiv) were reacted in abs. DMF (3 mL) according to GP-1. Semipreparative RP-HPLC [Agilent Zorbax SB C-18 5 μm 80 Å, 9.4 × 250 mm with 15–60% acetonitrile (0.1% TFA) in water (0.1% TFA) in a linear gradient over 45 min, 6 mL/min, *R*_t_ = 10 min] with subsequent lyophilization yielded linker structure **17** (37.2 mg, 53.6 μmol, 52%) as a yellowish oil. *R*_t_ = 7.83 min (system A), purity = 95.3%. ^1^H NMR (400 MHz, DMSO-*d*_6_): δ 9.48 (bs, 1H), 8.25–8.33 (m, 1H), 7.89 (d, ^3^*J* = 7.5 Hz, 2H), 7.69–7.70 (m, 2H), 7.41 (t, ^3^*J* = 7.4 Hz, 2H), 7.32–7.35 (m, 2H), 4.23–4.43 (m, 6H), 3.35–3.53 (m, 6H), 2.65–3.08 (m, 8H), 1.89 ppm (bs, 2H). ^13^C NMR (101 MHz, DMSO-*d*_6_): δ 169.8, 155.6, 143.9, 143.8, 140.7, 127.6, 127.2, 125.3, 120.1, 65.9, 53.6, 52.9, 52.1, 51.7, 50.4, 47.8, 46.6, 45.3, 42.6, 22.9 ppm. MS (HR-ESI^+^): exact mass calculated for [M + H]^+^: *m*/*z* 694.1965, measured: *m*/*z* 694.1957.

#### Di-*tert*-butyl 2,2′-(7-(5-(4-(3-Azidopropyl)piperazin-1-yl)-1-(*tert*-butoxy)-1,5-dioxopentan-2-yl)-1,4,7-triazonane-1,4-diyl)(*R*)-diacetate (**23**)

Carboxylic acid **24** (15.0 mg, 27.6 μmol, 1.00 equiv), 1-(3-azidopropyl)piperazine (**22**) (7.0 mg, 41.4 μmol, 1.50 equiv), HBTU (12.6 mg, 33.1 μmol, 1.20 equiv), and abs. DIPEA (7.3 μL, 55.2 μmol, 2.00 equiv) were reacted in abs. DMF (1 mL) according to GP-1. Semipreparative RP-HPLC [Agilent Zorbax SB C-18 5 μm 80 Å, 9.4 × 250 mm with 30–80% acetonitrile (0.1% TFA) in water (0.1% TFA) in a linear gradient over 45 min, 6 mL/min, *R*_t_ = 8 min, detection at 220 nm] with subsequent lyophilization yielded linker **23** (15.0 mg, 21.6 μmol, 78%) as a colorless oil. *R*_t_ = 10.20 min (system A), purity = 97.0%. ^1^H NMR (400 MHz, CD_3_OD): δ 3.97–3.98 (m, 3H), 2.59–3.66 (m), 1.91–2.13 (m, 5H), 1.45–1.50 ppm (m, 27H). ^13^C NMR (101 MHz, CD_3_OD): δ 173.0, 171.2, 168.7, 162.7, 162.4, 119.4, 116.5, 84.5, 83.4, 64.7, 56.3, 56.3, 55.7, 52.9, 52.7, 51.6, 51.1, 47.0, 46.3, 43.5, 39.7, 31.0, 28.5, 28.5, 28.4, 26.7, 24.7 ppm. IR (ATR): *ṽ* 2102 (m), 1727 (m), 1683 (s), 1456 (w), 1139 (m), 1251 (w), 1196 (m), 1149 (s), 1127 (s), 976 (w), 830 (w), 798 (w), 735 (m), 719 cm^–1^ (m). MS (HR-ESI^+^): exact mass calculated for [M + H]^+^: *m*/*z* 695.4820, measured: *m*/*z* 695.4824.

#### (5-Chloro-2-((5-cyanopyridin-3-yl)methoxy)-4-((2,2′-dimethyl-3′-(prop-2-yn-1-yloxy)-[1,1′-biphenyl]-3-yl)methoxy)benzyl)(sulfo)-l-alanine (**37**)

Aldehyde **25** (70 mg, 130 μmol, 1.00 equiv) and l-cysteic acid (231 mg, 652 μmol, 5.00 equiv) were stirred in a 1:1 mixture of DMF/MeOH (2 mL) under an argon atmosphere. After stirring for 20 min at room temperature, the reaction solution was cooled to 0 °C and sodium cyanoborohydride (12.3 mg, 196 μmol, 1.50 equiv) was added. After reaction completion, monitored by analytical RP-HPLC (system A), the solvent was removed under reduced pressure. The crude reaction mixture was purified by semipreparative RP-HPLC [Agilent Zorbax SB C-18 5 μm 80 Å, 9.4 × 250 mm with 37–90% acetonitrile (0.1% TFA) in water (0.1% TFA) in a linear gradient over 45 min, 6 mL/min, *R*_t_ = 12 min]. Subsequent lyophilization gave carboxylic acid **37** (80.0 mg, 116 μmol, 89%) as a colorless powder. mp 180 °C. *R*_t_ = 12.53 min (system A), purity: 99.1%. ^1^H NMR (400 MHz, DMSO-*d*_6_): δ 9.34 (bs, 1H), 9.12 (bs, 1H), 9.02–9.03 (m, 2H), 8.49 (s, 1H), 7.55 (s, 1H), 7.46 (d, ^3^*J* = 7.7 Hz, 1H), 7.22–7.28 (m, 2H), 7.18 (s, 1H), 7.04–7.09 (m, 2H), 6.74 (d, ^3^*J* = 7.6 Hz, 1H), 5.39–5.40 (m, 2H), 5.30–5.32 (m, 2H), 4.85 (d, ^4^*J* = 2.3 Hz, 2H), 4.32–4.35 (m, 1H), 4.20–4.24 (m, 2H), 3.59 (t, ^4^*J* = 2.0 Hz, 1H), 3.05–3.07 (m, 1H), 2.88–2.94 (m, 1H), 2.03 (s, 3H), 1.83 ppm (s, 3H). ^13^C NMR (101 MHz, DMSO-*d*_6_): δ 168.4, 156.3, 155.4, 155.4, 152.4, 151.9, 142.3, 141.5, 139.0, 134.5, 134.4, 132.5, 132.4, 129.3, 127.7, 126.2, 125.4, 124.1, 122.0, 116.9, 113.1, 113.0, 110.9, 109.1, 100.2, 79.5, 78.1, 69.7, 67.0, 56.2, 55.8, 48.6, 44.6, 15.3, 12.8 ppm. MS (HR-ESI^+^): exact mass calculated for [M + H]^+^: *m*/*z* 690.1672, measured: *m*/*z* 699.1674.

#### (5-Chloro-4-((2,2′-dimethyl-3′-(prop-2-yn-1-yloxy)-[1,1′-biphenyl]-3-yl)methoxy)-2-((5-(methylsulfonyl)pyridin-3-yl)methoxy)benzyl)(sulfo)-d-alanine (**38**)

Aldehyde **26** (100 mg, 169 μmol, 1.00 equiv) and l-cysteic acid (180 mg, 508 μmol, 3.00 equiv) were stirred in a 1:1 mixture of DMF/MeOH (2 mL) under an argon atmosphere. After stirring for 20 min at room temperature, the reaction solution was cooled to 0 °C and sodium cyanoborohydride (16.0 mg, 254 μmol, 1.50 equiv) was added. After reaction completion, monitored by analytical RP-HPLC (system A), the solvent was removed under reduced pressure. The crude reaction mixture was purified by semipreparative RP-HPLC [Agilent Zorbax SB C-18 5 μm 80 Å, 9.4 × 250 mm with 45–90% acetonitrile (0.1% TFA) in water (0.1% TFA) in a linear gradient over 45 min, 6 mL/min, *R*_t_ = 6 min]. Subsequent lyophilization gave carboxylic acid **38** (108 mg, 145 μmol, 86%) as a colorless powder. mp 162–165 °C. *R*_t_ = 11.9 min (system A), purity = 95.0%. ^1^H NMR (400 MHz, DMSO-*d*_6_): δ 9.93 (bs, 1H), 9.07–9.10 (m, 2H), 8.51 (s, 1H), 7.54 (s, 1H), 7.49 (d, ^3^*J* = 7.5 Hz, 1H), 7.22–7.29 (m, 3H), 7.04–7.10 (m, 2H), 6.74 (d, ^3^*J* = 7.5 Hz, 1H), 5.43–5.50 (m, 2H), 5.28–5.36 (m, 2H), 4.86 (d, ^4^*J* = 1.9 Hz, 2H), 4.18–4.32 (m, 4H), 3.58 (s, 1H), 3.03–3.07 (m, 1H), 2.87–2.93 (m, 1H), 2.03 (s, 3H), 1.83 ppm (s, 3H). ^13^C NMR (101 MHz, DMSO-*d*_6_): δ 168.4, 156.4, 155.5, 155.4, 153.5, 147.2, 142.3, 141.5, 136.9, 134.8, 134.6, 134.4, 132.7, 132.4, 129.3, 127.8, 126.2, 125.5, 124.1, 122.1, 113.1, 113.1, 110.9, 100.3, 79.5, 78.2, 69.7, 67.4, 56.2, 55.8, 48.6, 44.6, 43.6 ppm. IR (ATR): *ṽ* 1577 (w), 1505 (w), 1454 (w), 1409 (w), 1306 (m), 1232 (m), 1144 (s), 1089 (w), 1019 (m), 956 (w), 784 (w), 768 (w), 721 cm^–1^ (w). MS (HR-ESI^+^): exact mass calculated for [M + H]^+^: *m*/*z* 743.1500, measured: *m*/*z* 743.1491.

#### (*R*)-2-((5-Chloro-2-((5-cyanopyridin-3-yl)methoxy)-4-((2,2′-dimethyl-3′-(prop-2-yn-1-yloxy)-[1,1′-biphenyl]-3-yl)methoxy)benzyl)amino)-3-oxo-3-((2-sulfoethyl)amino)-propane-1-sulfonic Acid (**13**)

Carboxylic acid **37** (80.0 mg, 116 μmol, 1.00 equiv), taurine (21.8 mg, 174 μmol, 1.50 equiv), HBTU (52.8 mg, 139 mmol, 1.20 equiv), HOBt (15.7 mg, 116 μmol, 1.00 equiv), and DIPEA (5.0 μL, 232 mol, 2.00 equiv) were reacted in abs. DMF (3 mL) according to GP-1. Semipreparative RP-HPLC [Agilent Zorbax SB C-18 5 μm 80 Å, 9.4 × 250 mm with 15–60% acetonitrile (0.1% TFA) in water (0.1% TFA) in a linear gradient over 45 min, 6 mL/min, *R*_t_ = 10 min] with subsequent lyophilization yielded alkyne **13** (37.2 mg, 53.6 μmol, 52%) as a colorless powder. mp 180–190 °C (decomposition). *R*_t_ = 11.07 min (system A), purity: 92.0%. ^1^H NMR (400 MHz, DMSO-*d*_6_): δ 9.36 (bs, 1H), 9.02 (bs, 2H), 8.80 (bs, 1H), 8.51 (s, 1H), 7.53 (s, 1H), 7.46 (bs, 1H), 7.22–7.28 (m, 2H), 7.16 (s, 1H), 7.04–7.09 (m, 2H), 6.74 (d, ^3^*J* = 7.5 Hz, 1H), 5.25–5.45 (m, 4H), 4.85 (s, 1H), 4.74 (bs, 1H), 4.21–4.25 (m, 2H), 3.95–4.05 (m, 1H), 3.58–3.59 (m, 2H), 3.37–3.39 (m, 1H), 2.98–3.01, (m, 1H), 2.71–2.77 (m, 1H), 2.64 (t, ^3^*J* = 6.8 Hz, 1H), 2.03 (s, 3H), 1.83 ppm (s, 3H). ^13^C NMR (101 MHz, DMSO-*d*_6_): δ 165.3, 156.2, 155.4, 155.4, 152.3, 151.8, 142.3, 141.5, 139.1, 134.5, 134.5, 132.7, 129.3, 127.7, 126.2, 125.5, 124.1, 122.1, 116.9, 113.0, 112.7, 110.9, 109.1, 100.0, 79.5, 78.2, 69.7, 66.9, 56.4, 55.8, 49.8, 49.7, 44.4, 36.3, 15.3, 12.8 ppm. MS (HR-ESI^+^): exact mass calculated for [M + H]^+^: *m*/*z* 797.1713, measured: *m*/*z* 797.1712.

#### (*R*)-2-((5-Chloro-4-((2,2′-dimethyl-3′-(prop-2-yn-1-yloxy)-[1,1′-biphenyl]-3-yl)methoxy)-2-((5-(methylsulfonyl)pyridin-3-yl)methoxy)benzyl)amino)-3-oxo-3-((2-sulfoethyl)amino)propane-1-sulfonic Acid (**14**)

Carboxylic acid **38** (40.0 mg, 56.2 μmol, 1.00 equiv), taurine (14.1 mg, 113 μmol, 2.00 equiv), HBTU (32.0 mg, 84.4 mmol, 1.50 equiv), HOBt (7.6 mg, 56.2 mol, 1.00 equiv), and abs. DIPEA (19.5 μL, 113 mmol, 2.00 equiv) were reacted in abs. DMF (3 mL) according to GP-1. Semipreparative RP-HPLC [Agilent Zorbax SB C-18 5 μm 80 Å, 9.4 × 250 mm with 35–90% acetonitrile (0.1% TFA) in water (0.1% TFA) in a linear gradient over 45 min, 6 mL/min, *R*_t_ = 11 min] with subsequent lyophilization yielded alkyne **14** (41.2 mg, 64.7 μmol, 86%) as a colorless powder. mp 215 °C. *R*_t_ = 10.70 min (system A), purity = 95.1%. ^1^H NMR (400 MHz, DMSO-*d*_6_): δ 9.35 (bs, 1H), 9.13 (s, 1H), 9.08 (s, 1H), 8.98 (bs, 1H), 8.76 (t, ^3^*J* = 5.4 Hz, 1H), 8.54 (s, 1H), 7.53 (s, 1H), 7.49 (d, ^3^*J* = 7.6 Hz, 1H), 7.22–7.29 (m, 3H), 7.04–7.10 (m, 2H), 6.74 (d, ^3^*J* = 7.5 Hz, 1H), 5.42–5.52 (m, 2H), 5.26–5.35 (m, 2H), 4.85 (d, ^4^*J* = 2.1 Hz, 2H), 4.18–4.21 (m, 1H), 4.00–4.02 (m, 2H), 3.58 (t, ^4^*J* = 2.2 Hz, 1H), 3.34–3.36 (m, 1H), 2.98–3.01 (m, 1H), 2.71–2.78 (m, 1), 2.64 (t, ^3^*J* = 7.1 Hz, 2H), 2.03 (s, 3H), 1.83 ppm (s, 3H). ^13^C NMR (101 MHz, DMSO-*d*_6_): δ 165.3, 156.4, 155.5, 155.4, 153.2, 146.9, 142.3, 141.5, 137.0, 135.1, 134.6, 134.6, 134.5, 133.0, 132.7, 129.3, 127.8, 126.2, 125.5, 124.1, 122.1, 113.1, 112.7, 110.9, 100.1, 79.5, 78.2, 69.7, 67.3, 56.5, 55.9, 49.8, 49.7, 44.3, 43.6, 36.2, 15.3, 12.8 ppm. IR (ATR): *ṽ* 168 (w), 1607 (w), 1575 (w), 1506 (w), 1446 (w), 1409 (w), 1308 (m), 1154 (s), 1089 (w), 1036 (m), 769 (w), 722 (w), 673 cm^–1^ (w). MS (HR-ESI^+^): exact mass calculated for [M + H]^+^: *m*/*z* 850.1541, measured: *m*/*z* 850.1533.

#### (*R*)-2-((5-Chloro-4-((2,2′-dimethyl-3′-(prop-2-yn-1-yloxy)-[1,1′-biphenyl]-3-yl)methoxy)-2-((5-(methylsulfonyl)pyridin-3-yl)methoxy)benzyl)amino)-3-((2-(diethoxyphosphoryl)ethyl)amino)-3-oxopropane-1-sulfonic Acid (**15**)

Carboxylic acid **38** (20.0 mg, 28.1 μmol, 1.00 equiv), diethyl (2-aminoethyl)phosphonate (7.6 mg, 42.2 μmol, 1.50 equiv), HBTU (11.2 mg, 29.5 mmol, 1.05 equiv), HOBt (3.8 mg, 28.1 mol, 1.00 equiv), and abs. DIPEA (9.8 μL, 56.2 mmol, 2.00 equiv) were reacted in abs. DMF (1 mL) according to GP-1. Semipreparative RP-HPLC [Agilent Zorbax SB C-18 5 μm 80 Å, 9.4 × 250 mm with 40–90% acetonitrile (0.1% TFA) in water (0.1% TFA) in a linear gradient over 45 min, 6 mL/min, *R*_t_ = 8 min] with subsequent lyophilization yielded alkyne **15** (15.4 mg, 17.0 μmol, 60%) as a colorless powder. mp 117–120 °C. *R*_t_ = 12.10 min (system A), purity = 100%. ^1^H NMR (400 MHz, DMSO-*d*_6_): δ 9.39 (bs, 1H), 9.11 (s, 1H), 9.07 (s, 1H), 8.98 (bs, 1H), 8.89 (t, ^3^*J* = 5.4 Hz, 1H), 8.51 (s, 1H), 7.55 (s, 1H), 7.48–7.50 (m, 1H), 7.22–7.29 (m, 3H), 7.05–7.10 (m, 2H), 6.74 (d, ^3^*J* = 7.5 Hz, 1H), 5.41–5.48 (m, 2H), 5.26–5.32 (m, 2H), 4.86 (d, ^4^*J* = 1.9 Hz, 2H), 4.20–4.23 (m, 1H), 3.97–4.05 (m, 5H), 3.59 (s, 1H), 3.25–3.29 (m, 2H), 2.98–3.01 (m, 1H), 2.74–2.80 (m, 1H), 2.03 (s, 2H), 1.93–2.01 (m, 2H), 1.24 ppm (t, ^3^*J* = 7.0 Hz, 6H). ^13^C NMR (101 MHz, DMSO-*d*_6_): δ 165.6, 158.4, 158.0, 156.4, 155.5, 155.4, 153.5, 147.1, 142.3, 141.5, 136.9, 134.7, 134.6, 134.6, 134.4, 132.7, 129.3, 127.8, 126.2, 125.2, 124.1, 122.0, 113.1, 112.6, 110.9, 100.1, 79.5, 78.2, 69.7, 67.3, 61.2, 61.1, 56.3, 55.8, 49.7, 44.2, 43.6, 33.5, 25.4, 24.1, 16.3, 16.2, 15.3, 12.8 ppm. ^31^P NMR (162 MHz, DMSO-*d*_6_): δ 28.2 ppm. IR (ATR): *ṽ* 1687 (w), 1606 (w), 1575 (w), 1505 (w), 1446 (w), 1409 (w), 1306 (m), 1163 (s), 1145 (s), 1091 (w), 1021 (m), 964 (m), 785 (w), 766 (w), 589 cm^–1^ (w). MS (HR-ESI^+^): exact mass calculated for [M + H]^+^: *m*/*z* 906.2262, measured: *m*/*z* 906.2256.

#### (*R*)-2-((4-((3′-((1-(3-(4-((((9*H*-Fluoren-9-yl)methoxy)carbonyl)(sulfo)-d-alanyl)piperazin-1-yl)propyl)-1*H*-1,2,3-triazol-4-yl)methoxy)-2,2′-dimethyl-[1,1′-biphenyl]-3-yl)methoxy)-5-chloro-2-((5-cyanopyridin-3-yl)methoxy)benzyl)amino)-3-oxo-3-((2-sulfoethyl)amino)propane-1-sulfonic Acid (**39**)

Alkyne **13** (80.0 mg, 100 μmol, 1.00 equiv) reacted with the linker structure **16** (65.3 mg, 120 μmol, 1.20 equiv) with a premixed catalyst consisting of CuSO_4_ (4.80 mg, 30.1 μmol, 0.30 equiv), THPTA (6.54 mg, 15.1 μmol, 0.15 equiv), and sodium ascorbate (99.3 mg, 502 μmol, 5.00 equiv) in a 1:1 mixture of H_2_O/*t*-BuOH (3 mL) at room temperature for 16 h according to GP-2. The solvent was removed in vacuo, and the residue was used in the next step without further purification.

#### (*R*)-2-Amino-3-(4-(3-(4-(((3′-((2-chloro-5-((5-cyanopyridin-3-yl)methoxy)-4-((((*R*)-1-oxo-3-sulfo-1-((2-sulfoethyl)amino)propan-2-yl)amino)methyl)phenoxy)methyl)-2,2′-dimethyl-[1,1′-biphenyl]-3-yl)oxy)methyl)-1*H*-1,2,3-triazol-1-yl)propyl)piperazin-1-yl)-3-oxopropane-1-sulfonic Acid (**44**)

The residue from the previous reaction was dissolved in abs. DMF (3 mL), and the Fmoc group was removed with sodium azide (13.0 mg, 200 μmol, 2.00 equiv) according to GP-3. Semipreparative RP-HPLC purification [Agilent Zorbax SB C-18 5 μm 80 Å, 9.4 × 250 mm with 30–70% acetonitrile (0.1% TFA) in water (0.1% TFA) in a linear gradient over 45 min, 6 mL/min, *R*_t_ = 7 min] and subsequent lyophilization yielded primary amine **44** (44.0 mg, 39.4 μmol, 39% over two steps) as a colorless solid. mp 240–250 °C. *R*_t_ = 8.97 min (system A), purity: 98.5%. ^1^H NMR (400 MHz, DMSO-*d*_6_): δ 9.73 (bs, 1H), 9.36 (bs, 1H), 9.00–9.02 (m, 2H), 8.78–8.81 (m, 1H), 8.50 (s, 1H), 8.30 (s, 1H), 8.10–8.10 (m, 2H), 7.52 (d, ^3^*J* = 7.5 Hz, 1H), 7.45 (t, ^3^*J* = 7.6 Hz, 1H), 7.16–7.26 (m, 3H), 7.07 (d, ^3^*J* = 7.6 Hz, 1H), 6.73 (d, ^3^*J* = 7.5 Hz, 1H), 5.34–5.45 (m, 2H), 5.17–5.31 (m, 3H), 4.51 (t, ^3^*J* = 6.7 Hz, 2H), 4.26 (bs), 4.00 (bs), 3.55 (bs), 3.38–3.39 (m), 3.19 (bs), 2.96–3.03 (m), 2.73–2.80 (m), 2.65–2.67 (m, 1H), 2.29 (bs, 2H), 2.02–2.03 (m, 3H), 1.79 ppm (m, 3H). ^13^C NMR (101 MHz, DMSO-*d*_6_): δ 166.4, 165.4, 158.6, 158.2, 156.3, 156.2, 156.2, 155.4, 155.4, 152.4, 151.9, 143.3, 142.3, 141.6, 139.0, 134.8, 134.7, 134.5, 132.7, 129.3, 128.0, 126.4, 125.5, 124.5, 124.0, 121.8, 117.0, 113.1, 113.1, 112.7, 110.8, 109.1, 100.1, 69.8, 66.9, 61.8, 56.4, 53.2, 50.8, 49.8, 49.7, 47.6, 46.7, 44.4, 42.0, 36.2, 24.3, 15.4, 12.8 ppm. MS (HR-ESI^+^): exact mass calculated for [M + H]^+^: *m*/*z* 1117.2979, measured: *m*/*z* 1117.2975.

#### (*R*)-2-((*R*)-4-(4,7-Bis(2-(*tert*-butoxy)-2-oxoethyl)-1,4,7-triazonan-1-yl)-5-(*tert*-butoxy)-5-oxopen-tanamido)-3-(4-(3-(4-(((3′-((2-chloro-5-((5-cyanopyridin-3-yl)methoxy)-4-((((*R*)-1-oxo-3-sulfo-1-((2-sulfoethyl)amino)propan-2-yl)amino)methyl)phenoxy)methyl)-2,2′-dimethyl-[1,1′-biphenyl]-3-yl)oxy)methyl)-1*H*-1,2,3-triazol-1-yl)propyl)piperazin-1-yl)-3-oxopropane-1-sulfonic Acid (**7**)

Amine **44** (44.0 mg, 39.4 μmol, 1.00 equiv), (*R*)-NODA-GA(^*t*^Bu)_3_ (32.1 mg, 59.1 μmol, 1.50 equiv), DCC (16.2 mg, 78.7 μmol, 2.00 equiv), pyridine (13.7 μL, 78.7 μmol, 2.00 equiv), and HOBt (5.3 mg, 39.4 μmol, 1.00 equiv) were reacted in abs. DMF (1 mL) according to GP-1. After removing the solvent in vacuo, the residue was used in the next step without purification.

#### 2,2′-(7-((*R*)-1-Carboxy-4-(((*R*)-1-(4-(3-(4-(((3′-((2-chloro-5-((5-cyanopyridin-3-yl)methoxy)-4-((((*R*)-1-oxo-3-sulfo-1-((2-sulfethyl)amino)propan-2-yl)amino)methyl)phenoxy)methyl)-2,2′-dimethyl-[1,1′-biphenyl]-3-yl)oxy)methyl)-1*H*-1,2,3-triazol-1-yl)propyl)piperazin-1-yl)-1-oxo-3-sulfopropan-2-yl)amino)-4-oxobutyl)-1,4,7-triazonane-1,4-diyl)diacetic Acid (**1**)

*tert*-Butyl ester deprotection was performed with the residue from the previous reaction according to GP-4 in 300 μL of the deprotection cocktail. Semipreparative RP-HPLC purification [Agilent Zorbax SB C-18 5 μm 80 Å, 9.4 × 250 mm with 20–70% acetonitrile (0.1% TFA) in water (0.1% TFA) in a linear gradient over 45 min, 6 mL/min, *R*_t_ = 10 min] and subsequent lyophilization yielded the NODA-GA conjugate **1** (5.0 mg, 3.4 μmol, 67% over two steps) as a colorless powder. mp 225 °C (decomposition). *R*_t_ = 8.64 min (system A), purity = 100%. *ṽ* 1772 (w), 1651 (m), 1575 (w), 1505 (w), 1455 (m), 1308 (w), 1169 (s), 1090 (w), 1037 (s), 721 cm^–1^ (w). MS (HR-ESI^+^): exact mass calculated for [M + H]^+^: *m*/*z* 1474.4521, measured: *m*/*z* 1474.4484.

#### (*R*)-2-((4-((3′-((1-(3-(4-(((((9*H*-Fluoren-9-yl)methoxy)carbonyl)(sulfo)-d-alanyl)(sulfo)-d-alanyl)piperazin-1-yl)propyl)-1*H*-1,2,3-triazol-4-yl)methoxy)-2,2′-dimethyl-[1,1′-biphenyl]-3-yl)methoxy)-5-chloro-2-((5-cyanopyridin-3-yl)methoxy)benzyl)amino)-3-oxo-3-((2-sulfoethyl)amino)propane-1-sulfonic Acid (**40**)

Alkyne **13** (24.0 mg, 34.6 μmol, 1.00 equiv) reacted with the linker structure **16** (30.3 mg, 38.1 μmol, 1.10 equiv) with a premixed catalyst consisting of CuSO_4_ (0.6 mg, 3.5 μmol, 0.10 equiv), THPTA (2.8 mg, 5.2 μmol, 0.15 equiv), and sodium ascorbate (34.3 mg, 173 μmol, 5.00 equiv) in a 1:1 mixture of H_2_O/*t*-BuOH (3 mL) at room temperature for 16 h according to GP-2. The solvent was removed in vacuo, and the residue was used in the next step without further purification.

#### (*R*)-2-Amino-3-(((*R*)-1-(4-(3-(4-(((3′-((2-chloro-5-((5-cyanopyridin-3-yl)methoxy)-4-((((*R*)-1-oxo-3-sulfo-1-((2-sulfoethyl)amino)propan-2-yl)amino)methyl)phenoxy)methyl)-2,2′-dimethyl-[1,1′-biphenyl]-3-yl)oxy)methyl)-1*H*-1,2,3-triazol-1-yl)propyl)piperazin-1-yl)-1-oxo-3-sulfopropan-2-yl)amino)-3-oxopropane-1-sulfonic Acid (**45**)

The residue from the previous reaction was dissolved in abs. DMF (2 mL), and the Fmoc group was removed with sodium azide (11.2 mg, 173 μmol, 5.00 equiv) according to GP-3. Semipreparative RP-HPLC purification [Agilent Zorbax SB C-18 5 μm 80 Å, 9.4 × 250 mm with 25–60% acetonitrile (0.1% TFA) in water (0.1% TFA) in a linear gradient over 45 min, 6 mL/min, *R*_t_ = 8 min] with subsequent lyophilization yielded primary amine **45** (27.3 mg, 21.5 μmol, 62% over two steps) as a colorless powder. mp 265 °C (decomposition). *R*_t_ = 8.38 min (system A), purity = 95.4%. ^1^H NMR (400 MHz, DMSO-*d*_6_): δ 9.61 (bs, 1H), 9.36 (bs, 1H), 9.08–9.09 (m, 1H), 9.01–9.03 (m, 3H), 8.80 (t, ^3^*J* = 5.4 Hz, 1H), 8.51 (s, 1H), 8.30 (s, 1H), 8.08 (bs, 3H), 7.52–7.53 (m, 1H), 7.44–7.47 (m, 1H), 7.23–7.27 (m, 2H), 7.16–7.19 (m, 2H), 7.07 (d, ^3^*J* = 7.5 Hz, 1H), 6.71 (d, ^3^*J* = 7.5 Hz, 1H), 5.34–5.45 (m, 2H), 5.24–5.30 (m, 2H), 5.18–5.21 (m, 2H), 5.02–5.04 (m, 1H), 4.40–4.50 (m, 2H), 4.22–4.25 (m, 2H), 3.99–4.02 (m, 3H), 3.37–3.50 (m, 5H), 3.13 (bs, 3H), 2.92–3.02 (m, 4H), 2.72–2.78 (m, 2H), 2.66 (t, ^3^*J* = 7.0, 2H), 2.28 (bs, 2H), 3.11 (s, 3H), 1.80–1.81 ppm (s, 3H). ^13^C NMR (101 MHz, DMSO-*d*_6_): δ 169.3, 166.7, 166.5, 165.4, 158.6, 158.2, 157.8, 156.3, 156.2, 155.5, 155.4, 152.3, 151.8, 143.2, 142.3, 141.6, 139.1, 134.7, 134.5, 132.7, 129.3, 127.8, 126.4, 125.5, 124.5, 124.0, 121.8, 116.9, 116.5, 113.6, 113.1, 113.1, 112.7, 110.8, 109.1, 100.1, 69.8, 66.9, 61.8, 56.4, 53.5, 50.5, 49.8, 46.7, 46.0, 44.4, 36.3, 24.3, 15.3, 12.9 ppm. *ṽ* 1651 (w), 1574 (w), 1505 (w), 1445 (w), 1167 (s), 1091 (w), 1036 (s), 723 cm^–1^ (w). MS (HR-ESI^+^): exact mass calculated for [M + H]^+^: *m*/*z* 1268.2924, measured: *m*/*z* 1268.2915.

#### (*R*)-2-((*R*)-4-(4,7-Bis(2-(*tert*-butoxy)-2-oxoethyl)-1,4,7-triazonan-1-yl)-5-(*tert*-butoxy)-5-oxopentanamido)-3-(((*R*)-1-(4-(3-(4-(((3′-((2-chloro-5-((5-cyanopyridin-3-yl)methoxy)-4-((((*R*)-1-oxo-3-sulfo-1-((2-sulfoethyl)amino)propan-2-yl)amino)methyl)phenoxy)methyl)-2,2′-dimethyl-[1,1′-biphenyl]-3-yl)oxy)methyl)-1*H*-1,2,3-triazol-1-yl)propyl)piperazin-1-yl)-1-oxo-3-sulfopropan-2-yl)amino)-3-oxopropane-1-sulfonic Acid (**8**)

Amine **45** (17.0 mg, 13.4 μmol, 1.00 equiv), (*R*)-NODA-GA(^*t*^Bu)_3_ (14.6 mg, 26.8 μmol, 2.00 equiv), DCC (5.5 mg, 26.8 μmol, 2.00 equiv), pyridine (5.4 μL, 67.0 μmol, 5.00 equiv), and HOBt (1.8 mg, 13.4 μmol, 1.00 equiv) were reacted in abs. DMF (1 mL) according to GP-1. Semipreparative purification was omitted, and the residue was subjected to *tert*-butyl deprotection in the next step.

#### 2,2′-(7-((*R*)-1-Carboxy-4-(((*R*)-1-(((*R*)-1-(4-(3-(4-(((3′-((2-chloro-5-((5-cyanopyridin-3-yl)methoxy)-4-((((*R*)-1-oxo-3-sulfo-1-((2-sulfethyl)amino)propan-2-yl)amino)methyl)phenoxy)methyl)-2,2′-dimethyl-[1,1′-biphenyl]-3-yl)oxy)methyl)-1*H*-1,2,3-triazol-1-yl)propyl)piperazin-1-yl)-1-oxo-3-sulfopropan-2-yl)amino)-1-oxo-3-sulfopropan-2-yl)amino)-4-oxobutyl)-1,4,7-triazonane-1,4-diyl)diacetic Acid (**2**)

*tert*-Butyl ester deprotection was performed according to GP-4 in 500 μL of the deprotection cocktail. Semipreparative RP-HPLC purification [Agilent Zorbax SB C-18 5 μm 80 Å, 9.4 × 250 mm with 20–60% acetonitrile (0.1% TFA) in water (0.1% TFA) in a linear gradient over 45 min, 6 mL/min, *R*_t_ = 13 min] and subsequent lyophilization yielded the NODA-GA-conjugate **2** (9.2 mg, 5.6 μmol, 42% over two steps) as a colorless powder. mp 240 °C (decomposition). *R*_t_ = 8.57 min (system A), purity = 100%. *ṽ* 1651 (m), 1574 (w), 1505 (w), 1455 (w), 1410 (w), 1309 (w), 1166 (s), 1037 (s), 723 cm^–1^ (w). MS (HR-ESI^+^): exact mass calculated for [M + H]^+^: *m*/*z* 1627.4460, measured: *m*/*z* 1627.4447.

#### (*R*)-2-((4-((3′-((1-(3-(4-((((9*H*-Fluoren-9-yl)methoxy)carbonyl)(sulfo)-d-alanyl)piperazin-1-yl)propyl)-1*H*-1,2,3-triazol-4-yl)methoxy)-2,2′-dimethyl-[1,1′-biphenyl]-3-yl)methoxy)-5-chloro-2-((5-(methylsulfonyl)pyridin-3-yl)methoxy)benzyl)amino)-3-oxo-3-((2-sulfoethyl)amino)propane-1-sulfonic Acid (**41**)

Alkyne **14** (27.0 mg, 31.8 μmol, 1.00 equiv) reacted with the linker structure **16** (16.4 mg, 30.2 μmol, 0.95 equiv) with a premixed catalyst consisting of CuSO_4_ (0.8 mg, 4.8 μmol, 0.15 equiv), THPTA (1.4 mg, 3.2 μmol, 0.10 equiv), and sodium ascorbate (31.5 mg, 159 μmol, 5.00 equiv) in a 1:1 mixture of H_2_O/*t*-BuOH (3 mL) at room temperature for 16 h according to GP-2. The solvent was removed in vacuo, and the residue was used in the next step without further purification.

#### (*R*)-2-Amino-3-(4-(3-(4-(((3′-((2-chloro-5-((5-(methylsulfonyl)pyridin-3-yl)methoxy)-4-((((*R*)-1-oxo-3-sulfo-1-((2-sulfoethyl)amino)propan-2-yl)amino)methyl)phenoxy)methyl)-2,2′-dimethyl-[1,1′-biphenyl]-3-yl)oxy)methyl)-1*H*-1,2,3-triazol-1-yl)propyl)piperazin-1-yl)-3-oxopropane-1-sulfonic Acid (**46**)

The residue from the previous reaction was dissolved in abs. DMF (3 mL), and the Fmoc group was removed with sodium azide (10.3 mg, 159 μmol, 5.00 equiv) according to GP-3. Semipreparative RP-HPLC purification [Agilent Zorbax SB C-18 5 μm 80 Å, 9.4 × 250 mm with 25–70% acetonitrile (0.1% TFA) in water (0.1% TFA) in a linear gradient over 45 min, 6 mL/min, *R*_t_ = 9 min] with subsequent lyophilization yielded primary amine **46** (25.7 mg, 21.9 μmol, 69% over two steps) as a colorless powder. mp 250 °C (decomposition). *R*_t_ = 8.87 min (system A), purity = 100%. ^1^H NMR (400 MHz, DMSO-*d*_6_): δ 9.74 (bs, 1H), 9.35 (bs, 1H), 9.10 (s, 1H), 9.06 (d, ^4^*J* = 1.9 Hz, 1H), 8.97 (bs, 1H), 8.75 (t, ^3^*J* = 5.1 Hz, 1H), 8.51 (s, 1H), 8.30 (s, 1H), 8.09–8.10 (m, 3H), 7.46–7.52 (m, 2H), 7.17–7.28 (m, 4H), 7.07 (d, ^3^*J* = 7.6 Hz, 1H), 6.74 (d, ^3^*J* = 7.4 Hz, 1H), 5.41–5.50 (m, 2H), 5.27–5.35 (m, 2H), 5.17–5.24 (m, 2H), 4.51 (t, ^3^*J* = 6.7, 3H), 4.18–4.21 (m, 1H), 4.01 (bs, 3H), 3.55 (bs, 3H), 3.32–3.38 (m, 2H), 3.18 (bs, 2H), 2.97–3.02 (m, 3H), 2.72–2.88 (m, 2H), 2.63 (t, ^3^*J* = 6.5 Hz, 2H), 2.29 (bs, 2H), 2.02–2.03 (m, 3H), 1.80–1.81 ppm (s, 3H). ^13^C NMR (101 MHz, DMSO-*d*_6_): δ 166.3, 165.3, 158.5, 158.2, 156.4, 156.2, 155.4, 155.4, 153.5, 147.1, 143.3, 142.3, 141.6, 136.9, 134.8, 134.8, 134.7, 134.5, 132.8, 129.3, 128.0, 126.4, 125.5, 124.5, 124.0, 121.8, 113.1, 113.1, 112.7, 112.7, 110.8, 100.1, 69.8, 67.3, 61.8, 56.4, 53.2, 50.8, 49.8, 49.7, 47.6, 46.7, 44.3, 43.6, 42.0, 36.2, 24.3, 1.54, 12.8 ppm. IR (ATR): *ṽ* 1672 (m), 1606 (w), 1574 (w), 1504 (w), 1445 (m), 1409 (w), 1305 (m), 1166 (s), 1145 (s), 1035 (s), 967 (w), 766 (w), 721 (w), 597 cm^–1^ (w). MS (HR-ESI^+^): exact mass calculated for [M + H]^+^: *m*/*z* 1170.2808, measured: *m*/*z* 1170.2803.

#### (*R*)-2-((*R*)-4-(4,7-Bis(2-(*tert*-butoxy)-2-oxoethyl)-1,4,7-triazonan-1-yl)-5-(*tert*-butoxy)-5-oxopentanamido)-3-(4-(3-(4-(((3′-((2-chloro-5-((5-(methylsulfonyl)pyridin-3-yl)methoxy)-4-((((*R*)-1-oxo-3-sulfo-1-((2-sulfoethyl)amino)propan-2-yl)amino)methyl)phenoxy)methyl)-2,2′-dimethyl-[1,1′-biphenyl]-3-yl)oxy)methyl)-1*H*-1,2,3-triazol-1-yl)propyl)piperazin-1-yl)-3-oxopropane-1-sulfonic Acid (**9**)

Amine **46** (20.0 mg, 17.1 μmol, 1.00 equiv), (*R*)-NODA-GA(^*t*^Bu)_3_ (18.6 mg, 34.2 μmol, 2.00 equiv), HBTU (9.7 mg, 25.6 μmol, 1.50 equiv), abs. DIPEA (6.0 μL, 34.2 μmol, 2.00 equiv), and HOBt (2.3 mg, 17.1 μmol, 1.00 equiv) were reacted in abs. DMF (1 mL) according to GP-4. The solvent was removed under reduced pressure and used in the next reaction without further purification.

#### 2,2′-(7-((*R*)-1-Carboxy-4-(((*R*)-1-(4-(3-(4-(((3′-((2-chloro-5-((5-(methylsulfonyl)pyridin-3-yl)methoxy)-4-((((*R*)-1-oxo-3-sulfo-1-((2-sulfethyl)amino)propan-2-yl)amino)methyl)phenoxy)methyl)-2,2′-dimethyl-[1,1′-biphenyl]-3-yl)oxy)methyl)-1*H*-1,2,3-triazol-1-yl)propyl)piperazin-1-yl)-1-oxo-3-sulfopropan-2-yl)amino)-4-oxobutyl)-1,4,7-triazonane-1,4-diyl)diacetic Acid (**3**)

*tert*-Butyl ester deprotection was performed according to GP-4 in 400 μL of the deprotection cocktail. Semipreparative RP-HPLC purification [Agilent Zorbax SB C-18 5 μm 80 Å, 9.4 × 250 mm with 20–80% acetonitrile (0.1% TFA) in water (0.1% TFA) in a linear gradient over 45 min, 6 mL/min, *R*_t_ = 11 min] and subsequent lyophilization yielded the NODA-GA-conjugate **3** (13.3 mg, 8.7 μmol, 51% over two steps) as a colorless powder. mp 230 °C (decomposition). *R*_t_ = 9.05 min (system A), purity = 95.0%. IR (ATR): *ṽ* 1726 (w), 1651 (w), 1574 (w), 1505 (w), 1454 (w), 1410 (w), 1305 (m), 1166 (s), 1146 (s), 1036 (s), 768 (w), 727 cm^–1^ (w). MS (HR-ESI^+^): exact mass calculated for [M + H]^+^: *m*/*z* 1528.4377, measured: *m*/*z* 1528.4356.

#### (*R*)-2-((4-((3′-((1-(3-(4-((((9*H*-Fluoren-9-yl)methoxy)carbonyl)(sulfo)-d-alanyl)piperazin-1-yl)propyl)-1*H*-1,2,3-triazol-4-yl)methoxy)-2,2′-dimethyl-[1,1′-biphenyl]-3-yl)methoxy)-5-chloro-2-((5-(methylsulfonyl)pyridin-3-yl)methoxy)benzyl)amino)-3-((2-(diethoxyphosphoryl)ethyl)amino)-3-oxopropane-1-sulfonic Acid (**42**)

Alkyne **15** (34.0 mg, 37.5 μmol, 1.00 equiv) reacted with the linker structure **16** (18.3 mg, 33.8 μmol, 0.90 equiv) with a premixed catalyst consisting of CuSO_4_ (0.9 mg, 5.6 μmol, 0.15 equiv), THPTA (1.6 mg, 3.8 μmol, 0.10 equiv), and sodium ascorbate (37.2 mg, 188 μmol, 5.00 equiv) in a 1:1 mixture of H_2_O/*t*-BuOH (3 mL) at room temperature for 16 h according to GP-2. The solvent was removed in vacuo, and the residue was used in the next step without further purification.

#### (*R*)-2-Amino-3-(4-(3-(4-(((3′-((2-chloro-4-((((*R*)-1-((2-(diethoxyphosphoryl)ethyl)amino)-1-oxo-3-sulfopropan-2-yl)amino)methyl)-5-((5-(methylsulfonyl)pyridin-3-yl)methoxy)phenoxy)methyl)-2,2′-dimethyl-[1,1′-biphenyl]-3-yl)oxy)methyl)-1*H*-1,2,3-triazol-1-yl)propyl)piperazin-1-yl)-3-oxopropane-1-sulfonic Acid (**47**)

The residue from the previous reaction was dissolved in abs. DMF (3 mL), and the Fmoc group was removed with sodium azide (12.2 mg, 188 μmol, 5.00 equiv) according to GP-3. Semipreparative RP-HPLC purification [Agilent Zorbax SB C-18 5 μm 80 Å, 9.4 × 250 mm with 33–88% acetonitrile (0.1% TFA) in water (0.1% TFA) in a linear gradient over 45 min, 6 mL/min, *R*_t_ = 12 min] with subsequent lyophilization yielded primary amine **47** (47.3 mg, 32.6 μmol, 87% over two steps) as a colorless powder. mp 190 °C (decomposition). *R*_t_ = 9.48 min (system A), purity = 100%. ^1^H NMR (400 MHz, DMSO-*d*_6_): δ 9.40 (bs, 1H), 9.11 (d, ^4^*J* = 1.4 Hz, 1H), 9.07 (d, ^4^*J* = 2.0 Hz, 1H), 8.97 (bs, 1H), 8.89 (t, ^3^*J* = 5.5 Hz, 1H), 8.51 (t, ^4^*J* = 1.9 Hz, 1H), 8.29 (s, 1H), 8.11–8.12 (m, 2H), 7.54 (s, 1H), 7.48 (d, ^3^*J* = 7.5 Hz, 1H), 7.24–7.29 (m, 2H), 7.18–7.20 (m, 1H), 7.08 (d, ^3^*J* = 7.3 Hz, 1H), 6.73 (d, ^3^*J* = 7.3 Hz, 1H), 5.41–5.51 (m, 2H), 5.29–5.35 (m, 2H), 5.21–5.26 (m, 2H), 4.50 (t, ^3^*J* = 6.8 Hz, 2H), 4.03–4.06 (m), 3.23–3.33 (m, 2H), 3.16–3.19 (m, 1H), 2.95 (m, 2H), 2.75–2.84 (m, 2H), 2.27–2.32 (m, 2H), 1.93–2.03 (m, 4H), 1.82 (s, 3H), 1.24 (t, ^3^*J* = 7.0 Hz, 4H). ^13^C NMR (101 MHz, DMSO-*d*_6_): δ 166.3, 165.6, 158.9, 158.5, 158.2, 157.8, 156.5, 156.2, 155.5, 153.5, 147.2, 143.2, 142.3, 141.6, 136.9, 134.7, 134.6, 134.5, 132.8, 132.7, 129.4, 127.9, 126.4, 125.5, 124.5, 123.9, 121.8, 113.1, 112.7, 110.7, 100.1, 69.8, 67.4, 61.7, 61.2, 61.2, 56.3, 53.2, 50.5, 49.8, 47.7, 46.7, 44.2, 43.6, 42.0, 33.5, 25.5, 24.3, 2.41., 16.3, 15.3, 12.9 ppm. ^31^P NMR (162 MHz, DMSO-*d*_6_): δ 28.2 ppm. IR (ATR): *ṽ* 1673 (m), 1606 (w), 1574 (w), 1504 (w), 1445 (w), 1409 (w), 1306 (w), 1173 (s), 1145 (s), 1033 (m), 966 (m), 798 (w), 720 cm^–1^ (w). MS (HR-ESI^+^): exact mass calculated for [M + H]^+^: *m*/*z* 1226.3529, measured: *m*/*z* 1226.3517.

#### (*R*)-2-((*R*)-4-(4,7-Bis(2-(*tert*-butoxy)-2-oxoethyl)-1,4,7-triazonan-1-yl)-5-(*tert*-butoxy)-5-oxopentanamido)-3-(4-(3-(4-(((3′-((2-chloro-4-((((*R*)-1-((2-(diethoxyphosphoryl)ethyl)amino)-1-oxo-3-sulfopropan-2-yl)amino)methyl)-5-((5-(methylsulfonyl)pyridin-3-yl)methoxy)phenoxy)methyl)-2,2′-dimethyl-[1,1′-biphenyl]-3-yl)oxy)methyl)-1*H*-1,2,3-triazol-1-yl)propyl)piperazin-1-yl)-3-oxopropane-1-sulfonic Acid (**11**)

Amine **47** (10.0 mg, 8.2 μmol, 1.00 equiv), (*R*)-NODA-GA(^*t*^Bu)_3_ (8.9 mg, 16.3 μmol, 2.00 equiv), HBTU (4.6 mg, 12.2 μmol, 1.50 equiv), abs. DIPEA (2.8 μL, 16.3 μmol, 2.00 equiv), and HOBt (1.1 mg, 8.2 μmol, 1.00 equiv) were reacted in abs. DMF (1 mL) according to GP-1. Semipreparative purification [Agilent Zorbax SB C-18 5 μm 80 Å, 9.4 × 250 mm with 30–90% acetonitrile (0.1% TFA) in water (0.1% TFA) in a linear gradient over 45 min, 6 mL/min, *R*_t_ = 13 min] with subsequent lyophilization yielded NODA-GA-tris(^*t*^Bu)_3_-conjugate **11** (9.0 mg, 5.1 μmol, 63%) as a colorless oil. *R*_t_ = 11.50 min (system A), purity = 95.3%. IR (ATR): *ṽ* 1727 (m), 1674 (s), 1577 (w), 1455 (w), 1393 (w), 1370 (w), 1309 (w), 1200 (s), 1148 (s), 1040 (m), 696 (w), 834 (w), 799 (w), 720 cm^–1^ (w). MS (HR-ESI^+^): exact mass calculated for [M + H]^+^: *m*/*z* 1753.6943, measured: *m*/*z* 1753.6947.

#### 2,2′-(7-((*R*)-1-Carboxy-4-(((*R*)-1-(4-(3-(4-(((3′-((2-Chloro-5-((5-(methylsulfonyl)pyridin-3-yl)methoxy)-4-((((*R*)-1-oxo-1-((2-phosphonoethyl)amino)-3-sulfopropan-2-yl)amino)methyl)phenoxy)methyl)-2,2′-dimethyl-[1,1′-biphenyl]-3-yl)oxy)methyl)-1*H*-1,2,3-triazol-1-yl)propyl)piperazin-1-yl)-1-oxo-3-sulfopropan-2-yl)amino)-4-oxobutyl)-1,4,7-triazonane-1,4-diyl)diacetic Acid (**5**)

Dealkylation of phosphonate esters and subsequent *tert*-butyl deprotection of NODA-GA tris(^*t*^Bu)_3_-conjugate **11** (9.0 mg, 5.1 μmol, 1.00 equiv) were performed with trimethylsilyl bromide (8.1 μL, 66.8 μmol, 13.0 equiv) and 400 μL of the deprotection cocktail, respectively, according to GP-5. Semipreparative RP-HPLC purification [Agilent Zorbax SB C-18 5 μm 80 Å, 9.4 × 250 mm with 20–80% acetonitrile (0.1% TFA) in water (0.1% TFA) in a linear gradient over 45 min, 6 mL/min, *R*_t_ = 12 min] and subsequent lyophilization yielded the NODA-GA-conjugate **11** (4.6 mg, 3.0 μmol, 57% over two steps) as a colorless powder. mp 215 °C (decomposition). *R*_t_ = 15.88 min (system B), purity = 100%. IR (ATR): *ṽ* 1650 (m), 1573 (w), 1502 (w), 1451 (w), 1408 (w), 1305 (m), 1145 (s), 1036 (m), 768 (w), 719 cm^–1^ (w). MS (HR-ESI^+^): exact mass calculated for [M + H]^+^: *m*/*z* 1528.4472, measured: *m*/*z* 1528.4458.

#### (*R*)-2-((4-((3′-((1-(3-(4-((*R*)-2-((((9*H*-Fluoren-9-yl)methoxy)carbonyl)amino)-3-(diethoxyphosphoryl)propanoyl)piperazin-1-yl)propyl)-1*H*-1,2,3-triazol-4-yl)methoxy)-2,2′-dimethyl-[1,1′-biphenyl]-3-yl)methoxy)-5-chloro-2-((5-(methylsulfonyl)pyridin-3-yl)methoxy)benzyl)amino)-3-((2-(diethoxyphosphoryl)ethyl)amino)-3-oxopropane-1-sulfonic Acid (**43**)

Alkyne **15** (60.0 mg, 66.2 μmol, 1.00 equiv) reacted with the linker structure 1**8** (60.0 mg, 66.2 μmol, 0.90 equiv) with a premixed catalyst consisting of CuSO_4_ (1.6 mg, 9.9 μmol, 0.15 equiv), THPTA (2.9 mg, 6.6 μmol, 0.10 equiv), and sodium ascorbate (65.6 mg, 331 μmol, 5.00 equiv) in a 1:1 mixture of H_2_O/*t*-BuOH (3 mL) at room temperature for 16 h according to GP-2. The solvent was removed in vacuo, and the residue was used in the next step without further purification.

#### (*R*)-2-((4-((3′-((1-(3-(4-((*R*)-2-Amino-3-(diethoxyphosphoryl)propanoyl)piperazin-1-yl)propyl)-1*H*-1,2,3-triazol-4-yl)methoxy)-2,2′-dimethyl-[1,1′-biphenyl]-3-yl)methoxy)-5-chloro-2-((5-(methylsulfonyl)pyridin-3-yl)methoxy)benzyl)amino)-3-((2-(diethoxyphosphoryl)ethyl)amino)-3-oxopropane-1-sulfonic Acid (**48**)

The residue from the previous reaction was dissolved in abs. DMF (3 mL), and the Fmoc group was removed with sodium azide (21.5 mg, 331 μmol, 5.00 equiv) according to GP-3. Semipreparative RP-HPLC purification [Agilent Zorbax SB C-18 5 μm 80 Å, 9.4 × 250 mm with 30–70% acetonitrile (0.1% TFA) in water (0.1% TFA) in a linear gradient over 45 min, 6 mL/min, *R*_t_ = 9 min] with subsequent lyophilization yielded primary amine **48** (19.0 mg, 14.8 μmol, 50% over two steps) as a colorless powder. mp 225 °C (decomposition). *R*_t_ = 9.74 min (system A), purity = 98.0%. ^1^H NMR (400 MHz, DMSO-*d*_6_): δ 9.40 (bs, 2H), 9.11 (s, 1H), 9.07 (s, 1H), 8.98 (bs, 1H), 8.90 (t, ^3^*J* = 5.3 Hz, 1H), 8.51 (s, 1H), 8.29–8.35 (m, 4H), 7.54 (s, 1H), 7.49 (d, ^3^*J* = 7.4 Hz, 1H), 7.24–7.29 (m, 3H), 7.19 (d, ^3^*J* = 8.2 Hz, 1H), 7.08 (d, ^3^*J* = 7.5 Hz, 1H), 6.73 (d, ^3^*J* = 7.3 Hz, 1H), 5.41–5.51 (m, 3H), 5.26–5.35 (m, 3H), 5.21 (s, 2H), 4.63 (bs, 2H), 4.50 (t, ^3^*J* = 6.6 Hz, 3H), 4.20–4.23 (m, 1H), 3.98–4.02 (m), 3.25–3.31 (m, 3H), 3.14 (bs, 3H), 2.75–2.81 (m, 2H), 2.29–2.35 (m, 4H), 1.99 (s, 3H), 1.93–1.97 (m, 3H), 1.81 (s, 3H), 1.71–1.74 (m, 4H), 1.24 ppm (t, ^3^*J* = 7.0 Hz, 12H). ^13^C NMR (101 MHz, DMSO-*d*_6_): δ 166.5, 165.6, 160.8, 158.8, 158.7, 158.5, 158.2, 156.4, 156.2, 155.5, 153.5, 147.1, 143.2, 142.3, 141.6, 136.9, 134.7, 134.6, 134.5, 132.7, 132.7, 129.3, 127.8, 126.4, 125.5, 124.5, 123.9, 121.8, 118.3, 115.4, 113.0, 112.6, 110.7, 100.1, 69.8, 67.4, 62.1, 62.0, 61.7, 61.2, 61.1, 56.3, 53.2, 50.7, 49.7, 46.7, 45.9, 45.8, 44.2, 43.6, 42.6, 33.5, 27.4, 25.9, 25.9, 25.4, 24.5, 24.1, 16.3, 16.2, 16.1, 16.1, 15.3, 12.8 ppm. ^31^P NMR (162 MHz, DMSO-*d*_6_): δ 28.2 (s, 1H), 26.8 ppm, (s, 1H). IR (ATR): *ṽ* 1673 (s), 1606 (w), 1575 (w), 1504 (w), 1445 (w), 1409 (w), 1307 (m), 1199 (s), 1174 (s), 1145 (s), 1021 (s), 967 (m), 830 (w), 798 (m), 720 cm^–1^ (m). MS (HR-ESI^+^): exact mass calculated for [M + H]^+^: *m*/*z* 1282.4250, measured: *m*/*z* 1282.4251.

#### (*R*)-2-((4-((3′-((1-(3-(4-((*R*)-2-((*R*)-4-(4,7-Bis(2-(*tert*-butoxy)-2-oxoethyl)-1,4,7-triazonan-1-yl)-5-(*tert*-butoxy)-5-oxopentanamido)-3-(diethoxyphosphoryl)propanoyl)piperazin-1-yl)propyl)-1*H*-1,2,3-triazol-4-yl)methoxy)-2,2′-dimethyl-[1,1′-biphenyl]-3-yl)methoxy)-5-chloro-2-((5-(methylsulfonyl)pyridin-3-yl)methoxy)benzyl)amino)-3-((2-(diethoxyphosphoryl)ethyl)amino)-3-oxopropane-1-sulfonic Acid (**12**)

Amine **48** (20.0 mg, 15.6 μmol, 1.00 equiv), (*R*)-NODA-GA(^*t*^Bu)_3_ (17.0 mg, 31.2 μmol,2.00 equiv), HBTU (8.9 mg, 23.4 μmol, 1.50 equiv), abs. DIPEA (5.4 μL, 31.2 μmol, 2.00 equiv), and HOBt (4.2 mg, 15.6 μmol, 1.00 equiv) were reacted in abs. DMF (1 mL) according to GP-1. Semipreparative purification [Agilent Zorbax SB C-18 5 μm 80 Å, 9.4 × 250 mm with 40–90% acetonitrile (0.1% TFA) in water (0.1% TFA) in a linear gradient over 45 min, 6 mL/min, *R*_t_ = 8 min] with subsequent lyophilization yielded NODA-GA-tris(^*t*^Bu)_3_-conjugate **12** (25.0 mg, 13.8 μmol, 89%) as a colorless powder. mp 140 °C (decomposition). *R*_t_ = 11.70 min (system A), purity = 100%. IR (ATR): *ṽ* 1727 (w), 1678 (m), 1578 (w), 1454 (w), 1393 (w), 1369 (w), 1308 (w), 1251 (m), 1199 (m), 1148 (s), 1026 (m), 967 (w), 889 (w), 831 (w), 799 (m), 719 cm^–1^ (m). MS (HR-ESI^+^): exact mass calculated for [M + H]^+^: *m*/*z* 1808.7664, measured: *m*/*z* 1808.7684.

#### 2,2′-(7-((*R*)-1-Carboxy-4-(((*R*)-1-(4-(3-(4-(((3′-((2-chloro-5-((5-(methylsulfonyl)pyridin-3-yl)methoxy)-4-((((*R*)-1-oxo-1-((2-phosphonoethyl)amino)-3-sulfopropan-2-yl)amino)methyl)phenoxy)methyl)-2,2′-dimethyl-[1,1′-biphenyl]-3-yl)oxy)methyl)-1*H*-1,2,3-triazol-1-yl)propyl)piperazin-1-yl)-1-oxo-3-phosphonopropan-2-yl)amino)-4-oxobutyl)-1,4,7-triazonane-1,4-diyl)diacetic Acid (**6**)

Dealkylation of phosphonate esters and subsequent *tert*-butyl deprotection of NODA-GA tris(^*t*^Bu)_3_-conjugate **12** (20.0 mg, 11.1 μmol, 1.00 equiv) were performed with trimethylsilyl bromide (19.0 μL, 144 μmol, 13.0 equiv) and 400 μL of the deprotection cocktail, respectively, according to GP-5. Semipreparative RP-HPLC purification [Agilent Zorbax SB C-18 5 μm 80 Å, 9.4 × 250 mm with 25–80% acetonitrile (0.1% TFA) in water (0.1% TFA) in a linear gradient over 45 min, 6 mL/min, *R*_t_ = 8 min] and subsequent lyophilization yielded the NODA-GA-conjugate **6** (9.1 mg, 5.9 μmol, 53% over two steps) as a colorless powder. mp 220 °C (decomposition). *R*_t_ = 16.09 min (system B), purity = 100%. IR (ATR): *ṽ* 1667 (m), 1651 (m), 1574 (w), 1505 (w), 1454 (w), 1409 (w), 1305 (m), 1170 (s), 1144 (s), 1036 (m), 797 (w), 719 cm^–1^(w). MS (HR-ESI^+^): exact mass calculated for [M + H]^+^: *m*/*z* 1527.4534, measured: *m*/*z* 1527.4514.

#### (*R*)-2-((4-((3′-((1-(3-(4-((*R*)-4-(4,7-Bis(2-(*tert*-butoxy)-2-oxoethyl)-1,4,7-triazonan-1-yl)-5-(*tert*-butoxy)-5-oxopentanoyl)piperazin-1-yl)propyl)-1*H*-1,2,3-triazol-4-yl)methoxy)-2,2′-dimethyl-[1,1′-biphenyl]-3-yl)methoxy)-5-chloro-2-((5-(methylsulfonyl)pyridin-3-yl)methoxy)benzyl)amino)-3-((2-(diethoxyphosphoryl)ethyl)amino)-3-oxopropane-1-sulfonic Acid (**10**)

Alkyne **15** (15.0 mg, 16.5 μmol, 1.00 equiv) reacted with the linker structure **23** (9.8 mg, 14.1 μmol, 0.85 equiv) with a premixed catalyst consisting of CuSO_4_ (0.4 mg, 2.5 μmol, 0.15 equiv), THPTA (0.7 mg, 1.7 μmol, 0.10 equiv), and sodium ascorbate (16.4 mg, 82.7 μmol, 5.00 equiv) in a 1:1 mixture of H_2_O/*t*-BuOH (2 mL) at room temperature for 16 h according to GP-2. The solvent was removed in vacuo, and the residue was purified by semipreparative RP-HPLC purification (Agilent Zorbax SB C-18 5 μm 80 Å, 9.4 × 250 mm with 32–90% acetonitrile (0.1% TFA) in water (0.1% TFA) in a linear gradient over 45 min, 6 mL/min, *R*_t_ = 15 min), and subsequent lyophilization yielded the NODA-GA-tris(^*t*^Bu)_3_-conjugate **10** (11.0 mg, 6.8 μmol, 41%) as a colorless powder. mp 155 °C (decomposition). *R*_t_ = 11.90 min (system A), purity = 95.7%. *ṽ* 1726 (w), 1681 (m), 1578 (w), 1455 (w), 1393 (w), 1369 (w), 1308 (w), 1251 (m), 1200 (m), 1147 (s), 1027 (m), 967 (w), 832 (w), 799 (m), 720 cm^–1^ (m). MS (HR-ESI^+^): exact mass calculated for [M + H]^+^: *m*/*z* 1600.7004, measured: *m*/*z* 1600.6986.

#### 2,2′-(7-((*R*)-1-Carboxy-4-(4-(3-(4-(((3′-((2-chloro-5-((5-(methylsulfonyl)pyridin-3-yl)methoxy)-4-((((*R*)-1-oxo-1-((2-phosphonoethyl)amino)-3-sulfopropan-2-yl)amino)methyl)phenoxy)methyl)-2,2′-dimethyl-[1,1′-biphenyl]-3-yl)oxy)methyl)-1*H*-1,2,3-triazol-1-yl)propyl)piperazin-1-yl)-4-oxobutyl)-1,4,7-triazonane-1,4-diyl)diacetic Acid (**4**)

Dealkylation of phosphonate esters and subsequent *tert*-butyl deprotection of NODA-GA tris(^*t*^Bu)_3_-conjugate **10** (11.0 mg, 6.9 μmol, 1.00 equiv) were performed with trimethylsilyl bromide (18.2 μL, 138 μmol, 20.0 equiv) and 400 μL of the deprotection cocktail, respectively, according to GP-5. Semipreparative RP-HPLC purification [Agilent Zorbax SB C-18 5 μm 80 Å, 9.4 × 250 mm with 25–80% acetonitrile (0.1% TFA) in water (0.1% TFA) in a linear gradient over 45 min, 6 mL/min, *R*_t_ = 8 min] and subsequent lyophilization afforded the NODA-GA conjugate **4** (5.7 mg, 4.1 μmol, 54% over two steps) as a colorless powder. mp 220 °C (decomposition). *R*_t_ = 16.09 min (system B), purity = 100%. *ṽ* 1678 (m), 1643 (m), 1579 (w), 1443 (m), 1408 (m), 1307 (m), 1199 (s), 1173 (s), 1145 (s), 1039 (m), 890 (w), 799 (w), 720 (w). MS (HR-ESI^+^): exact mass calculated for [M + H]^+^: *m*/*z* 1376.4500, measured: *m*/*z* 1376.4484.

## Supporting Information Available

Methods, synthetic procedures of literature-unknown compounds, copies of ^1^H and ^13^C NMR, IR, and HR-MS spectra as well as HPLC chromatograms of intermediate and final compounds, radio-HPLC chromatograms, stability assay and *K*_D_ measurement results, micro-PET images of all final radioligands, chemical structures of BMS-1166 and WL12, ^1^H, ^13^C, and ^31^P NMR spectra, IR spectra, HRMS, and HPLC chromatograms for the synthesized compounds, radio-HPLC chromatograms of radiolabeled compounds, including their stability studies in PBS and human serum, FACS analysis of PC3 PD-L1-overexpressing and mock cells, immunostaining of tumor xenografts, saturation binding and real-time binding assay data, qualitative PET scans, data of dynamic [^18^F]FDG uptake scans as a surrogate of blood flow, and metabolization of [^64^Cu]Cu-**3** (PDF)
Molecular formula strings for the synthesized compounds (CSV)

## Abbreviations

BSA: bovine serum albumin
CuAAC: copper-catalyzed azide–alkyne cycloaddition
PD-L1: programmed death ligand 1
PET: positron emission tomography
SUV: standard uptake value
