# Supplementary material for: Design, Synthesis, and Biological Evaluation of Small-Molecule-Based Radioligands with Improved Pharmacokinetic Properties for Imaging of Programmed Death Ligand 1
Source: J Med Chem. 2023 Dec 1;66(23):15894–915. doi: 10.1021/acs.jmedchem.3c01355 (PMC10726354; doi:10.1021/acs.jmedchem.3c01355)
Supplement: Supplementary file 1 — jm3c01355_si_001.pdf [file jm3c01355_si_001.pdf]

Supporting Information

Design, Synthesis, and Biological Evaluation of Small Molecule Based Radioligands with Improved Pharmacokinetic Properties for Imaging of Programmed Death Ligand 1

Fabian Krutzek[a], Cornelius K. Donat[a], Martin Ullrich[a], Sven Stadlbauer[a,b]*

[a] Helmholtz-Zentrum Dresden-Rossendorf, Institute of Radiopharmaceutical Cancer Research, Bautzner Landstraße 400, 01328 Dresden, Germany

[b] Faculty of Chemistry and Food Chemistry, School of Science, Technische Universität Dresden, Mommsenstraße 4, 01069 Dresden, Germany

* Corresponding author. E-mail address: s.stadlbauer@hzdr.de

Content

1. Chemical Structures 2
2. ^{1}H and ^{13}C NMR spectra of literature unknown compounds 3
3. IR spectra of literature unknown compounds 23
4. HR-MS spectra of literature unknown compounds 32
5. HPLC chromatograms of HPLC purified compounds 42
6. Radio-HPLC Chromatograms 52
7. Stability of radiolabeled compounds 54
   7.1 Kinetic stability in PBS (pH 7.4) 54
   7.2 Proteolytic stability in human serum 55
8. *In vitro*: FACS analysis of PC3 PD-L1 positive and mock cells, immunostaining of tumor xenografts 55
9. *In vitro*: Saturation binding assays 58
10. *In vitro*: Real-time binding assay 59
11. *In vivo*: Qualitative PET scans 61
12. *In vivo*: Dynamic [^{18}F]FDG uptake as surrogate measure of blood flow 65
13. *In vivo*: metabolization of [^{64}Cu]Cu-**3** 67
14. References 71

1. Chemical Structures

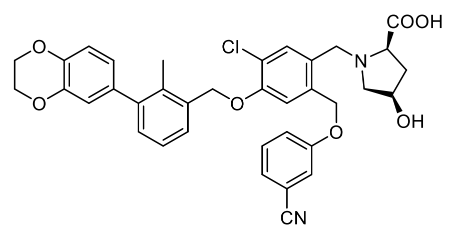

BMS-1166

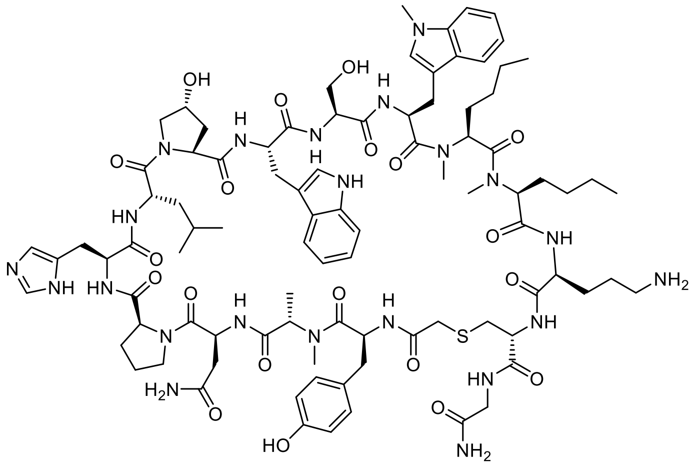

WL12

Chart 1: Chemical structures of PD-L1 inhibitor BMS-1166[1] and cyclic peptide WL12[2]

2. ^{1}H and ^{13}C NMR spectra of literature unknown compounds

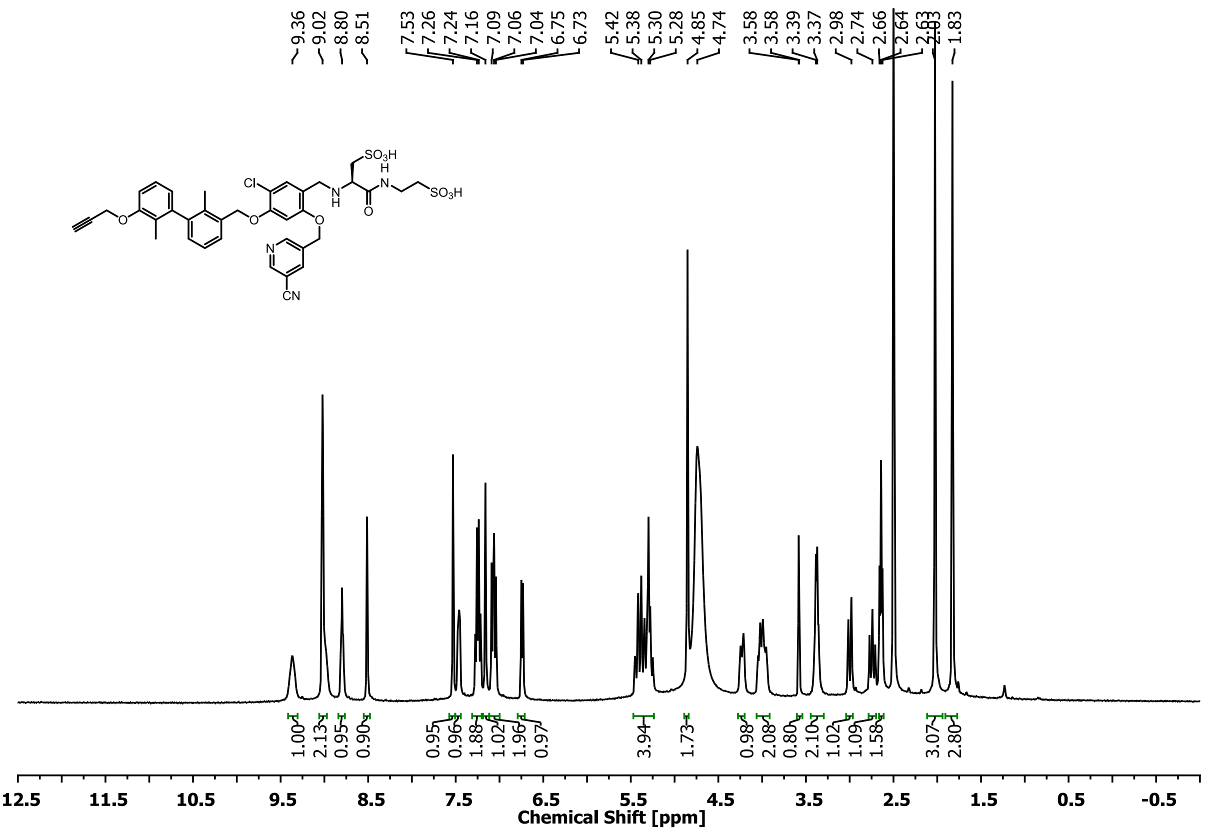

Figure S1: ^{1}H NMR spectrum (DMSO-d_6, 600 MHz, 298 K) of compound 13.

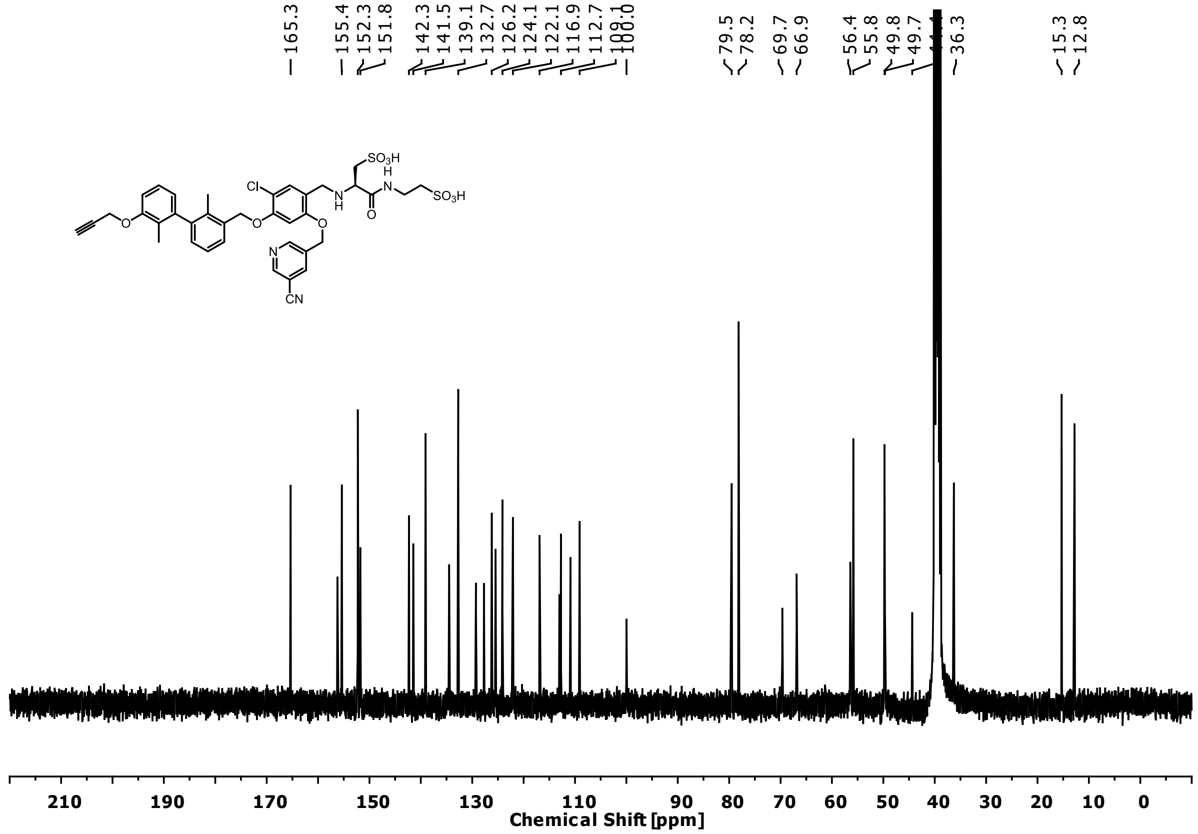

Figure S2: ^{13}C NMR spectrum (DMSO-d_6, 151 MHz, 298 K) of compound 13.

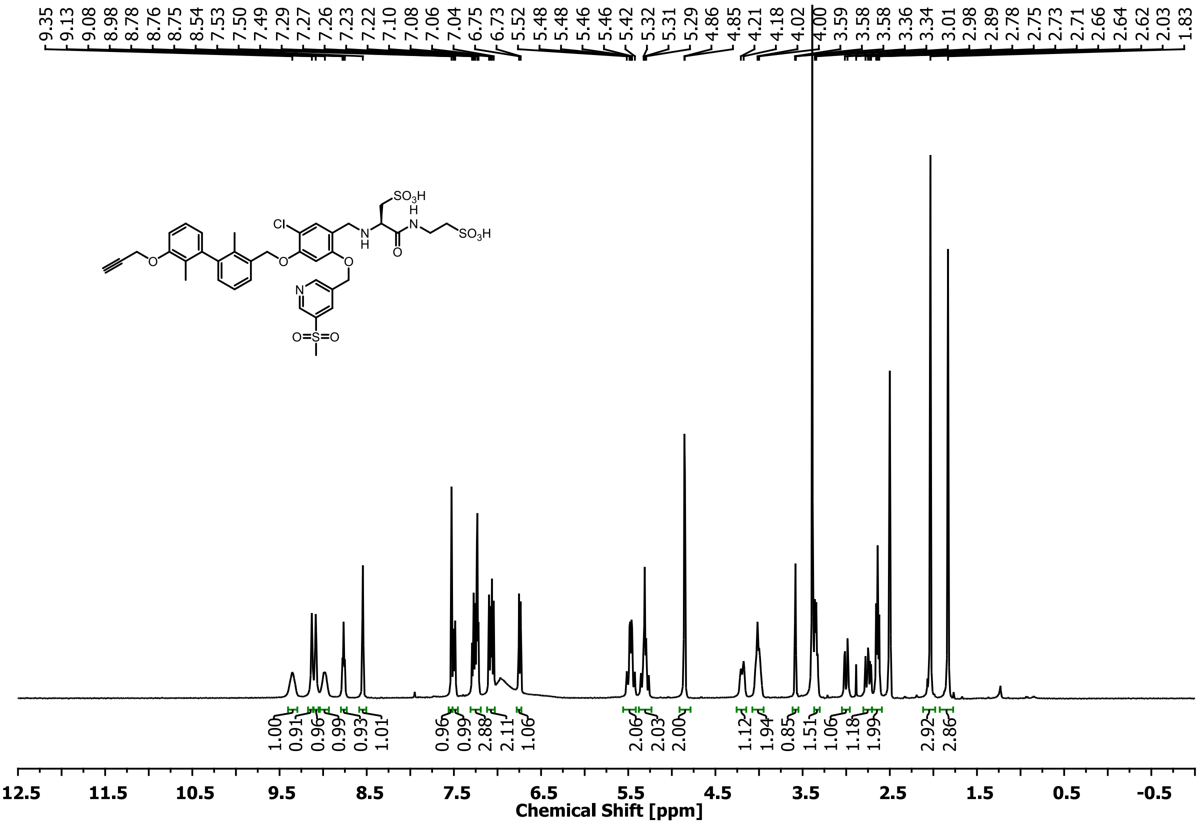

Figure S3: ^{1}H NMR spectrum (DMSO-d_6, 600 MHz, 298 K) of compound 14.

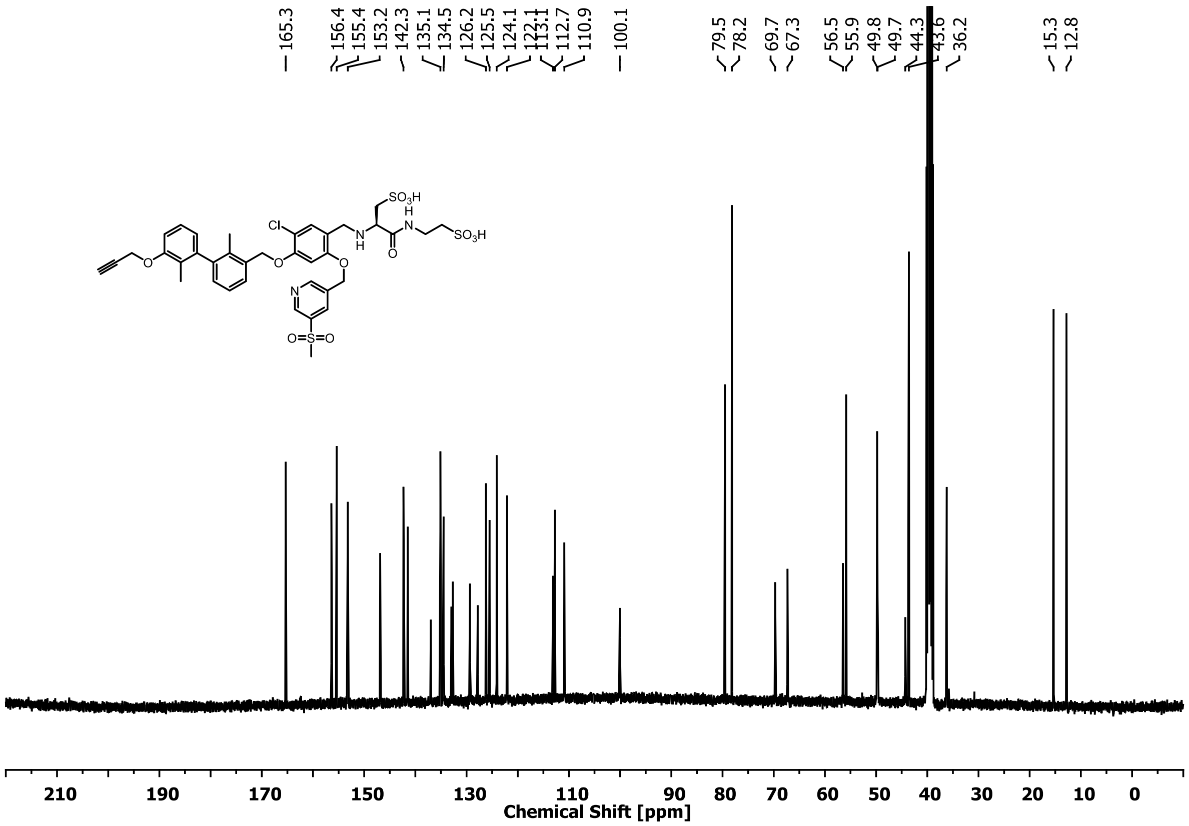

Figure S4: ^{13}C NMR spectrum (DMSO-d_6, 151 MHz, 298 K) of compound 14.

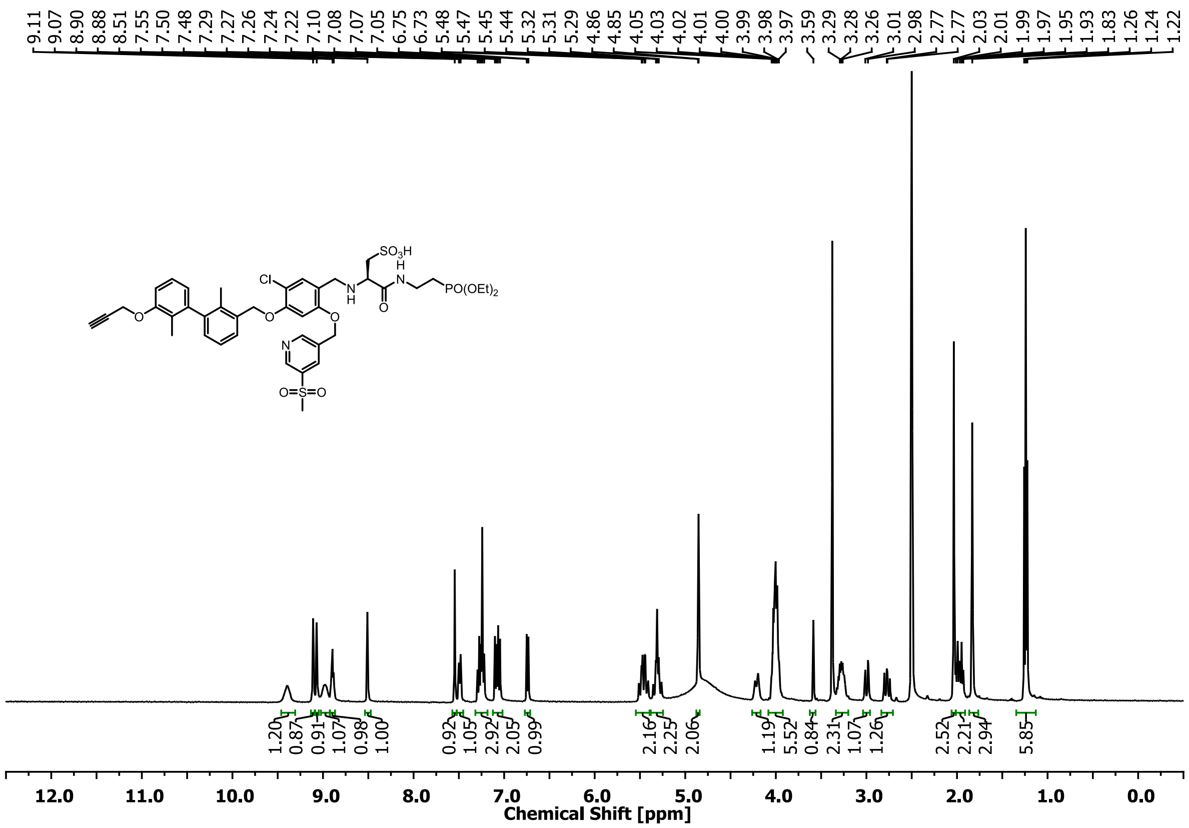

Figure S5: ^{1}H NMR spectrum (DMSO-d_6, 600 MHz, 298 K) of compound 15.

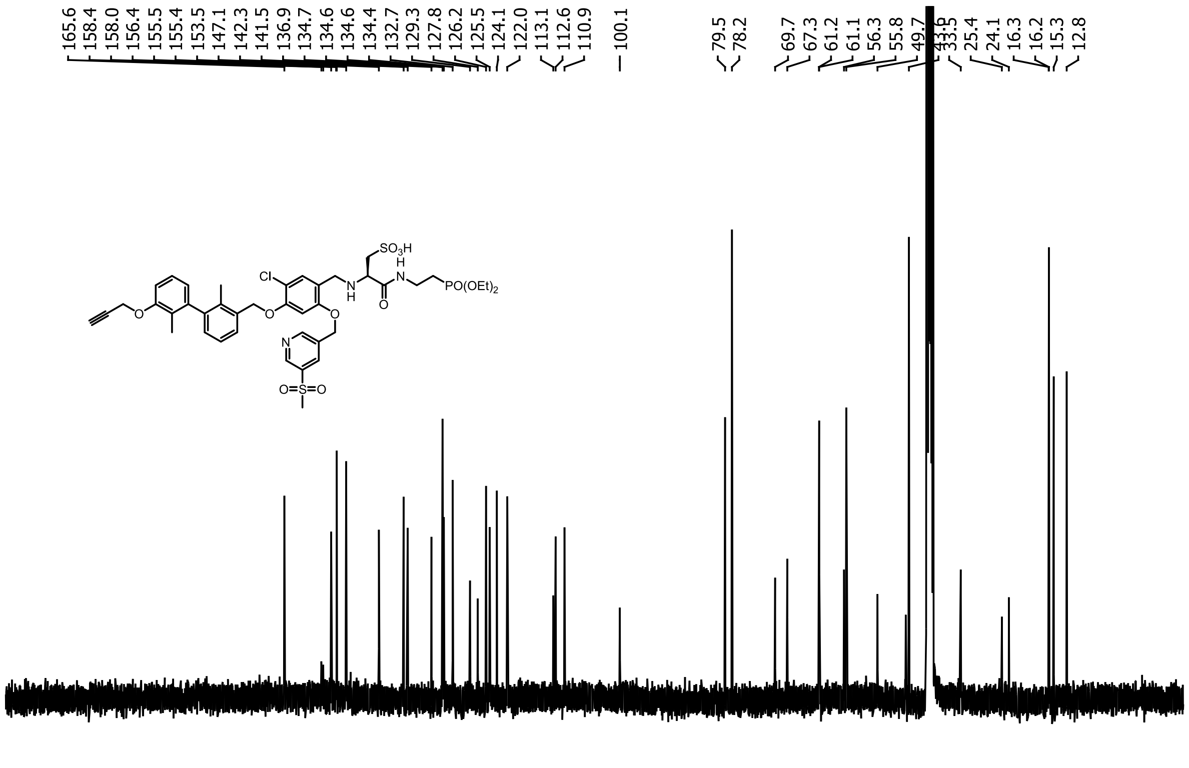

Figure S6: ^{13}C NMR spectrum (DMSO-d_6, 151 MHz, 298 K) of compound 15.

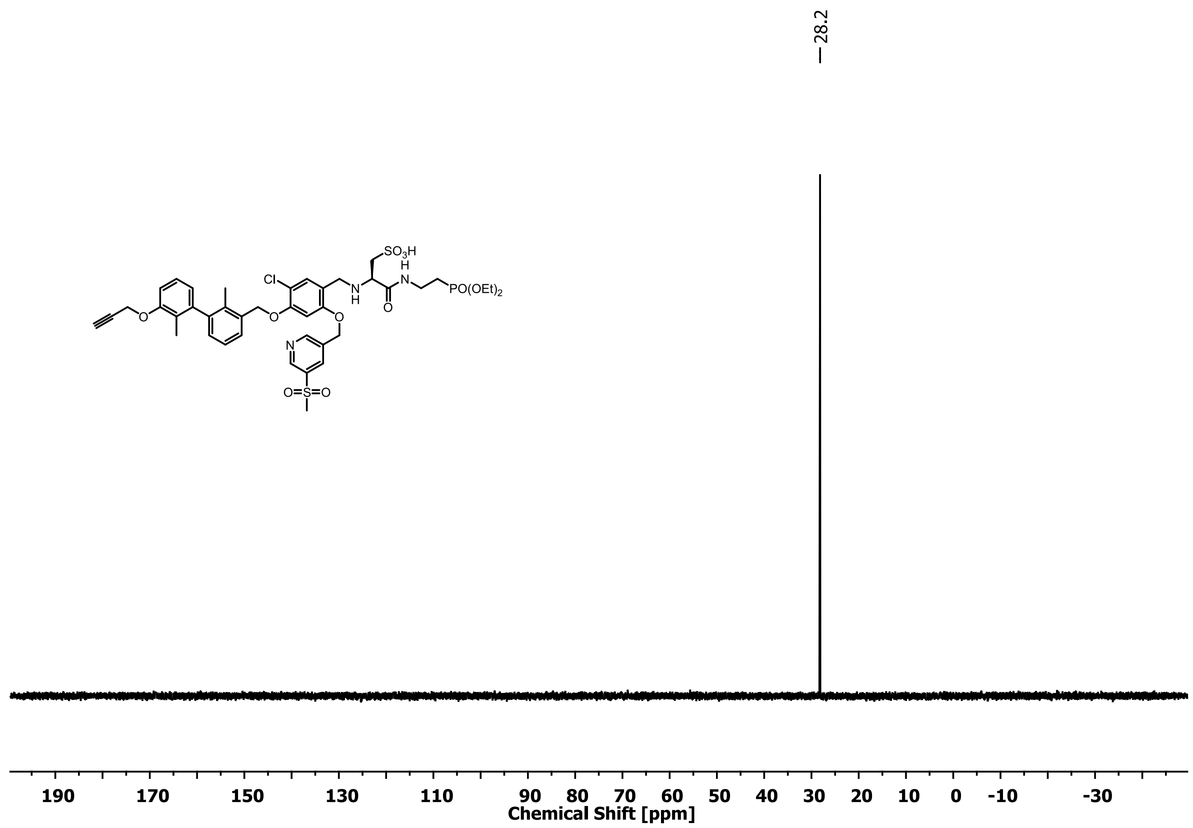

Figure S7: ^{31}P NMR spectrum (DMSO-d_6, 162 MHz, 298 K) of compound 15.

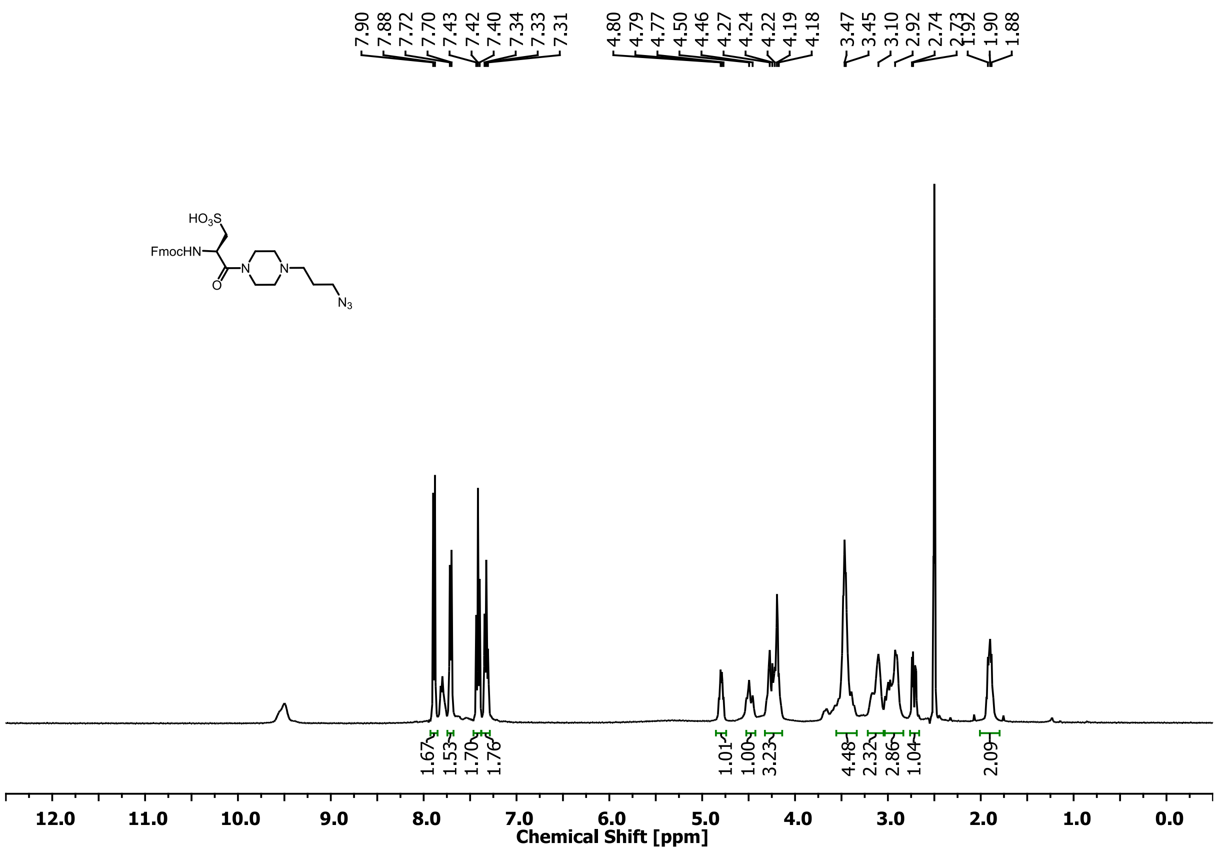

Figure S8: ^{1}H NMR spectrum (DMSO-d_6, 600 MHz, 298 K) of compound 16.

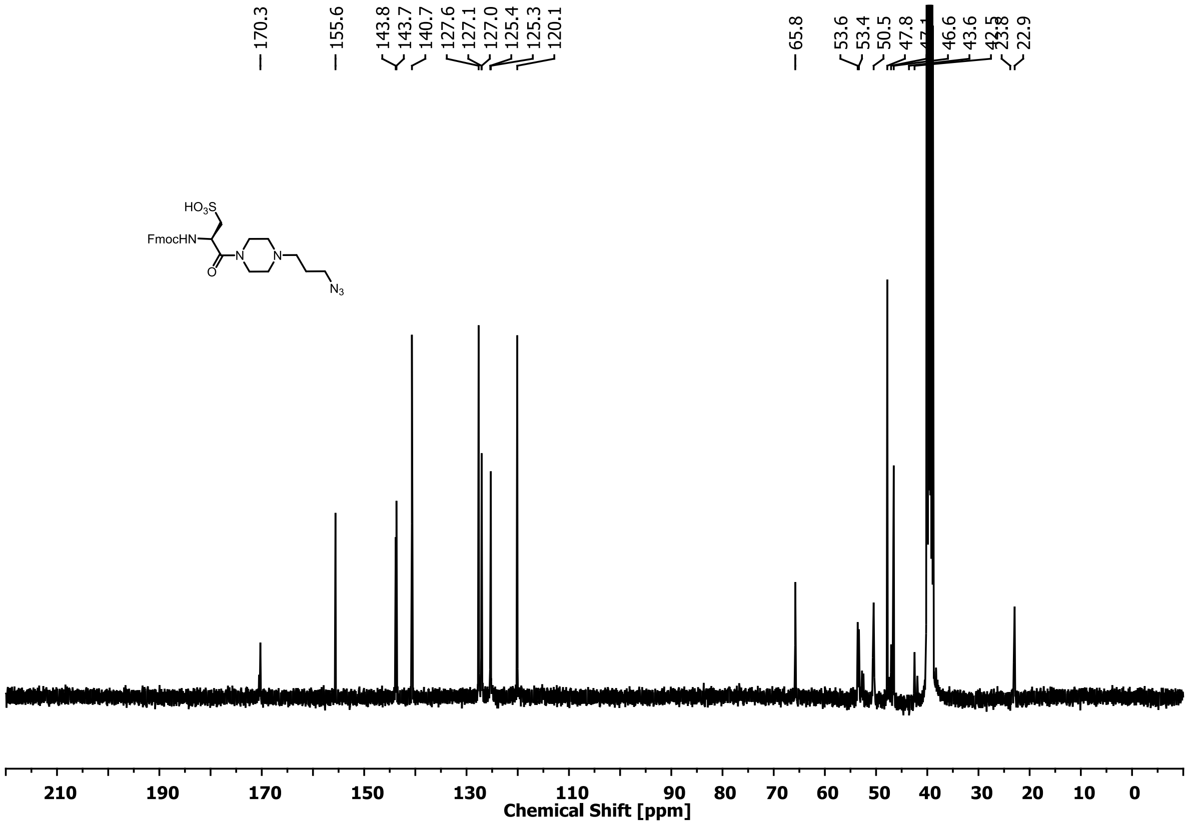

Figure S9: **^{13}C NMR spectrum (DMSO-d_6, 151 MHz, 298 K) of compound 16.**

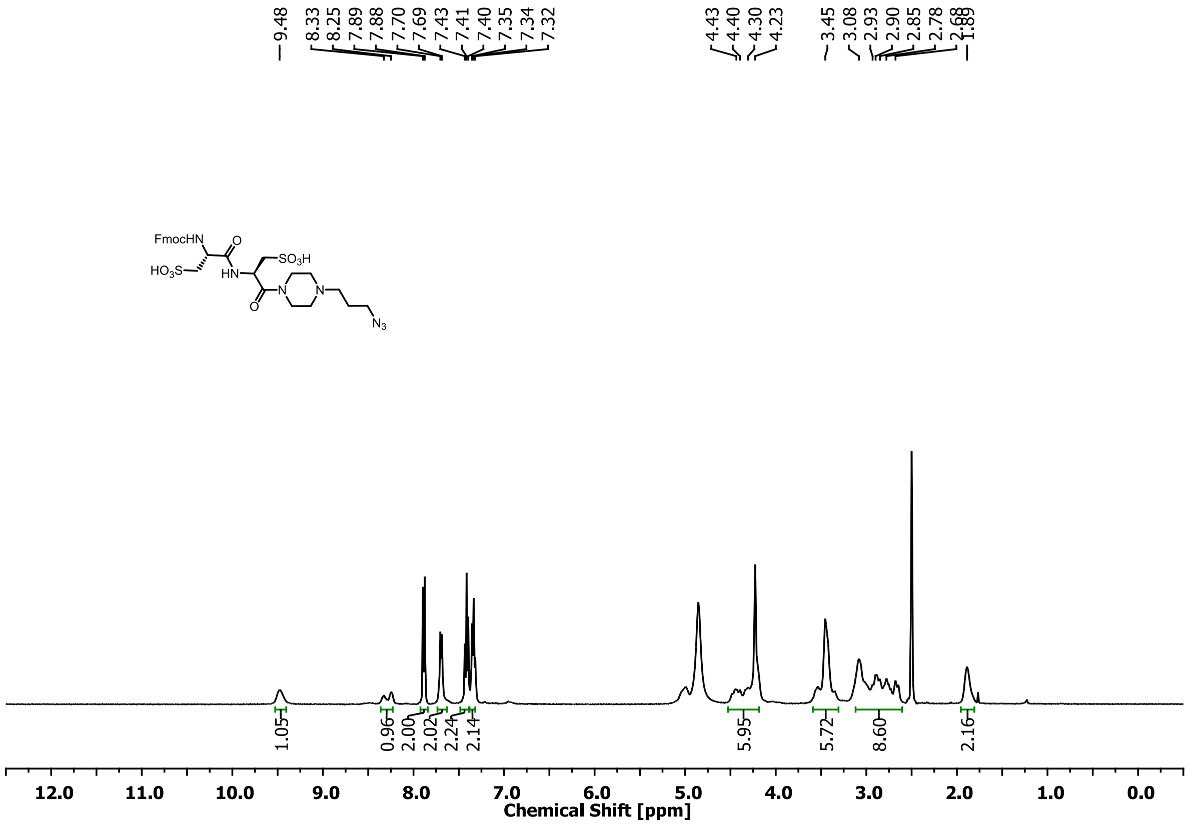

Figure S10: **^{1}H NMR spectrum (DMSO-d_6, 600 MHz, 298 K) of compound 17.**

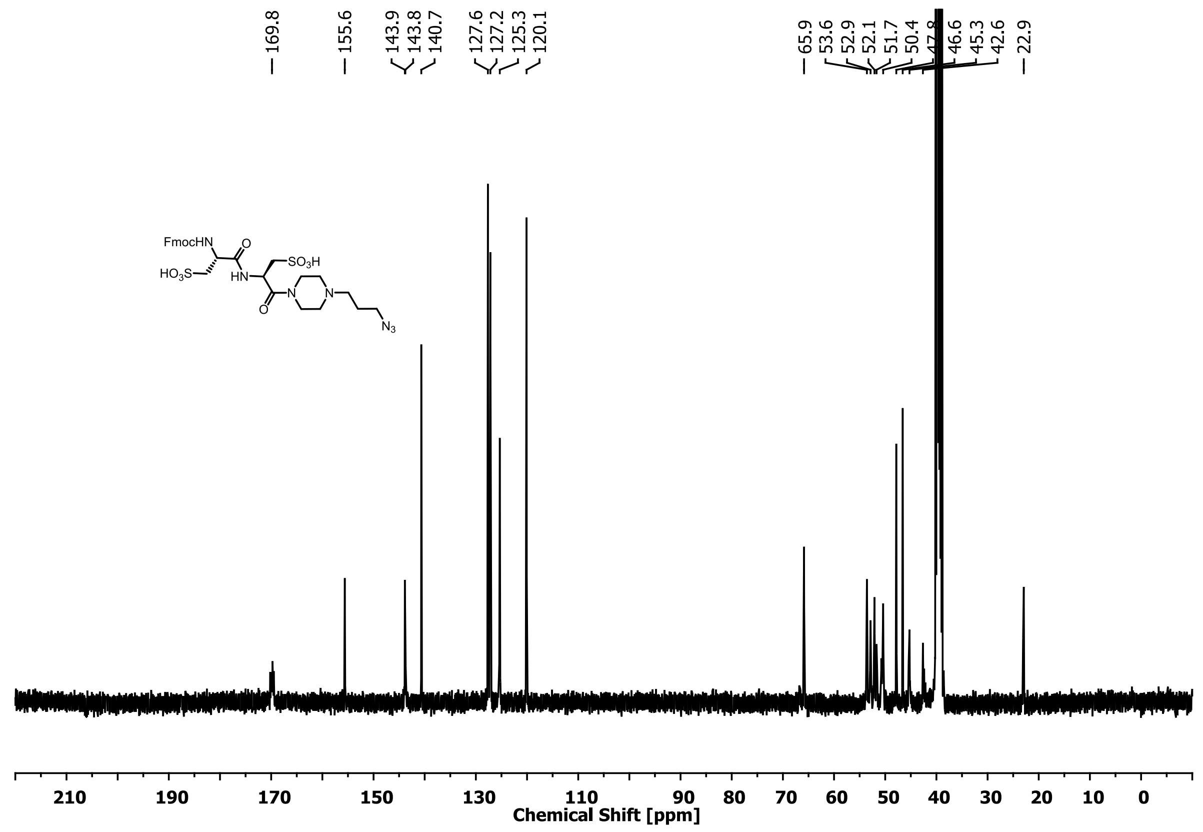

Figure S11: ^{13}C NMR spectrum (DMSO-d_6, 151 MHz, 298 K) of compound 17.

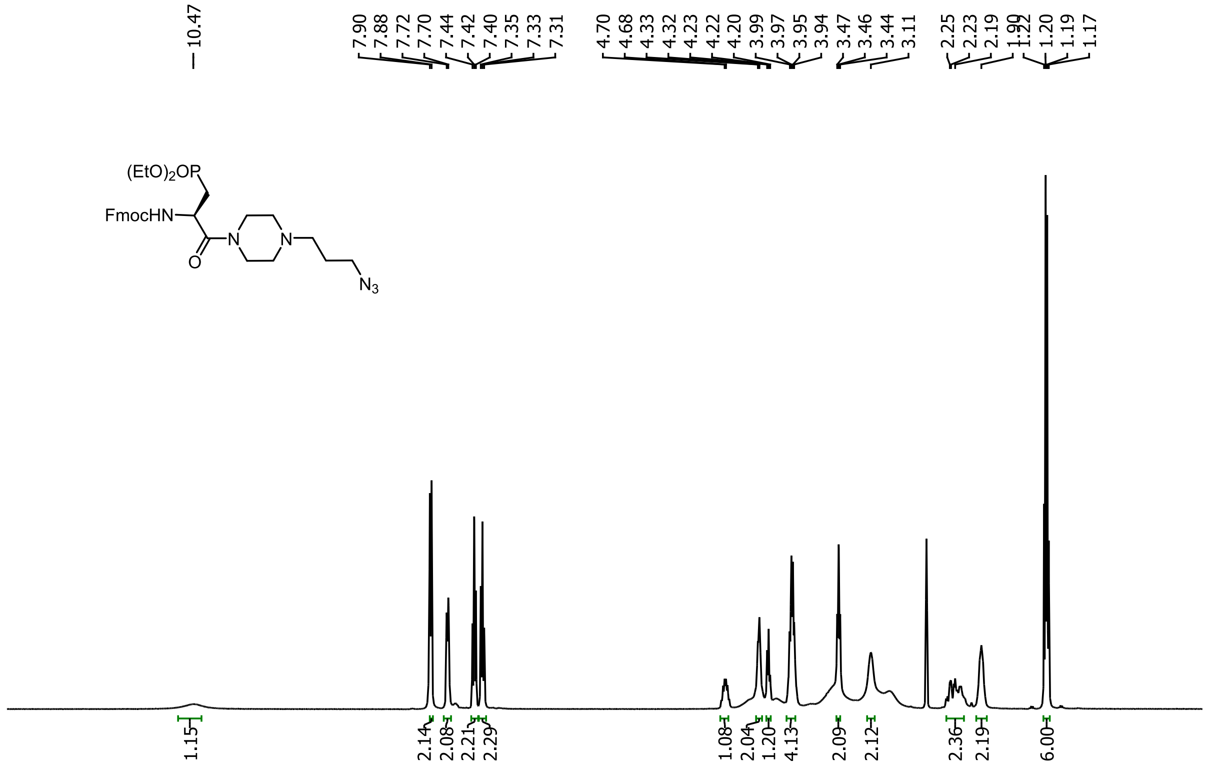

Figure S12: ^{1}H NMR spectrum (DMSO-d_6, 600 MHz, 298 K) of compound 18.

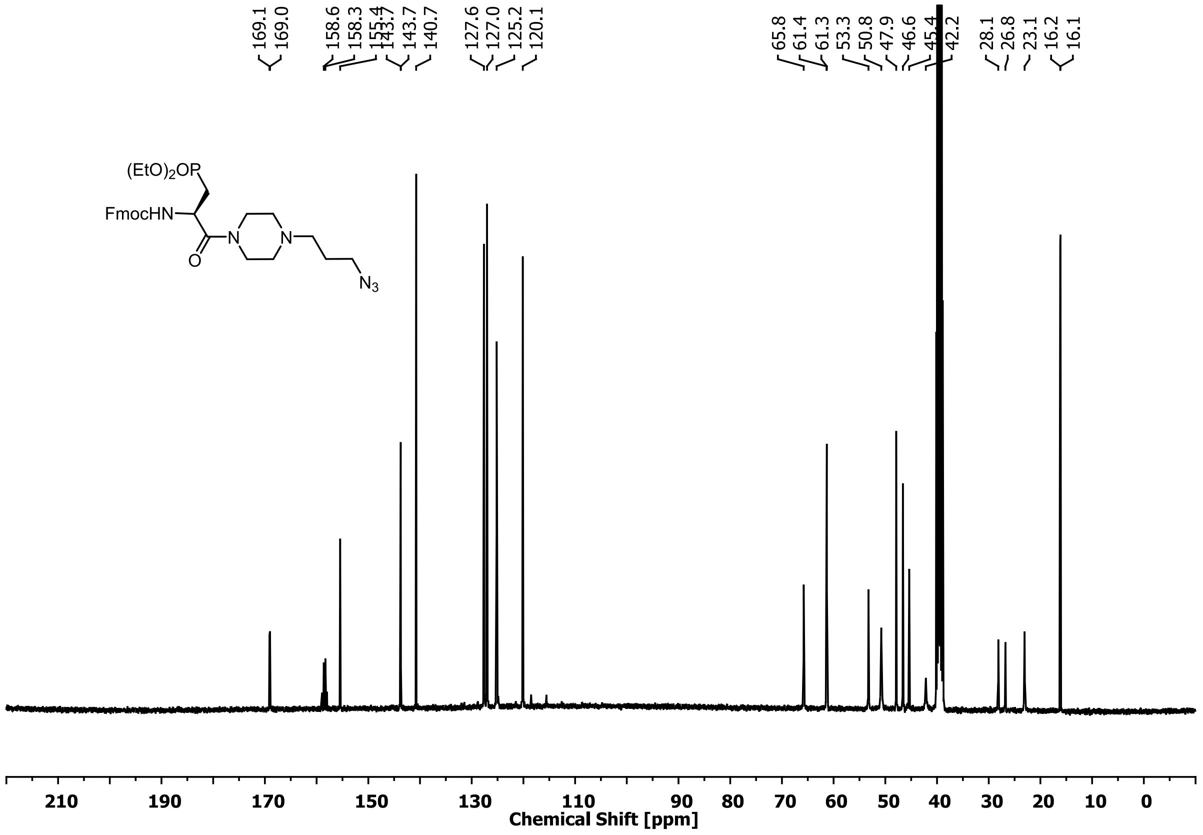

Figure S13: ^{13}C NMR spectrum (DMSO-d_6, 151 MHz, 298 K) of compound 18.

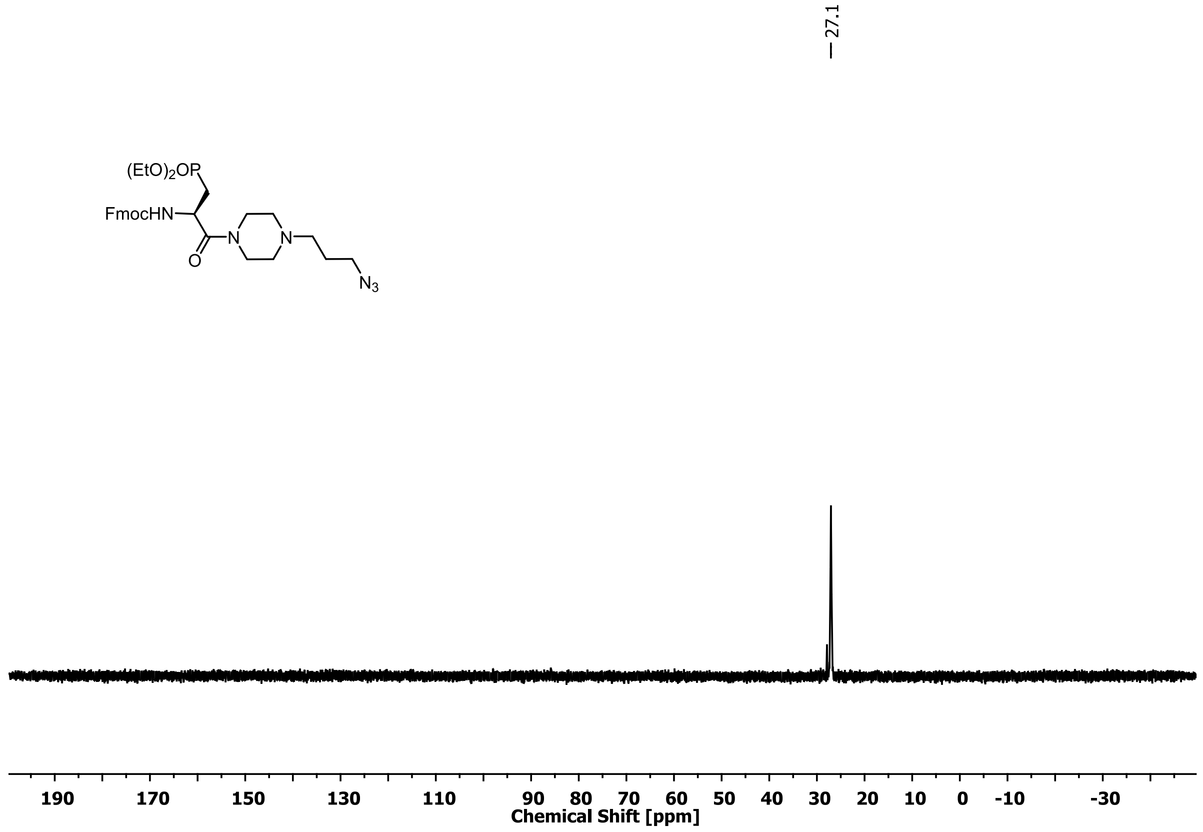

Figure S14: ^{31}P NMR spectrum (DMSO-d_6, 162 MHz, 298 K) of compound 18.

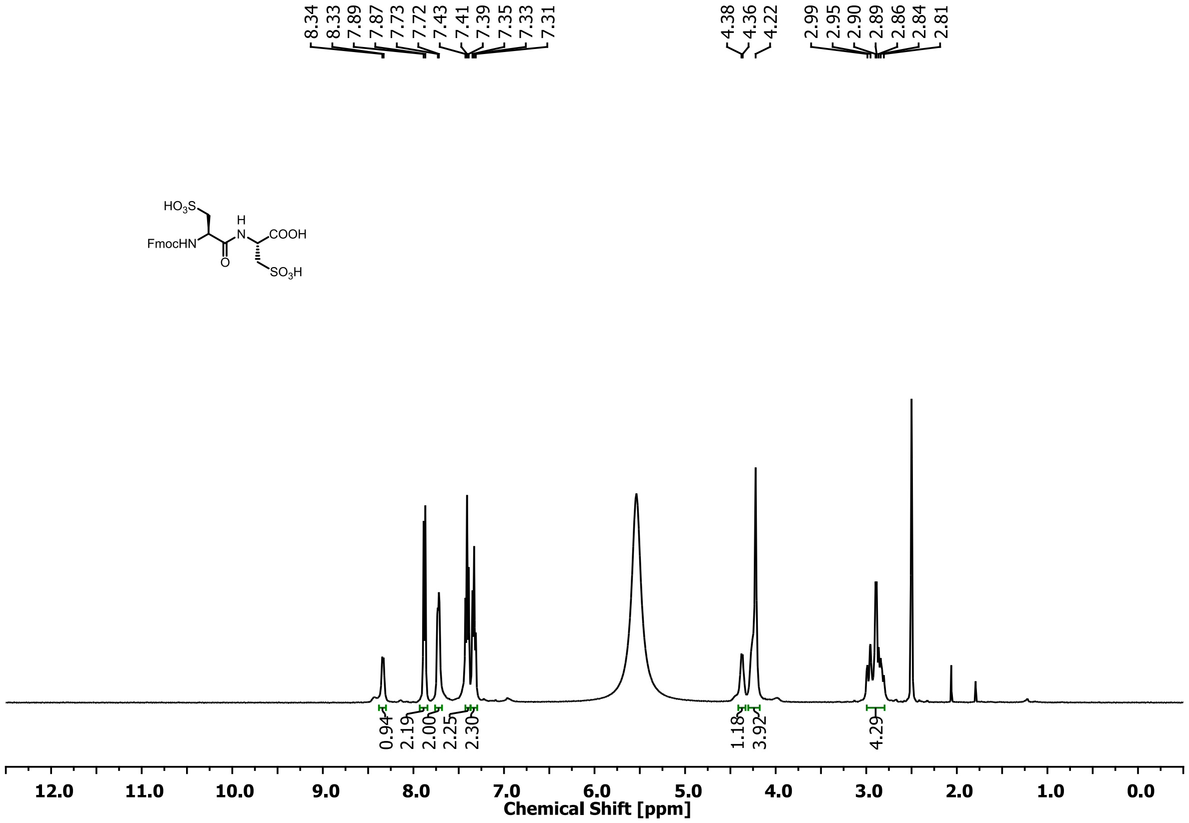

Figure S15: ^{1}H NMR spectrum (DMSO-d_6, 600 MHz, 298 K) of compound 20.

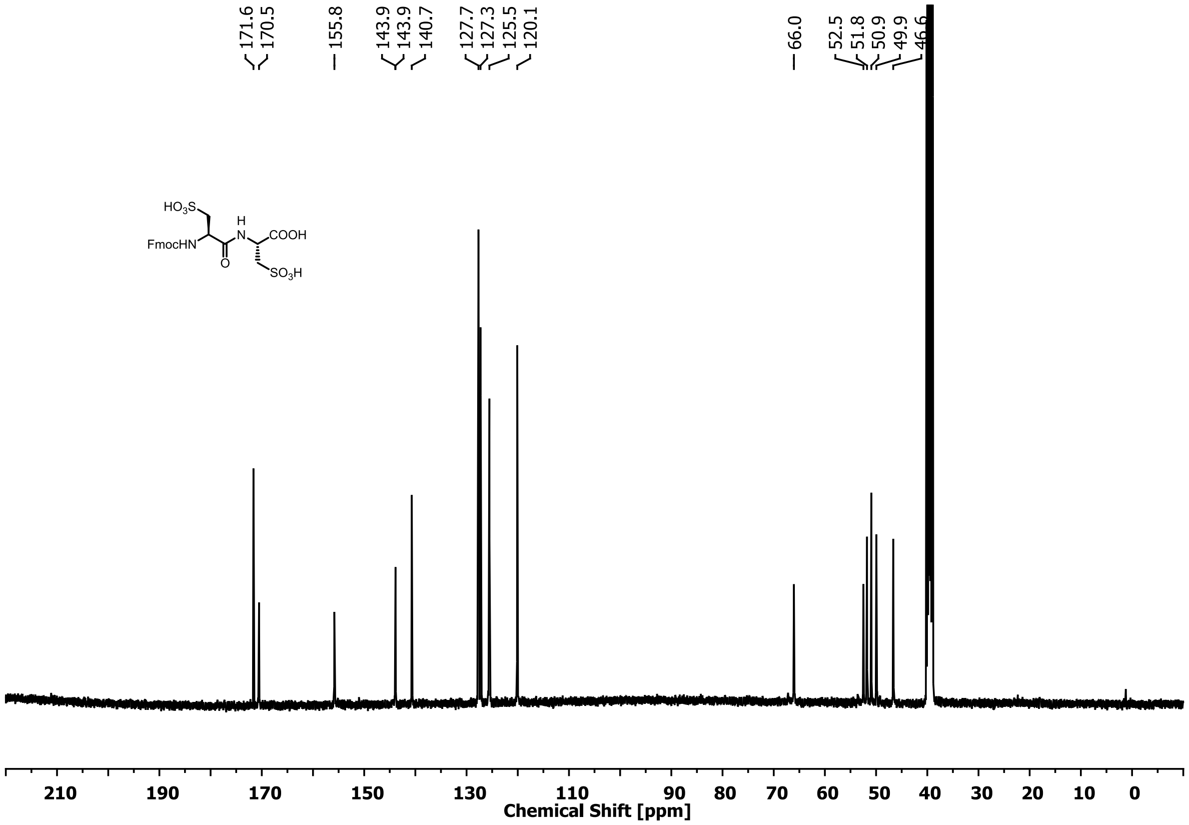

Figure S16: ^{13}C NMR spectrum (DMSO-d_6, 151 MHz, 298 K) of compound 20.

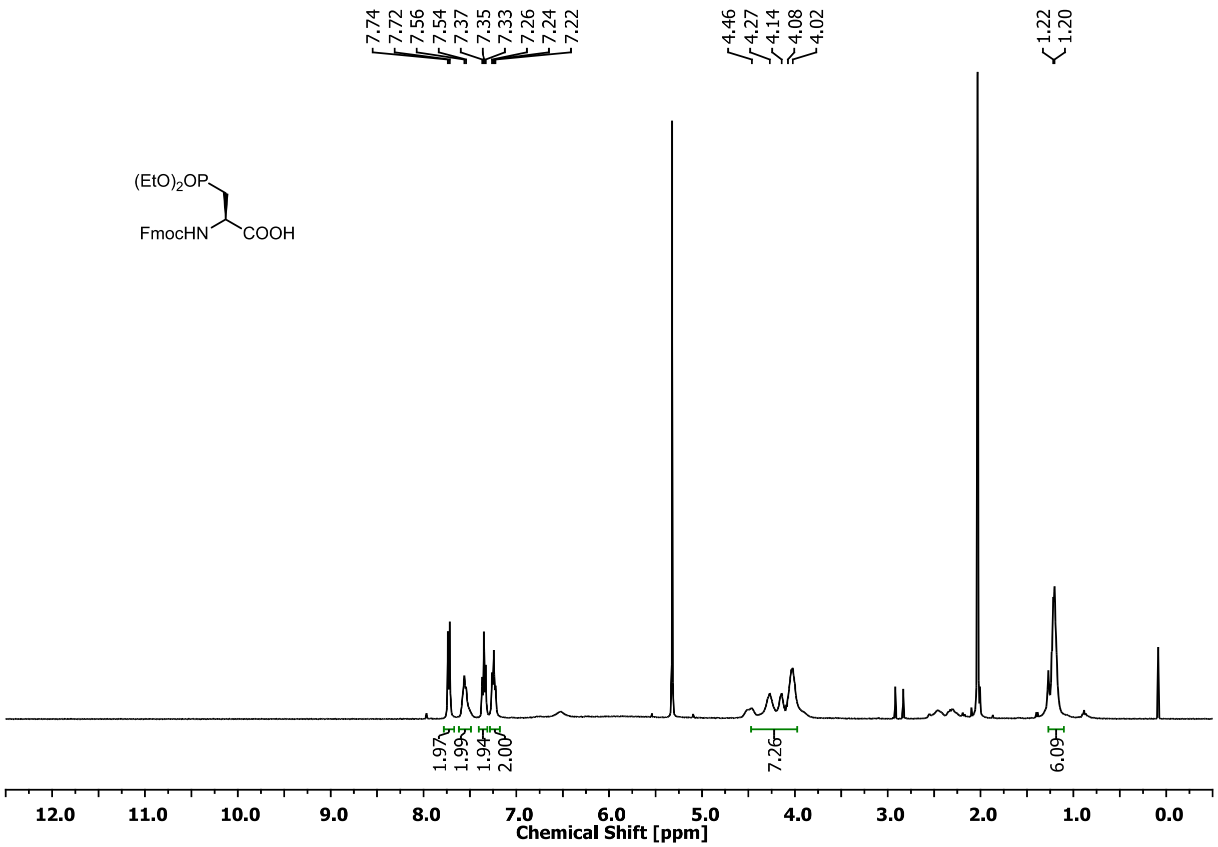

Figure S17: ^{1}H NMR spectrum (DMSO-d_6, 600 MHz, 298 K) of compound 21.

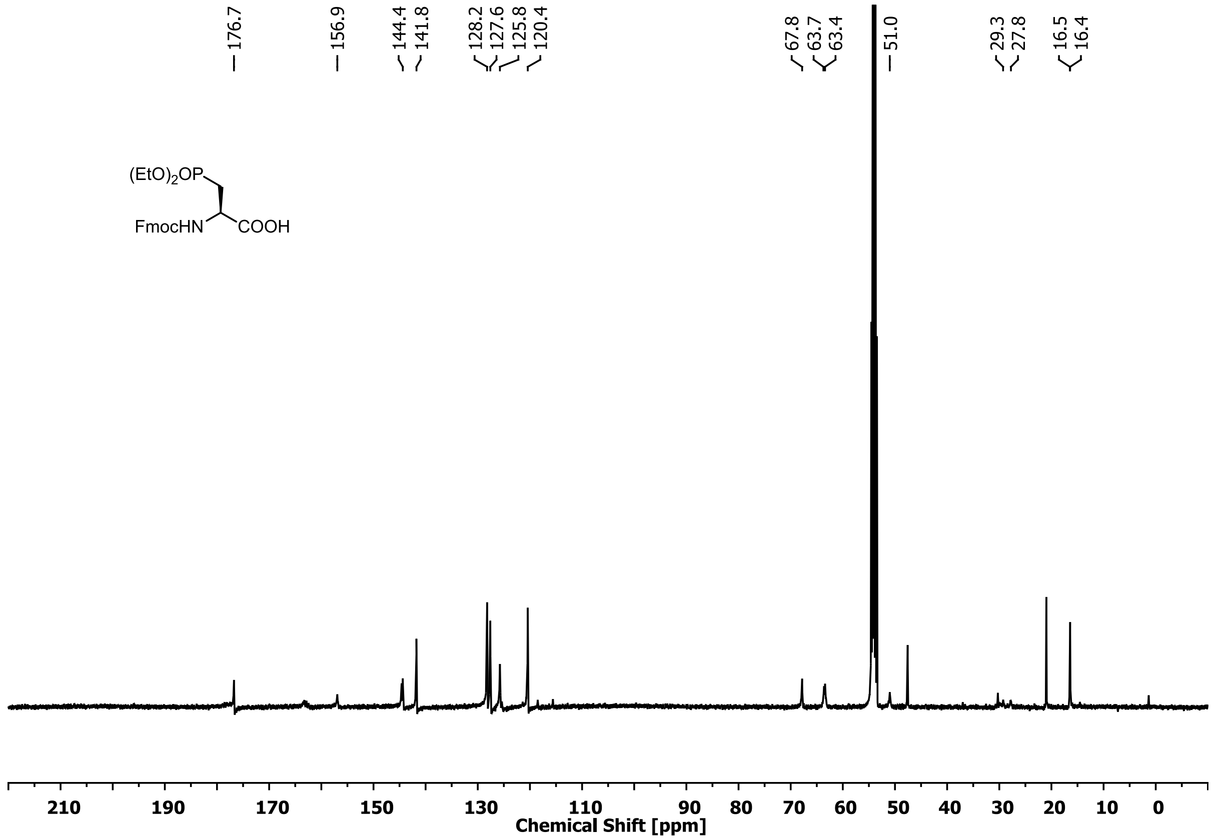

Figure S18: ^{13}C NMR spectrum (DMSO-d_6, 151 MHz, 298 K) of compound 21.

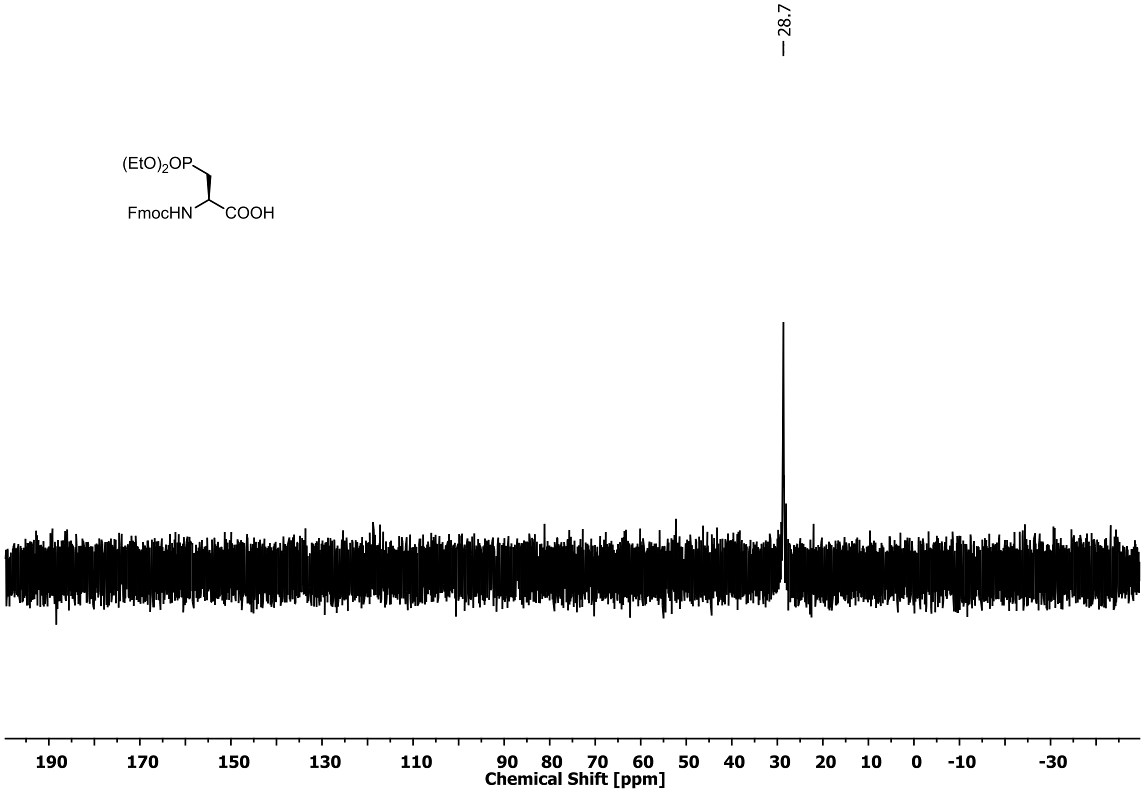

Figure S19: ^{31}P NMR spectrum (DMSO-d_6, 162 MHz, 298 K) of compound 21.

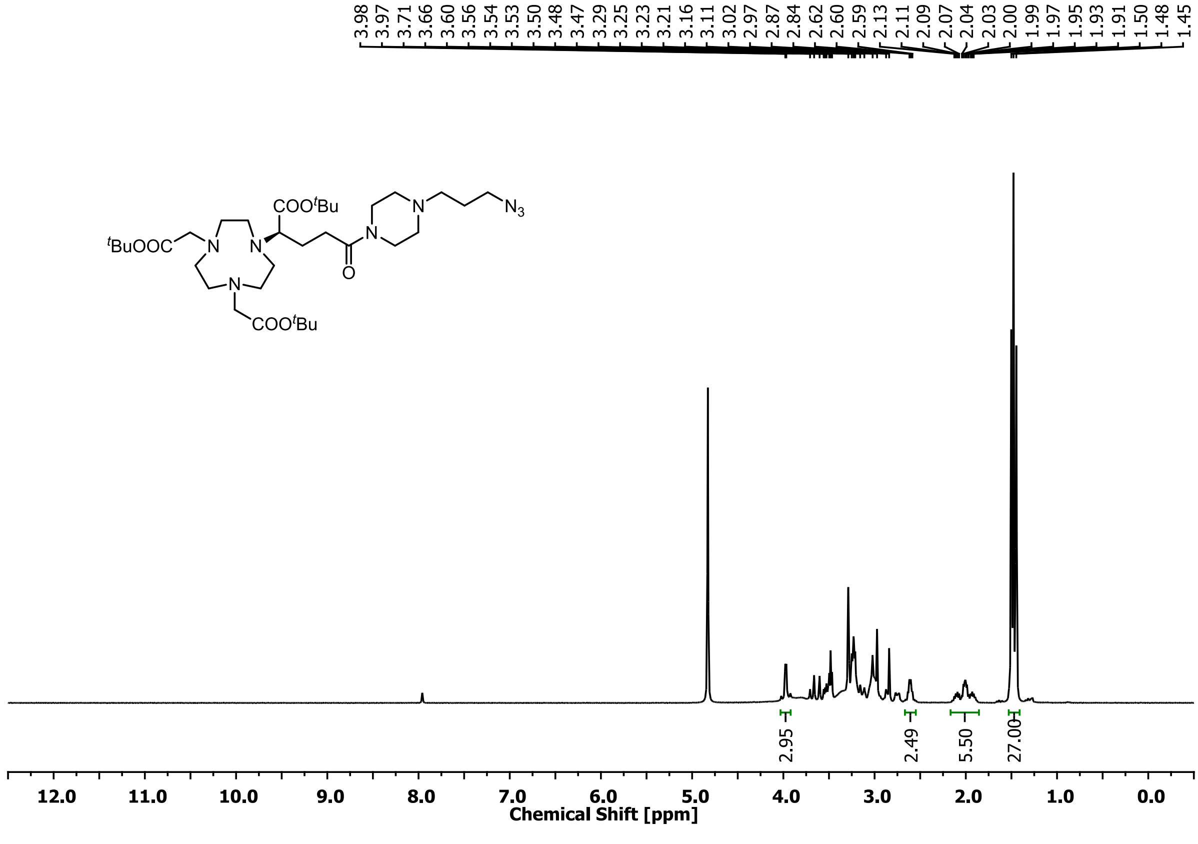

Figure S20: ^{1}H NMR spectrum (DMSO-d_6, 600 MHz, 298 K) of compound 23.

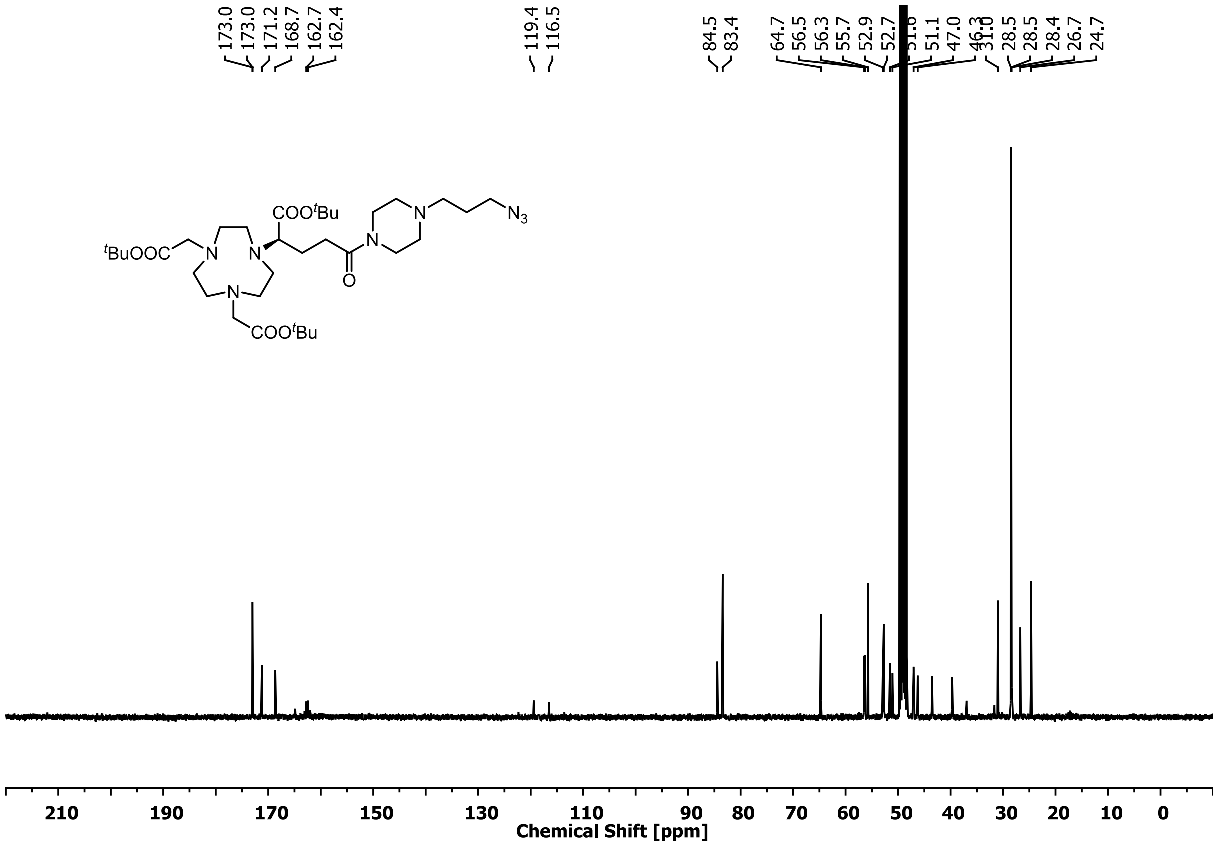

Figure S21: ^{13}C NMR spectrum (DMSO-d_6, 151 MHz, 298 K) of compound 23.

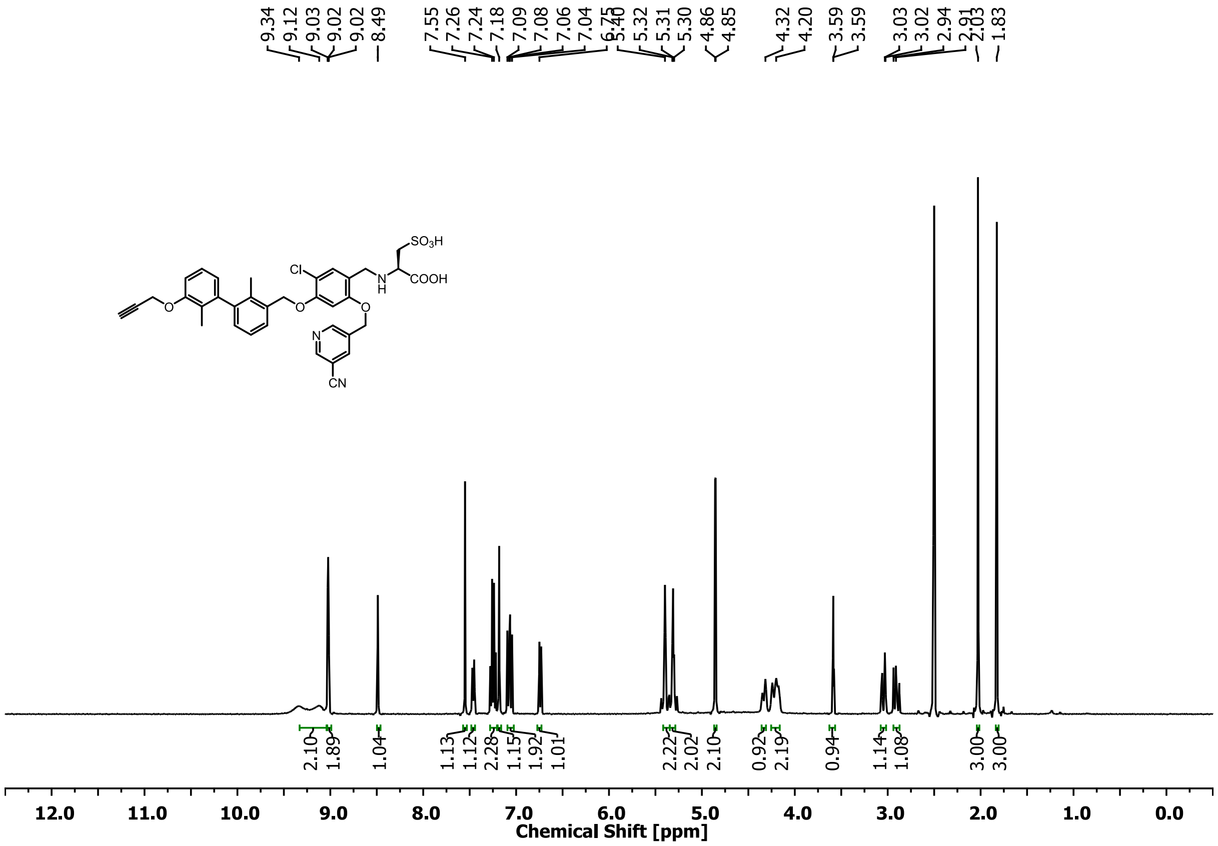

Figure S22: ^{1}H NMR spectrum (DMSO-d_6, 600 MHz, 298 K) of compound 37.

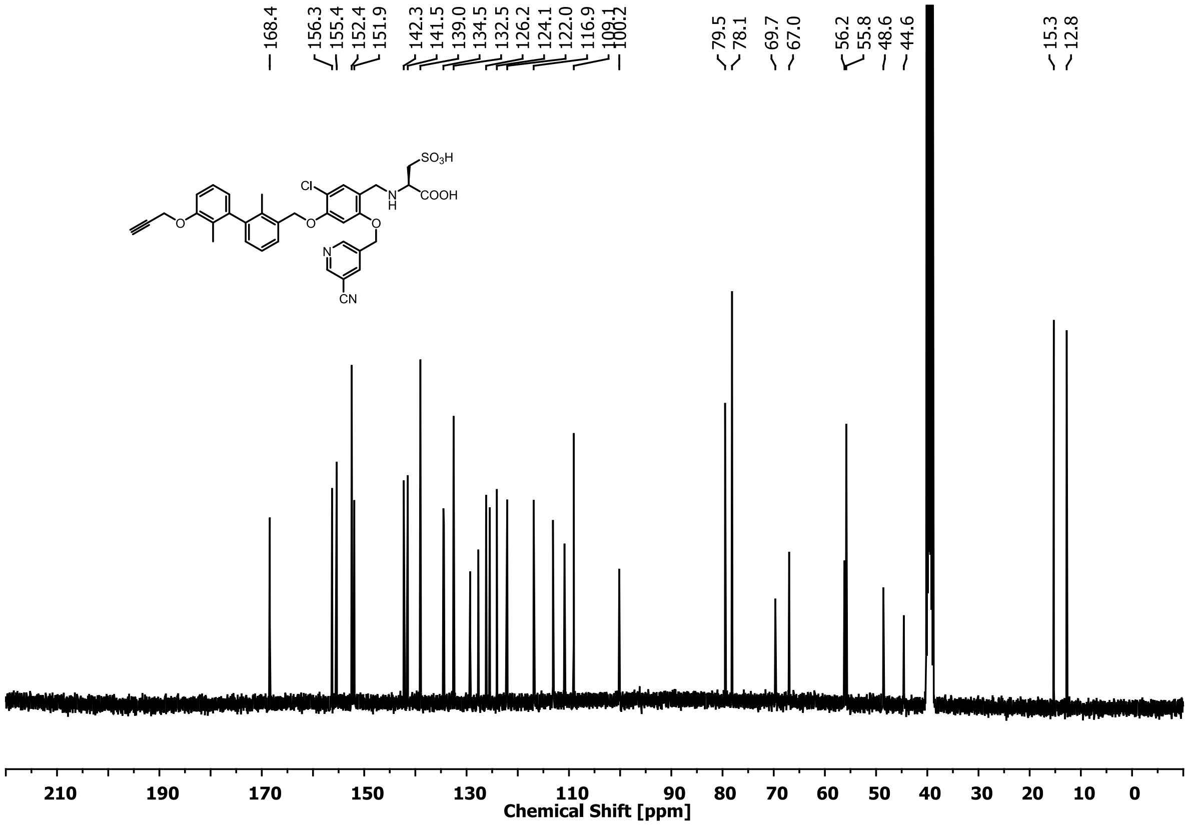

Figure S23: ^{13}**C NMR spectrum (DMSO-*d*$_6$, 151 MHz, 298 K) of compound 37.**

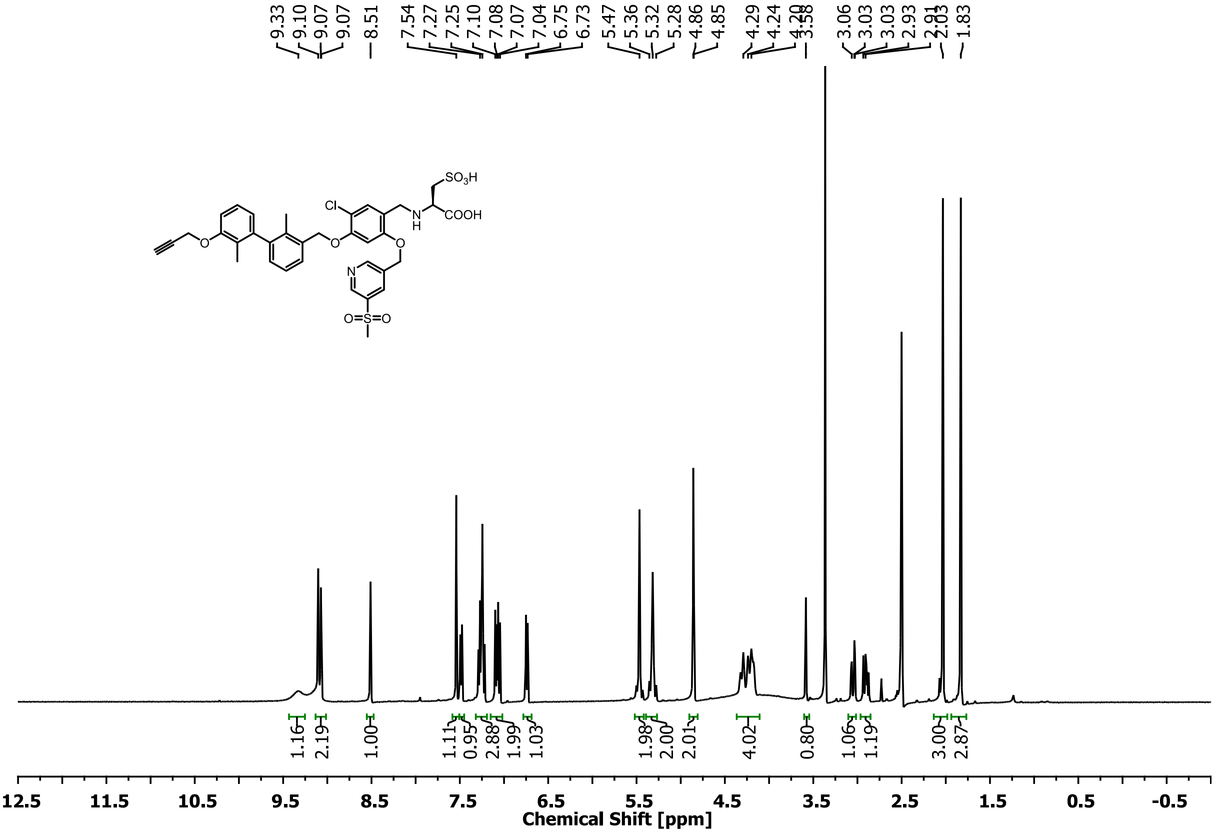

Figure S24: ^{1}**H NMR spectrum (DMSO-*d*$_6$, 600 MHz, 298 K) of compound 38.**

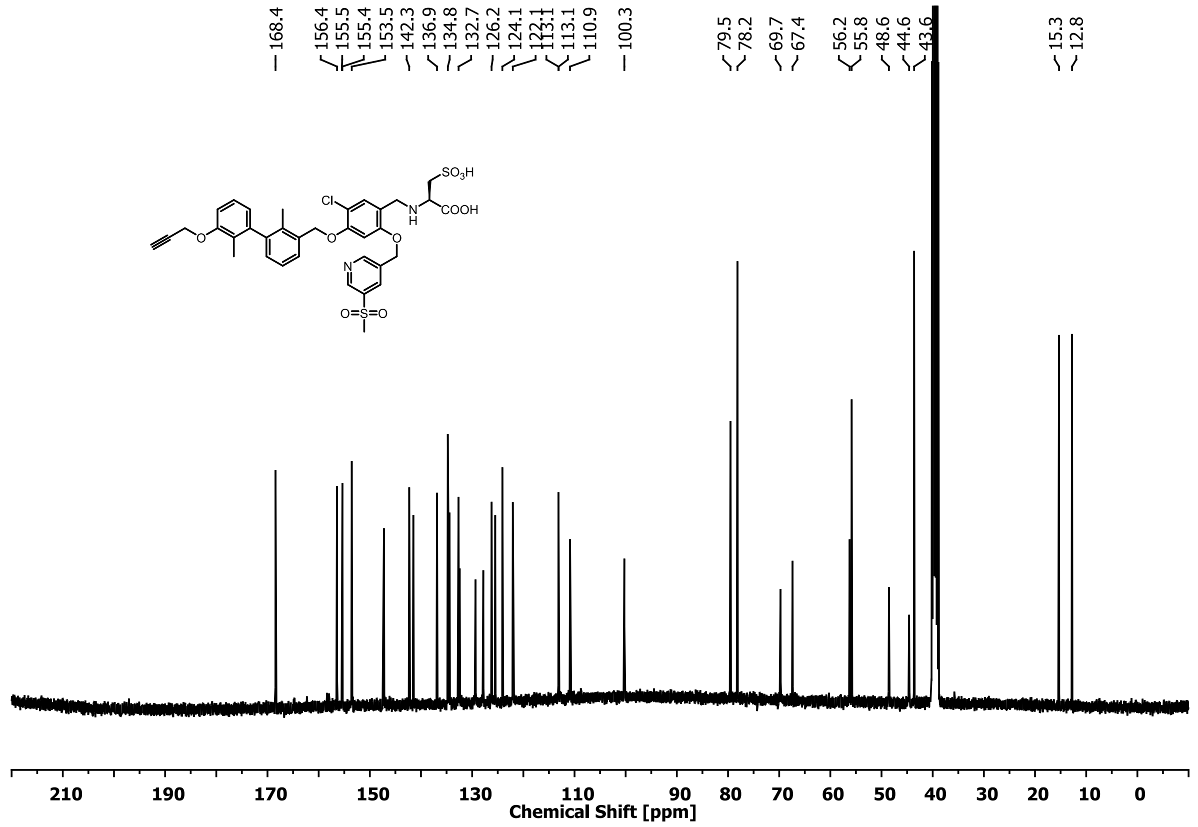

Figure S25: ^{13}C NMR spectrum (DMSO-d_6, 151 MHz, 298 K) of compound 38.

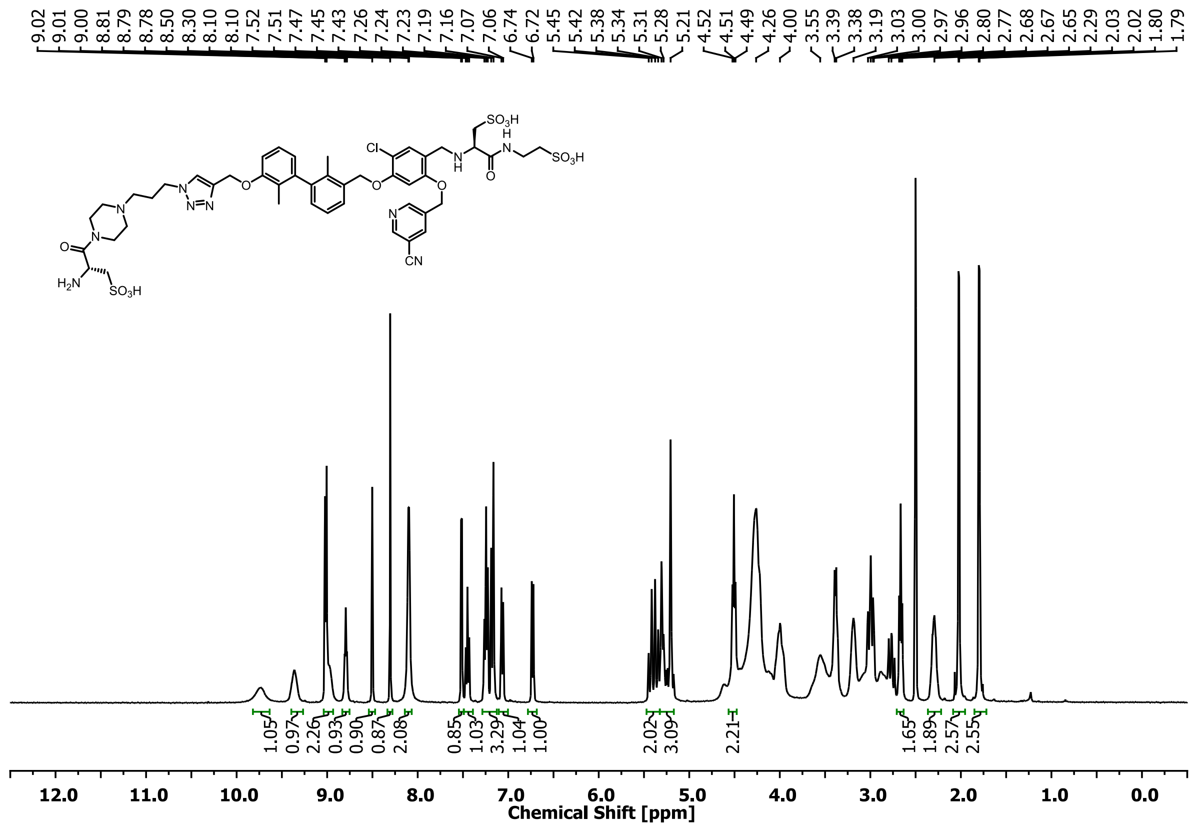

Figure S26: ^{1}H NMR spectrum (DMSO-d_6, 600 MHz, 298 K) of compound 44.

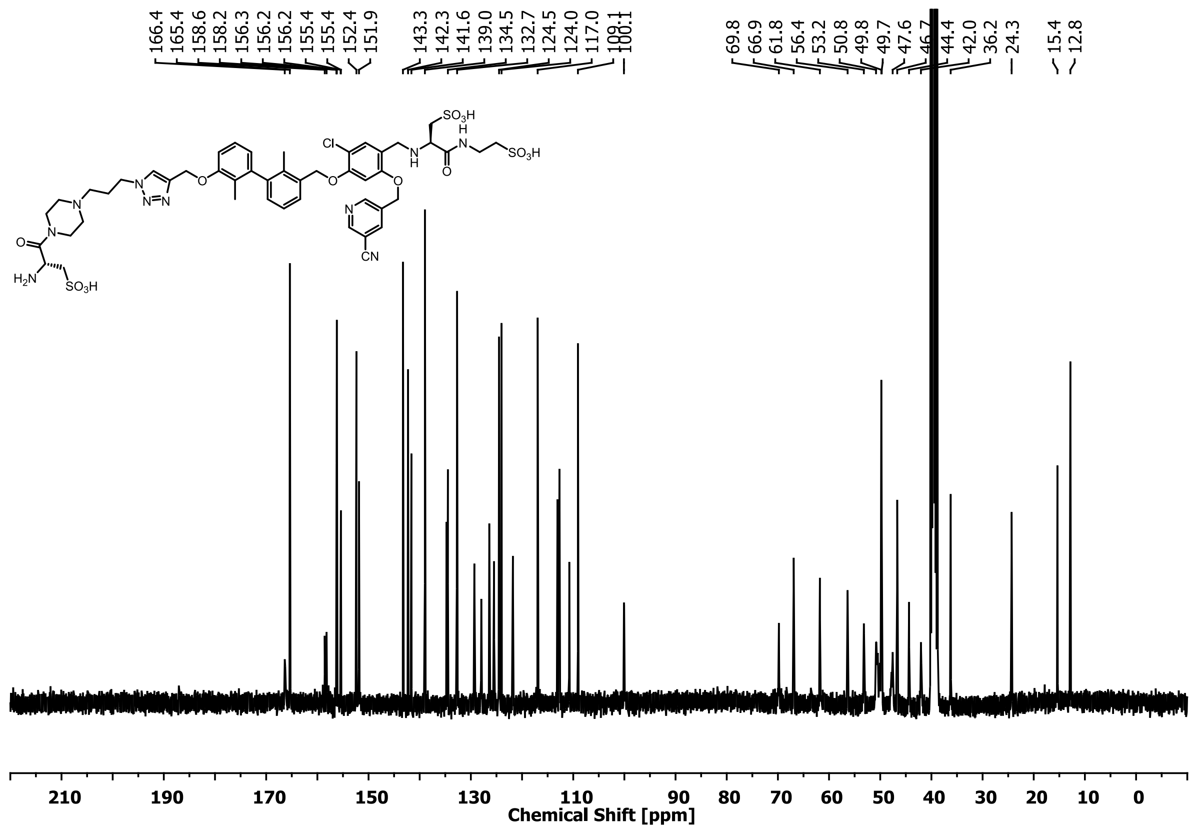

Figure S27: ^{13}C NMR spectrum (DMSO-d_6, 151 MHz, 298 K) of compound 44.

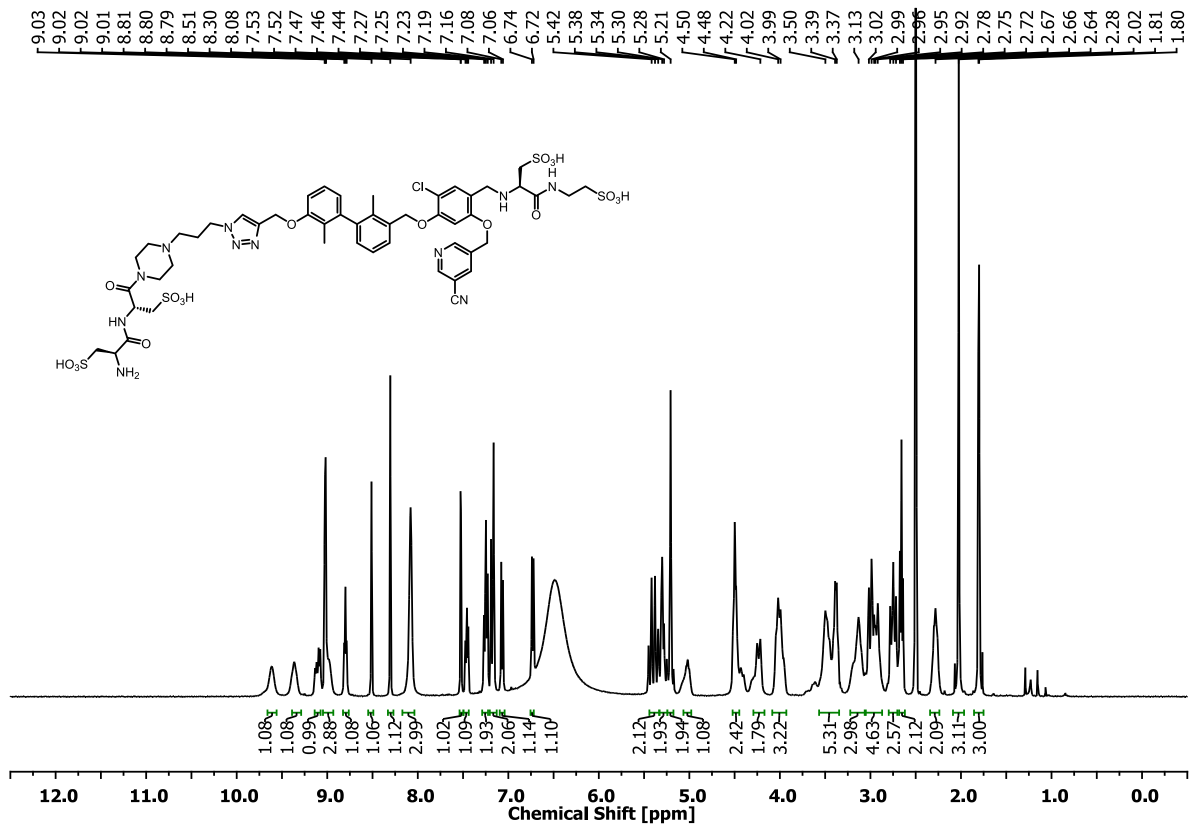

Figure S28: ^{1}H NMR spectrum (DMSO-d_6, 600 MHz, 298 K) of compound 45.

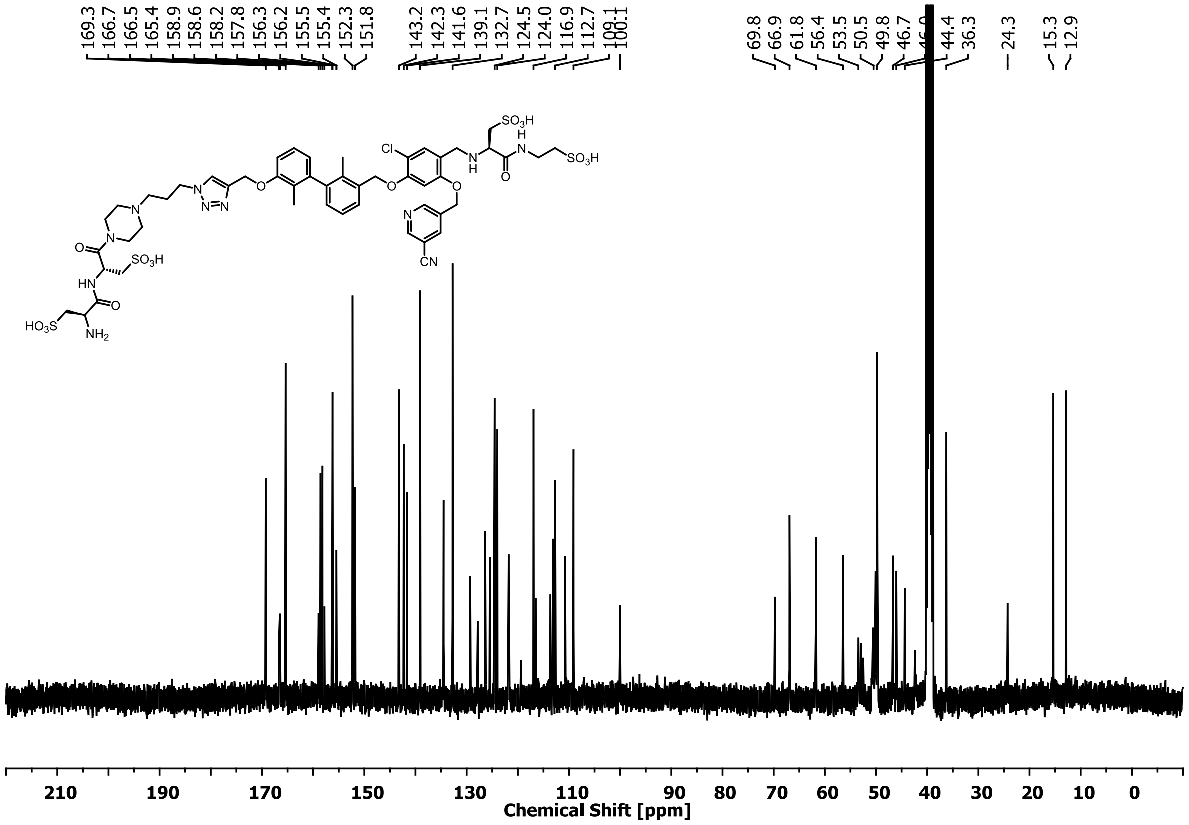

Figure S29: ^{13}C NMR spectrum (DMSO-d_6, 151 MHz, 298 K) of compound 45.

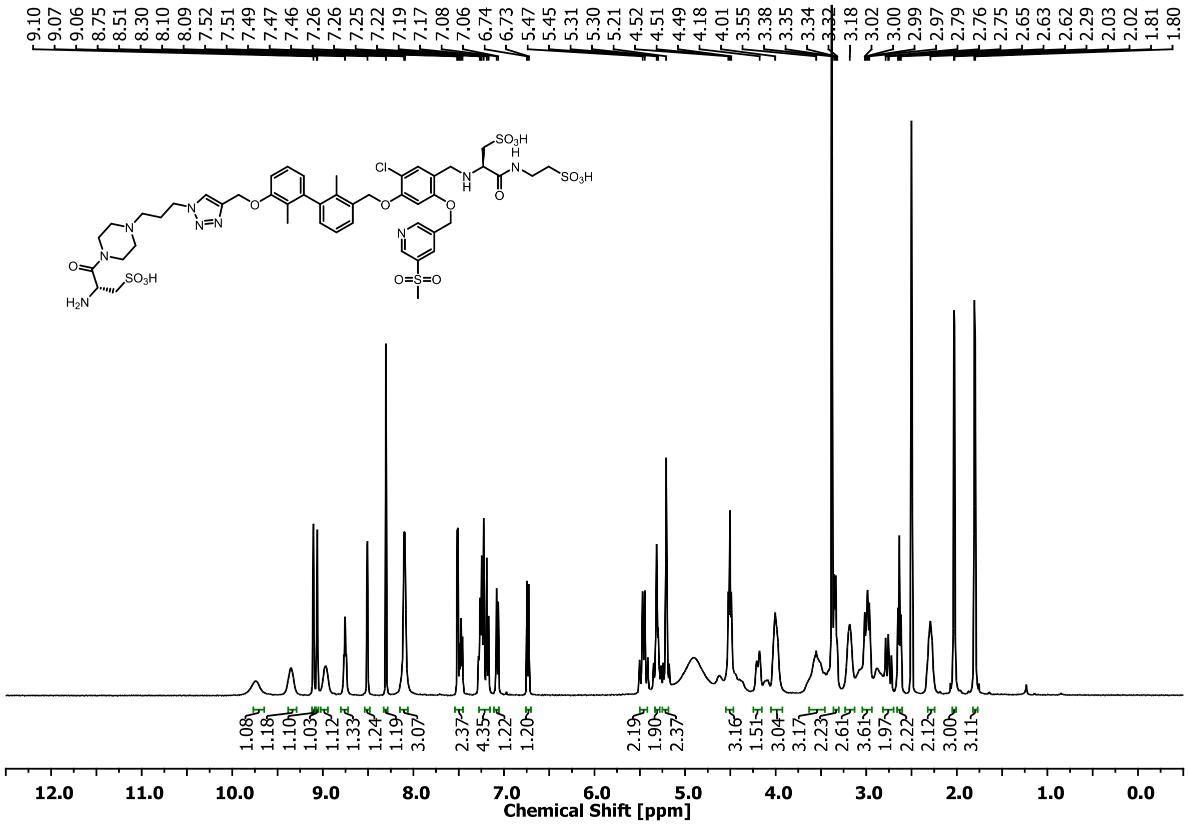

Figure S30: ^{1}H NMR spectrum (DMSO-d_6, 600 MHz, 298 K) of compound 46.

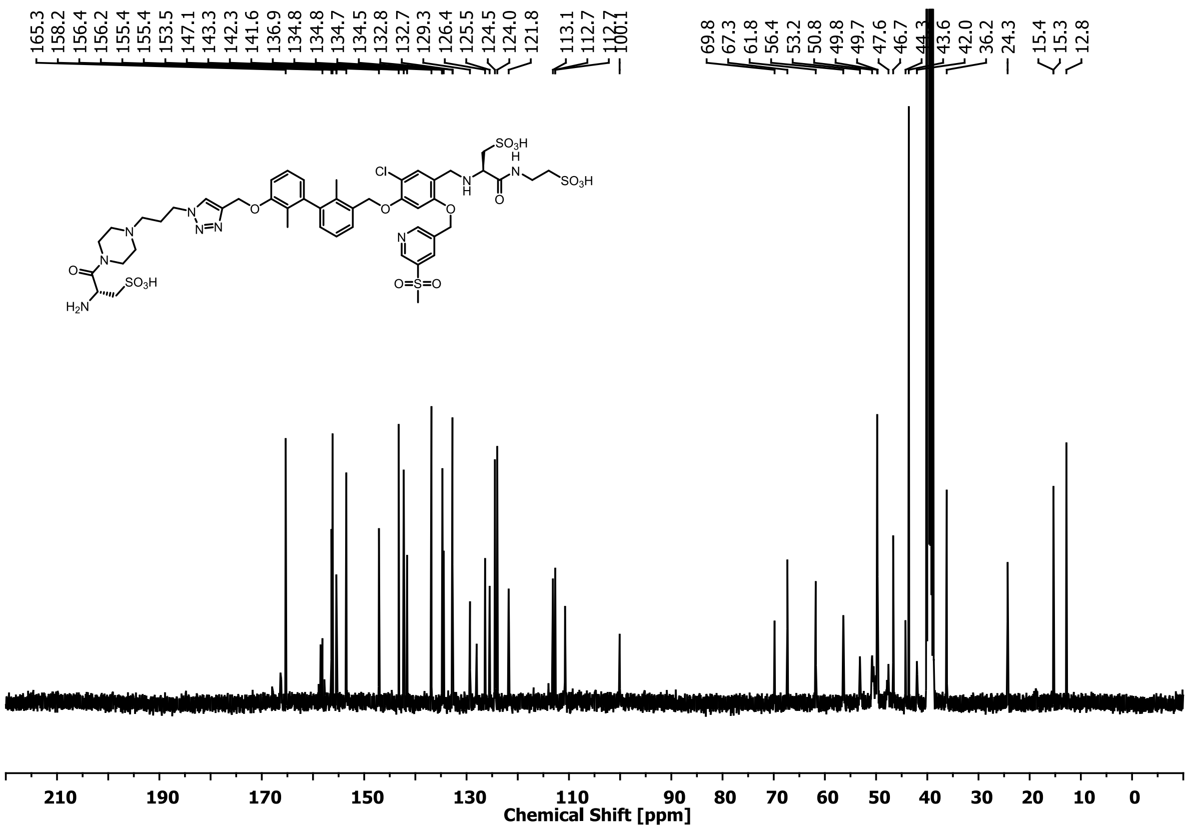

Figure S31: ^{13}C NMR spectrum (DMSO-d_6, 151 MHz, 298 K) of compound 46.

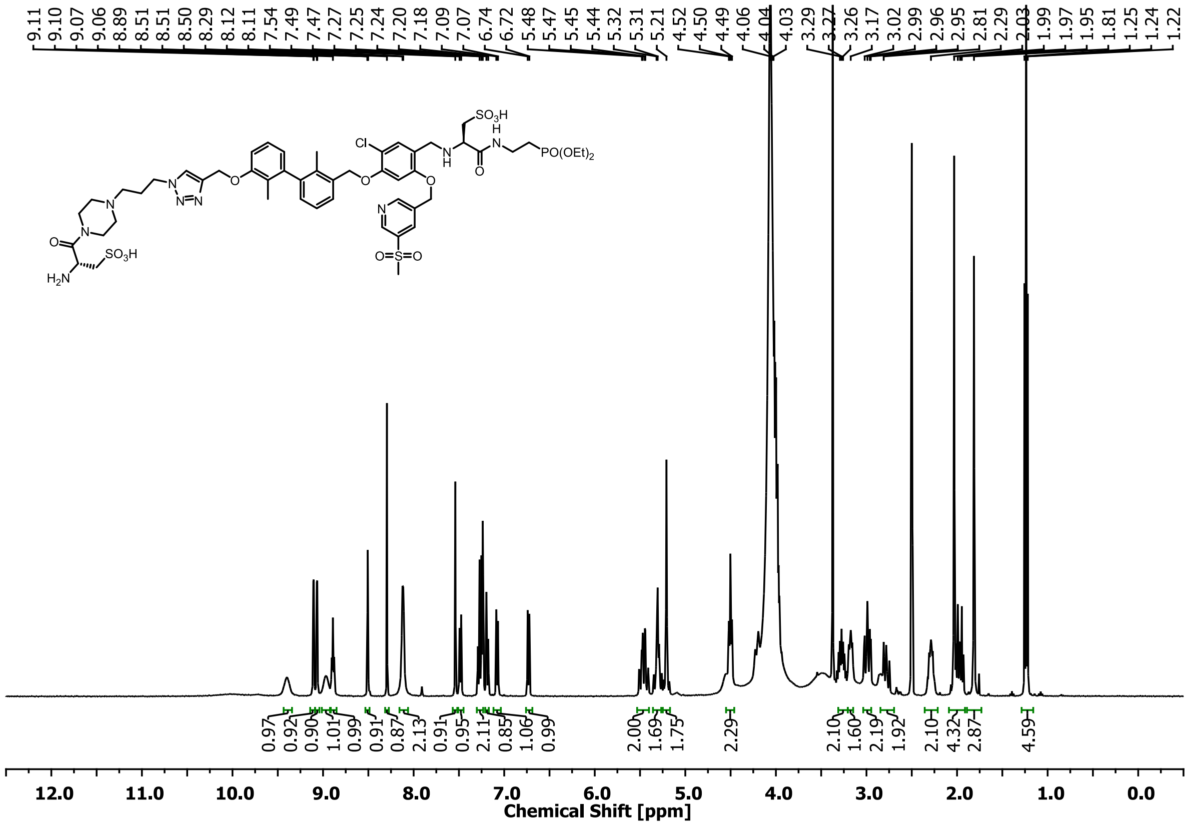

Figure S32: ^{1}H NMR spectrum (DMSO-d_6, 600 MHz, 298 K) of compound 47.

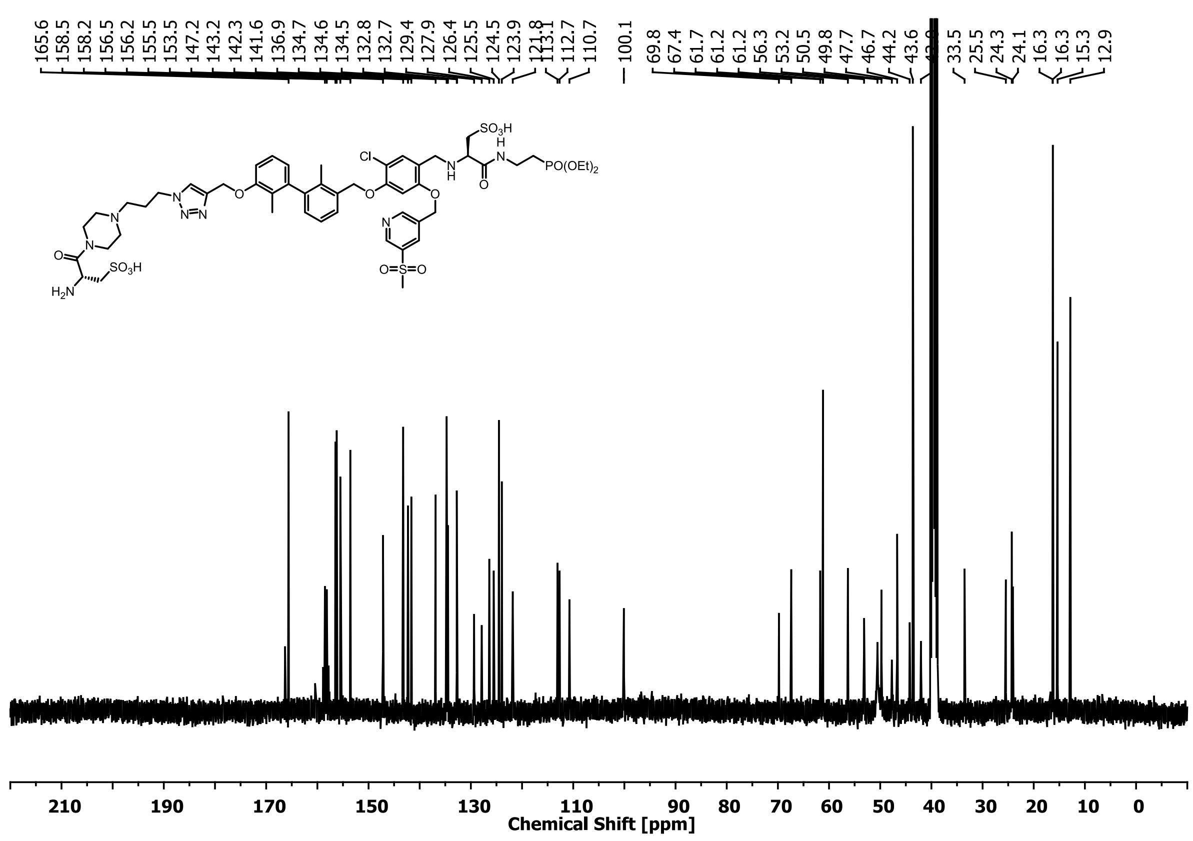

Figure S33: ^{13}C NMR spectrum (DMSO-d_6, 151 MHz, 298 K) of compound 47.

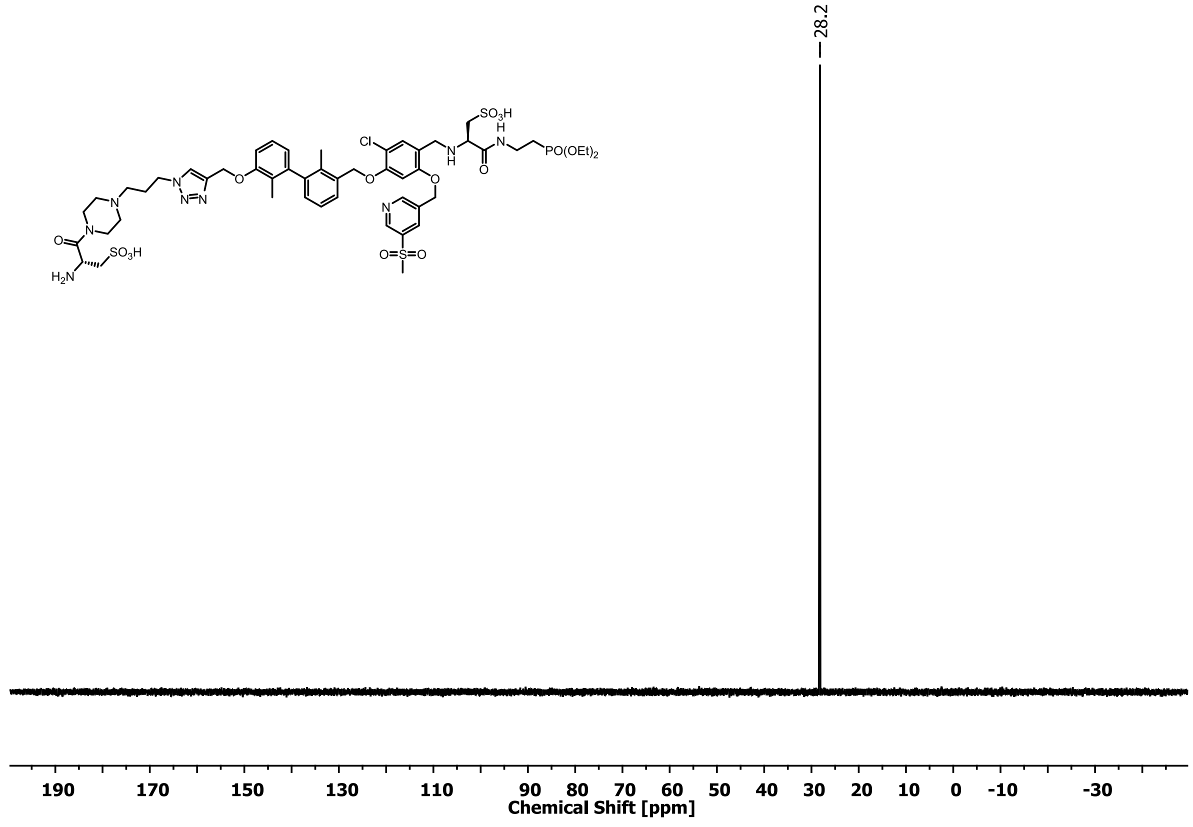

Figure S34: ^{31}P NMR spectrum (DMSO-d_6, 162 MHz, 298 K) of compound 47.

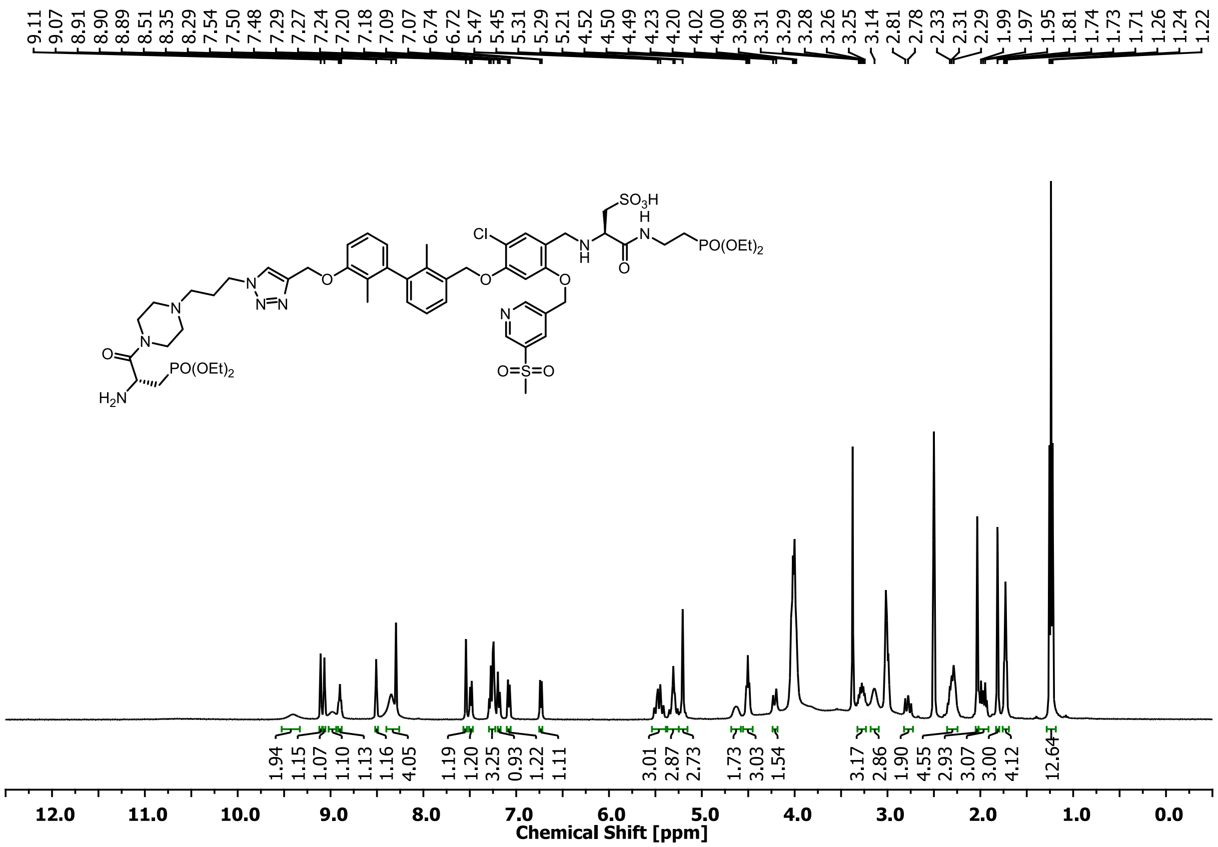

Figure S35: ^{1}H NMR spectrum (DMSO-d_6, 600 MHz, 298 K) of compound 48.

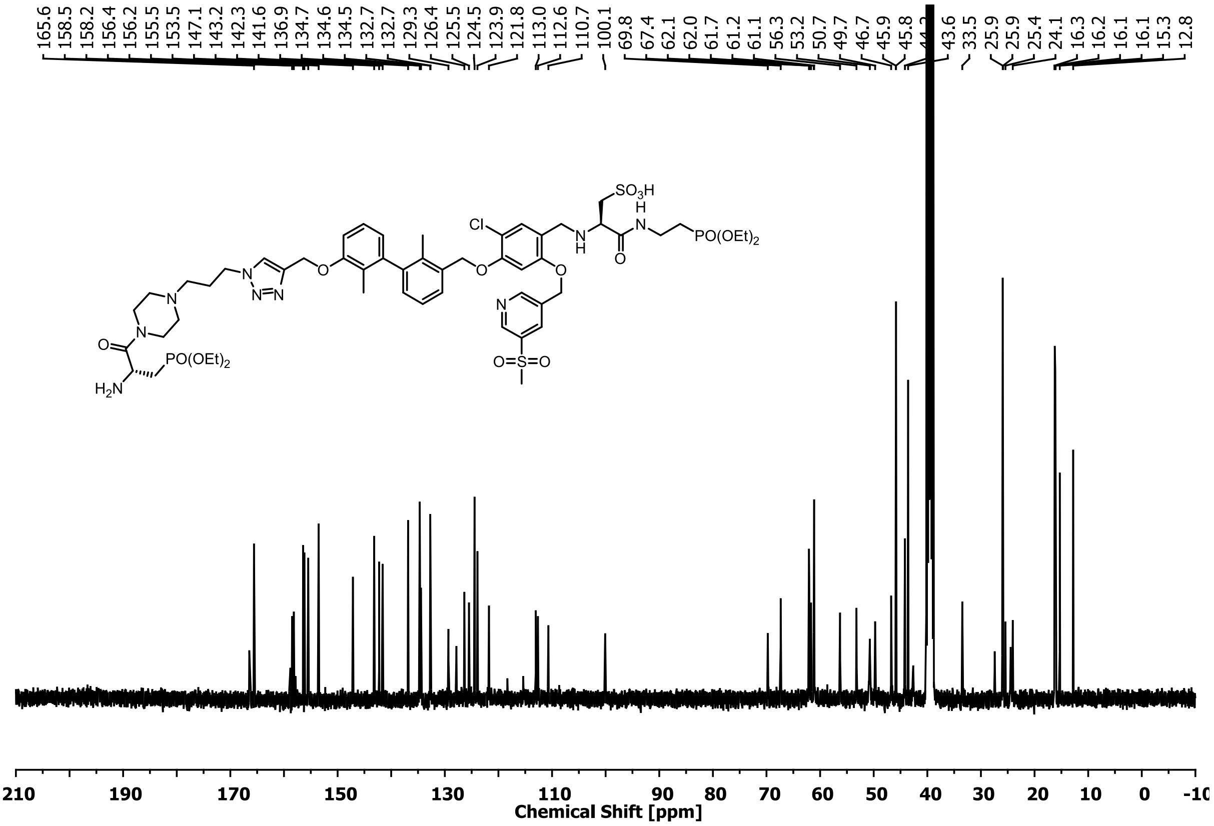

Figure S36: ^{13}C NMR spectrum (DMSO-d_6, 151 MHz, 298 K) of compound 48.

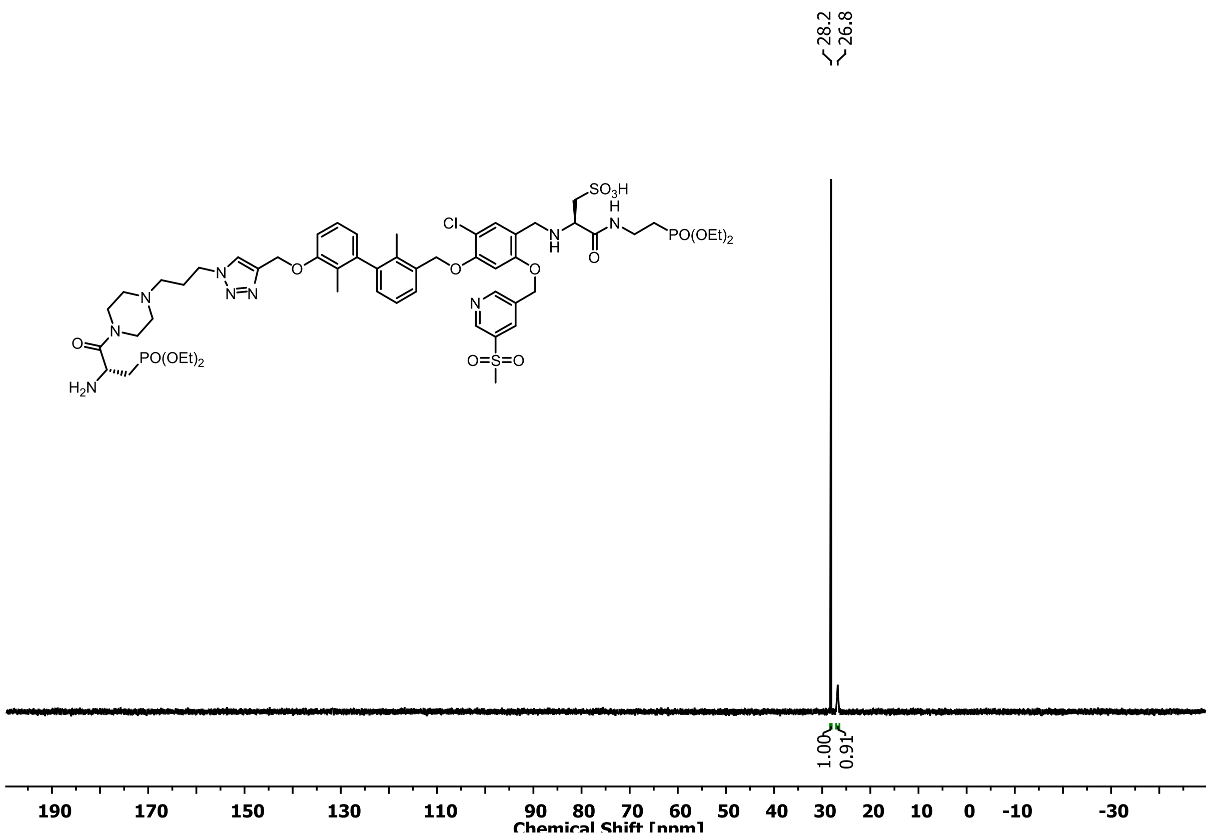

Figure S37: ^{31}P NMR spectrum (DMSO-d_6, 162 MHz, 298 K) of compound 48.

3. IR spectra of literature unknown compounds

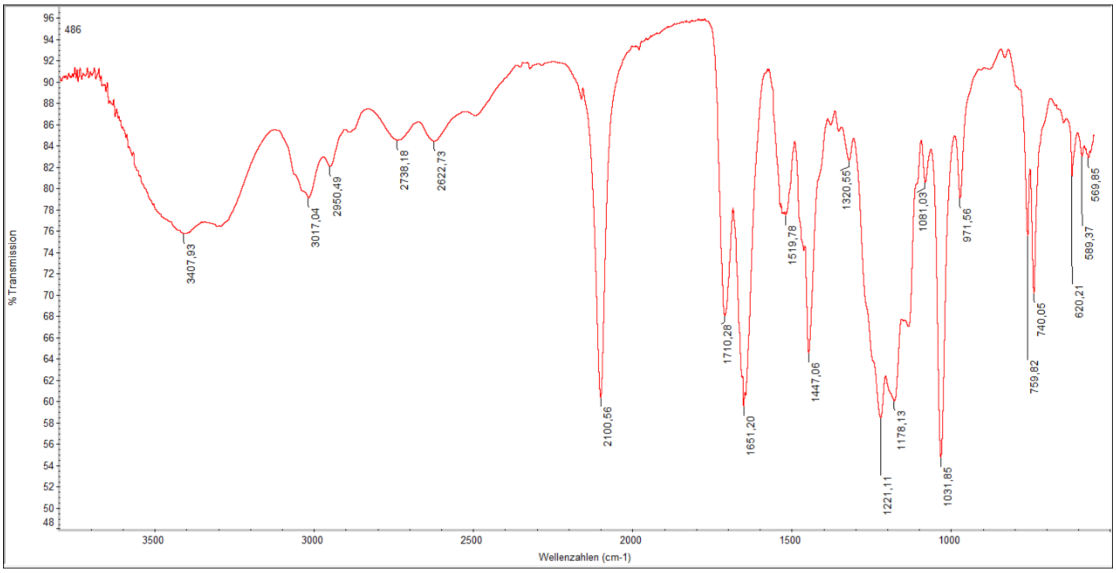

Figure S38: ATR-IR spectrum of compound 16.

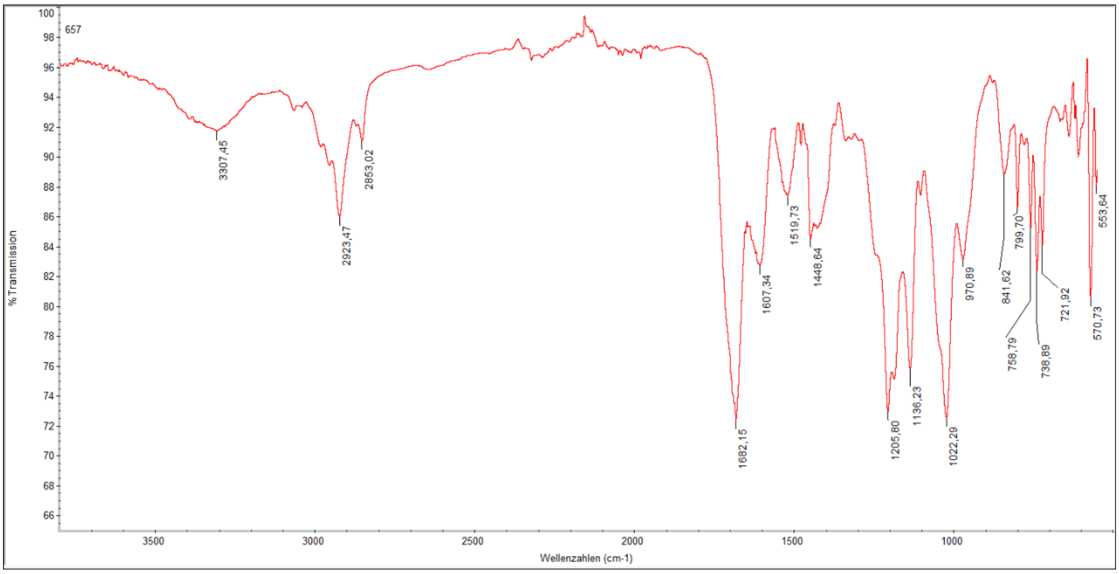

Figure S39: ATR-IR spectrum of compound 21.

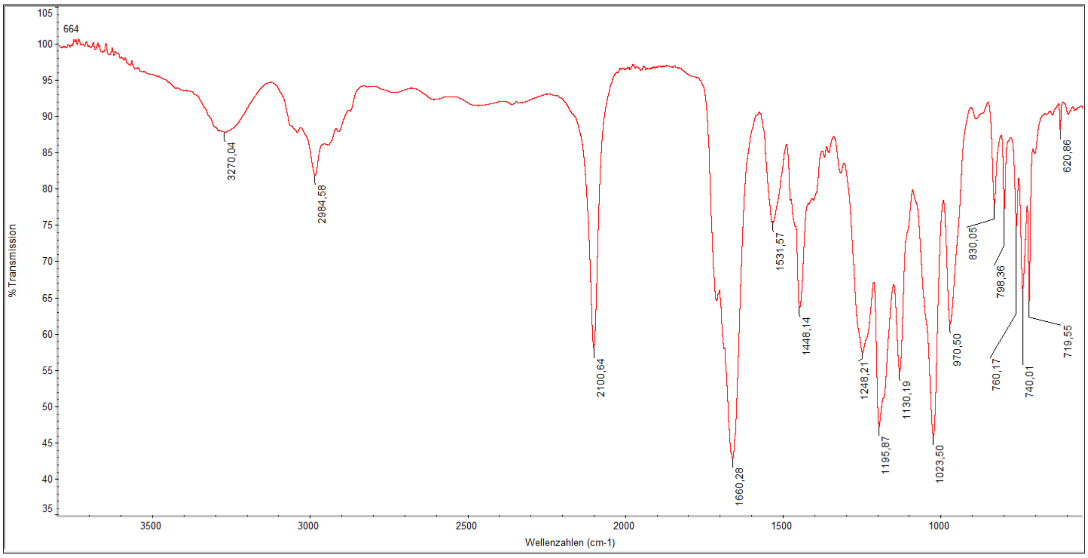

Figure S40: ATR-IR spectrum of compound 18.

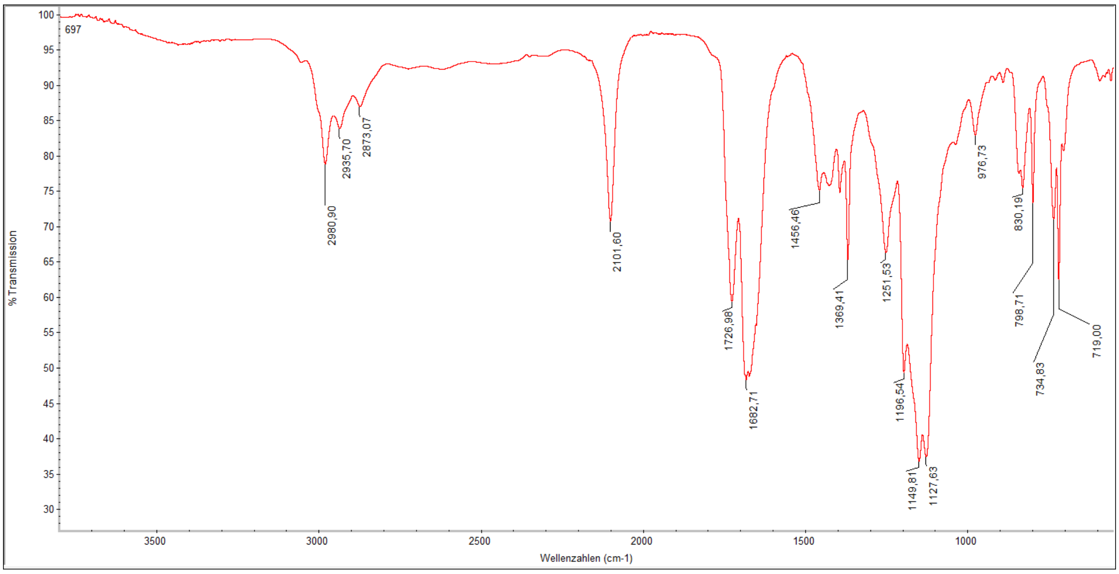

Figure S41: ATR-IR spectrum of compound 23.

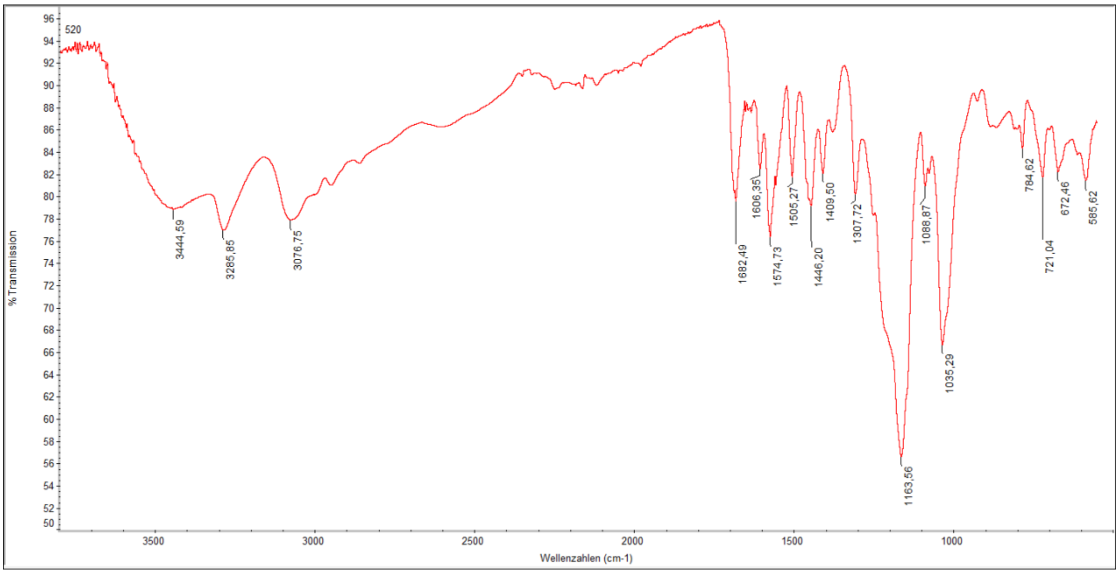

Figure S42: ATR-IR spectrum of compound 13.

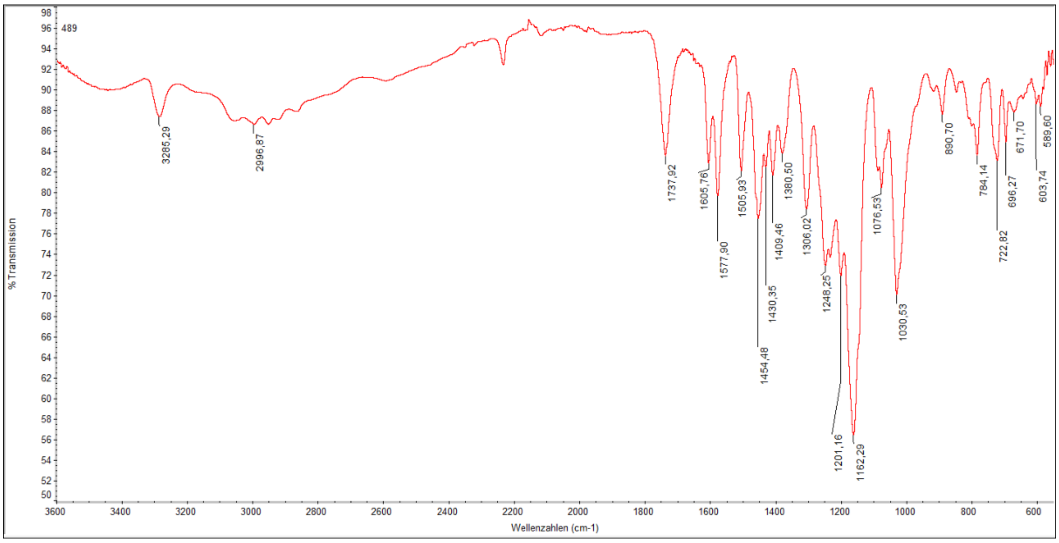

Figure S43: ATR-IR spectrum of compound 37.

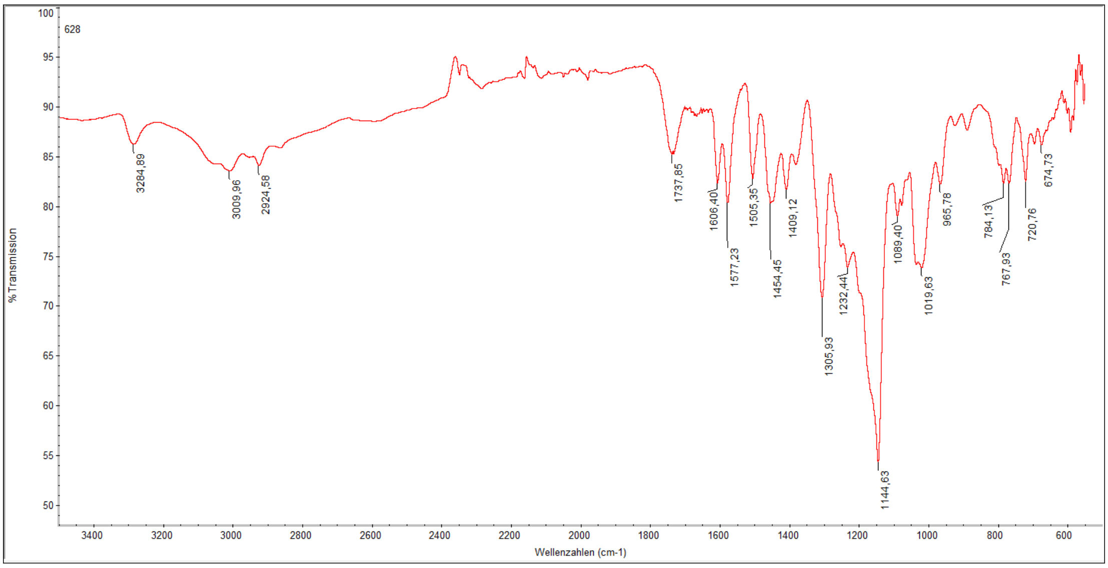

Figure S44: ATR-IR spectrum of compound 38.

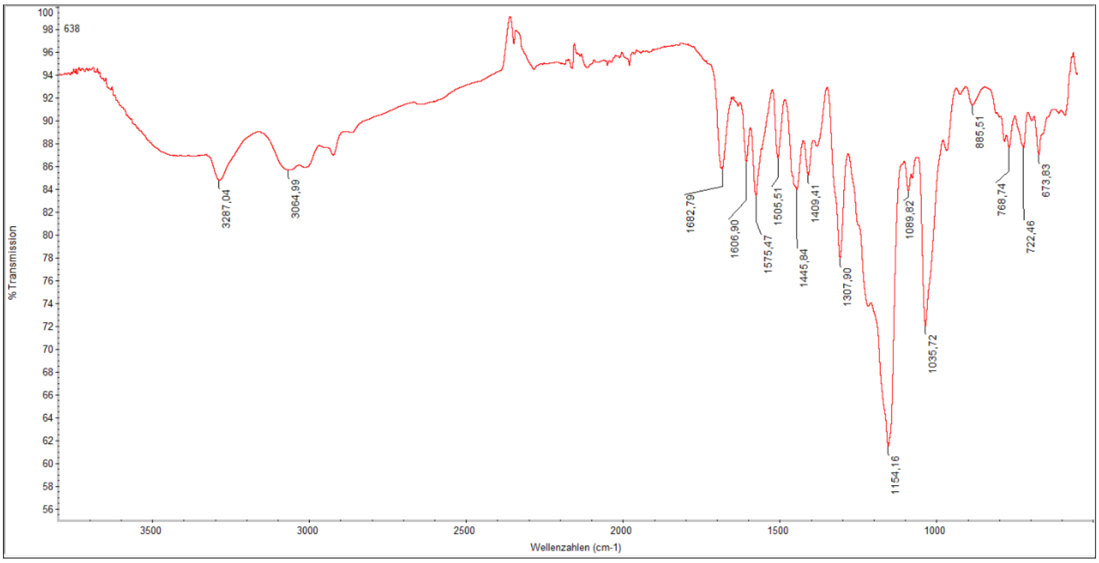

Figure S45: ATR-IR spectrum of compound 14.

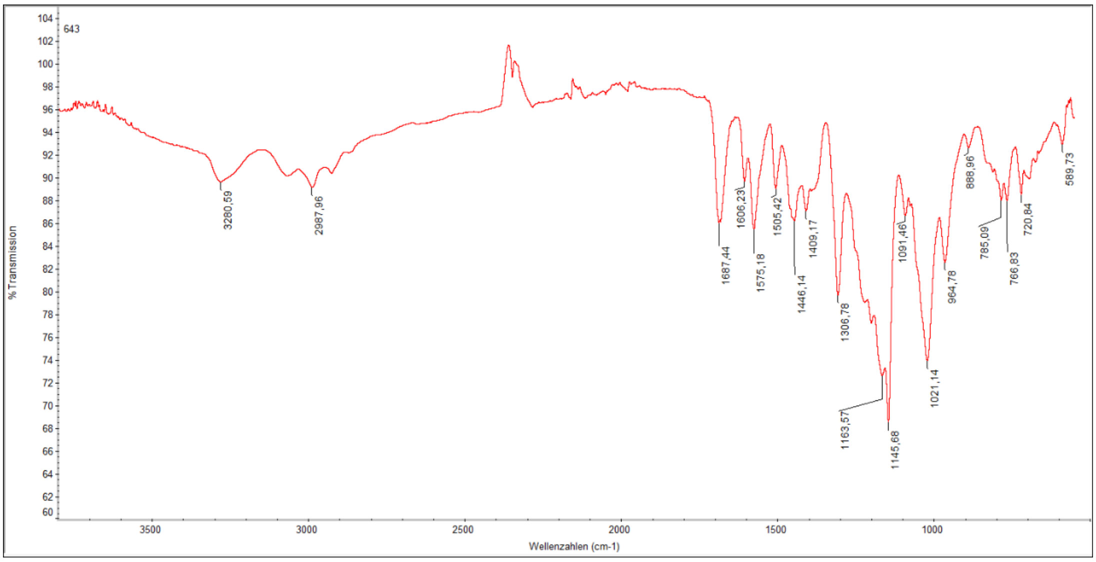

Figure S46: ATR-IR spectrum of compound 15.

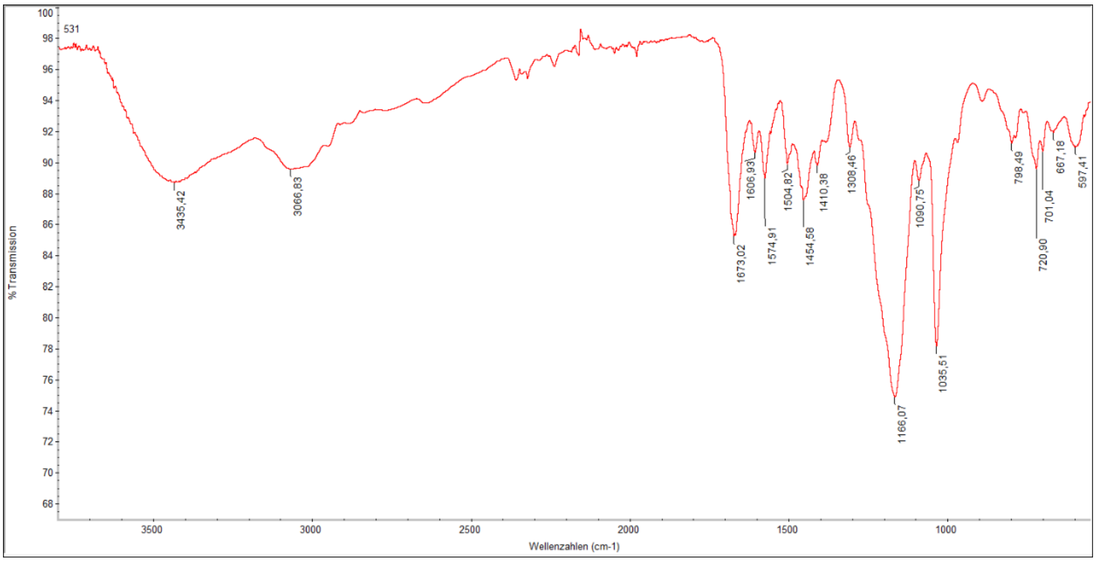

Figure S47: ATR-IR spectrum of compound 44.

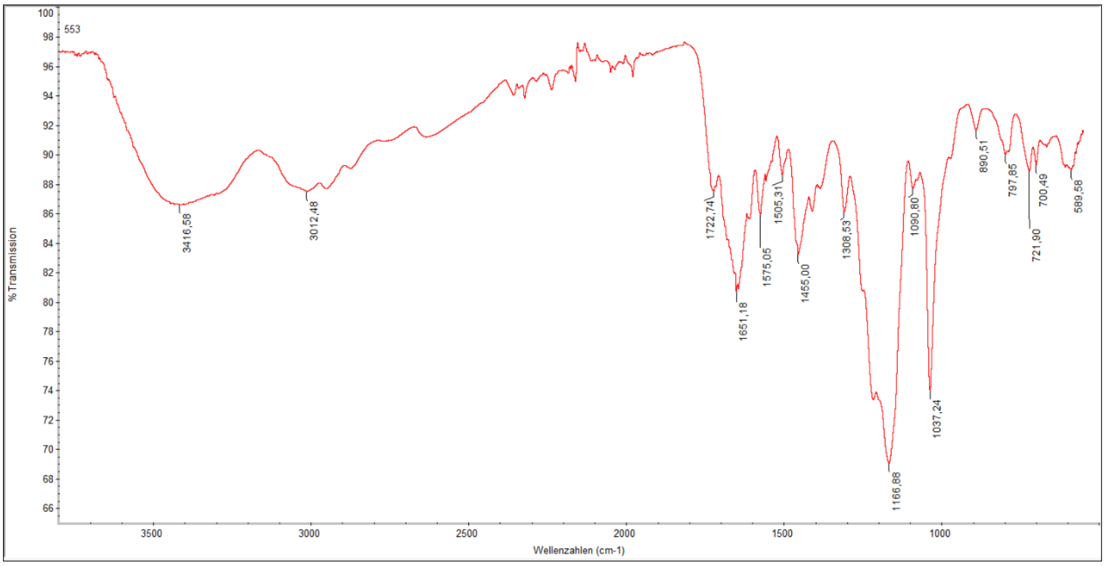

Figure S48: ATR-IR spectrum of compound 1.

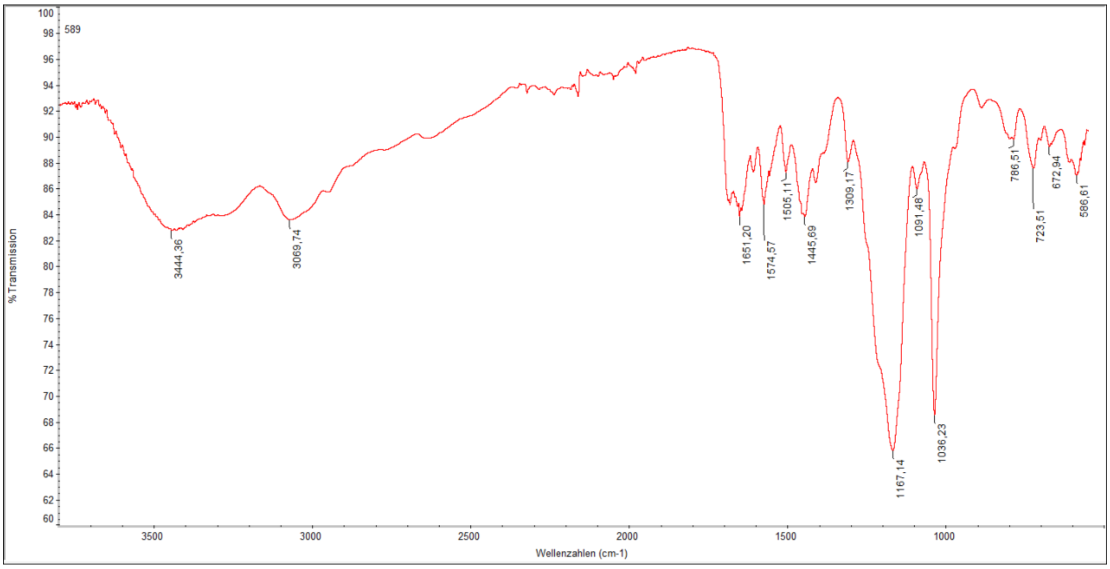

Figure S49: ATR-IR spectrum of compound 45.

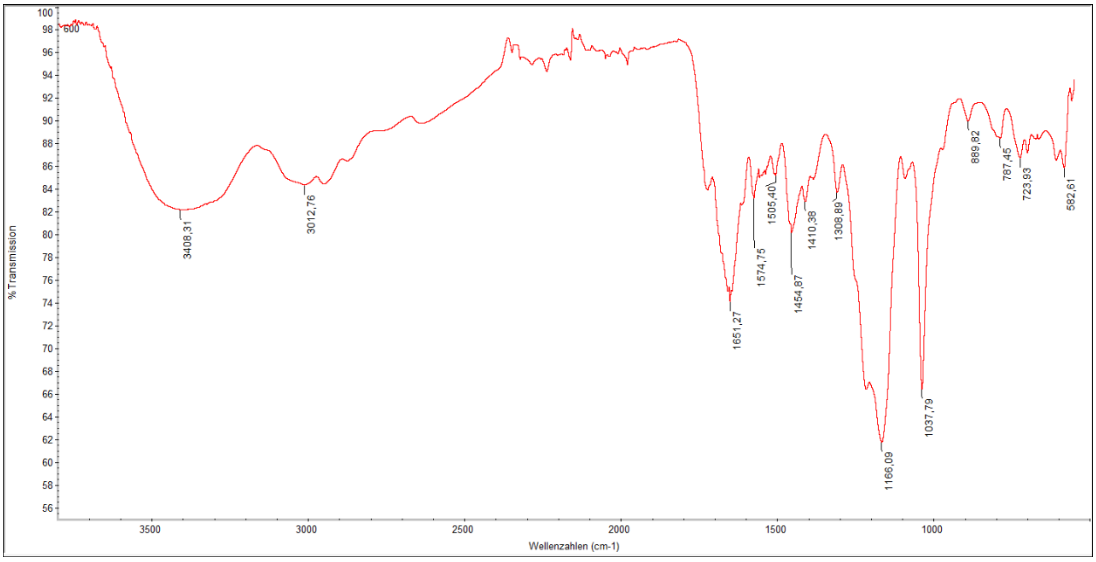

Figure S50: ATR-IR spectrum of compound 2.

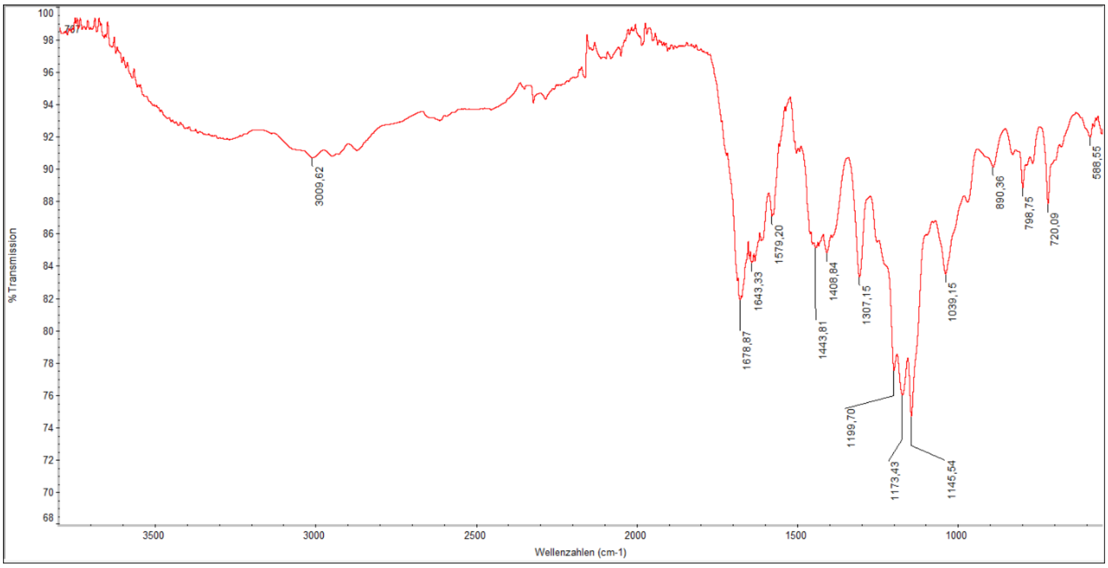

Figure S51: ATR-IR spectrum of compound 4.

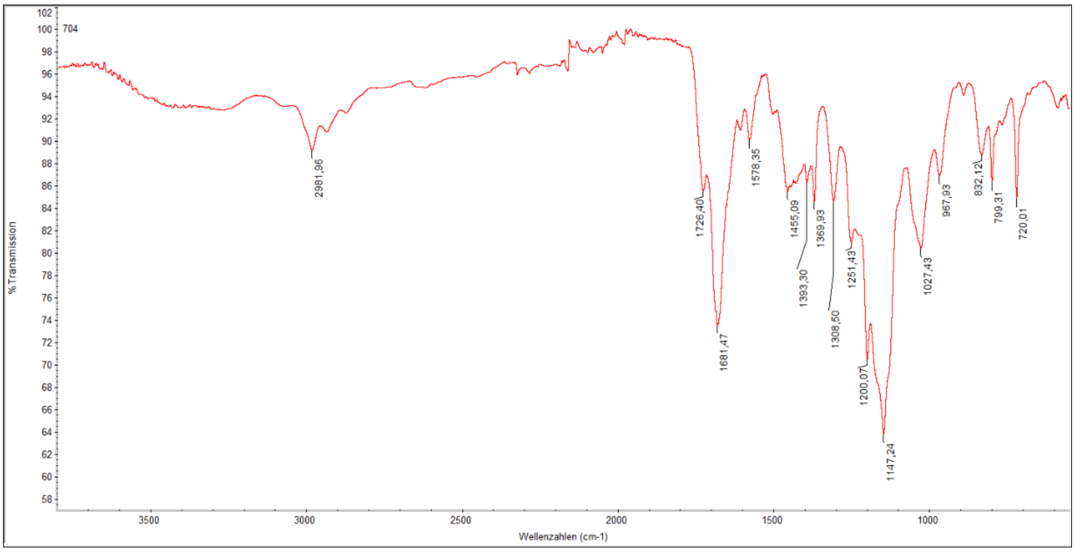

Figure S52: ATR-IR spectrum of compound 10.

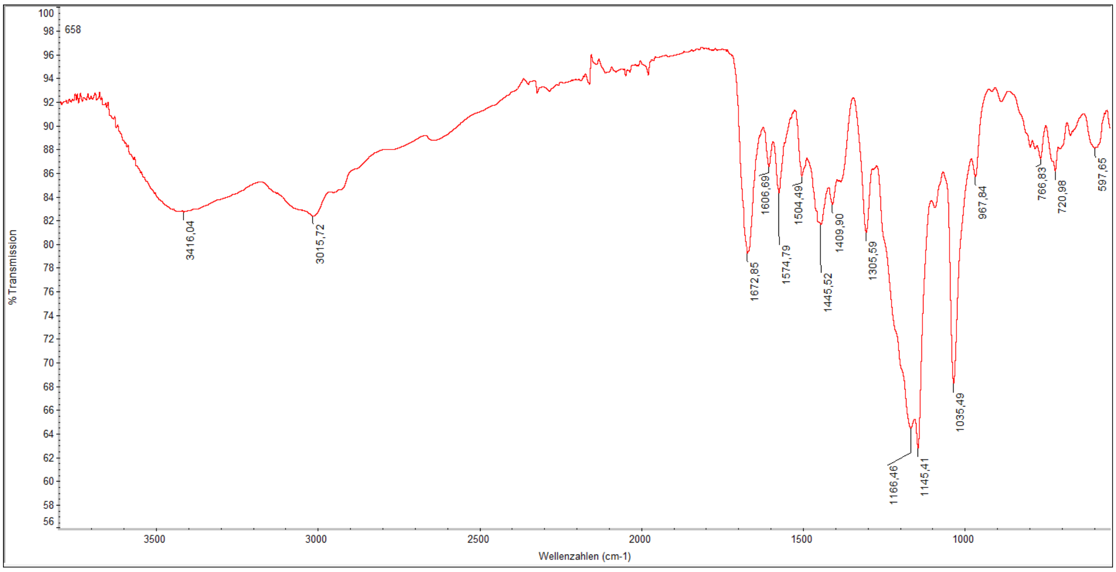

Figure S53: ATR-IR spectrum of compound 46.

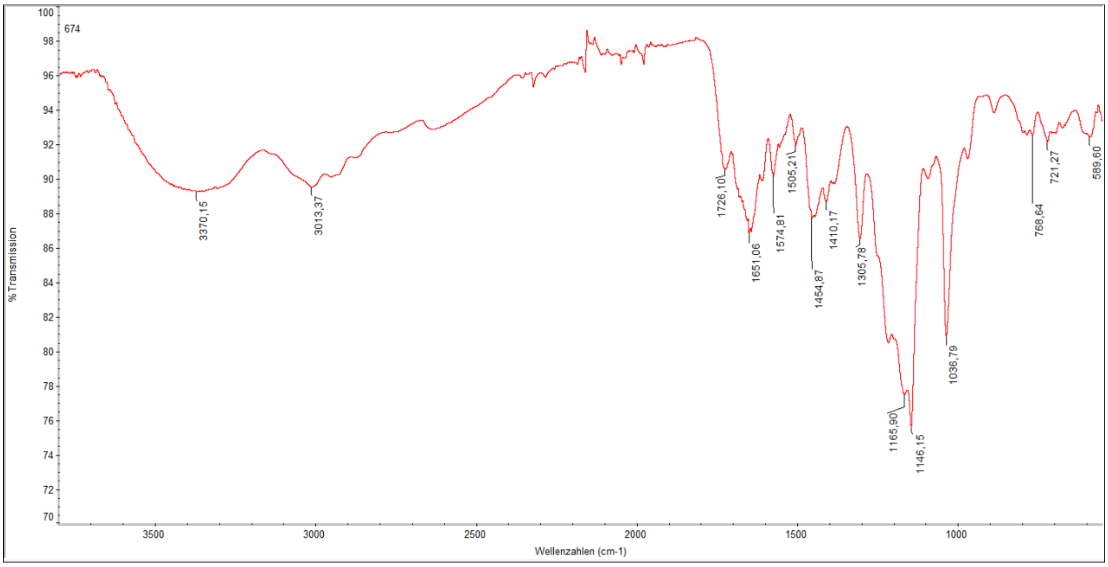

Figure S54: ATR-IR spectrum of compound 3.

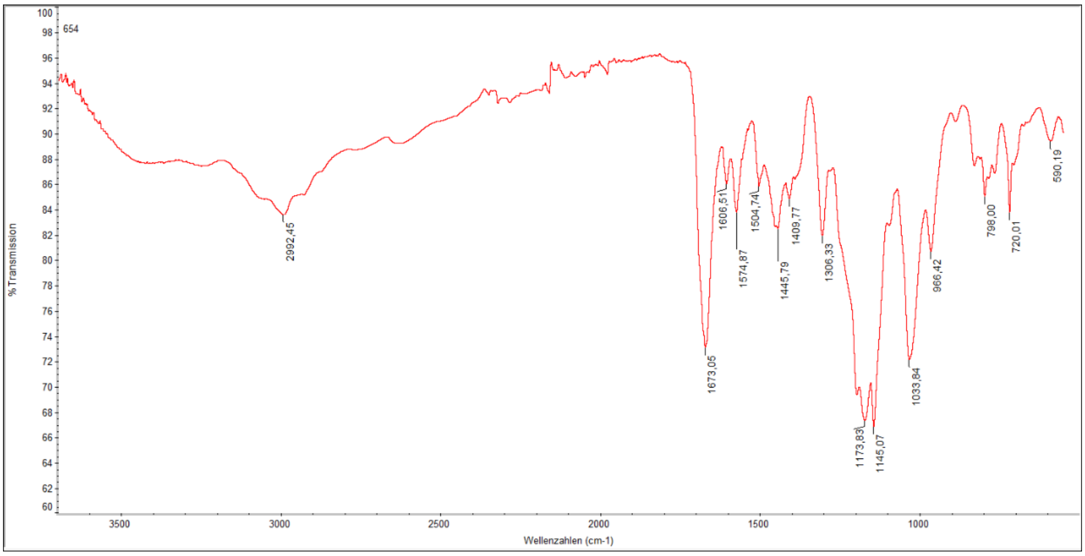

Figure S55: ATR-IR spectrum of compound 47.

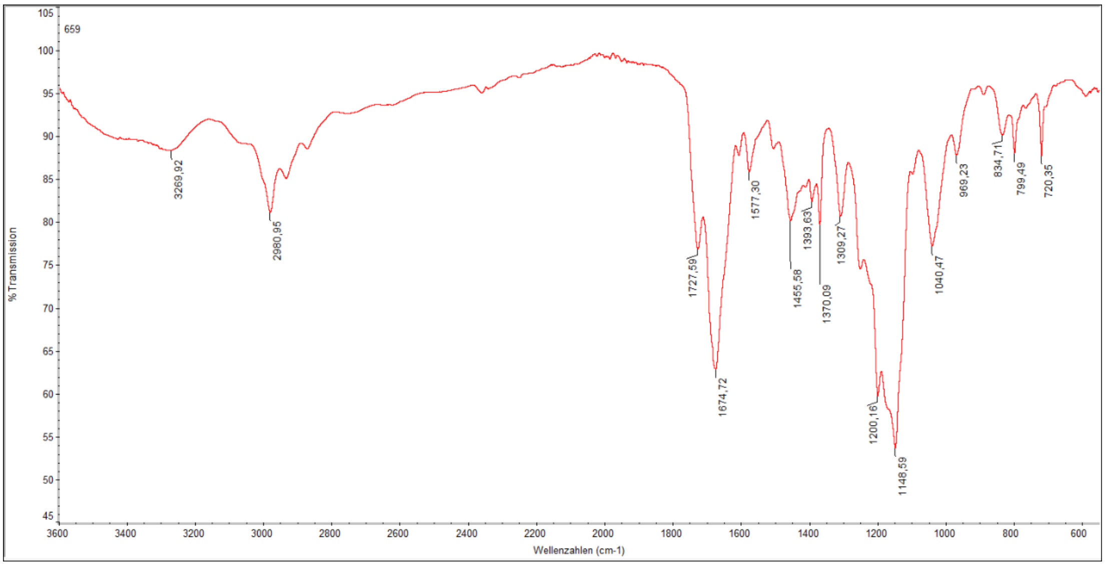

Figure S56: ATR-IR spectrum of compound 11.

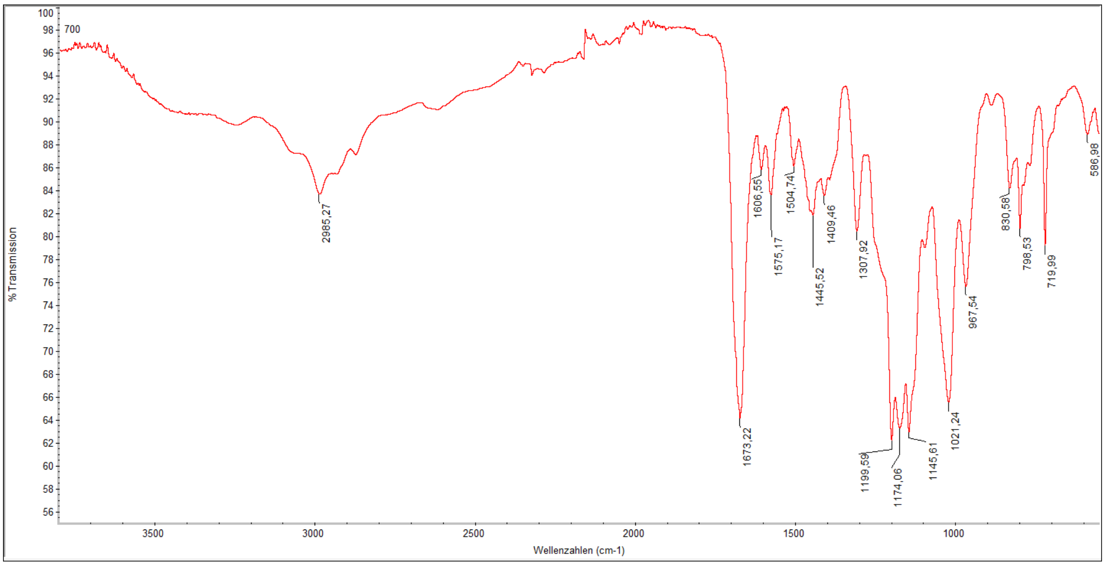

Figure S57: ATR-IR spectrum of compound 48.

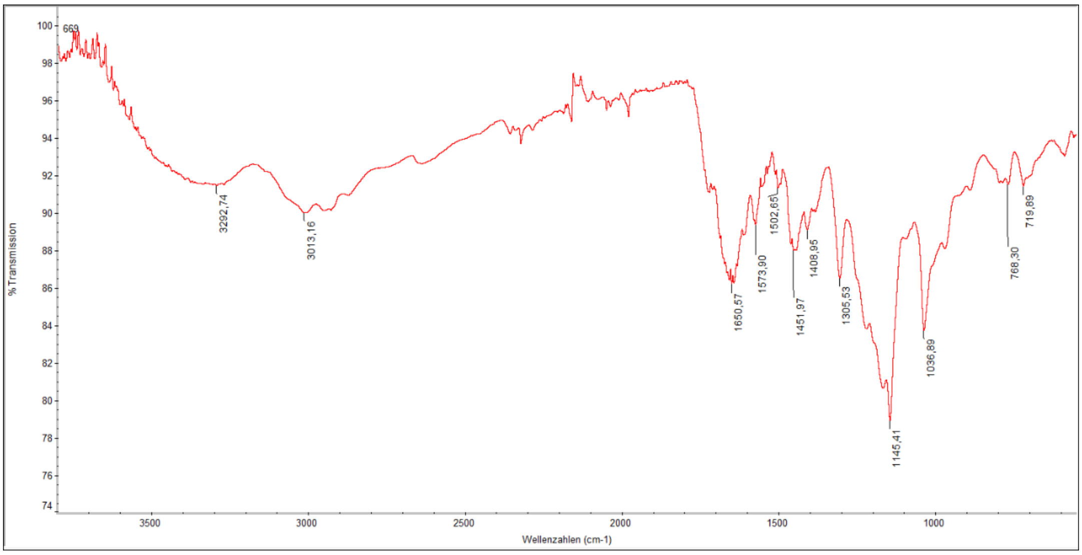

Figure S58: ATR-IR spectrum of compound 12.

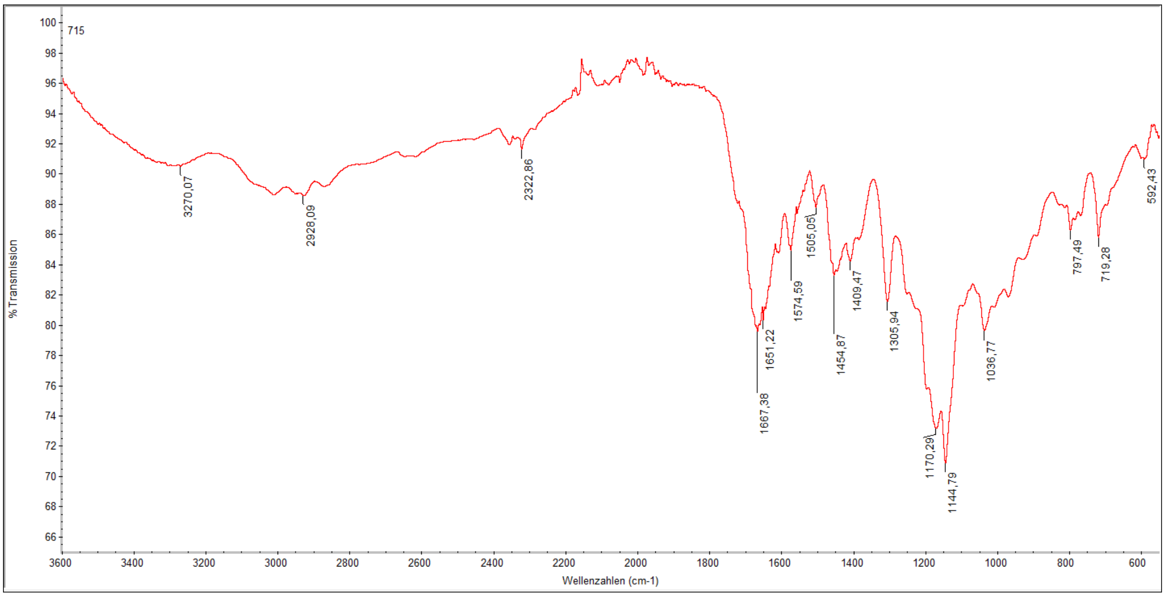

Figure S59: ATR-IR spectrum of compound 6.

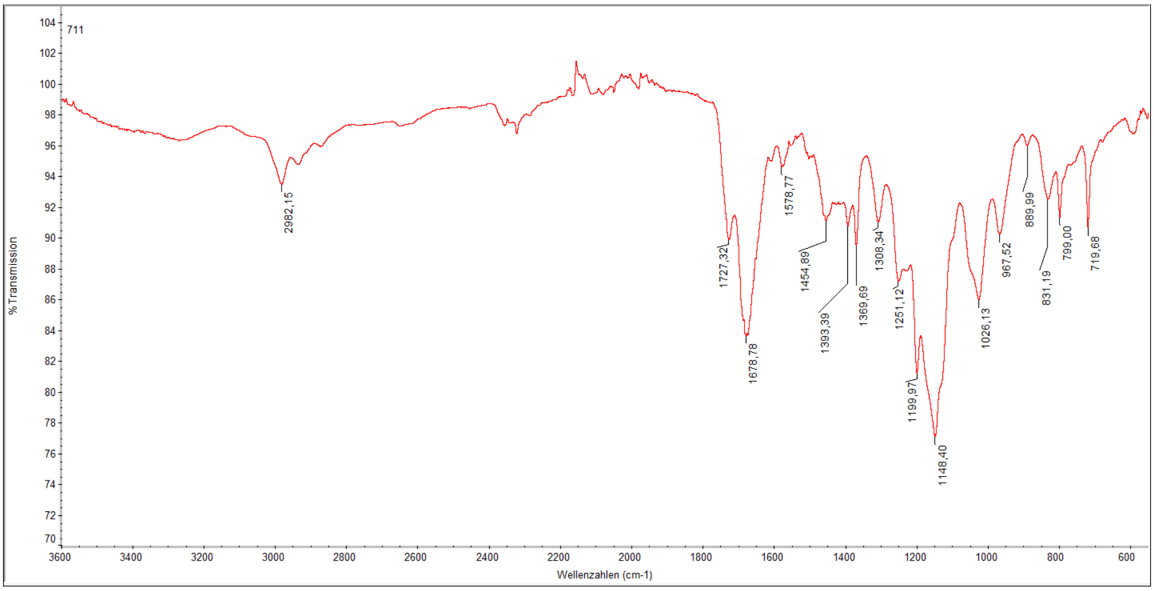

Figure S60: ATR-IR spectrum of compound 12.

4. HR-MS spectra of literature unknown compounds

Fragmentor Voltage	Collision Energy	Ionization Mode
175	0	ESI

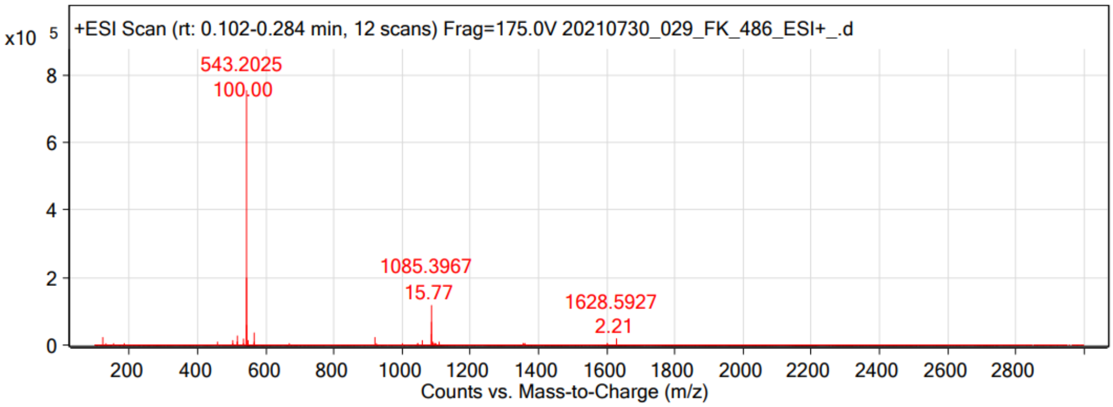

Figure S61: HR-MS Spectrum (ESI+) of compound 16.

Fragmentor Voltage	Collision Energy	Ionization Mode
175	0	ESI

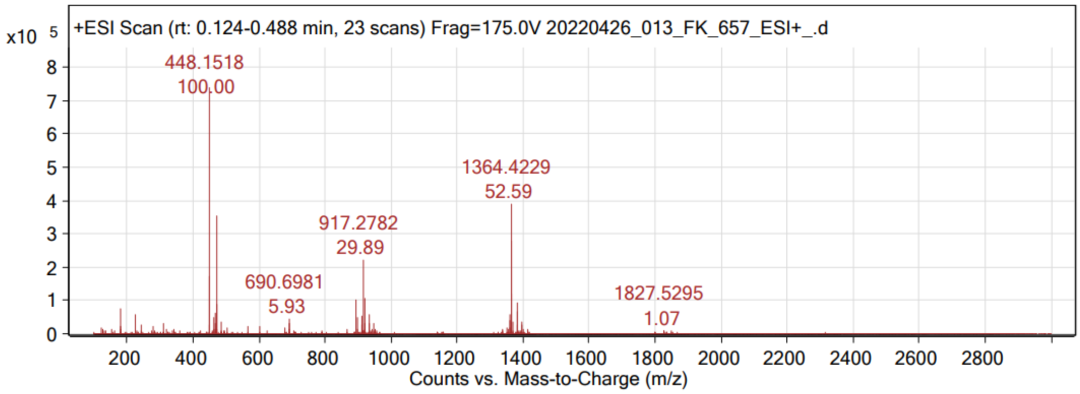

Figure S62: HR-MS Spectrum (ESI+) of compound 21.

Fragmentor Voltage	Collision Energy	Ionization Mode
175	0	ESI

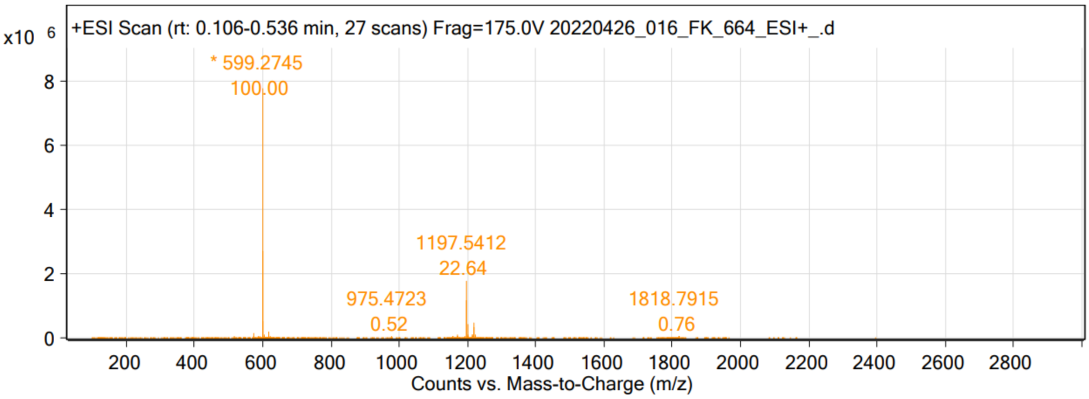

Figure S63: HR-MS Spectrum (ESI+) of compound 18.

Fragmentor Voltage	Collision Energy	Ionization Mode
175	0	ESI

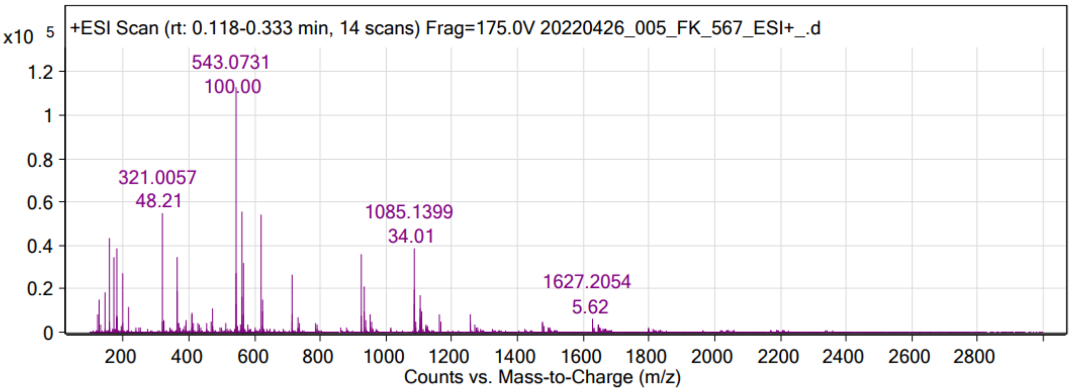

Figure S64: HR-MS Spectrum (ESI+) of compound 20.

Fragmentor Voltage	Collision Energy	Ionization Mode
175	0	ESI

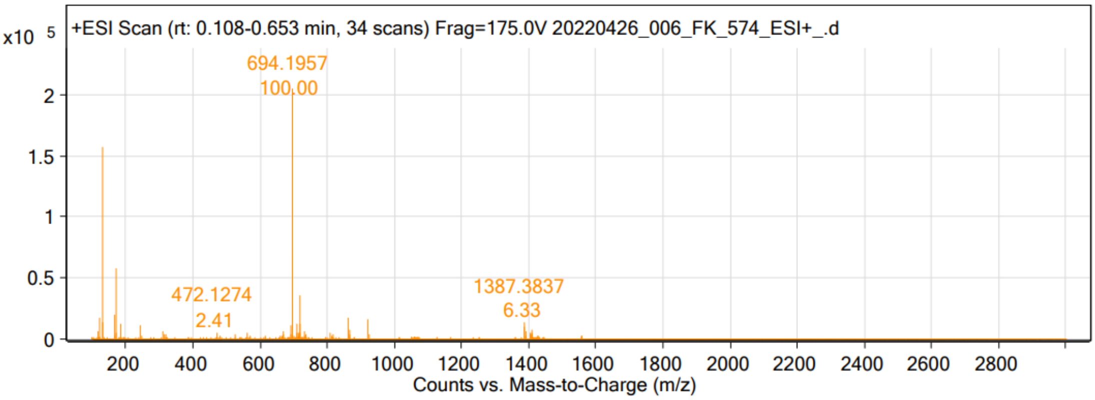

Figure S65: HR-MS Spectrum (ESI+) of compound 17.

Fragmentor Voltage	Collision Energy	Ionization Mode
175	0	ESI

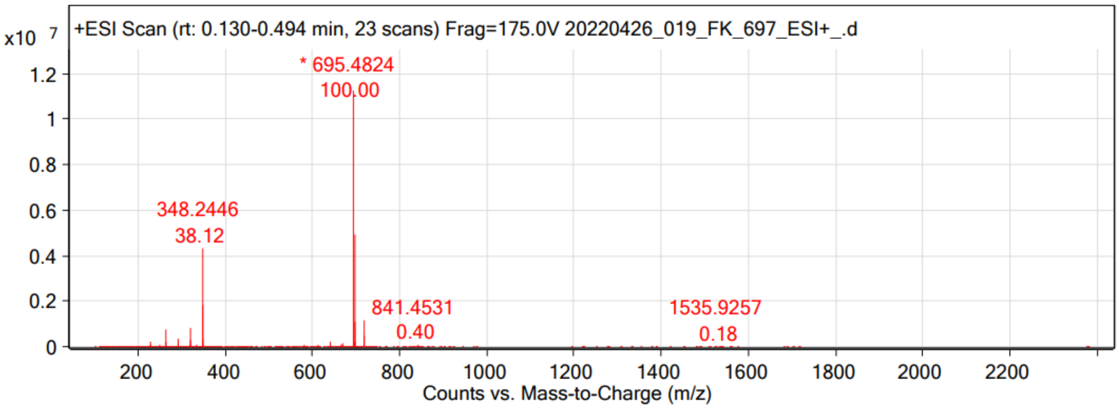

Figure S66: HR-MS Spectrum (ESI+) of compound 23.

Fragmentor Voltage	Collision Energy	Ionization Mode
175	0	ESI

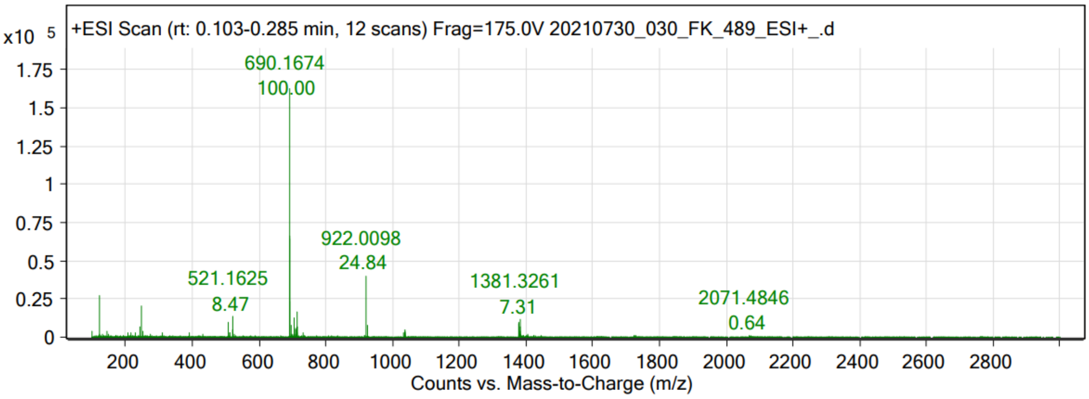

Figure S67: HR-MS Spectrum (ESI+) of compound 37.

Fragmentor Voltage	Collision Energy	Ionization Mode
175	0	ESI

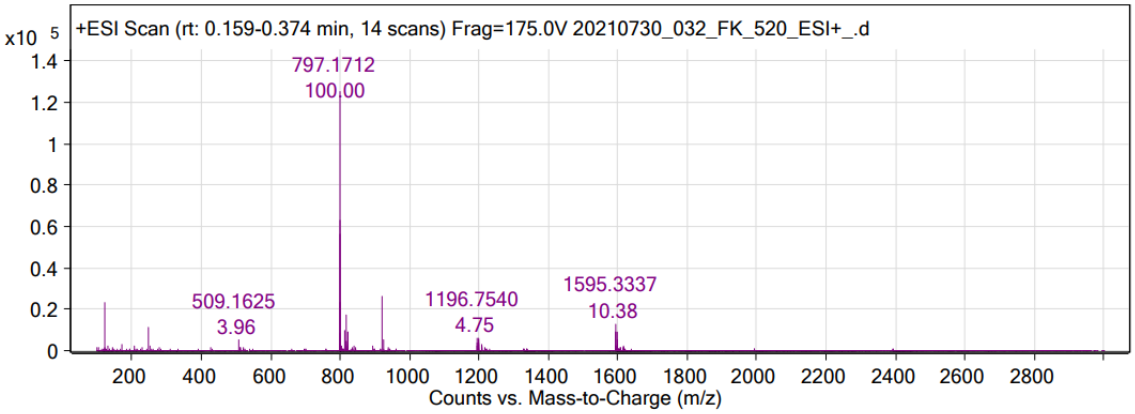

Figure S68: HR-MS Spectrum (ESI+) of compound 13.

Fragmentor Voltage	Collision Energy	Ionization Mode
175	0	ESI

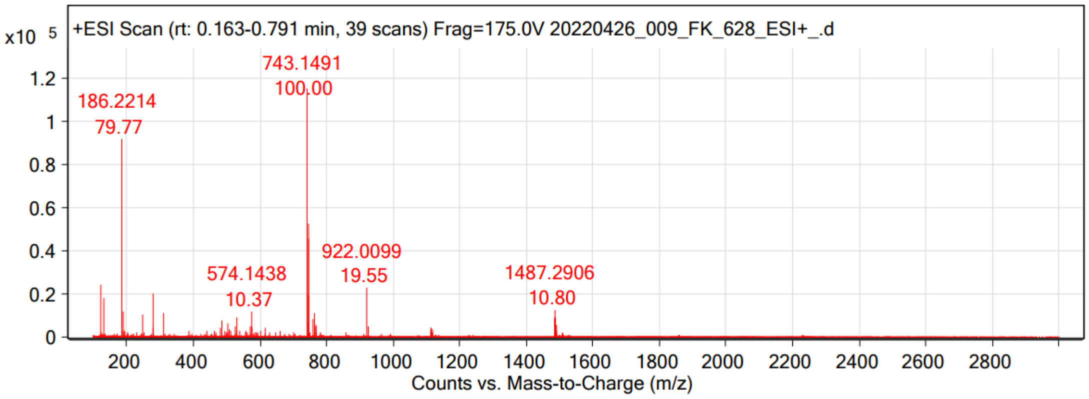

Figure S69: HR-MS Spectrum (ESI+) of compound 38.

Fragmentor Voltage	Collision Energy	Ionization Mode
175	0	ESI

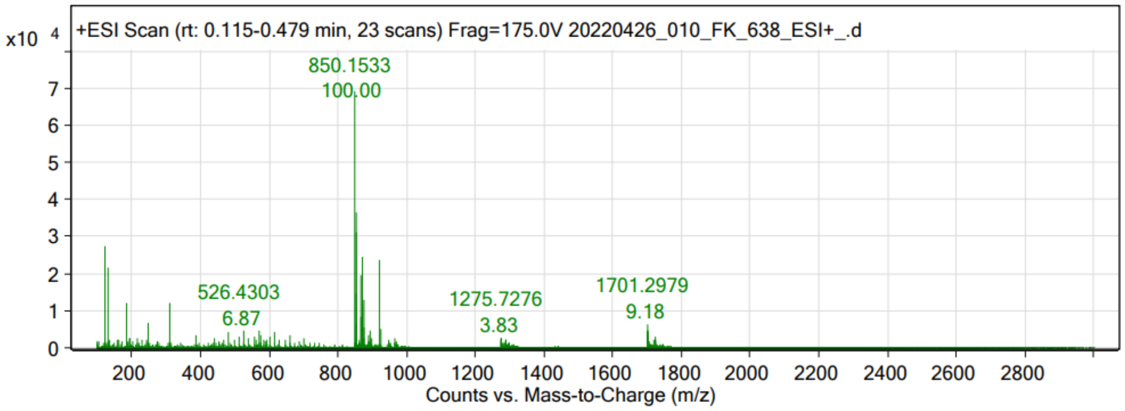

Figure S70: HR-MS Spectrum (ESI+) of compound 14.

Fragmentor Voltage	Collision Energy	Ionization Mode
175	0	ESI

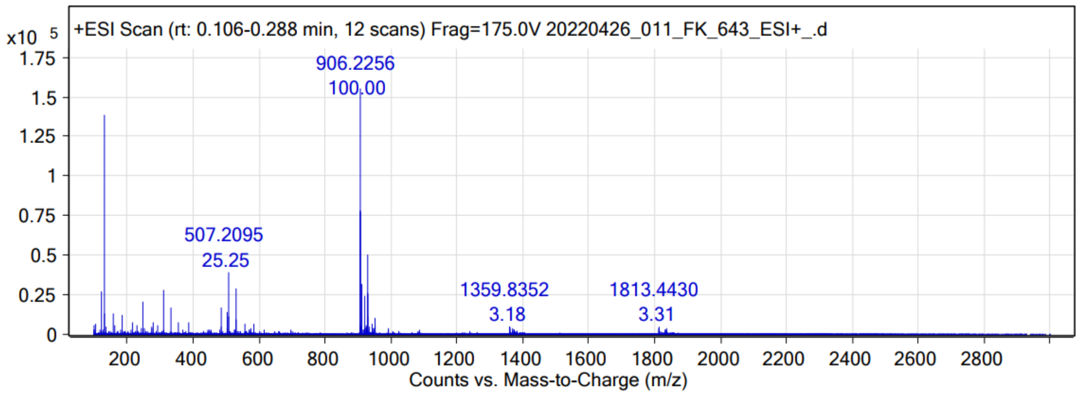

Figure S71: HR-MS Spectrum (ESI+) of compound 15.

Fragmentor Voltage	Collision Energy	Ionization Mode
175	0	ESI

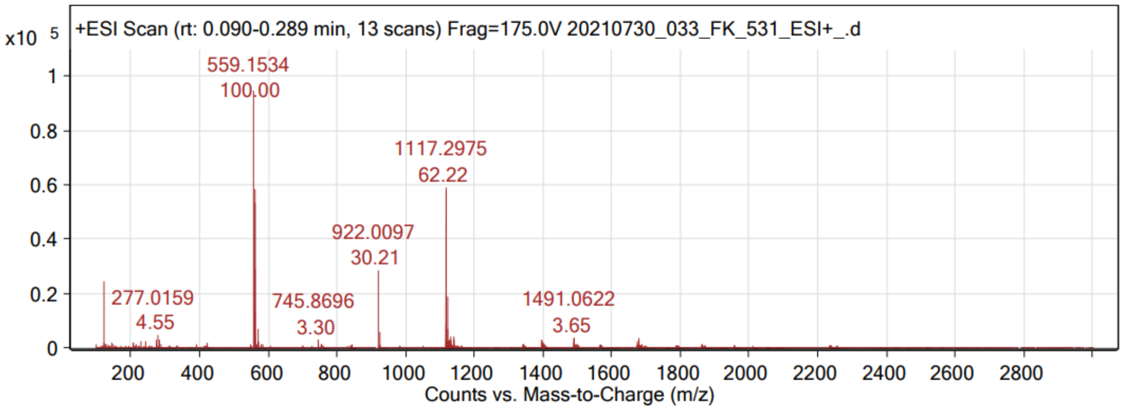

Figure S72: HR-MS Spectrum (ESI+) of compound 44.

Fragmentor Voltage	Collision Energy	Ionization Mode
175	0	ESI

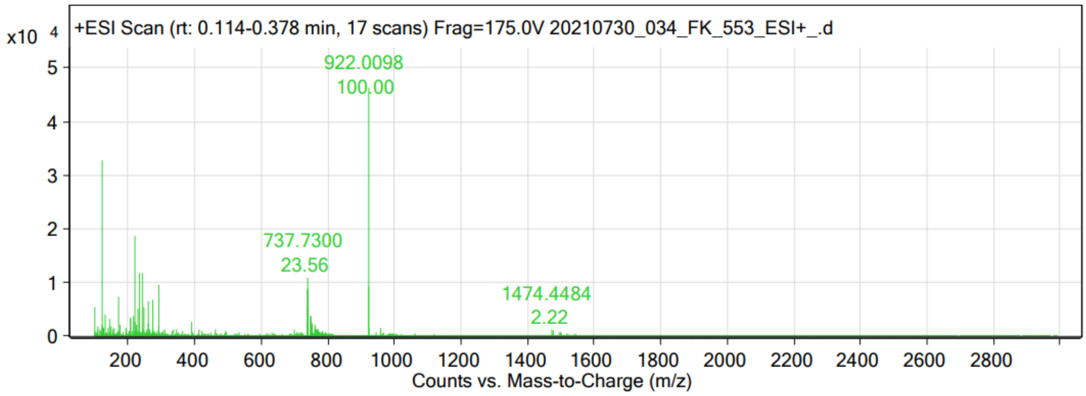

Figure S73: HR-MS Spectrum (ESI+) of compound 1.

Fragmentor Voltage	Collision Energy	Ionization Mode
175	0	ESI

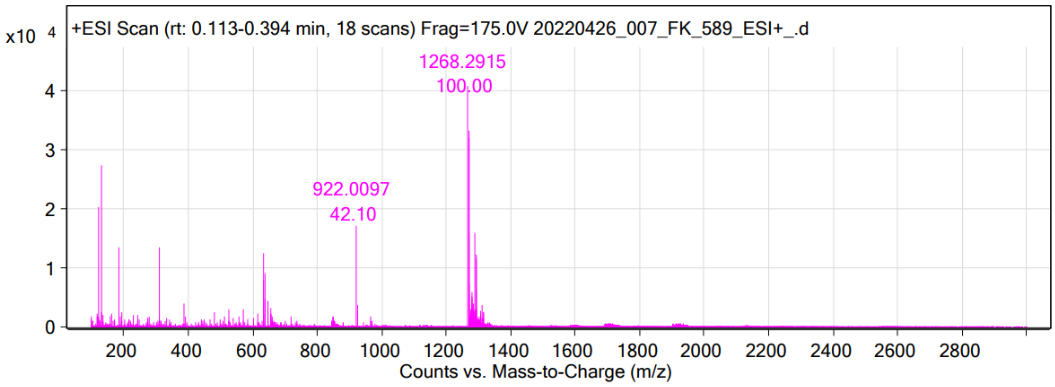

Figure S74: HR-MS Spectrum (ESI+) of compound 45.

Fragmentor Voltage	Collision Energy	Ionization Mode
175	0	ESI

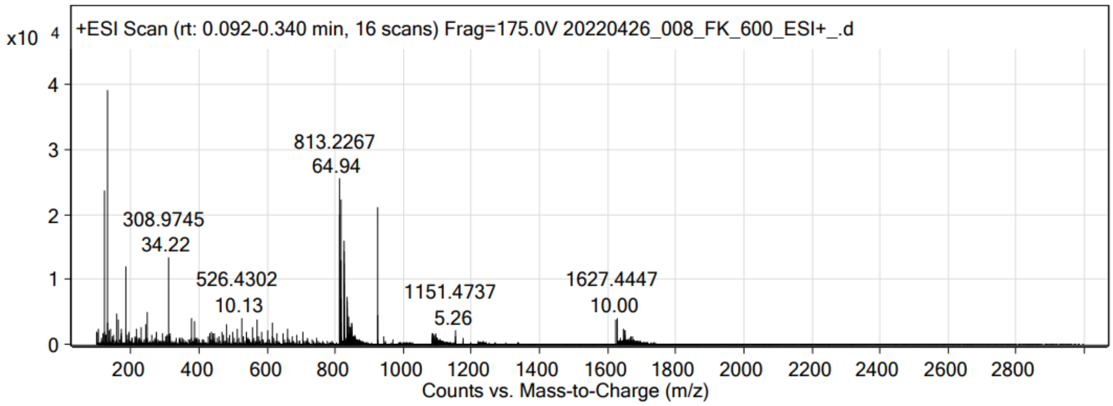

Figure S75: HR-MS Spectrum (ESI+) of compound 2.

Fragmentor Voltage	Collision Energy	Ionization Mode
175	0	ESI

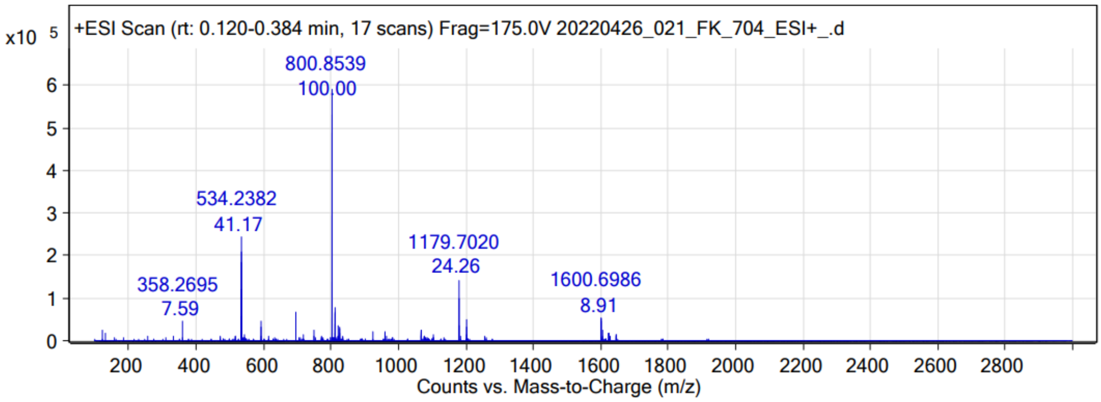

Figure S76: HR-MS Spectrum (ESI+) of compound 10.

Fragmentor Voltage	Collision Energy	Ionization Mode
175	0	ESI

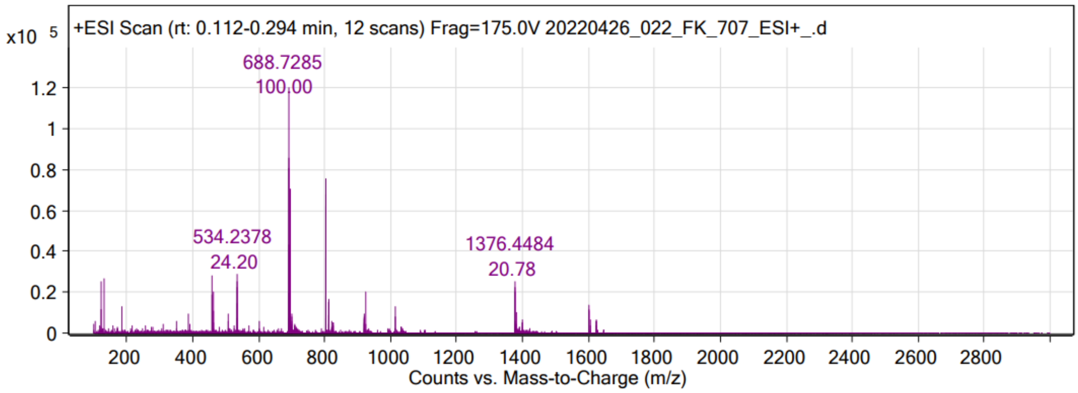

Figure S77: HR-MS Spectrum (ESI+) of compound 4.

Fragmentor Voltage	Collision Energy	Ionization Mode
175	0	ESI

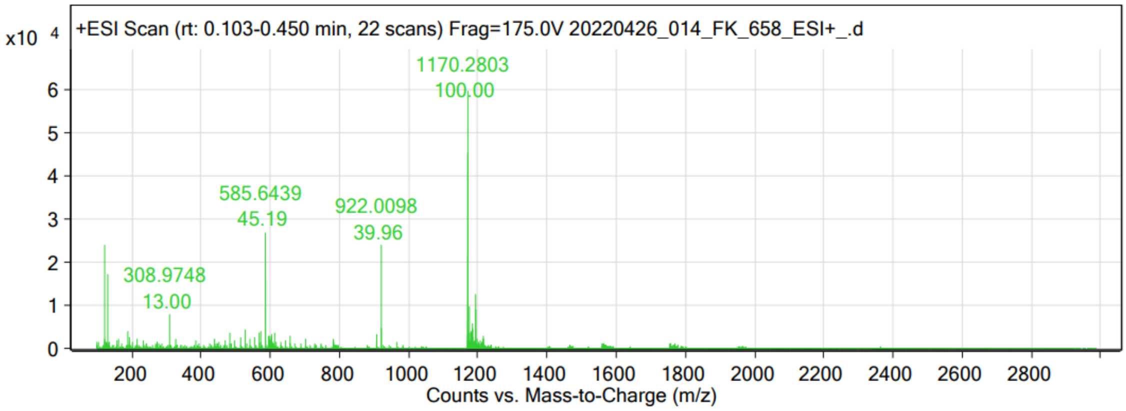

Figure S78: HR-MS Spectrum (ESI+) of compound 46.

Fragmentor Voltage	Collision Energy	Ionization Mode
175	0	ESI

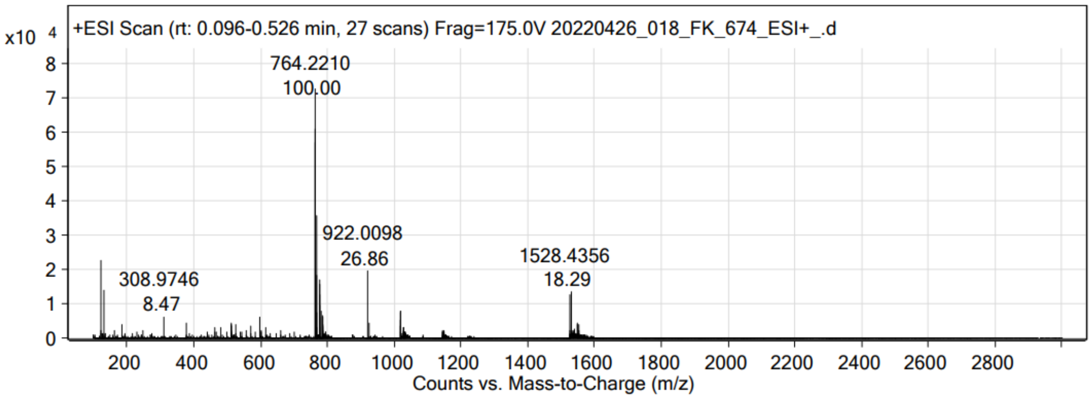

Figure S79: HR-MS Spectrum (ESI+) of compound 3.

Fragmentor Voltage	Collision Energy	Ionization Mode
175	0	ESI

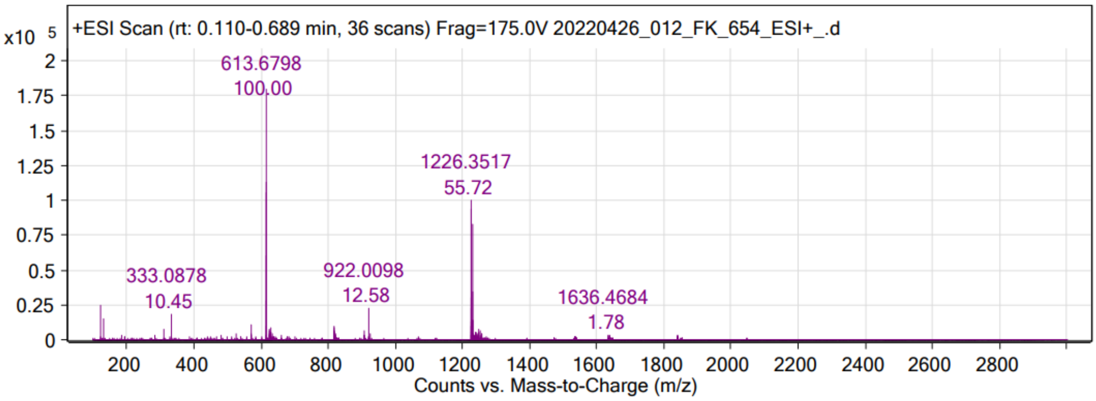

Figure S80: HR-MS Spectrum (ESI+) of compound 47.

Fragmentor Voltage	Collision Energy	Ionization Mode
175	0	ESI

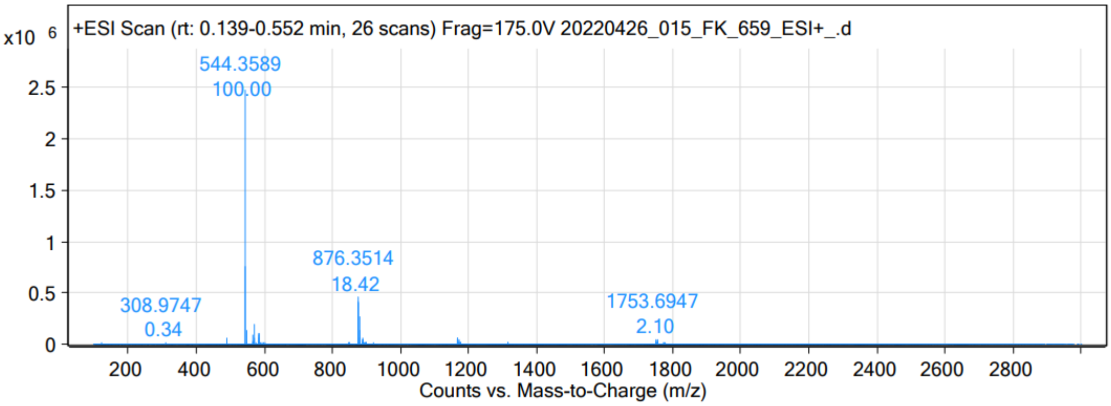

Figure S81: HR-MS Spectrum (ESI+) of compound 11.

Fragmentor Voltage	Collision Energy	Ionization Mode
175	0	ESI

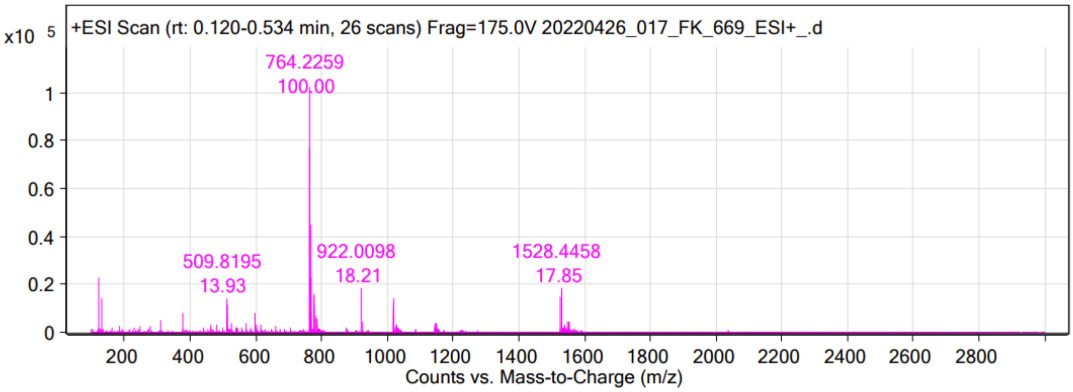

Figure S82: HR-MS Spectrum (ESI+) of compound 5.

Fragmentor Voltage	Collision Energy	Ionization Mode
175	0	ESI

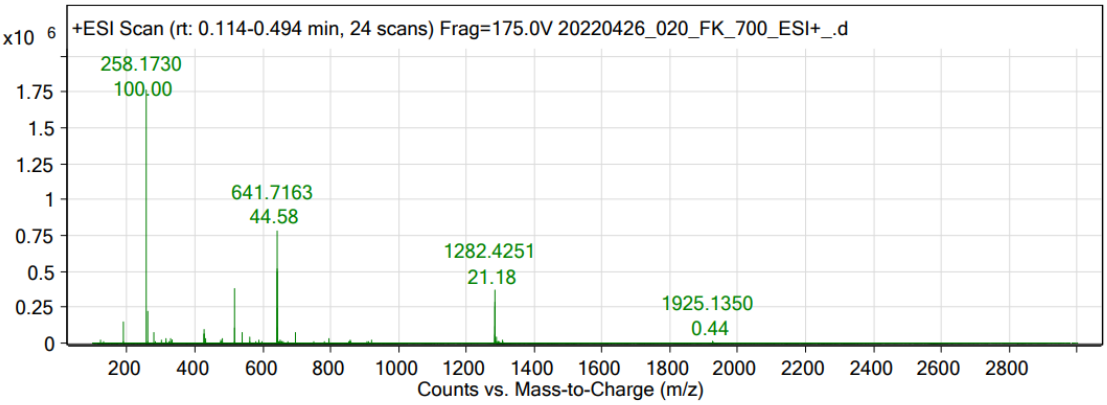

Figure S83: HR-MS Spectrum (ESI+) of compound 48.

Fragmentor Voltage	Collision Energy	Ionization Mode
175	0	ESI

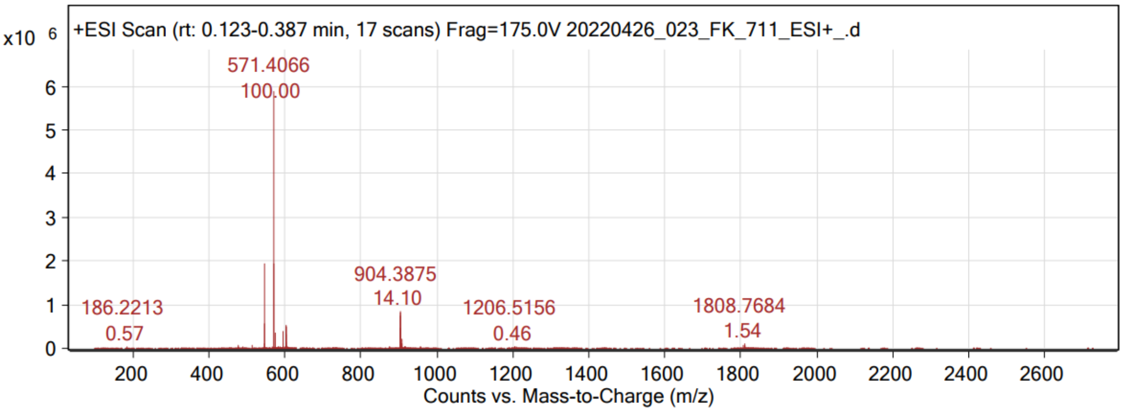

Figure S84: HR-MS Spectrum (ESI+) of compound 12.

Fragmentor Voltage	Collision Energy	Ionization Mode
175	0	ESI

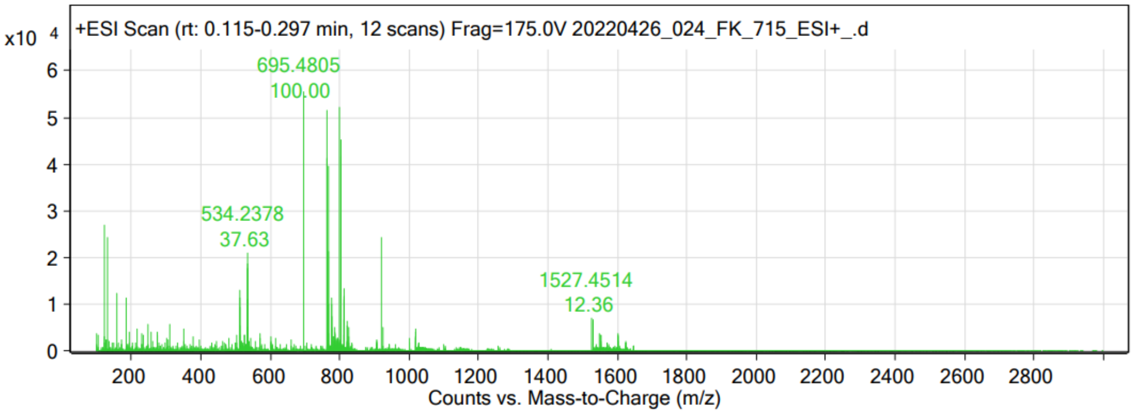

Figure S85: HR-MS Spectrum (ESI+) of compound 6.

5. HPLC chromatograms of HPLC purified compounds

System A: RP-HPLC, analytical (Agilent Zorbax 300 C-18, 5 µm, 4.6 x 150 mm) with 10–95% acetonitrile (0.1% TFA) in water (0.1% TFA) in a linear gradient over 15 min, 1 mL/min, 23 °C, detection at 254 nm.

System B: RP-HPLC, analytical (Agilent Zorbax 300 C-18, 5 µm, 4.6 x 200 mm) with 10–95% acetonitrile (0.1% TFA) in water (0.1% TFA) in a linear gradient over 30 min, 1 mL/min, 23 °C, detection at 254 nm.

System C: RP-HPLC, analytical (Phenomenex Jupiter 300 C-18, 5 µm, 4.6 x 250 mm) with 10–95% acetonitrile (0.1% TFA) in water (0.1% TFA) in a linear gradient over 40 min, 1 mL/min, 23 °C, γ-detection.

System D: RP-HPLC, analytical (Kintetex® 5 µm Phenyl-Hexyl 100 Å) with 5–95% acetonitrile (0.1% TFA) in water (0.1% TFA) in a linear gradient over 20 min, 1 mL/min, 23 °C, γ-detection.

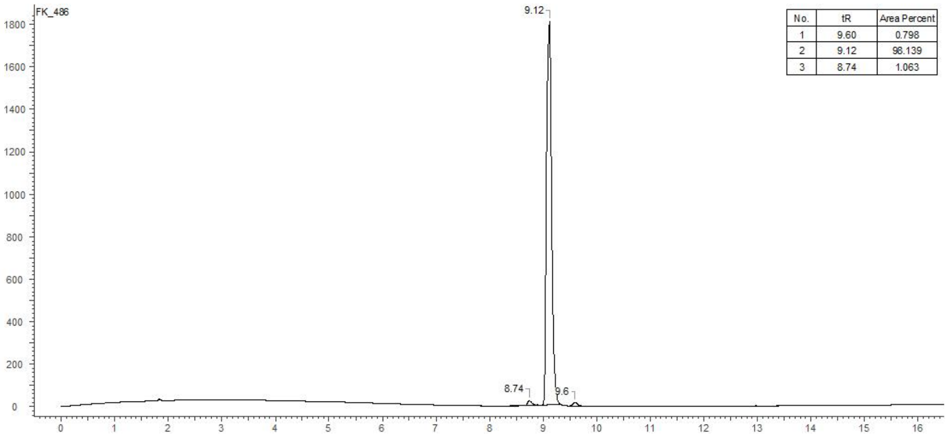

Figure S86: Analytical RP-HPLC chromatogram (System A) of compound 16.

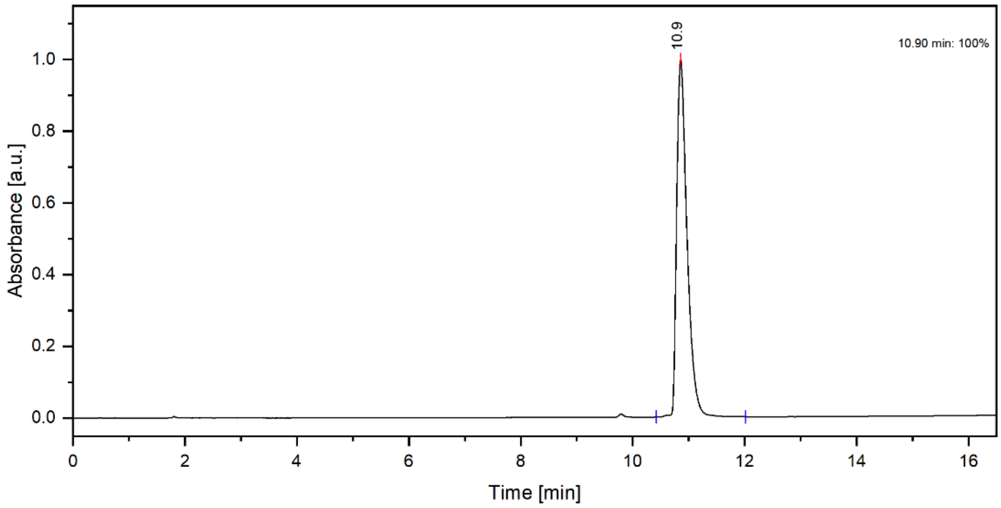

Figure S87: Analytical RP-HPLC chromatogram (System A) of compound 21.

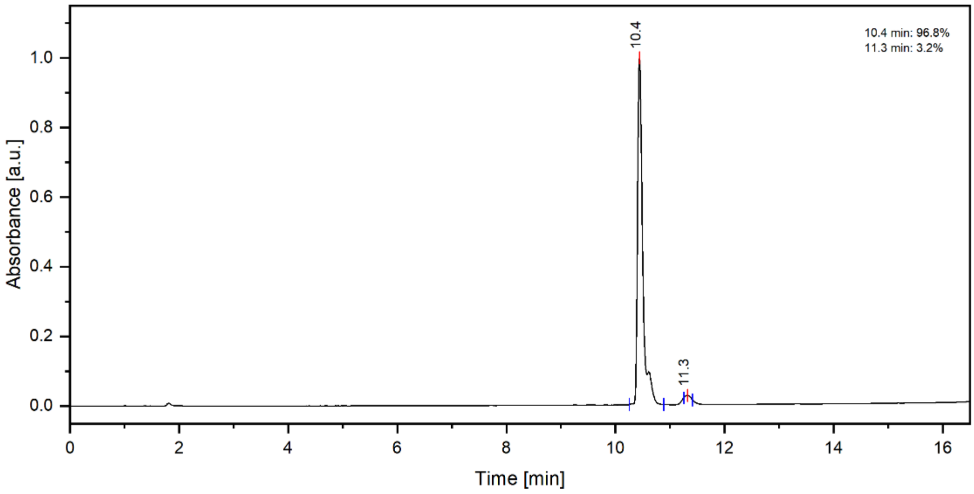

Figure S88: Analytical RP-HPLC chromatogram (System A) of compound 18.

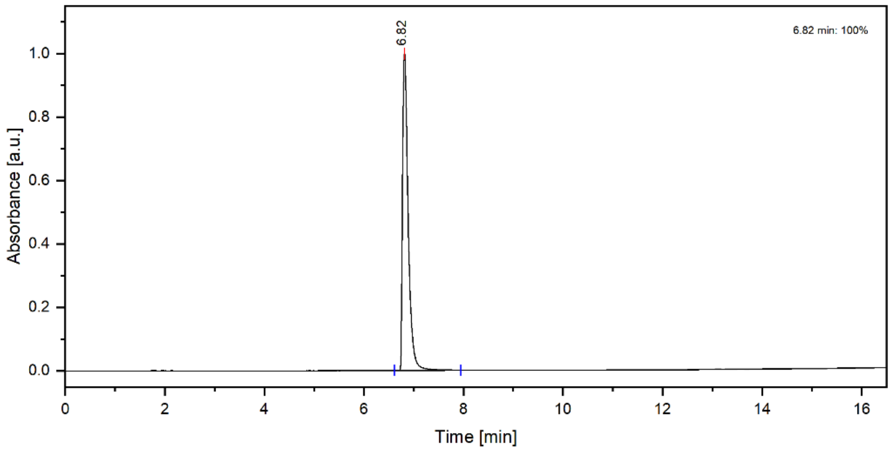

Figure S89: Analytical RP-HPLC chromatogram (System A) of compound 20.

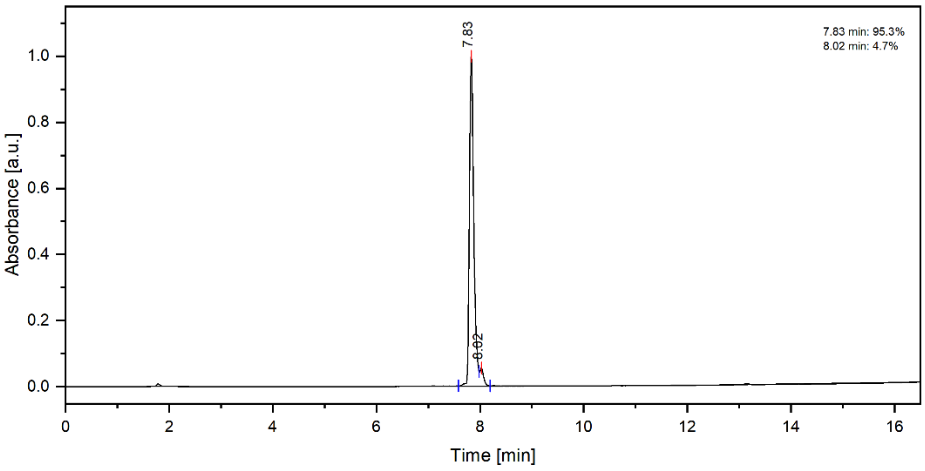

Figure S90: Analytical RP-HPLC chromatogram (System A) of compound 17.

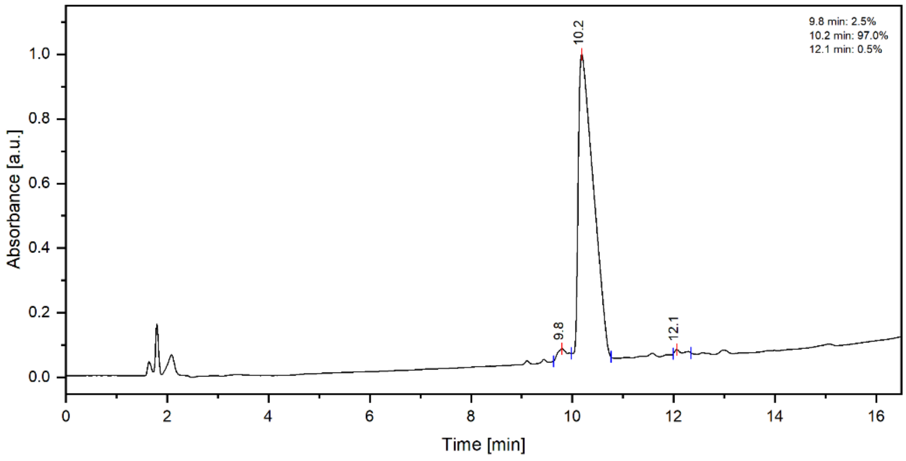

Figure S91: Analytical RP-HPLC chromatogram (System A) of compound 23.

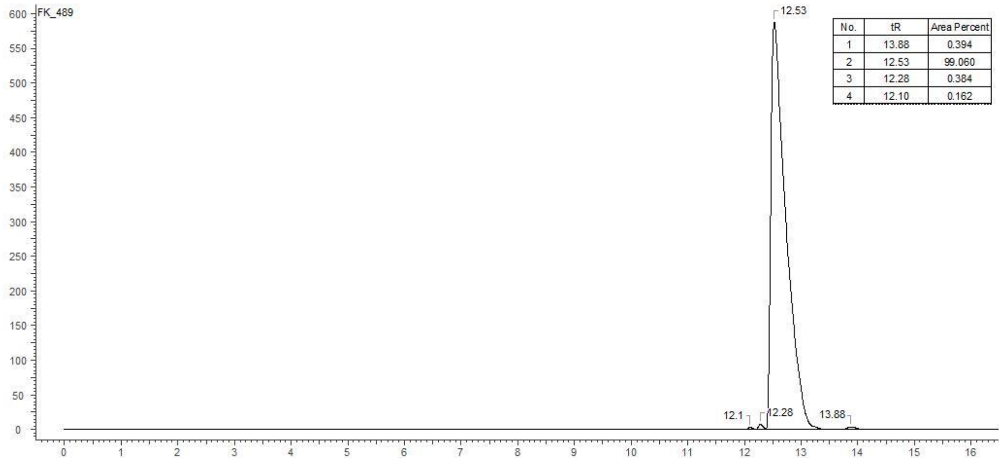

Figure S92: Analytical RP-HPLC chromatogram (System A) of compound 37.

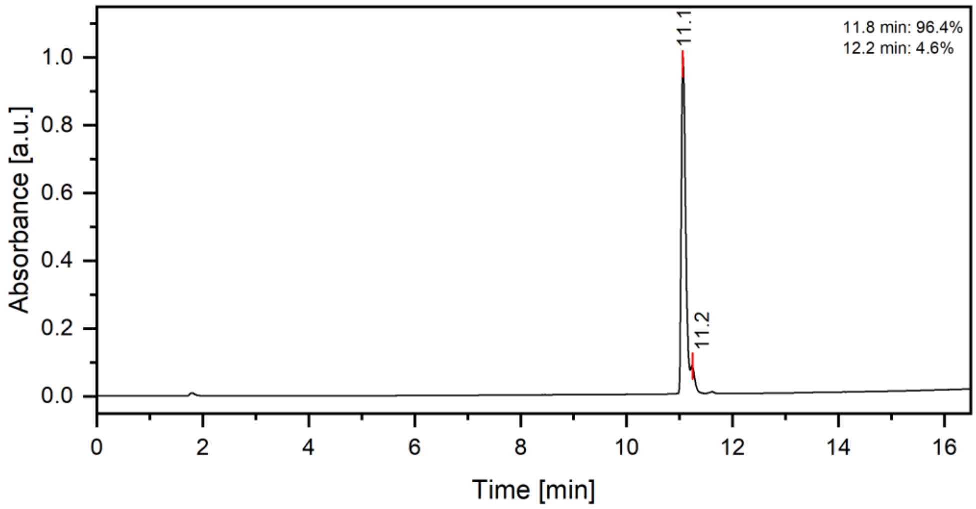

Figure S93: Analytical RP-HPLC chromatogram (System A) of compound 13.

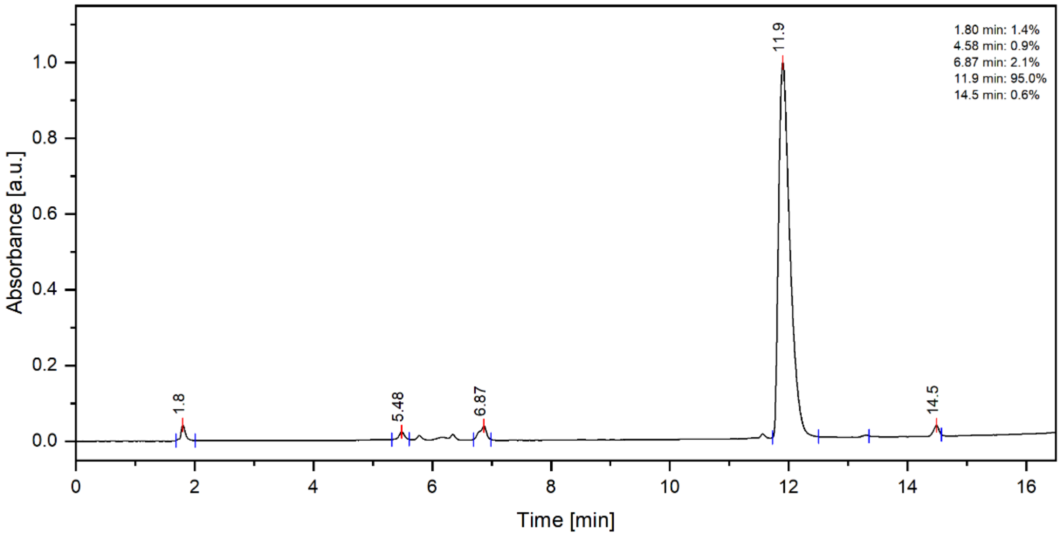

Figure S94: Analytical RP-HPLC chromatogram (System A) of compound 38.

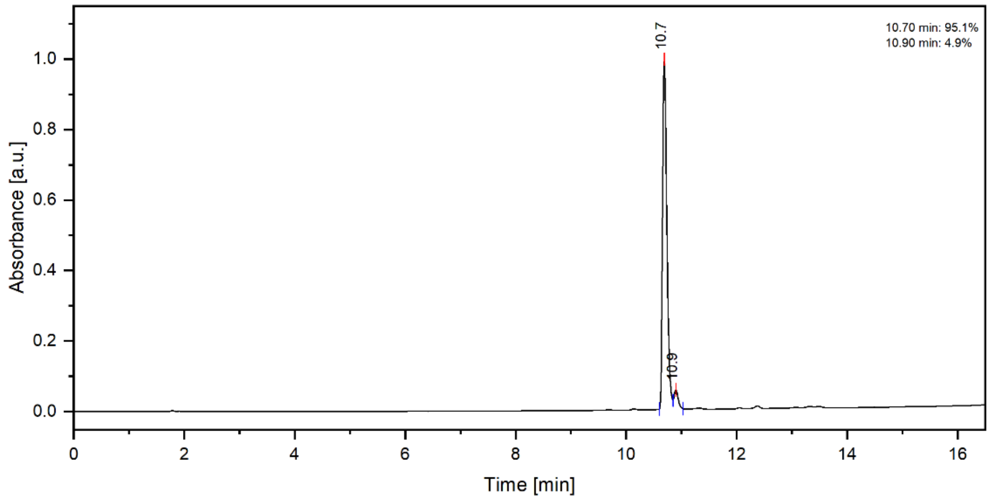

Figure S95: Analytical RP-HPLC chromatogram (System A) of compound 14.

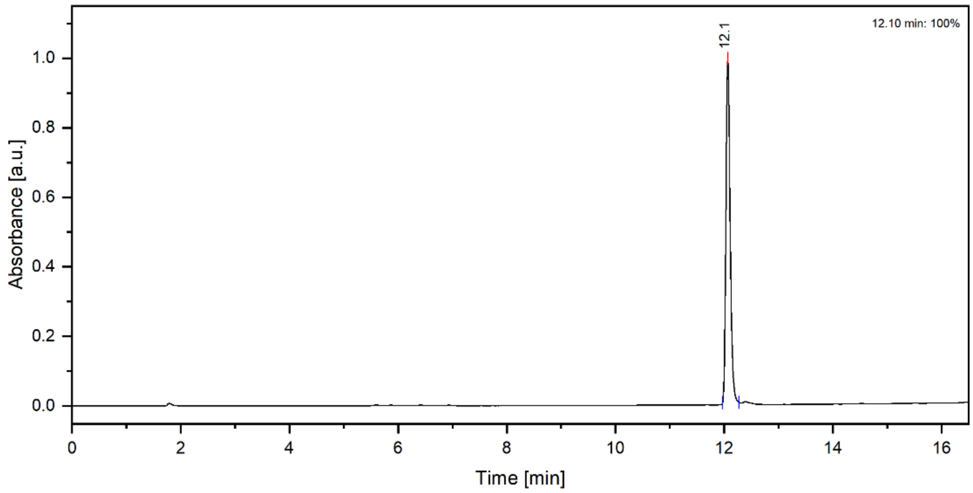

Figure S96: Analytical RP-HPLC chromatogram (System A) of compound 15.

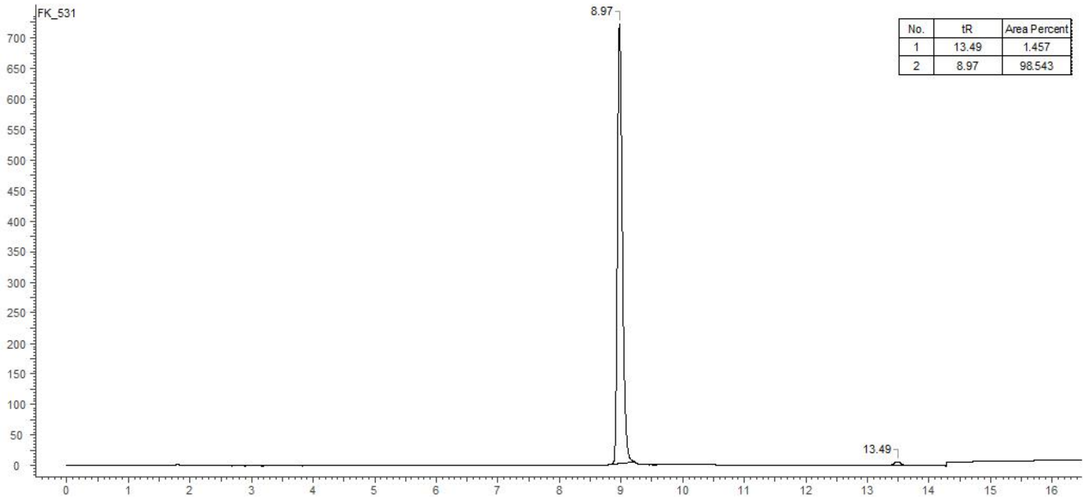

Figure S97: Analytical RP-HPLC chromatogram (System A) of compound 44.

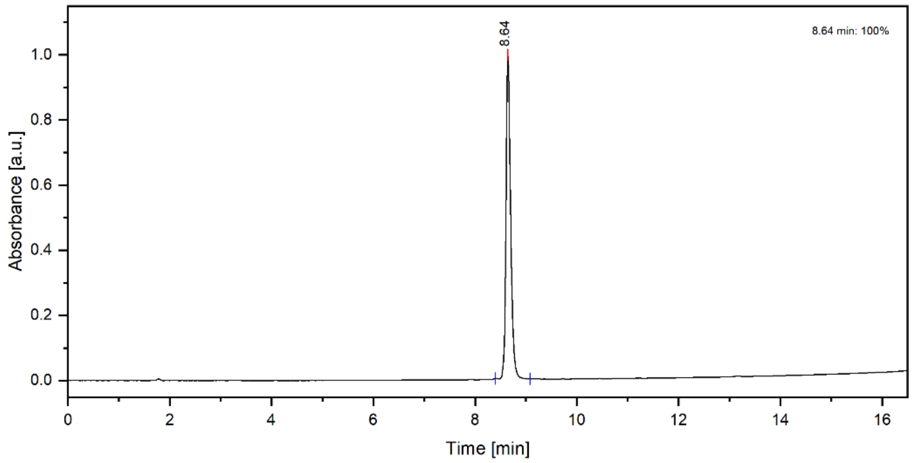

Figure S98: Analytical RP-HPLC chromatogram (System A) of compound 1.

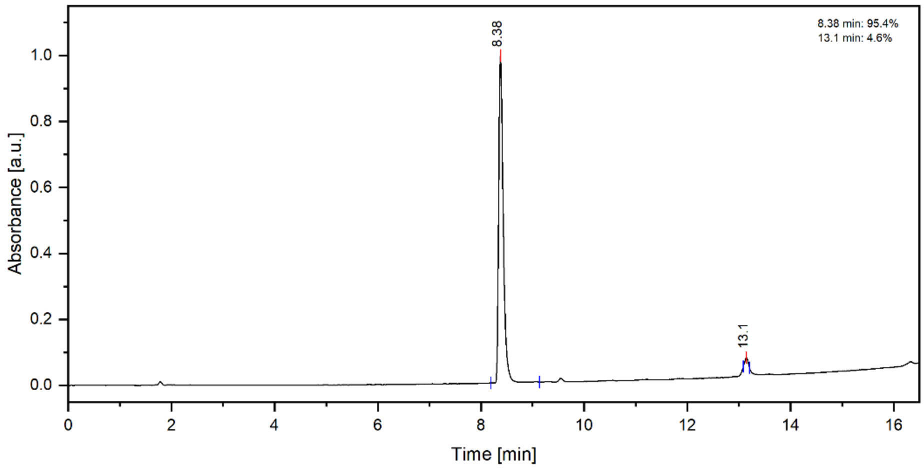

Figure S99: Analytical RP-HPLC chromatogram (System A) of compound 45.

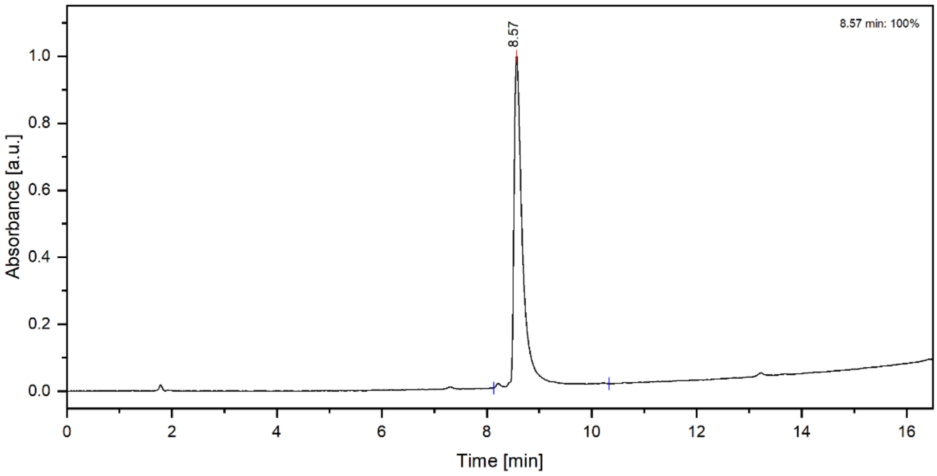

Figure S100: Analytical RP-HPLC chromatogram (System A) of compound 2.

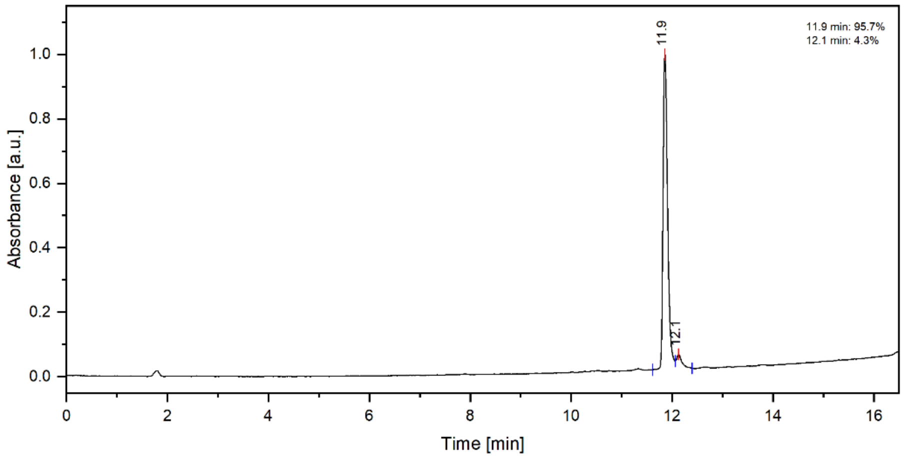

Figure S101: Analytical RP-HPLC chromatogram (System A) of compound 10.

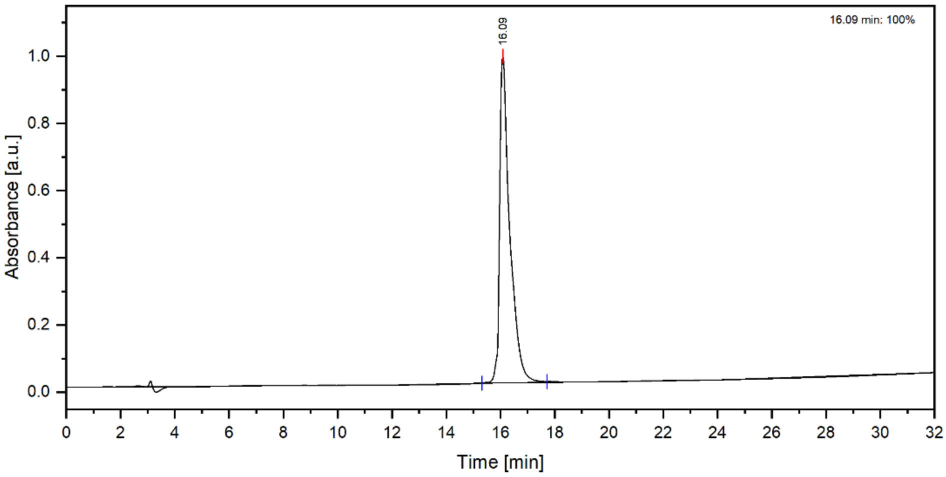

Figure S102: Analytical RP-HPLC chromatogram (System B) of compound 4.

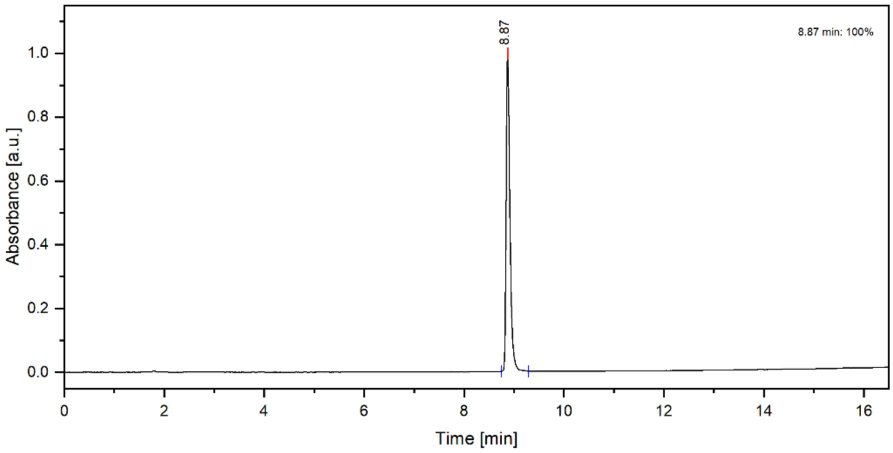

Figure S103: Analytical RP-HPLC chromatogram (System A) of compound 46.

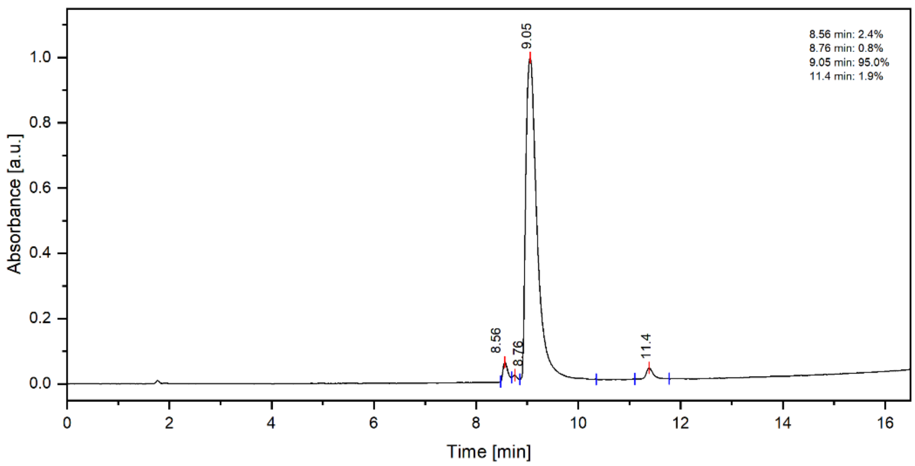

Figure S104: Analytical RP-HPLC chromatogram (System A) of compound 3.

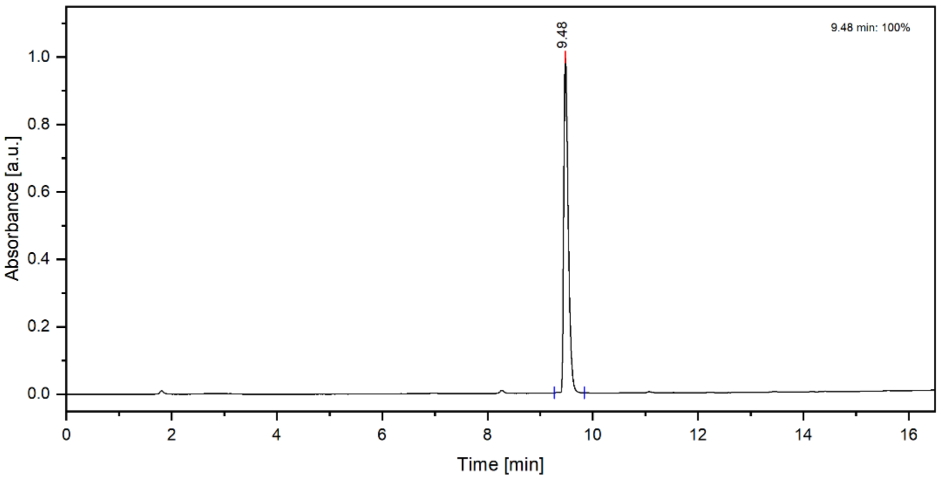

Figure S105: Analytical RP-HPLC chromatogram (System A) of compound 47.

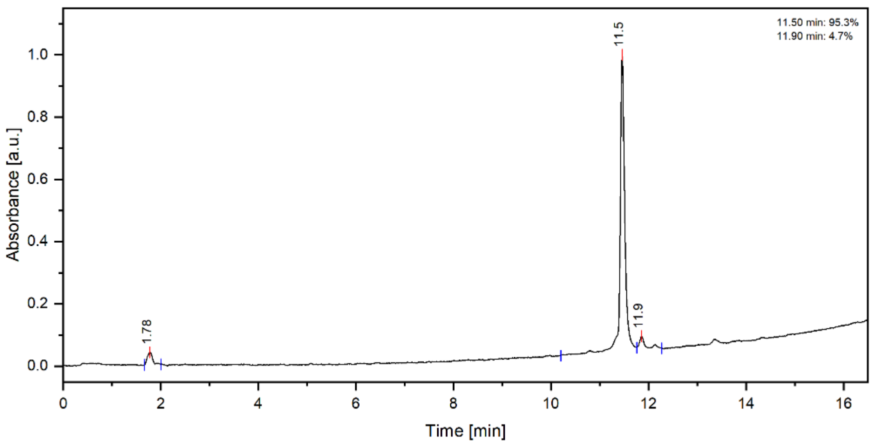

Figure S106: Analytical RP-HPLC chromatogram (System A) of compound 11.

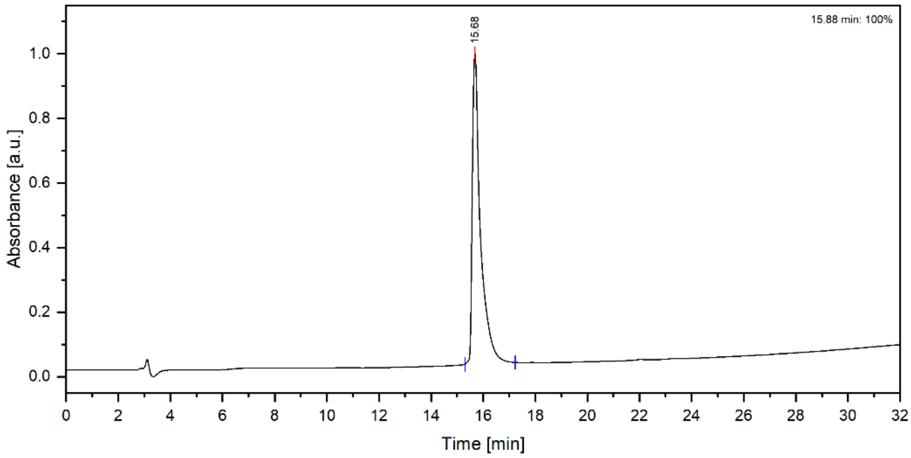

Figure S107: Analytical RP-HPLC chromatogram (System B) of compound 5.

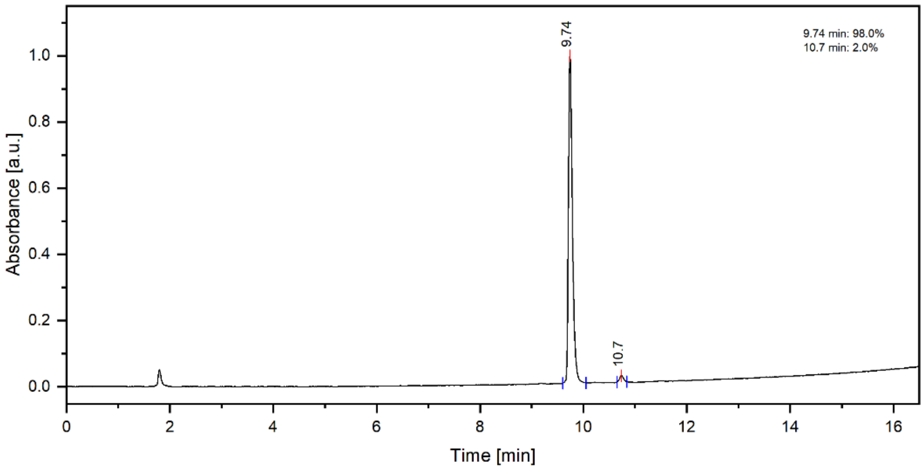

Figure S108: Analytical RP-HPLC chromatogram (System A) of compound 42.

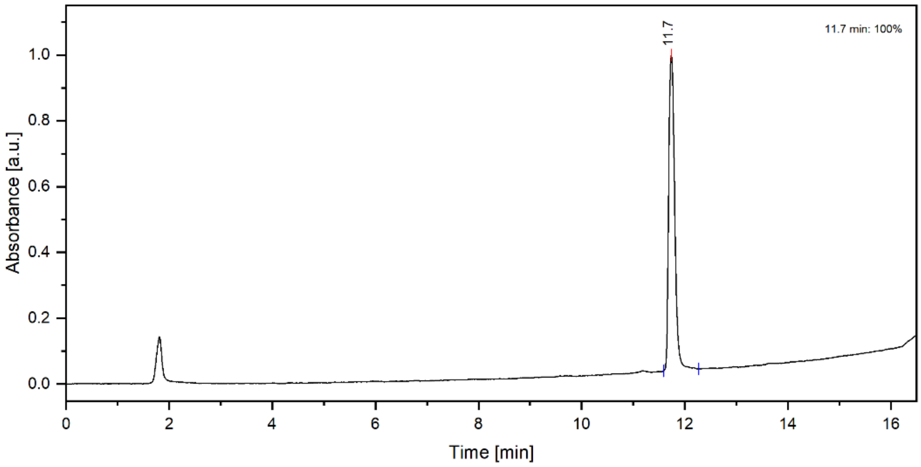

Figure S109: Analytical RP-HPLC chromatogram (System A) of compound 12.

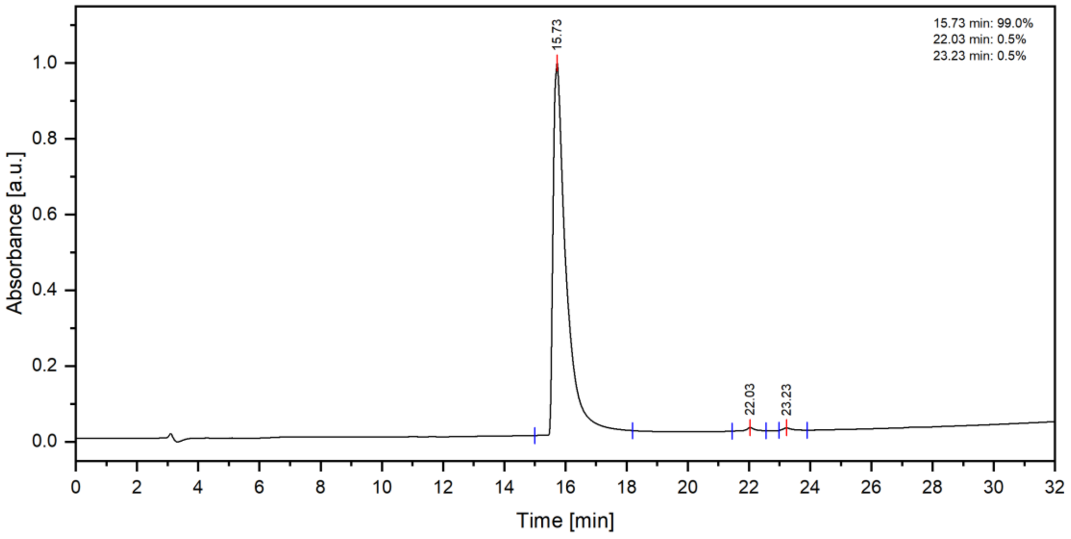

Figure S110: Analytical RP-HPLC chromatogram (System B) of compound 6.

6. Radio-HPLC Chromatograms

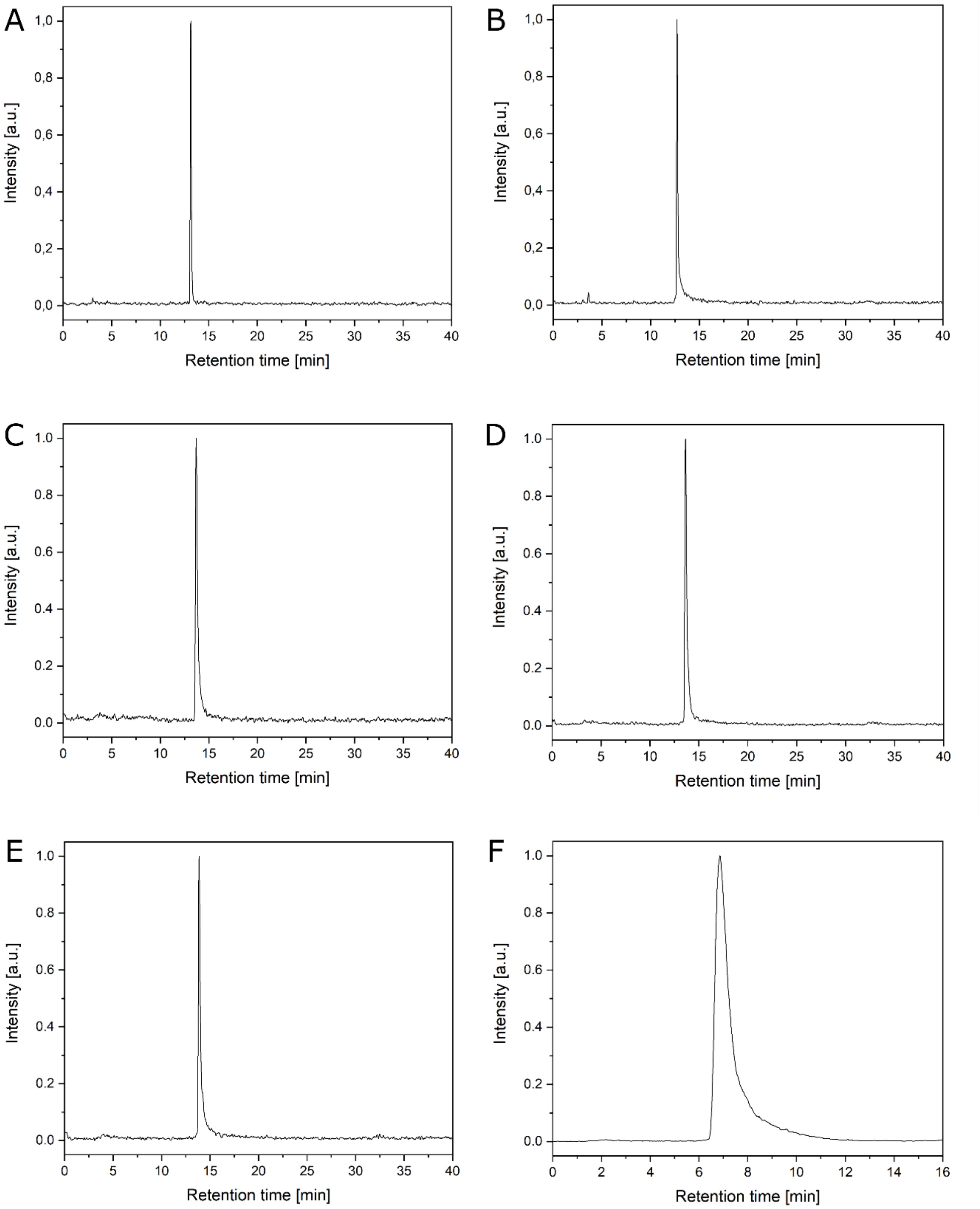

Figure S111: Radio-HPLC chromatograms of ^{64}Cu-labeled compounds A) [^{64}Cu]Cu-1 (System C), B) [^{64}Cu]Cu-2 (System C), C) [^{64}Cu]Cu-3 (System C), D) [^{64}Cu]Cu-4 (System C), E) [^{64}Cu]Cu-5 (System C) and F) [^{64}Cu]Cu-6 (System D).

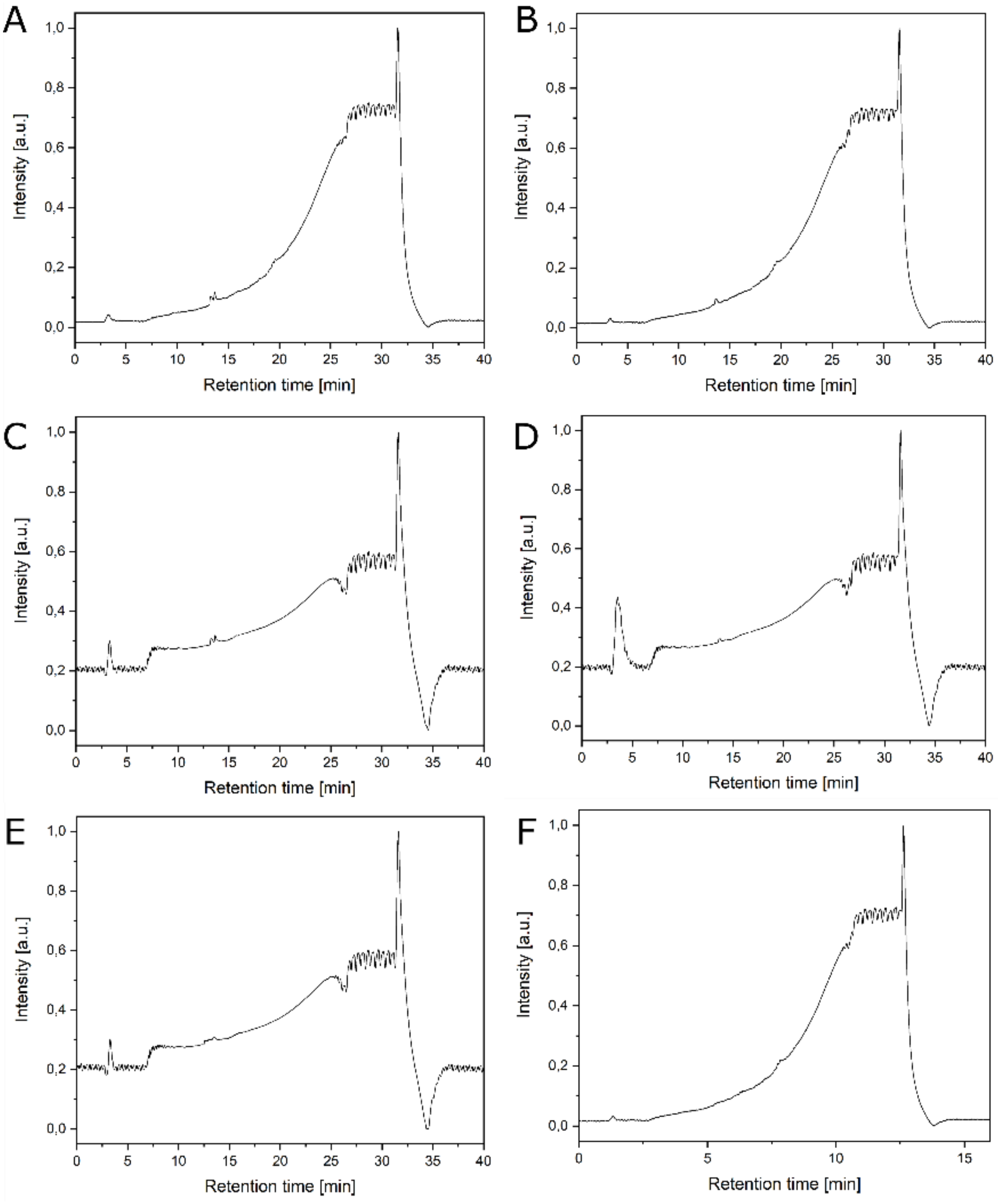

Figure S112: UV-Traces of HPLC chromatograms of ^{64}Cu-labeled compounds A) [^{64}Cu]Cu-1 (System C), B) [^{64}Cu]Cu-2, C) [^{64}Cu]Cu-3 (System C), D) [^{64}Cu]Cu-4, E) [^{64}Cu]Cu-5 (System C) and F) [^{64}Cu]Cu-6 (System D).

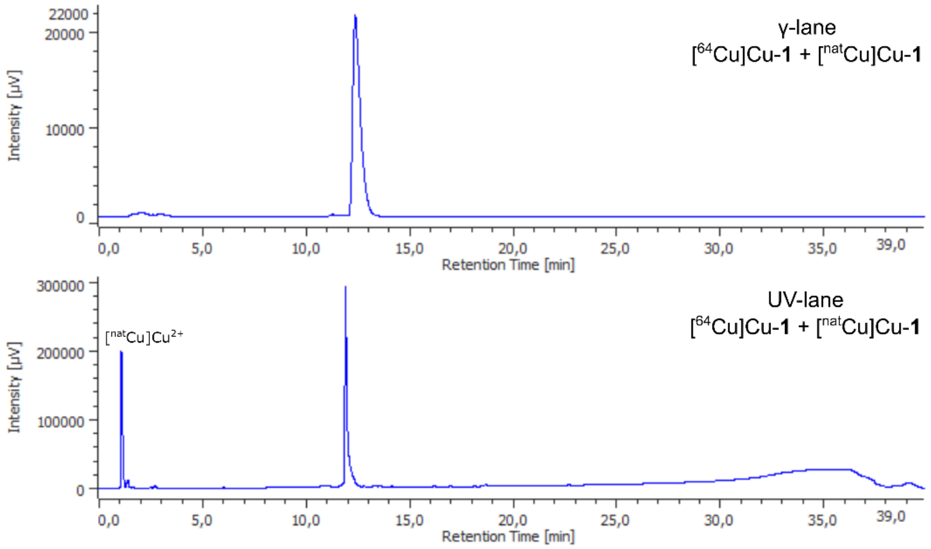

Figure S113: Upper trace: γ-lane of HPLC chromatogram of mixture [^{64}Cu]Cu-1 + [^{nat}Cu]Cu-1 (System C); lower trace: UV-lane of HPLC of HPLC chromatogram of mixture [^{64}Cu]Cu-1 + [^{nat}Cu]Cu-1 (System C).

7. Stability of radiolabeled compounds

7.1 Kinetic stability in PBS (pH 7.4)

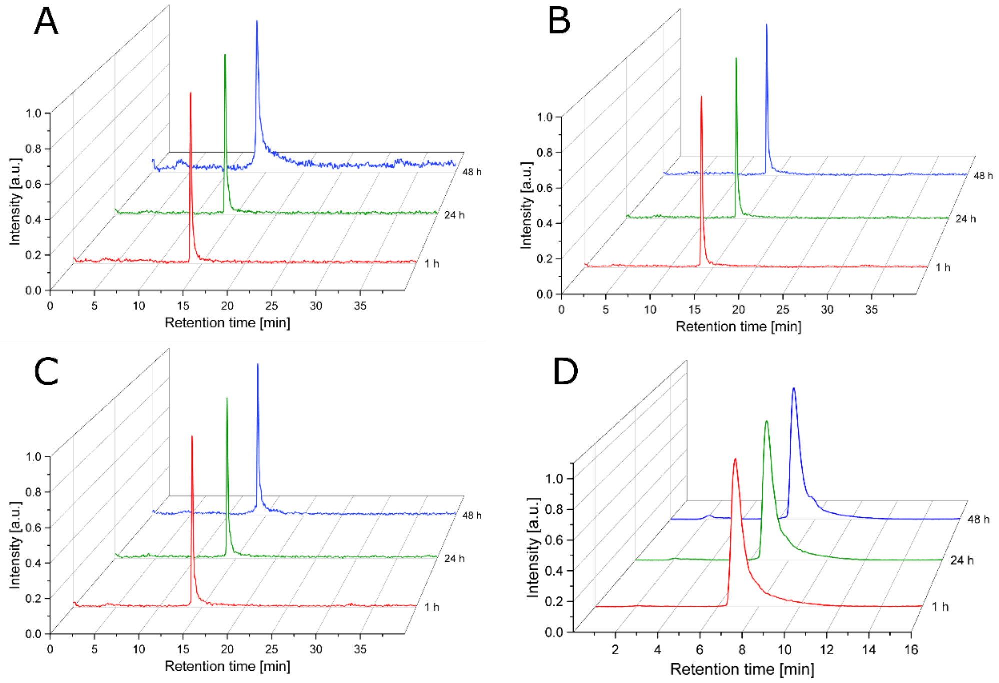

Figure S114: Radio-HPLC chromatograms of ^{64}Cu-labeled compounds A) [^{64}Cu]Cu-3 (System C); B) [^{64}Cu]Cu-4 (System C); C) [^{64}Cu]Cu-5 (System C); D) [^{64}Cu]Cu-6 (System D) after incubation in PBS solution at room temperature for 1 h (red), 24 h (green) and 48 h (blue).

7.2 Proteolytic stability in human serum

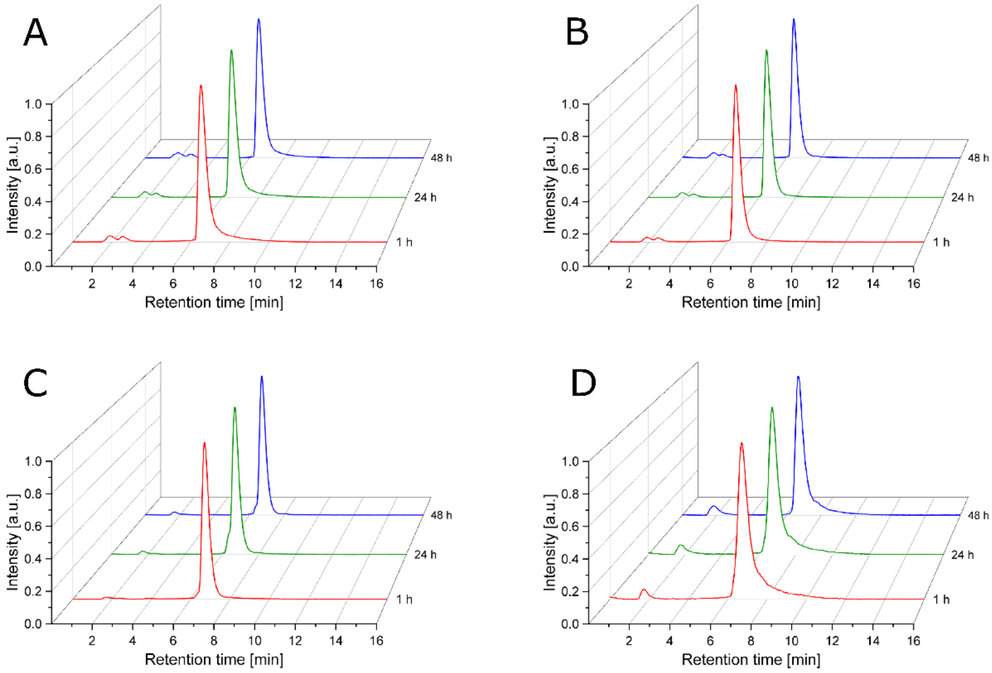

Figure S115: Radio-HPLC chromatograms of ^{64}Cu-labeled compounds (System D) A) [^{64}Cu]Cu-3; B) [^{64}Cu]Cu-4; C) [^{64}Cu]Cu-5; D) [^{64}Cu]Cu-6 after incubation in human serum at 37 °C and subsequent protein precipitation for 1 h (red), 24 h (green) and 48 h (blue).

8. *In vitro*: FACS analysis of PC3 PD-L1 positive and mock cells, immunostaining of tumor xenografts

Methodology:

FACS

Live cells were harvested using trypsin (0.05% Trypsin-EDTA, Gibco, USA), centrifuged, and washed. Thereafter, cells were resuspended and fixed in 4% formaldehyde for 15 minutes at room temperature (RT). Following centrifugation (300g, 3 min) and washing in phosphate buffered saline (PBS), cells were resuspended in PBS. Cells were then chilled on ice and ice-cold methanol added to a final concentration of 90%. Permeabilization in methanol was performed for 10 minutes on ice, followed by adding excess PBS and centrifugation (500g, 3 min). Resuspended cells were counted using a cell counter (CASY1 Model TT, Schaerfe System, Reutlingen, Germany) and approximately 50,000 cells used for staining. These cells were then incubated in 100 µl of diluted primary antibody [monoclonal α-human PD-L1, Rabbit IgG #E1L3N Cell Signaling (Danvers, MA, USA), 874 µg/mL; 1:1,000 in antibody dilution buffer] for 1 hour at RT. Following a double wash step with antibody dilution buffer, cells were resuspended in 100 µl of diluted fluorochrome-conjugated secondary antibody [Rabbit IgG (H+L) Cross-Adsorbed Secondary Antibody, Alexa Fluor 647, 2 mg/mL, 1:500; in antibody dilution buffer) for 30 min at RT under light shielding. After washing twice in antibody dilution buffer, cells were resuspended in 200 µl PBS and analyzed on a flow cytometer (Attune NxT, Thermo Fisher Scientific). The respective cell population was chosen by forward (FSC) vs. side scatter (SSC); while forward scatter height (FSC-H) vs. forward scatter area (FSC-A) was used to exclude doublets, dead cells, and debris.

Immunostaining

Tumors of animals approaching endpoints were excised immediately following euthanasia. Tissue was immersion-fixed in 4% paraformaldehyde for 48 h and stored in PBS (+0.05% sodium azide; w/v). Samples were then processed, embedded in paraffin and 4 µm sections adhered to SuperFrost+ slides (Fisher Scientific, Germany). Slides were stored for not more than 8 weeks. Following dewaxing in RotiHistol (Carl Roth, Karlsruhe, Germany), slides were rehydrated in a descending concentration series of ethanol (100, 96, 85, 70, 50% (v/v), H_2O). Antigen retrieval was performed using 10 mM citrate buffer (pH 6.0) in a steam bath. Sections were then permeabilized and endogenous peroxidases quenched with 3% (v/v) H_2O_2 in Tris-buffered saline [with 0.1% Tween 20 (TBS-T]. Tissue was then blocked with 10% (w/v) fetal bovine serum in TBS-T.

Incubation with the primary antibody [monoclonal α-human PD-L1, Rabbit IgG #E1L3N Cell Signaling (Danvers, MA, USA), 874 µg/mL; 1:20,000] followed overnight at 4°C in a humidified chamber. Primary antibody was detected using a 2-step polymer detection kit (SuperVision 2 HRP-Polymer, DCS Innovative Diagnostik-Systeme, Hamburg, Germany) with 3,3’-diaminobenzidine (DAB) as chromogen, according to the manufacturers protocol. Sections were counterstained with Mayer’s hematoxylin and coverslipped.

Slides were imaged on an AXIO Imager A1 microscope (Carl Zeiss Microscopy, Oberkochen, Germany) using Zeiss EC Plan-NEOFLUAR objectives (10x/0.3 Ph1 and 20x/0.5 Ph2) and an Axiocam MRc (CCD, 1388x1040 pixel).

Prior to this, positive (human placenta) and negative controls (omission of primary/secondary antibodies) were performed in a separate experiment, confirming antibody specificity and staining conditions.

Results:

As demonstrated in Suppl. Figure S116 (A-C, left side vs forward scatter plots), all three tested cell types (derived from the same parent cell line) yielded very similar results (such as viability, debris) in flow cytometry. Of the gated cells, over 99% of PC3 cell previously transduced for PD-L1, were found to express the target (right single parameter histogram, Suppl. Figure S116-A). In contrast, gated cells either previously transduced with a mock construct (Suppl. Figure S116-B) or of wild type (Suppl. Figure S116-C), did not exhibit any Alexa 674 fluorescence (below 0.1%), indicating no bound antibody and therefore no PD-L1 expression.

Similarly, immunostaining of random tumor xenografts confirmed FACS data of cultured cells. Using the same antibody on FFPE tissue sections derived from PC3 PD-L1 xenografted cells, resulted in a strong and ubiquitous staining across the whole tumor (Suppl. Figure S116-D) and individual cells (Suppl. Figure S116-F). Once again, tumors grown from cells expressing a mock construct showed an almost complete absence of immunoreactivity in the whole tumor (Suppl. Figure S116-E), with single cells showing a faint staining.

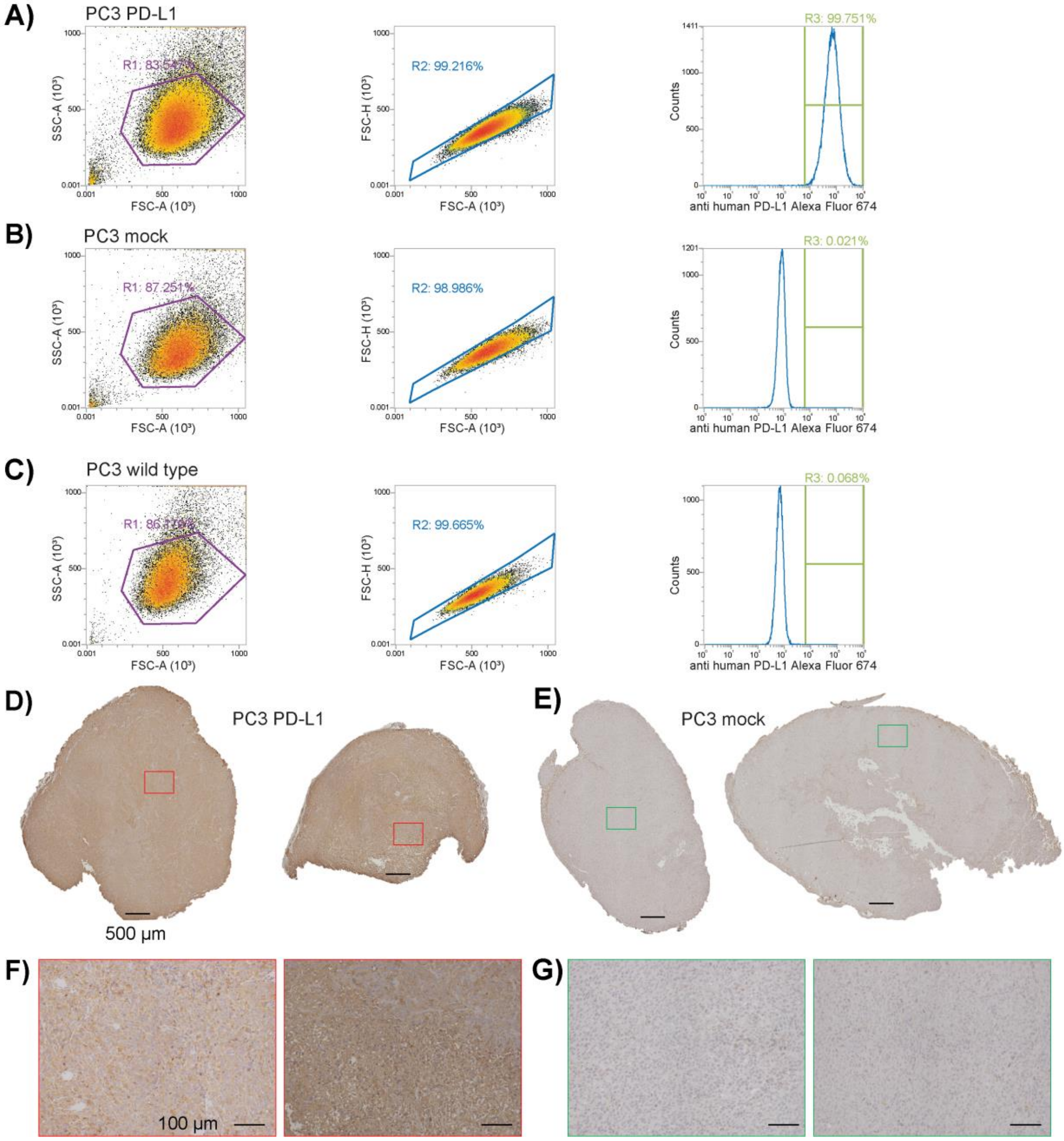

Figure S116: FACS analysis of approximately 50000 PC3 cells, transduced with human PD-L1 (A), mock construct (B) and PC3 wild-type cells showing stable overexpression (A, PC3 PD-L1) and absence (B, PC3 mock; C, PC3 wild-type) of the target in respective cells. Data shows plots of forward vs. side scatter (left side plots) and forward scatter height (FSC-H) vs. forward scatter area (center plots), along with gating. Single parameter histograms for Alexa 674 fluorescence (detection of the primary monoclonal anti human PD-L1 antibody) are presented on the right. Similarly, random tumors micrographs (10x), immunostained using the same antibody, confirmed target overexpression in tumors grown from PC3 PD-L1 cells (D), while tumors grown from PC3 mock cells lacked immunoreactivity (E). Higher magnification (20x) micrographs from the PC3 PD-L1 (F) and mock tumors (G).

9. *In vitro*: Saturation binding assays

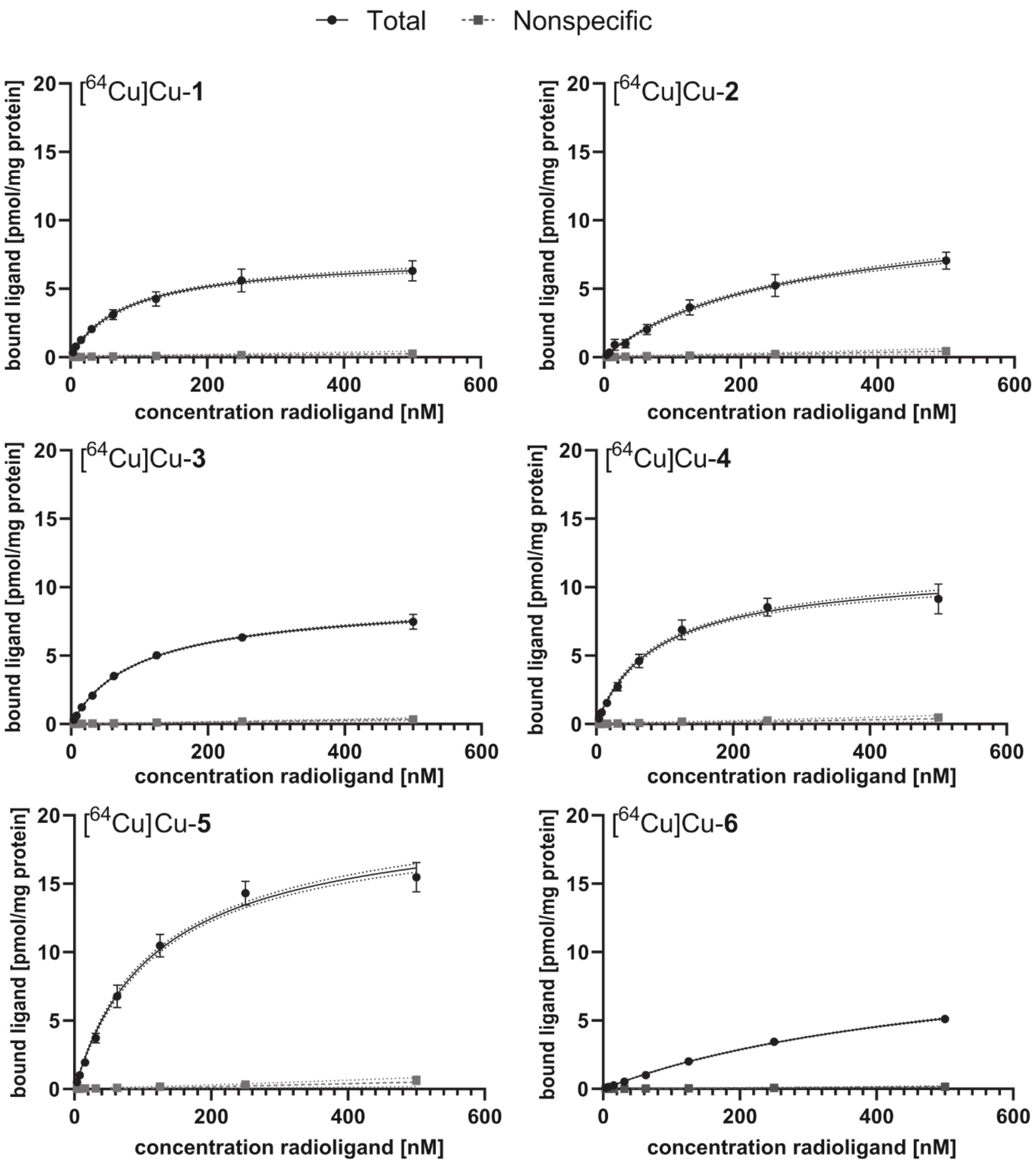

Figure S117: Nonlinear iterative curve fitting of saturation binding experimental data of compounds [^{64}Cu]Cu-1-6. Curves show representative fits for total and nonspecific binding of three individual experiments combined, along with 95% confidence levels (dotted lines).

10. *In vitro*: Real-time binding assay

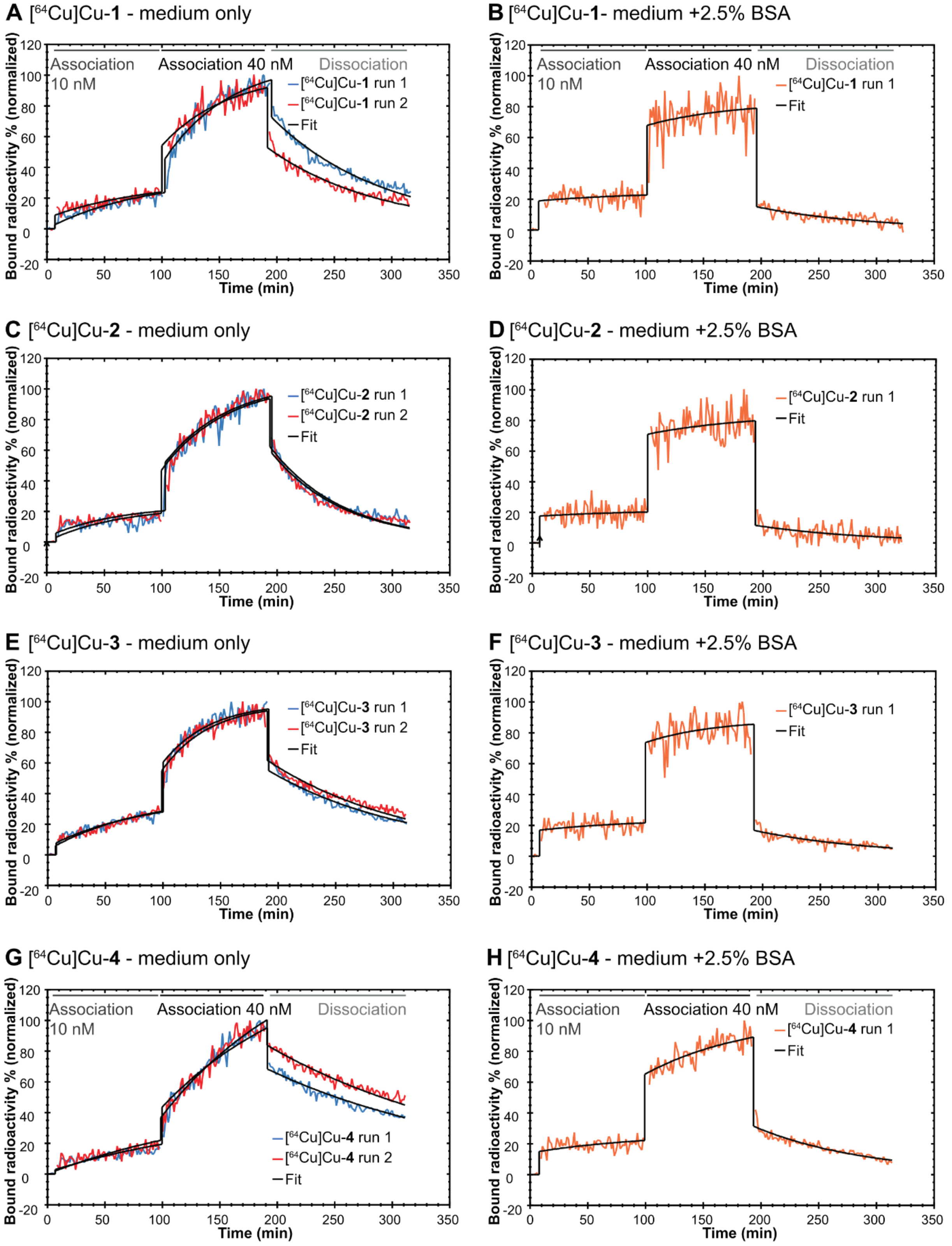

Figure S118: Real-time radioligand binding (trace) of compounds [^{64}Cu]Cu-1-4 in absence(A/C/E/G) and presence (B/D/F/H) of 2.5% bovine serum albumin (BSA). Kinetic parameters (association rate constant k_a, dissociation rate constant k_d and dissociation constant K_D) are reported in Table 2.

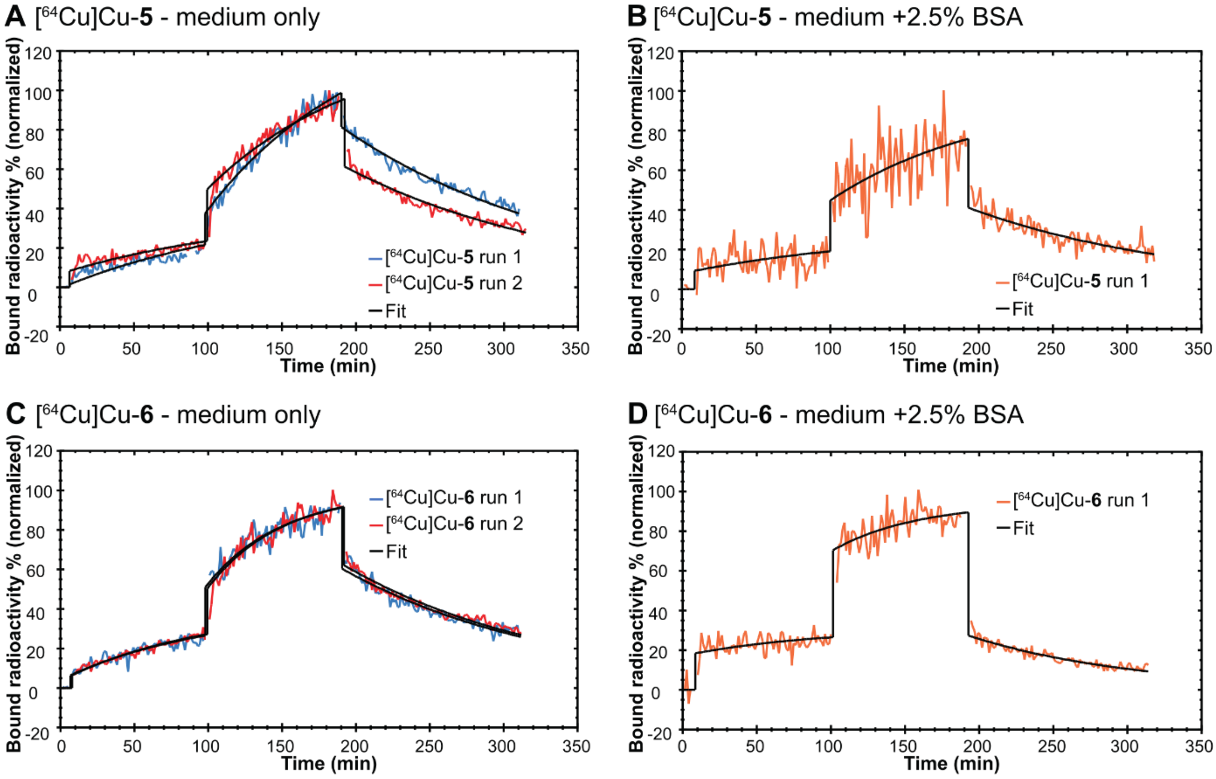

Figure S119: Real-time radioligand binding (trace) of compounds [^{64}Cu]Cu-5 and 6 in absence(A/C) and presence (B/D) of 2.5% bovine serum albumin (BSA). Kinetic parameters (association rate constant k_a, dissociation rate constant k_d and dissociation constant K_D) are reported in Table 2.

11. *In vivo*: PET MIP images

Third series

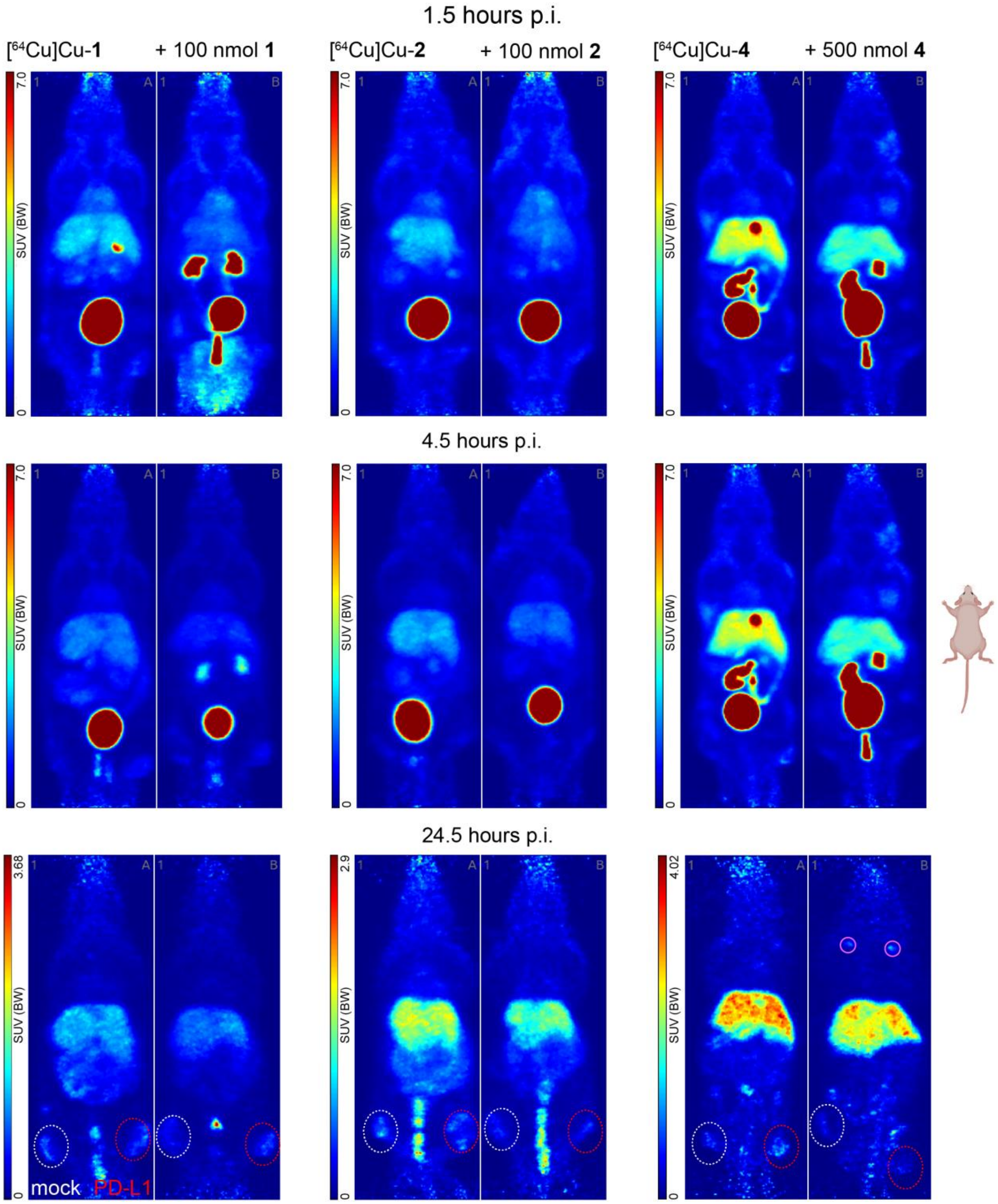

Figure S120: ***In vivo*** **distribution (maximum intensity projections) of ^{64}Cu-labeled third series compounds [^{64}Cu]Cu-1, 2 and 4 at 1.5 (1-2), 4.5 (4–59 and 24.5 (24-25) hours post injection (p.i.). SUV scales differ for 24.5 hour timepoint. Dashed circles indicate PC3 mock (white) or PC3 PD-L1 (red) tumors. Solid purple circles indicate bone/bone marrow tracer uptake for compound [^{64}Cu]Cu-4.**

Third series

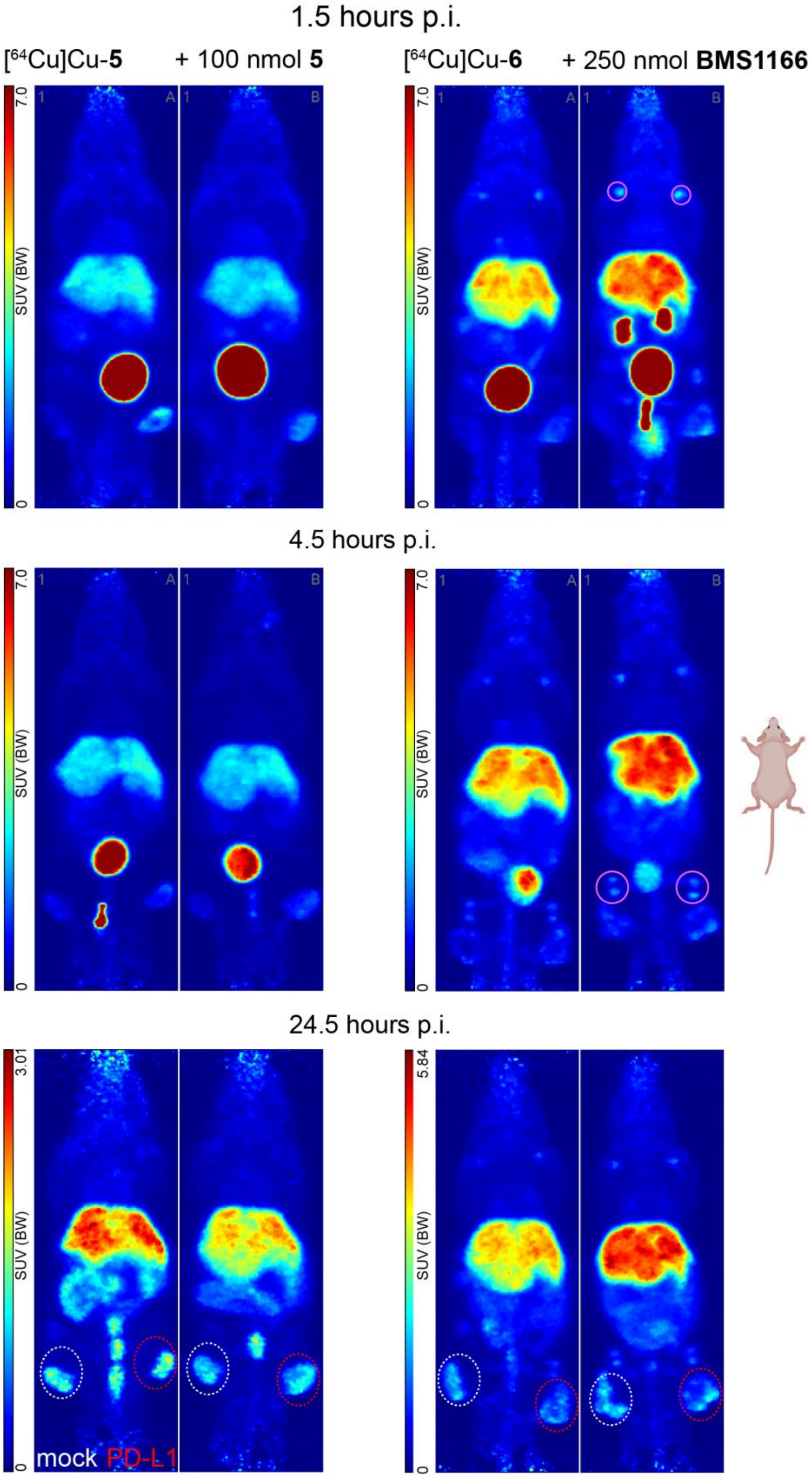

Figure S121: : ***In vivo*** **distribution (maximum intensity projections) of ^{64}Cu-labeled third series compounds [^{64}Cu]-5 and 6 at 1.5 (1-2), 4.5 (4–5) and 24.5 (24-25) hours post injection (p.i.). SUV scales differ for 24.5 hour timepoint Dashed circles indicate PC3 mock (white) or PC3 PD-L1 (red) tumors. Solid purple circles indicate bone/bone marrow tracer uptake for compound [^{64}Cu]Cu-6.**

Radiotracer production	Name of Compound	[^{64}Cu]Cu-**1**
	Starting amount radioactivity	~ 100 MBq
	Radioactivity amount of the Product	~ 97 MBq
	Activity Yield (AY)[1]	~ 97 MBq
	RCY (decay corrected)[2]	>99%
	Radiotracer Production time (including radiolabeling, purification, isolation, and reformulation)	30 min
	RCP	>99%
	Stability of the product to in vivo experiments	>99%
	A_m and reference time point (e.g. EOS)[3]	12.5 GBq/µmol
	Formulation	none
	Number of experiments	n = 5
In vivo	Species	Mouse, NMRI-nude mice (Rj:NMRI-Foxn1$^{nu/nu}$)
	A_m at start of in vivo study	~12
	Injected Radioactivity (MBq)	10.63±0.95 (PET, mean ± SD)
	Time between EOS and start of in vivo study	~20 min
	Number of animals	2

Radiotracer production	Name of Compound	[^{64}Cu]Cu-**2**
	Starting amount radioactivity	~ 100 MBq
	Radioactivity amount of the Product	~ 97 MBq
	Activity Yield (AY)[1]	~ 97 MBq
	RCY (decay corrected)[2]	>99%
	Radiotracer Production time (including radiolabeling, purification, isolation, and reformulation)	30 min
	RCP	>99%
	Stability of the product to in vivo experiments	>99%
	A_m and reference time point (e.g. EOS)[3]	12.5 GBq/µmol
	Formulation	none
	Number of experiments	n = 5
In vivo	Species	Mouse, NMRI-nude mice (Rj:NMRI-Foxn1$^{nu/nu}$)
	A_m at start of in vivo study	~12
	Injected Radioactivity (MBq)	10.97±0.39 (PET, mean ± SD)
	Time between EOS and start of in vivo study	~20 min
	Number of animals	2

Radiotracer production	Name of Compound	[^{64}Cu]Cu-**3**
	Starting amount radioactivity	~ 100 MBq
	Radioactivity amount of the Product	~ 97 MBq
	Activity Yield (AY)[1]	~ 97 MBq
	RCY (decay corrected)[2]	>99%
	Radiotracer Production time (including radiolabeling, purification, isolation, and reformulation)	30 min
	RCP	>99%
	Stability of the product to in vivo experiments	>99%
	A_m and reference time point (e.g. EOS)[3]	12.5 GBq/µmol
	Formulation	none
	Number of experiments	n = 15
In vivo	Species	Mouse, NMRI-nude mice (Rj:NMRI-Foxn1$^{nu/nu}$)
	A_m at start of in vivo study	~12
	Injected Radioactivity (MBq)	12.41±0.74 (PET, mean ± SD)<br>1.75±0.09 (Biodistribution, mean ± SD)<br>51.73±3.29 (Metabolites, mean ± SD)
	Time between EOS and start of in vivo study	~30 min
	Number of animals	4 (PET)<br>12 (Biodistribution)<br>3 (Metabolites)

Radiotracer production	**Name of Compound**	[^{64}Cu]Cu-**4**
	**Starting amount radioactivity**	~ 100 MBq
	**Radioactivity amount of the Product**	~ 97 MBq
	**Activity Yield (AY)[1]**	~ 97 MBq
	**RCY (decay corrected)[2]**	>99%
	**Radiotracer Production time (including radiolabeling, purification, isolation, and reformulation)**	30 min
	**RCP**	>99%
	**Stability of the product to in vivo experiments**	>99%
	**A_m and reference time point (e.g. EOS)[3]**	12.5 GBq/µmol
	**Formulation**	none
	**Number of experiments**	n = 5
In vivo	**Species**	Mouse, NMRI-nude mice (Rj:NMRI-Foxn1$^{nu/nu}$)
	**A_m at start of in vivo study**	~12
	**Injected Radioactivity (MBq)**	10.05±0.16 (PET, mean ± SD)
	**Time between EOS and start of in vivo study**	~20 min
	**Number of animals**	4

Radiotracer production	**Name of Compound**	[^{64}Cu]Cu-**5**
	**Starting amount radioactivity**	~ 100 MBq
	**Radioactivity amount of the Product**	~ 97 MBq
	**Activity Yield (AY)[1]**	~ 97 MBq
	**RCY (decay corrected)[2]**	>99%
	**Radiotracer Production time (including radiolabeling, purification, isolation, and reformulation)**	30 min
	**RCP**	>99%
	**Stability of the product to in vivo experiments**	>99%
	**A_m and reference time point (e.g. EOS)[3]**	12.5 GBq/µmol
	**Formulation**	none
	**Number of experiments**	n = 5
In vivo	**Species**	Mouse, NMRI-nude mice (Rj:NMRI-Foxn1$^{nu/nu}$)
	**A_m at start of in vivo study**	~12
	**Injected Radioactivity (MBq)**	10.61±0.89 (PET, mean ± SD)
	**Time between EOS and start of in vivo study**	~25 min
	**Number of animals**	4

Radiotracer production	**Name of Compound**	[^{64}Cu]Cu-**6**
	**Starting amount radioactivity**	~ 100 MBq
	**Radioactivity amount of the Product**	~ 97 MBq
	**Activity Yield (AY)[1]**	~ 97 MBq
	**RCY (decay corrected)[2]**	>99%
	**Radiotracer Production time (including radiolabeling, purification, isolation, and reformulation)**	30 min
	**RCP**	>99%
	**Stability of the product to in vivo experiments**	>99%
	**A_m and reference time point (e.g. EOS)[3]**	12.5 GBq/µmol
	**Formulation**	none
	**Number of experiments**	n = 5
In vivo	**Species**	Mouse, NMRI-nude mice (Rj:NMRI-Foxn1$^{nu/nu}$)
	**A_m at start of in vivo study**	~12
	**Injected Radioactivity (MBq)**	9.97±0.69 (PET, mean ± SD)
	**Time between EOS and start of in vivo study**	~30 min
	**Number of animals**	4

12. *In vivo*: Dynamic [^{18}F]FDG uptake as surrogate measure of blood flow

Methodology:

PET and data analysis:

Injections of PC3 PD-L1 and mock cells were performed under the same conditions outlined in the Material and Methods section. Similarly, preparation of animals for PET scans was also identical to procedures used for copper-64 labeled PD-L1 radiotracers.

[^{18}F]FDG was obtained from regular in house production for clinical use, meeting all requirements for human injection. Three male athymic NMRI-nude mice, bearing each one PC3 PD-L1 (Length: 7.03±0.72 mm, Width: 5.78±0.75 mm) and mock tumor (L: 7.9±2.12 mm, W: 6. 38±1.11 mm) were injected i.v. with 9.86±0.25 MBq (mean±SD) [^{18}F]FDG over 30 seconds, with PET acquisition started simultaneously. Data was reconstructed similar to dynamic scans for PD-L1 radiotracers, with 15x10 s, 5x30 s, 5x60 s, 4x300 s, 9x600 s and 3x1200 s frames. Three-dimensional volumes of interest were drawn over the tumors, applying a fixed threshold at 35 % of the measured maximum intensity. SUV_{mean} was extracted for each frame and graphed used a 4-knot smoothing spline algorithm (GraphPad Prism 9.4.1). SUV_{mean} data from three PC3 PD-L1 and three PC3 mock tumors was tested using a mixed-effects analysis with Geisser-Greenhouse correction for sphericity. Factors were time (seconds post injection) and [^{18}F]FDG uptake per tumor entity.

Results:

While [^{18}F]FDG is not a perfusion marker, such as [^{15}O]H_2O or [^{13}N]ammonia, it shows a linear correlation with [^{15}O]H_2O measured blood flow in tumors, primarily in the first minutes post injection, as previously reported.[3]

Over the whole period of three hours, [^{18}F]FDG uptake in both tumors developed similarly (Suppl. Figure S122-A/C), although PD-L1 tumors (Suppl. Figure S122-A/C, left) exhibited a slightly higher variation. This was also observed when just the first 10 minutes were investigated. A mixed-effects analysis for the full three hours with time (seconds post injection) and [^{18}F]FDG uptake per tumor entity (SUV_{mean}) as factors was performed. A significant main effect of time [$F(1.491, 5.962) = 118.4$, $P<0.0001$] was found. However, [^{18}F]FDG uptake per tumor entity was not significantly different [$F(1, 4) = 0.7974$, $P=0.4223$]. Hence, no post-hoc test was performed. When only the first 10 minutes were investigated, again a significant main effect of time [$F(1.905, 7.622) = 90.52$, $P<0.0001$] was observed but none for [^{18}F]FDG uptake per tumor entity [$F(1, 4) = 0.9042$, $P=0.3955$]. It can therefore be concluded that blood flow into both tumors is not significantly different and the tracer uptake into PC3 PD-L1 tumors is target specific.

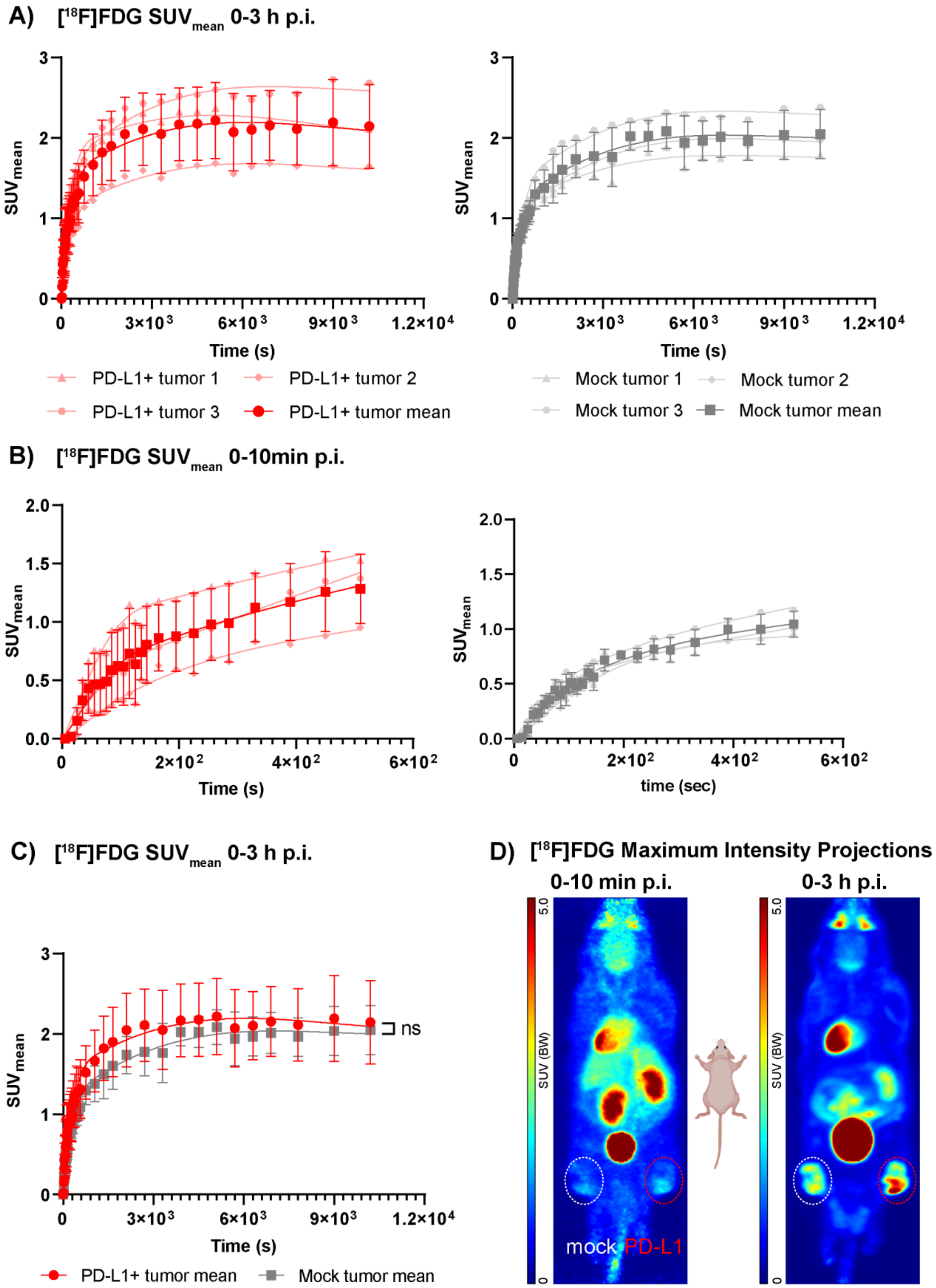

Figure S122: Time activity curves (TAC) showing i*n vivo* uptake ($SUV_{mean)}$ of [^{18}F]FDG into either PC3 PD-L1 overexpressing or mock tumors (n=3 each). Data is reported for 0-3 h post injection for PD-L1 positive (A, left) and mock tumors (A, right) and at 0-10 min p.i. (B). Mean uptake of [^{18}F]FDG for PC3 PD-L1 overexpressing tumors is not significantly different from mock tumors (C), as tested with a mixed-effects analysis. D) Maximum intensity projection PET images at 0-10 min (left) and 0-3 h p.i. in the same animal for mock (white) or PC3 PD-L1 overexpressing (red) tumors.

13. *In vivo*: metabolization of [^{64}Cu]Cu-3

Methodology:

Under desflurane anesthesia, three naive NMRI nude mice were i.v. injected with 51.7±3.29 MBq [^{64}Cu]Cu-**3** (at 16.5 GBq/µmol A_M; equals 4.786±0.30 µg).

In parallel, [^{64}Cu]Cu-**3** was immediately injected into a HPLC system (Hewlett Packard Series 1200; detector: Raytest Ramona; column: Zorbax C18 300SB 9,4 x250mm 4µm) and ran using H_2O + 0,1% TFA/MeCN + 0.1% TFA. Additionally, [^{64}Cu]Cu-**3** was left at room temperature for ~8 hours and ran again on the same system. This served as a reference for all later measurements and all detected activities were referenced to the time of the first HPLC run.

After designated timepoints (5 min, 60 min and 4 hours post injection), animals were euthanized under anesthesia, blood was collected via cardiac puncture using lithium heparinized syringes and tubes (Heparin-Sodium LEO 25.000 IU/5 mL). The aliquot was centrifuged (13000 g for 3 min) and proteins were precipitated in a plasma aliquot using "Supersol" in a ratio of 1:2. Urine was collected from the bladder and treated identically. The respective supernatant was then injected into the same HPLC system and analyzed.

Other target organs (liver, kidney) were removed, transferred into a 10 mL vial along with 1 mL of PBS. Tissue was then homogenized for 3 min on ice at 8700 rpm, using a T25 Ultra Turrax. The tissue slurry was transferred into 2 mL tubes and centrifuged at 13500 rpm at 4°C) for 3 mL. Again, proteins were precipitated using "Supersol" and injected into HPLC.

Results:

Ex vivo metabolite analysis [^{64}Cu]Cu-**3** in blood, urine, liver and kidneys, along with HPLC references is presented in Suppl. Figure S123 (A-E). As shown before (Suppl. Figure S114), [^{64}Cu]Cu-**3** was stable in injection buffer for several hours (Suppl. Figure S123-E, t_R = 13.1 min). The main identifiable metabolite observed was free copper (t_R = 3.8 min), released from the NODAGA-chelator. This was verified when compared with [^{64}Cu]$CuCl_2$, injected separately. Blood and urine contained mostly intact tracer at 5 min, 60 min and 4 hours post injection (Suppl. Figure S123-A/D, quantification in F). Metabolization (transchelation) was highest in liver and kidney (Suppl. Figure S123-B/D, quantification in F) with 45 and 39% intact (copper-bearing) tracer at 4 hours post injection. Taken together, this data shows that [^{64}Cu]Cu-**3** metabolization (transchelation) in the first hours is restricted to excreting organs, while the tracer in circulation is mostly intact. Furthermore, the only observed radiometabolite is free copper, hence interference with tumor uptake is unlikely.

[^{64}Cu]Cu-**3** metabolization

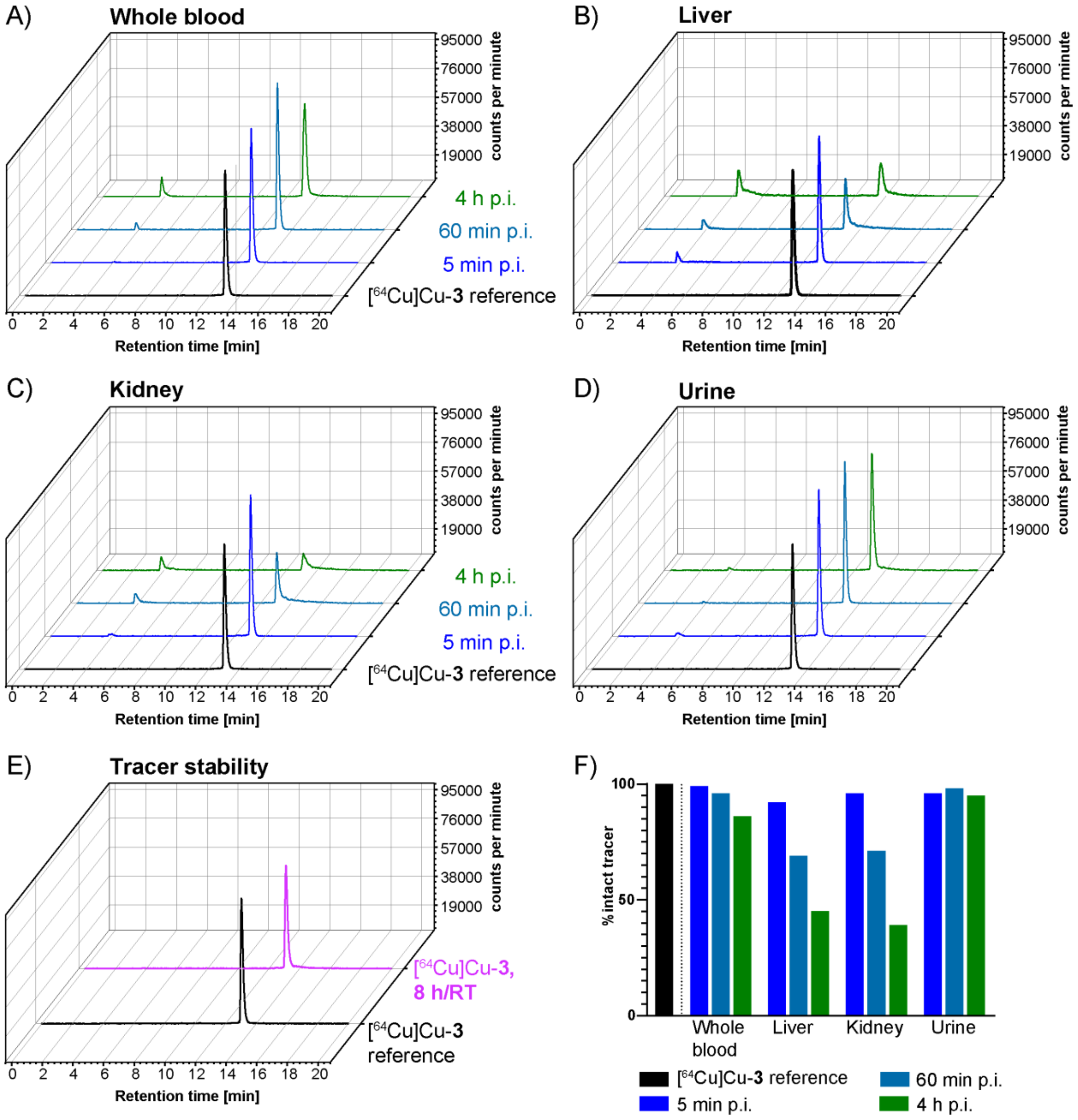

Figure S116: A–D) Radio-HPLC chromatograms of metabolism studies (A: whole blood, B: liver, C: kidney, D: urine) for [^{64}Cu]Cu-3 after 5 min, 60 min and 4 h p.i.; E) Reference radio-HPLC chromatogram of [^{64}Cu]Cu-3 before animal injection and after incubation for 8 hours at room temperature in reaction medium; F) Quantification of stability by percentage of intact radiotracer in whole blood, liver, kidney and urine at all three time point.

14. *In vivo*: PET- SUV data

Standardized uptake values (SUV_{max})

Supplementary table 1: *In vivo* standardized uptake values (SUV_{max} ± standard deviation) of ^{64}Cu-labeled third series compounds [^{64}Cu]Cu-1-6 at 1.5 (1–2), 4.5 (4–5) and 24.5 (24–25) hours post injection (p.i.). Compounds used for blocking are described for each tracer and were intravenously injected in sterile saline at stated time before the radiotracer. Animals designated for tracer-only received matched type and volume of vehicle at the same time.

**Compound (# of animals)**		SUV_{max} 1.5 (1–2) hours p.i.		4.5 (4–5) hours p.i.		24.5 (24–25) hours p.i.		Blocking conditions
		Compound	+block	Compound	+block	Compound	+block	
[^{64}Cu]Cu-**1** (n=1)	PC3 PD-L1 overexpressing tumor	1.5	1.3	1.1	1.0	1.4	1.1	100 nmol **1** ~5 min prior to radiotracer
	PC3 mock tumor	1.0	1.4	0.8	1.0	1.2	0.7	
[^{64}Cu]Cu-**2** (n=1)	PC3 PD-L1 overexpressing tumor	1.2	1.2	0.7	0.7	1.1	0.7	100 nmol **2** ~5 min prior to radiotracer
	PC3 mock tumor	1.1	1.1	0.7	0.7	1.3	0.6	
[^{64}Cu]Cu-**3** (n=2)	PC3 PD-L1 overexpressing tumor	3.5 ± 0.2	1.0 ± 0.1	2.6 ± 0.1	0.7 ± 0.3	1.35 ± 0.15	0.65 ± 0.05	1000 nmol **4** ~5 min prior to radiotracer
	PC3 mock tumor	0.85 ± 0.05	0.80 ± 0.1	0.55 ± 0.15	0.55 ± 0.05	0.7 ± 0.2	0.45 ± 0.05	
[^{64}Cu]Cu-**4** (n=2)	PC3 PD-L1 overexpressing tumor	1.97 ± 0.23	0.85 ± 0.05	1.2 ± 0.4	1.05 ± 0.35	2.0 ± 0.1	1.1 ± 0.1	500 nmol **4** ~5 min prior to radiotracer
	PC3 mock tumor	0.8 ± 0.10	0.65 ± 0.05	0.45 ± 0.05	0.6 ± 0.2	1.25 ± 0.15	0.8 ± 0.3	
[^{64}Cu]Cu-**5** (n=2)	PC3 PD-L1 overexpressing tumor	3.25 ± 0.25	1.75 ± 0.35	1.75 ± 0.15	1.65 ± 0.15	1.2 ± 0.8	1.55 ± 0.05	100 nmol **5** ~30 min prior to radiotracer
	PC3 mock tumor	0.80 ± 0.10	0.75 ± 0.05	1.1 ± 0.1	0.85 ± 0.05	1.05 ± 0.65	1.6 ± 0.00	
[^{64}Cu]Cu-**6** (n=2)	PC3 PD-L1 overexpressing tumor	1.83 ± 0.37	2.28 ± 0.46	1.55 ± 0.05	1.8 ± 0.3	2.2 ± 0.1	2.3 ± 0.4	250 nmol **1** ~5 min prior to radiotracer
	PC3 mock tumor	0.83 ± 0.08	1.04 ± 0.15	1.35 ± 0.15	1.7 ± 0.1	2.2 ± 0.2	3.05 ± 0.35	

15. *In vivo*: Body weight

Experiment	Compound	# animals	body weight (g) of animal													
			1	2	3	4	5	6	7	8	9	10	11	12	Mean	SD
PET	[^{64}Cu]Cu-1	2	40.7	38.3											**39.50**	1.70
	[^{64}Cu]Cu-2	2	35.6	37.2											**36.40**	1.13
	[^{64}Cu]Cu-3	4	32.38	27.3	29.17	31.11									**29.99**	2.23
	[^{64}Cu]Cu-4	4	34.33	36.9	34.99	35.84									**35.52**	1.11
	[^{64}Cu]Cu-5	4	34.48	33.14	30.48	35.51									**33.40**	2.18
	[^{64}Cu]Cu-6	4	34.21	32.55	32.15	33.16									**33.02**	0.90
	[^{18}F]FDG	3	35.01	33.2	38.9										**35.70**	2.91
Biodistribution	[^{64}Cu]Cu-3	12	39.08	34.10	33.51	30.26	36.75	39.18	32.44	39.52	37.99	30.77	33.15	33.13	**34.99**	3.35
Metabolites	[^{64}Cu]Cu-3	3	40.42	53.5	41.32										**45.08**	7.31

16. References

(1) Guzik, K.; Zak, K. M.; Grudnik, P.; Magiera, K.; Musielak, B.; Torner, R.; Skalniak, L.; Domling, A.; Dubin, G.; Holak, T. A. Small-Molecule Inhibitors of the Programmed Cell Death-1/Programmed Death-Ligand 1 (PD-1/PD-L1) Interaction via Transiently Induced Protein States and Dimerization of PD-L1. *J. Med. Chem.* **2017**, *60*, 5857-5867. DOI: 10.1021/acs.jmedchem.7b00293.
(2) Chatterjee, S.; Lesniak, W. G.; Miller, M. S.; Lisok, A.; Sikorska, E.; Wharram, B.; Kumar, D.; Gabrielson, M.; Pomper, M. G.; Gabelli, S. B.; et al. Rapid PD-L1 Detection in Tumors with PET Using a Highly Specific Peptide. *Biochem. Biophys. Res. Commun.* **2017**, *483* (1), 258-263. DOI: https://doi.org/10.1016/j.bbrc.2016.12.156.
(3) Mullani, N. A.; Herbst, R. S.; O'Neil, R. G.; Gould, K. L.; Barron, B. J.; Abbruzzese, J. L. Tumor blood flow measured by PET dynamic imaging of first-pass 18F-FDG uptake: a comparison with 15O-labeled water-measured blood flow. *Journal of Nuclear Medicine* **2008**, *49* (4), 517-523.
